# Cobalt-catalyzed highly enantioselective hydrogenation of α,β-unsaturated carboxylic acids

**DOI:** 10.1038/s41467-020-17057-z

**Published:** 2020-06-26

**Authors:** Xiaoyong Du, Ye Xiao, Jia-Ming Huang, Yao Zhang, Ya-Nan Duan, Heng Wang, Chuan Shi, Gen-Qiang Chen, Xumu Zhang

**Affiliations:** 1 Department of Chemistry and Shenzhen Grubbs Institute, Southern University of Science and Technology, 518000 Shenzhen, China; 2 Academy for Advanced Interdisciplinary Studies, Southern University of Science and Technology, 518000 Shenzhen, China

**Keywords:** Sustainability, Synthetic chemistry methodology

## Abstract

Asymmetric hydrogenation of α,β-unsaturated acids catalyzed by noble metals has been well established, whereas, the asymmetric hydrogenation with earth-abundant-metal was rarely reported. Here, we describe a cobalt-catalyzed asymmetric hydrogenation of α,β-unsaturated carboxylic acids. By using chiral cobalt catalyst bearing electron-donating diphosphine ligand, high activity (up to 1860 TON) and excellent enantioselectivity (up to >99% ee) are observed. Furthermore, the cobalt-catalyzed asymmetric hydrogenation is successfully applied to a broad spectrum of α,β-unsaturated carboxylic acids, such as various α-aryl and α-alkyl cinnamic acid derivatives, α-oxy-functionalized α,β-unsaturated acids, α-substituted acrylic acids and heterocyclic α,β-unsaturated acids (30 examples). The synthetic utility of the protocol is highlighted by the synthesis of key intermediates for chiral drugs (6 cases). Preliminary mechanistic studies reveal that the carboxy group may be involved in the control of the reactivity and enantioselectivity through an interaction with the metal centre.

## Abstract (web summary)

A large number of marketed drugs contains a chiral carboxylic acid scaffold. Here, the authors report the asymmetric hydrogenation of α,β-unsaturated carboxylic acids to α-chiral carboxylic acids using a cobalt catalyst bearing an electron-donating chiral diphosphine ligand.

## Introduction

**Fig. 1:**
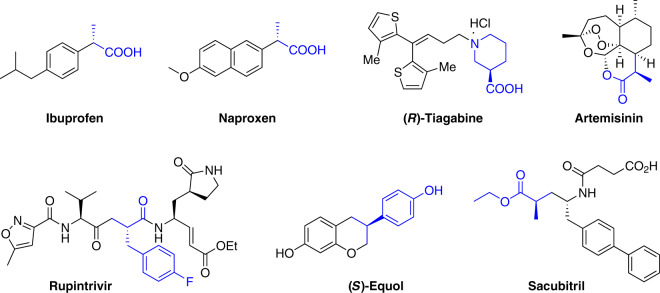
Biologically active compounds and drugs derived from chiral carboxylic acid. Ibuprofen and Naproxen (nonsteroidal anti-inflammatory drugs); (*R*)-Tiagabine (*γ*-aminobutyric acid reuptake inhibitor); Artemisinin (antimalarial drug); Rupintrivir (rhinovirus protease inhibitor); (*S*)-Equol (soy isoflavonoid metabolite); Sacubitril (antihypertensive drug used in combination with Valsartan).

Chiral carboxylic acids are prevalent structural units in varieties of pharmaceutical molecules, agrochemicals, flavors, and fragrances. Some examples are exhibited in Fig. 1. Chiral carboxylic acids such as Ibuprofen, Naproxen^1,2^, and (*R*)-Tiagabine^3^ are well-known drugs. The key intermediates for a large number of drugs or bioactive compounds, such as Artemisinin^4,5^, Rupintrivir^6,7^, (*S*)-Equol^8^, and Sacubitril^9–12^ are chiral carboxylic acids. In addition, chiral carboxylic acids are versatile intermediates for organic synthesis, as they are utilized in the construction of various C–C bonds via decarboxylative coupling reaction^13–16^, and in the activation of C–H bond as a directing group^17,18^. Thus, the development of efficient methods for the preparation of chiral carboxylic acids is highly demanded in both academic research and industrial production.

**Fig. 2:**
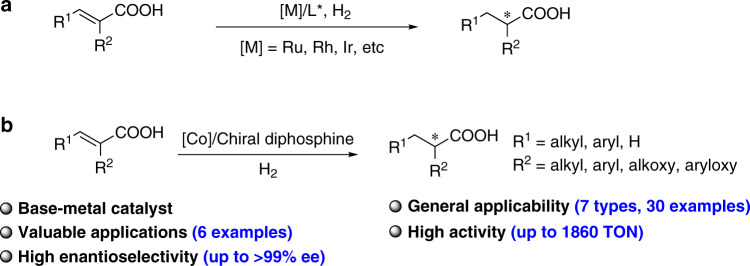
Transition metal-catalyzed asymmetric hydrogenation of α,β-unsaturated carboxylic acids. **a** Previous work: noble metal-based catalysts. **b** This work: cobalt-catalyzed asymmetric hydrogenation of α,β-unsaturated carboxylic acids.

Transition-metal-catalyzed enantioselective hydrogenation of α,β-unsaturated acids is one of the most atom-economic and efficient approaches to access chiral carboxylic acids^19^. Various noble metal-based catalysts, such as Ru catalysts with chiral diphosphine ligands^20–24^, Rh catalysts with chiral phosphorus or nitrogen-containing ligands^25–31^, and Ir catalysts with chiral P,O-^32^ and P,N-ligands^33–40^ have been developed for the hydrogenation of different unsaturated carboxylic acids in high enantioselectivities (Fig. 2a). As modification of ligands or catalysts is necessary to achieve high enantioselectivity for different substrate types, the development of catalytic system with wide applicability is still highly desirable. On the other hand, a major concern regarding the noble metal-based hydrogenation catalysts is the sustainability. Ru, Rh, and Ir are extremely scarce elements, occurring at very low abundances in the earth’s crust (5 × 10^−5^–10^−4^ ppm)^41^. Owing to the low abundance and high cost of the noble metals, the development of cheap earth-abundant metal substitutes to the noble metal catalysts is of high significance^42–54^.

Catalysts based on earth-abundant and environmentally benign cobalt are highly attractive for asymmetric alkene hydrogenation^55–57^. Early studies have showed the potential application of cobalt catalysts in the asymmetric alkene hydrogenation^58–61^ However, these systems suffer from limited enantioselectivities, and in many cases H_2_ could not be used as the stoichiometric reductant. Appreciable progress has been made in recent years, employing cobalt-based complexes for asymmetric hydrogenation of alkenes^62–72^. Chirik and coworkers report the highly enantioselective hydrogenation of styrene derivatives, cyclic alkenes, and enamides with cobalt complexes bearing *C*_1_-symmetric PNN-type pincer ligand or chiral diphosphine ligands^65,71,72^. Stereoselective olefin hydrogenations are also independently described by Lu and Huang group employing chiral IPO–Co and PPO–Co complexes^62,66,67,69^. Though important progresses have been made, the development of base metal catalyst for challenging substrates is still underexplored^56^.

As a continuation of our interest in transition metal-catalyzed asymmetric hydrogenation reactions, herein, we report a highly enantioselective cobalt-catalyzed hydrogenation of α,β-unsaturated carboxylic acids for the synthesis of chiral carboxylic acids. High yields and enantioselectivities are generally achieved for a wide range of substrates (up to 99% yield and up to >99% ee). Besides, the synthetic value of the methodology is demonstrated by its applications in the synthesis of important drugs (Fig. 2b). After the first submission of our paper, the asymmetric hydrogenation of α,β-unsaturated carboxylic acids is reported by Chirik and coworkers^73^ with a cobalt catalytic system, and high yields and enantioselectivities are achieved for a wide range of acrylic acid derivatives.

## Results

### Condition optimization

**Fig. 3:**
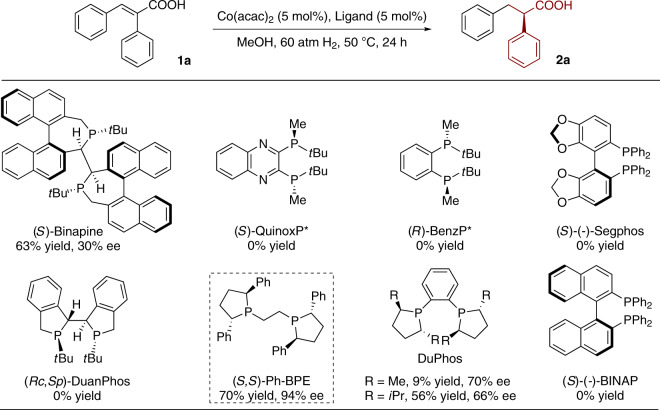
The performance of chiral diphosphine ligands in Co-catalyzed asymmetric hydrogenation of 1a. The yields were determined by ^1^H NMR and the enantioselectivities were determined by HPLC analysis in all cases.

In the initial study, asymmetric hydrogenation of (*E*)-2,3-diphenylacrylic acid **1a** was investigated by using 5 mol% of Co(acac)_2_ and 5 mol% of chiral ligand. The reaction was conducted under 60 atm H_2_ pressure at 50 °C in MeOH over 24 h. Several chiral diphosphine ligands were evaluated and we found that cobalt catalysts ligated by highly electron-rich and sterically demanding diphosphines were catalytically active (Fig. 3). In particular, the Ph-BPE was found to be the best one, which afforded the desired product **2a** in 70% yield and 94% ee. Me-DuPhos and *i*Pr-DuPhos were catalytically active, but only lower yields and moderate ee values were obtained. Other strongly electron-donating bis(alkylphosphine)s like Binaphine and Duanphos were also tested, but they afforded unsatisfactory yields and ee values. *P*-chiral diphosphine QuinoxP* and BenzP* gave no desired product. The less electron-donating bis(arylphosphine)s such as BINAP and Segphos were completely inactive in cobalt-catalyzed hydrogenation (Supplementary Table 2).

Next, various solvents and additives were tested (Table 1). It became apparent that alcoholic solvents are beneficial for the catalytic asymmetric hydrogenation, with *i*PrOH giving the highest activity and enantioselectivity (entries 1–2). The run in more acidic fluorinated solvent, trifluoroethanol (TFE) afforded moderate ee value (entry 3). Furthermore, reactions performed in dimethoxyethane (DME) and toluene led to very low conversions (entries 4–5). When tetrahydrofuran (THF) and 1,4-dioxane were used as solvents, the reactions were totally inhibited (entries 6–7). The investigation of the effect of additives in the reaction showed that the addition of one-electron reductant is benefit for obtaining full conversions with low catalyst loading (entries 8–12). To further improve the enantiocontrol, temperature and hydrogenation pressure effects were surveyed. 97% ee of **2a** could be achieved at room temperature under 40 atm H_2_ pressure with full conversion of **1a** (entry 13). The asymmetric hydrogenation also proceeded smoothly with full conversion and high ee when reducing the catalyst loading to 0.1 mol% (entry 14). Moreover, in the presence of 0.05 mol% of catalyst, **1a** reacted with H_2_ efficiently, giving **2a** in 93% yield on 2 mmol scale without any decrease of the ee value (TON up to 1860, entry 15). The absolute configuration of the product **2a** was established by comparison of its optical rotation with previous report (Supplementary Tables 3–5).

**Table 1:** Optimization for cobalt-catalyzed asymmetric hydrogenation of (*E*)-2,3-diphenylacrylic acid.


Entry	*x* (mol%)	Additive	Solvent	Conv. (%)^a^	ee (%)^b^
1	5	—	MeOH	70	94
2	5	—	*i*PrOH	>98	93
3^c^	5	—	TFE	>98	84
4	5	—	DME	7	43
5	5	—	Toluene	15	86
6	5	—	THF	NR	—
7	5	—	1,4-Dioxane	NR	—
8	1	—	*i*PrOH	61	93
9	1	Cs_2_CO_3_	*i*PrOH	7	88
10	1	KO*t*Bu	*i*PrOH	55	93
11	1	Mn	*i*PrOH	>98	96
12	1	Zn	*i*PrOH	>98	97
13^d^	1	Zn	*i*PrOH	>98	97
14^e^	0.1	Zn	*i*PrOH	>98	97
15^e^	0.05	Zn	*i*PrOH	93	97

*TFE* trifluoroethanol, *DME* dimethoxyethane, *THF* tetrahydrofuran.

Conditions: **1a** (0.1 mmol) in solvent (0.6 mL) under 60 atm H_2_ pressure at 50 °C for 24 h.

^a^Determined by ^1^H NMR.

^b^Determined by HPLC analysis.

^c^Using TFE (0.6 mL) and THF (0.6 mL) as solvent.

^d^Under 40 atm H_2_, room temperature.

^e^2 mmol scale, under 80 atm H_2_, 7 d.

### Substrate scope

**Fig. 4:**
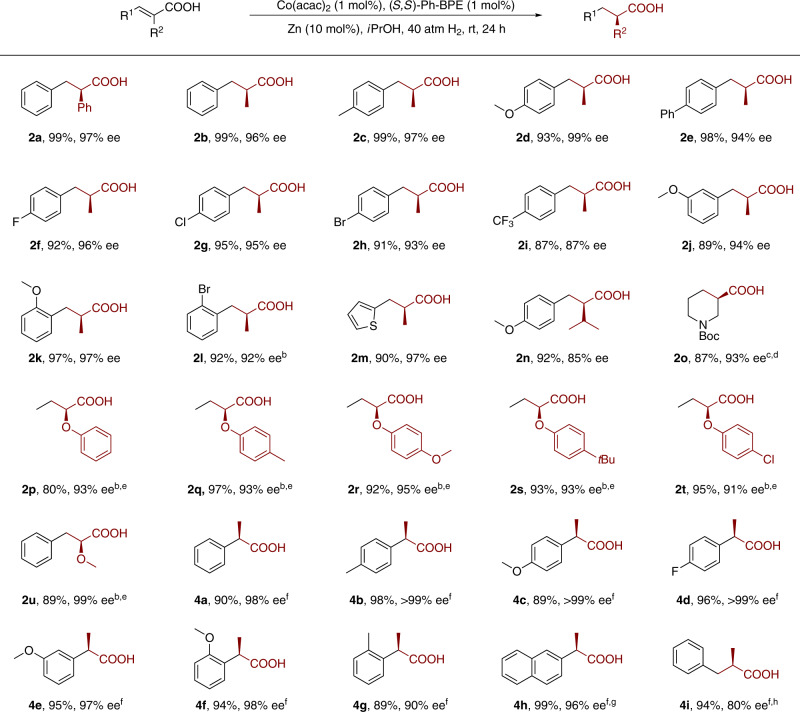
Cobalt-catalyzed asymmetric hydrogenation of various α,β-unsaturated carboxylic acids. ^a^Conditions: **1** (0.1 mmol), Co(acac)_2_ (1 mol%), (*S*,*S*)-Ph-BPE (1 mol%) and Zn (10 mol%) in *i*PrOH (0.6 mL) under 40 atm H_2_ pressure at room temperature for 24 h. Yield of isolated products, unless noted otherwise. The ee values were determined by HPLC analysis. ^b^Under 60 atm H_2_ pressure, 50 °C. ^c^Under 80 atm H_2_ pressure, 50 °C. ^d^With 5 mol% of Cat. ^e^With 5 mol% of CoCl_2_ and (*S*,*S*)-Ph-BPE in MeOH (0.4 mL), 72 h. ^f^**3** (0.1 mmol), CoCl_2_ (5 mol%), (*S*,*S*)-Ph-BPE (5 mol%), and Zn (50 mol%) in HFIP (0.4 mL) under 60 atm H_2_ pressure at 50 °C for 48 h. ^g^In 0.6 mL solvent (MeOH/HFIP = 2/1), 72 h. ^h^In 0.4 mL MeOH.

To delineate the scope of the Co-catalyzed asymmetric hydrogenation, the catalytic system was applied to the reactions of different types of α,β-unsaturated acids. α-Aryl and α-alkyl cinnamic acid derivatives **1** were hydrogenated with 1 mol% catalyst loading under the optimized condition (Fig. 4). Most of the reactions proceeded smoothly under 40 atm H_2_ pressure at ambient temperature, providing the desired products in high isolated yields and excellent enantioselectivities (95–99% ee). The method works efficiently for α-methyl cinnamic acid derivatives bearing both electron-donating (**1c–1e**) and -withdrawing groups (**1f–1i**). Substituents at the *para* and *meta* positions of the phenyl ring are tolerated under the reaction conditions, furnishing the hydrogenation products in high yields with excellent enantioselectivity (**1j–1l**). Heteroaromatic α,β-unsaturated acid **1m** was also efficiently hydrogenated to give the desired product **2m** in 90% isolated yield with 97% ee. Increasing the steric demand in the α*-*substituted position by using an *i*Pr group led to the formation of **2n** with 86% ee.

Our preliminary results show that the [Co]/BPE is also active for hydrogenation of *N*-heterocyclic acids to produce chiral heterocyclic acids, which are present in various pharmaceuticals^74^. The asymmetric hydrogenation of *N*-Boc-1,2,5,6-tetrahydropyridine-3-carboxylic acid **1o** gave the desired product **2o** with an impressive 93% ee. α-Oxy-functionalized α,β-unsaturated acids were also subjected to our [Co]/BPE system due to the corresponding hydrogenation products are important building blocks for asymmetric synthesis in both pharmaceutical and agrochemical industries^75^. To our delight, the catalyst system showed high efficiency in the asymmetric hydrogenation of α-alkoxy- and α-aryloxy-substituted α,β-unsaturated acids. A broad range of α-aryloxy and α-alkoxy substituted α,β-unsaturated acids **1p–1u** were hydrogenated smoothly to give the desired chiral α-oxy-functionalized acids in good to excellent yields and enantioselectivities (80–95% yield, 90–99% ee).

Finally, we turned our attention to the asymmetric hydrogenation of α-substituted acrylic acids (Fig. 4). It is revealed that solvents have critical roles in [Co]/BPE catalyzed hydrogenation of α-substituted acrylic acids, affecting both catalytic activity and selectivity. Full conversion and 98% ee were obtained in hexafluoroisopropanol (HFIP, Supplementary Table 6). A wide range of α-aryl acrylic acids (**3a**–**3h**) were subjected to the cobalt-catalyzed hydrogenation, affording the corresponding chiral carboxylic acids in good to excellent yields and enantioselectivities (89–99% yields, 90–99% ee). Furthermore, 2-alkyl acrylic acid **3i** was also smoothly hydrogenated with 80% ee.

### Synthetic applications

**Fig. 5:**
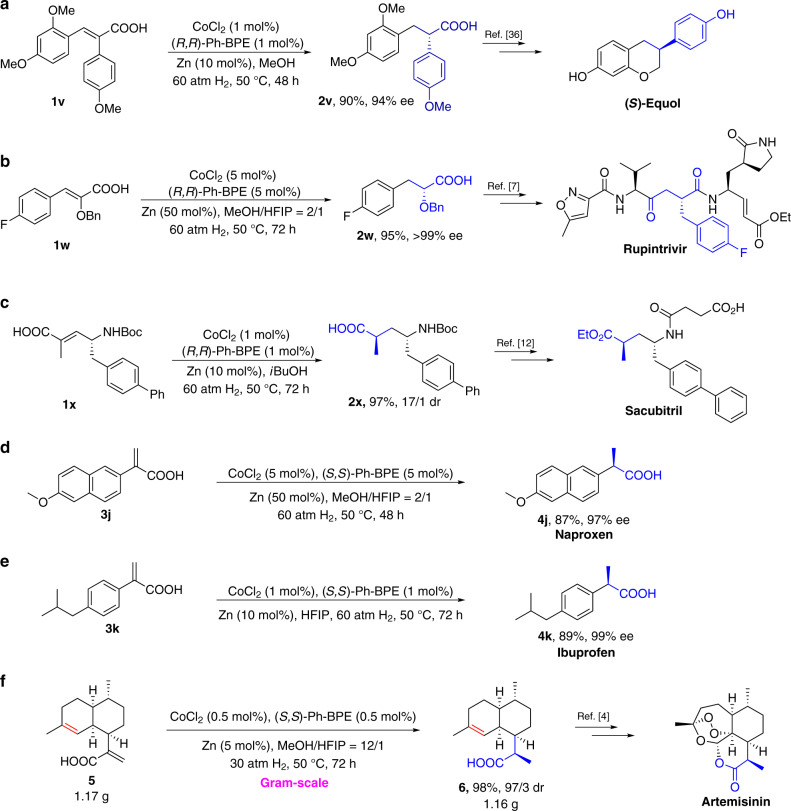
Practical synthetic applications of cobalt-catalyzed asymmetric hydrogenation. **a** Synthesis of (*S*)-Equol intermediate via cobalt-catalyzed hydrogenation. **b** Synthesis of Rupinnavir intermediate. **c** Synthesis of Sacubitril intermediate. **d** Asymmetric synthesis of Naproxen. **e** Asymmetric synthesis of Ibuprofen. **f** Gram-scale asymmetric hydrogenation of artemisinic acid **5**.

To demonstrate the synthetic utility of cobalt-catalyzed enantioselective hydrogenation of α,β-unsaturated acids, its synthetic application in series of chiral natural products and drugs was studied. The critical synthon to (*S*)-Equol, (*S*)-**2v**, could be easily accessed under standard conditions with high enantioselectivity (94% ee, Fig. 5a). Similarly, chiral carboxylic acid **2w**, a key intermediate in the synthesis of rhinovirus protease inhibitor Rupintrivir, was obtained in 95% isolated yield and >99% ee (Fig. 5b). Following optimization of the reaction conditions, Sacubitril intermediate **2x** was obtained in 97% isolated yield and 17/1 dr (Fig. 5c, Supplementary Table 7).

The asymmetric hydrogenation of α-substituted acrylic acids is of highly practical value because optically pure α-substituted propionic acids, such as ibuprofen and naproxen, are well-known non-steroid anti-inflammatory drugs, which can be readily prepared through asymmetric hydrogenation of the corresponding α-aryl acrylic acids. Indeed, Naproxen (**4j**) and Ibuprofen (**4k**) were prepared with high yields and enantioselectivities by using cobalt-based catalytic system (Fig. 5d, e). Based on the excellent performance of Co/BPE in the above reaction, we carried out the gram-scale asymmetric hydrogenation of AA (artemisinic acid) **5** in the presence of 0.5 mol% of chiral cobalt catalyst. Desired product (*R*)-DHAA (dihydroartemisinic acid) **6** was obtained in excellent isolated yield and dr value (1.16 g, 97/3 dr, Fig. 5f). It is noteworthy that the trisubstituted olefin in **5** is unreactive under the Co-catalyzed hydrogenation conditions.

### Mechanism study

**Fig. 6:**
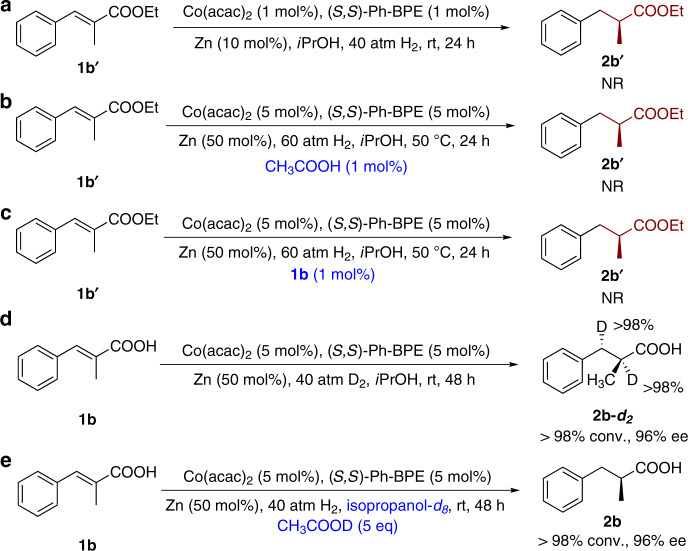
Control experiments and mechanistic investigations. **a** Hydrogenation of ester **1b′** under standard conditions. **b** Hydrogenation of ester **1b′** in the presence of catalytic amount of CH_3_COOH. **c** Reaction of ester **1b′** in the presence of catalytic amount of **1b**. **d** Deuterium-labeling experiment. **e** Hydrogenation of **1b** in isopropanol-*d*_8_ with CH_3_COOD as additive.

To provide insight into the possible catalyst activation mode and the mechanism of the asymmetric hydrogenation of α,β-unsaturated carboxylic acids, several control and catalytic experiments were conducted. Previous research by Chirik and coworkers revealed that additives such as Zn, Mn, or LiCH_2_Si(CH_3_)_3_ were necessary in the cobalt-catalyzed asymmetric hydrogenation of enamines and acted as an activator for pre-catalyst to generate catalytic active cobalt speices^65,71^. However, the reaction of α,β-unsaturated carboxylic acid **1a**, in the absence of any additives, afford **2a** in >98% conversion and 94% ee (Table 1, entry 2). The data suggested that the carboxylic acid might serve as an activator and mediate the activation process. In sharp contrast to the high reactivity and enantioselectivity observed in the case of **1b** (99% yield and 96% ee, Fig. 4), no reaction occurred for the corresponding ethyl ester **1b′** under standard conditions (Fig. 6a). Moreover, no hydrogenation product was observed when AcOH or **1b** was added to the reaction mixture as external carboxylic acid (Fig. 6b, c). These results indicated that the carboxy group of substrate may be involved in the control of the reactivity and enantioselectivity through interaction with the metal center^19,22–24,33^.

The deuterium-labeling experiments were also conducted to elucidate the reaction mechanism. The reaction of **1b** with D_2_ under standard condition produced **2b-*d***_**2**_ smoothly in >98% conversion and 96% ee (Fig. 6d). Performing the hydrogenation reaction under 40 atm of H_2_ in isopropanol-*d*_8_ solution with 5 eq. CH_3_COOD gave **2b** in >98% conversion, and no deuterated products were observed (Fig. 6e). These data suggested that the H_2_ served as the hydrogen source and protonation of the Co-alkyl intermediate was probably not involved in the current reaction (Supplementary Figs. 1–5). Besides, EPR experiments were also conducted to monitor the process of the current reaction using **1b** as model substrate. The EPR spectra changed greatly after the addition of (*S*,*S*)-Ph-BPE and substrate **1b**, suggesting that the coordination of the ligand and the exchange between acac and substrate **1b** probably happened (Supplementary Figs. 6–11).

**Fig. 7:**
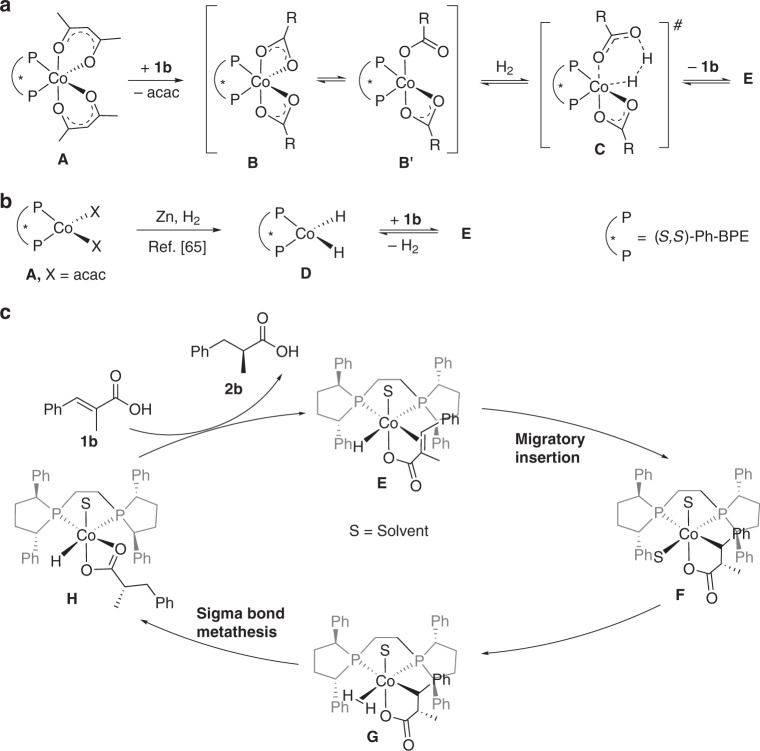
Proposed catalytic cycle. **a** Generation of catalytic species **E** in the absence of Zn. **b** Generation of catalytic species **E** in the presence of Zn. **c** Proposed catalytic cycle.

Based on our experimental observations and the previous study on iridium-catalyzed enantioselective hydrogenation of α,β-unsaturated acids^33^, we proposed a plausible mechanism (Fig. 7). The key catalytic intermediate **E** could be generated through two pathways. In the absence of Zn, complex **E** can be generated through carboxy group mediated H_2_ heterolytic process. Coordination of Co(acac)_2_ with (*S*,*S*)-Ph-BPE generates Co(II) complex **A**, which then undergoes ligand exchange with more acidic substrate **1b** to produce complex **B**, complex **B** and **B′** are in equilibrium with each other. Heterolysis of H_2_ by complex **B′** produces the key catalytic species **E** via transition state **C**. Intermediate **E** may be also produced through protonation^76^ of dihydride complex **D**^65,73^ with **1b** when employing one-electron reductant. Chirik and coworkers^73^ reported an alternative mechanism which involved the migratory insertion of the dihydride complex and the subsequent reduction elimination as key step. Note that the addition of one-electron reductant is beneficial for obtaining full conversions with low catalyst loading (vide supra). The success of Zn or Mn used in this reaction is presumably due to enhanced activation of Co(II) precursor and suppressed deactivation of the active catalyst. The key intermediate **E** then enters the catalytic cycle. Intramolecular migratory insertion of complex **E** produces five-membered intermediate **F**. Coordination of H_2_ to **F** forms complex **G**, which undergoes subsequent sigma-bond metathesis to give complex **H**. The ligand exchange of intermediate **H** with unsaturated carboxylate substrate releases the hydrogenation product **2b** and regenerates the cobalt hydride complex **D**. Mechanistic investigations indicate that the carboxy group has a pivotal role in the improvement of the reactivity and enantioselectivity via coordination with the metal center, and that is why the current reaction does not work for α,β-unsaturated esters.

## Discussion

In summary, we have developed a highly efficient cobalt-catalyzed asymmetric hydrogenation of α,β-unsaturated carboxylic acids. The cobalt catalyst system exhibited both high activities (up to 1860 TON) and excellent enantioselectivities (up to >99% ee) for a broad spectrum of α,β-unsaturated acids. It also affords an efficient way to the key intermediates of many chiral natural products and drugs with high enantioselectivities. Moreover, this operationally simple and atom-economic protocol could be easily scaled-up in gram-scale using 0.5 mol% catalyst loading for the asymmetric synthesis of key intermediate of Artemisinin. Mechanistic studies suggest that the carboxy group may be involved in the control of the reactivity and enantioselectivity through interaction with the metal center. Additional mechanistic and DFT studies of the asymmetric transformations with such cobalt system are currently underway in our laboratory.

## Methods

### General procedure for asymmetric hydrogenatio**n**

In an argon-filled glovebox, Co(acac)_2_ (0.010 M in *i*PrOH, 0.10 mL, 0.001 mmol) and (*S*,*S*)-Ph-BPE (0.010 M in THF, 0.10 mL, 0.001 mmol) were stirred in a vial at room temperature for 10 min. Then zinc dust (0.65 mg, 0.01 mmol) and *i*PrOH (0.50 mL) were added and the mixture was stirred for 15 min. After that, substrate (0.1 mmol) was added to the reaction mixture. The vial was subsequently transferred into an autoclave and purged by three cycles of pressurization/venting with H_2_. The reaction was then stirred under H_2_ (40 atm) at room temperature for 24 h. The hydrogen gas was released slowly and carefully. The resulting solution was concentrated in vacuum and the residue was purified by chromatography on silica gel. The ee values were determined by HPLC with a chiral column.

## Supplementary information

Supplementary Information

## Data Availability

The authors declare that the data supporting the findings of this study are available within the paper and its supplementary information files.
