# Supplementary material for: Cobalt-catalyzed highly enantioselective hydrogenation of α,β-unsaturated carboxylic acids
Source: Nat Commun. 2020 Jun 26;11:3239. doi: 10.1038/s41467-020-17057-z (PMC7319995; doi:10.1038/s41467-020-17057-z)
Supplement: Supplementary file 1 — Supplementary Information [file 41467_2020_17057_MOESM1_ESM.pdf]

Supplementary Information

Cobalt-Catalyzed Highly Enantioselective Hydrogenation of *α,β*-Unsaturated Carboxylic Acids

Du *et al*

Supplementary Methods

General information

All the reactions dealing with air- or moisture- sensitive compounds were carried out in a dry reaction vessel under an argon atmosphere or in an argon-filled glove box. Unless otherwise noted, all reagents and solvents were purchased from commercial suppliers without further purification. Anhydrous solvents were purchased from J&K Chemical and degassed by bubbling argon over a period of 30 min. Purification of products was carried out by flash chromatography using silica gel (200-300 mesh). Thin layer chromatography (TLC) was performed on EM reagents 0.25 mm silica 60-F plates. $Co(acac)_2$ and other metal precursors were purchased from Strem or Alfa.

^{1}H, ^{13}C, ^{19}F and ^{31}P NMR spectra were recorded on a Bruker Avance 400 MHz or a Bruker Avance 600 MHz spectrometer with tetramethylsilane as the internal standard. Chemical shifts are reported in parts per million (ppm, δ scale) downfield from TMS at 0.00 ppm and referenced to the $CDCl_3$ at 7.26 ppm for ^{1}H NMR or 77.0 ppm for ^{13}C NMR. Data are reported as: multiplicity (s = singlet, d = doublet, t = triplet, q = quartet, m = multiplet), coupling constant in hertz (Hz) and signal area integration in natural numbers. ^{13}C NMR and ^{31}P NMR analyses were recorded with ^{1}H decoupling. Enantiomeric excess values were determined with Agilent 1290 or 1260 Series HPLC instrument on a chiral stationary phase. Optical rotations were measured using a 1 mL cell with a 1 dm path length on a Rudolph Autopol I polarimeter at 589 nm.

Asymmetric hydrogenation of 2,3-disubstituted acrylic acids

Method A (for 1a-o).

In an argon-filled glovebox, $Co(acac)_2$ (0.010 M in *i*PrOH, 0.10 mL, 0.001 mmol) and (*S,S*)-Ph-BPE (0.010 M in THF, 0.10 mL, 0.001 mmol) were stirred in a vial at room temperature for 10 min. Then zinc dust (0.65 mg, 0.01 mmol) and *i*PrOH (0.50 mL) were added and the mixture was stirred for 15 min. After that, substrate (0.1 mmol) was added to the reaction mixture. The vial was subsequently transferred into an autoclave and purged by three cycles of pressurization/venting with H_2. The reaction was then stirred under H_2 (40 atm) at room temperature for 24 h. The hydrogen gas was released slowly and carefully. The resulting solution was concentrated in vacuum and the residue was purified by chromatography on silica gel. The ee values were determined by HPLC with a chiral column.

Method B (for 1p-u).

In an argon-filled glovebox, $CoCl_2$ (0.025 M in THF, 0.2 mL, 0.005 mmol) and (*S,S*)-Ph-BPE (2.53 mg, 0.005 mmol) in MeOH (0.2 mL) were stirred in a vial at room temperature for 10 min. Then zinc dust (0.65 mg, 0.01 mmol) and MeOH (0.2 mL) were added and the mixture was stirred for 15 min. After that, substrate (0.1 mmol) was added to the reaction mixture. The vial was subsequently transferred into an autoclave and purged by three cycles of pressurization/venting with H_2. The reaction was then stirred under H_2 (60 atm) at 50 °C for 72 h. The hydrogen gas was released slowly and carefully. The resulting solution was concentrated in vacuum and the residue was purified by chromatography on silica gel. The ee values were determined by HPLC with a chiral column.

Supplementary Table 1. Cobalt precursors screening[a]

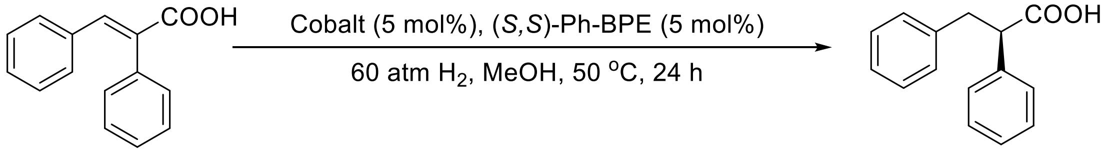

1a **2a**

entry	cobalt	conv. [%][b]	*ee* [%][c]
1	$Co(BF_4)_2{\cdot}6H_2O$	ND	--
2	Co(II) stearate	23	--
3	Co(II) oxalate	ND	--
4	$CoCl_2$	ND	--
5	$Co(acac)_2$	70	94
6	$Co(OAc)_2{\cdot}4H_2O$	57	87

[a] Reaction conditions: **1** (0.1 mmol), [Co] (5 mol%), (*S,S*)-Ph-BPE (5 mol%) in MeOH (0.4 mL) under 60 atm H_2 pressure at 50 °C for 24 h. [b] Determined by ^1H NMR. [c] Determined by HPLC analysis.

Supplementary Table 2. Ligand screening[a]

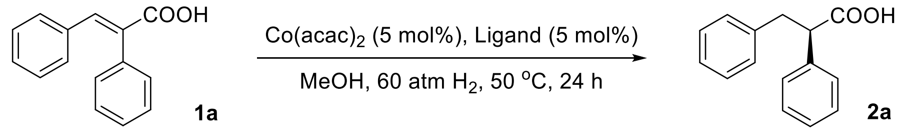

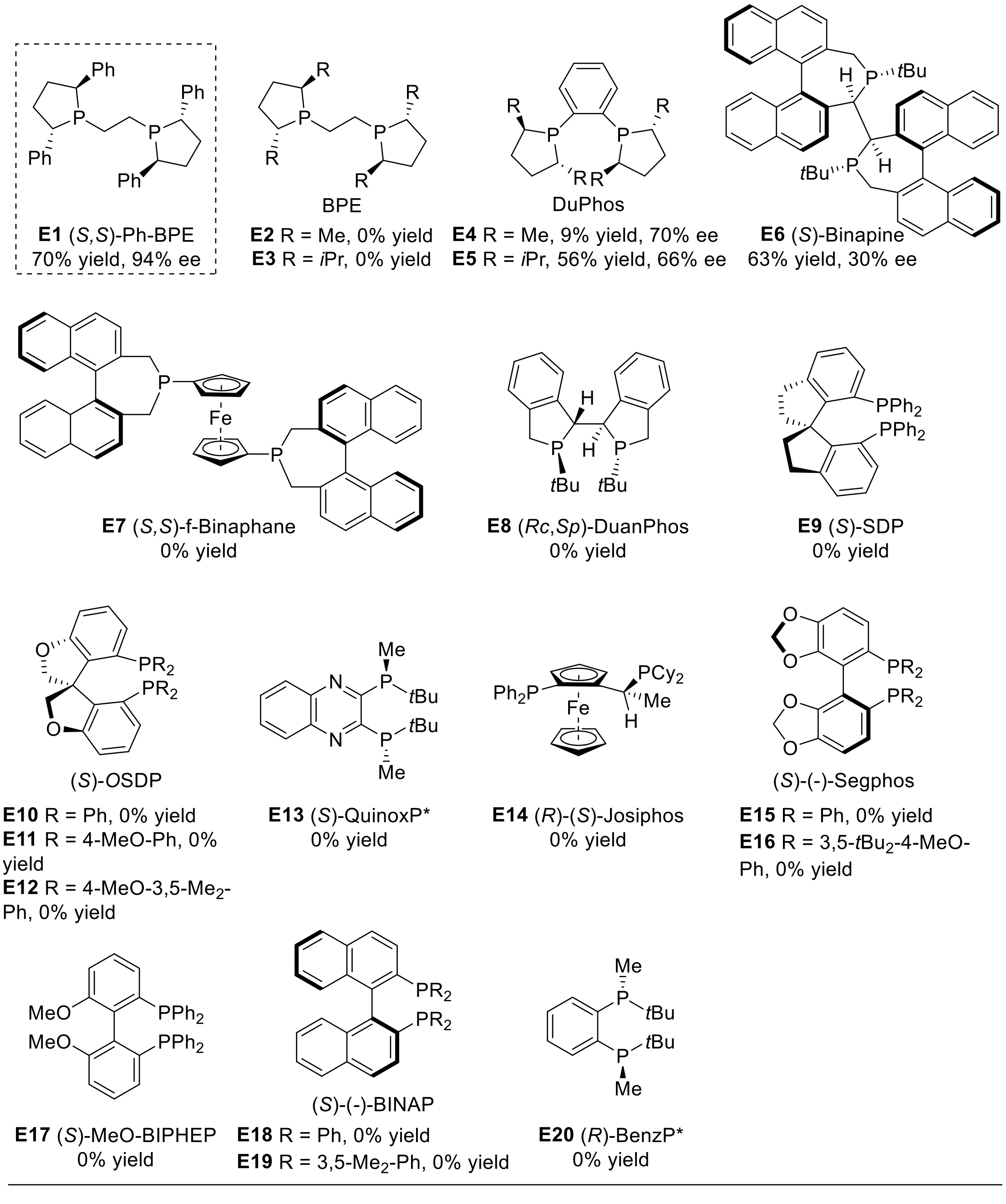

[a] Reaction conditions: **1** (0.1 mmol), $Co(acac)_2$ (5 mol%), Ligand (5 mol%) in MeOH (0.4 mL) under 60 atm H_2 pressure at 50 °C for 24 h. Conversions were determined by ^{1}H NMR and ee values were determined by HPLC analysis.

Supplementary Table 3. Solvent screening[a]

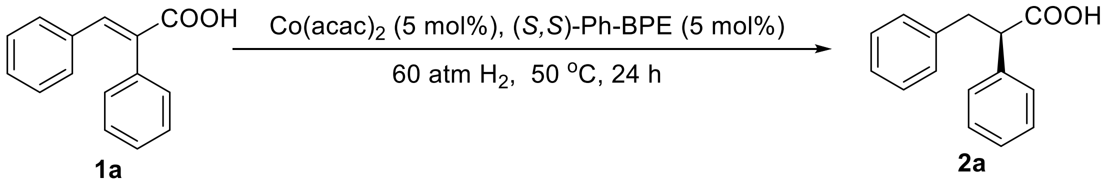

entry	solvent	conv. [%][b]	*ee* [%][c]
1	MeOH	70	94
2	*t*BuOH	88	87
3	*i*PrOH	>98	93
4	TFE	>98	84
5	DME	7	43
6	toluene	15	86
7	CH_3CN	58	64
8	THF	ND	--
9	1,4-Dioxane	ND	--
10	Et_2O	ND	--

[a] Reaction conditions: **1** (0.1 mmol), $Co(acac)_2$ (5 mol%), (*S,S*)-Ph-BPE (5 mol%) in solvent (0.6 mL) under 60 atm H_2 pressure at 50 °C for 24 h. [b] Determined by ^1H NMR. [c] Determined by HPLC analysis.

Supplementary Table 4. Additive screening[a]

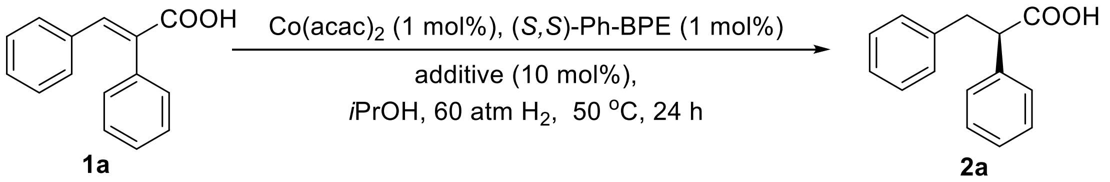

entry	aditive	conv. [%][b]	*ee* [%][c]
1	Zn	>98	97
2	Mn	>98	96
3	LiO*t*Bu	36	95
4	NaO*t*Bu	48	95
5	KO*t*Bu	55	93
6	Cs_2CO_3	7	88
7	NaOH	57	93
8	none	61	93

[a] Reaction conditions: **1** (0.1 mmol), $Co(acac)_2$ (1 mol%), (*S,S*)-Ph-BPE (1 mol%), additive (10 mol%) in *i*PrOH (0.6 mL) under 60 atm H_2 pressure at 50 °C for 24 h. [b] Determined by ^1H NMR. [c] Determined by HPLC analysis.

Supplementary Table 5. Pressure and temperature screening[a]

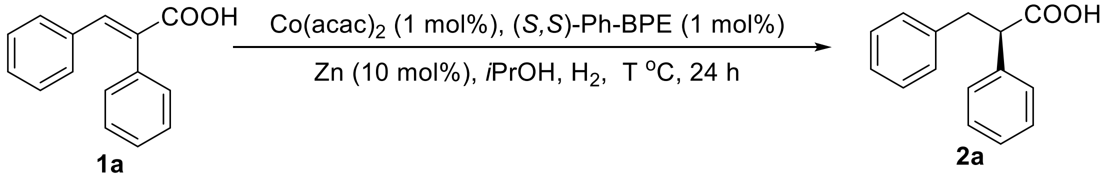

entry	H_2 [atm]	T [°C]	conv. [%][b]	*ee* [%][c]
1	60	80	>98	95.6
2	60	30	>98	96.9
3	60	rt	>98	97.4
4	18	rt	97	97.3
5	25	rt	>98	97.2
6	30	rt	>98	96.9
7	40	rt	>98	97.3

[a] Reaction conditions: **1** (0.1 mmol), Co(acac)$_2$ (1 mol%), (*S,S*)-Ph-BPE (1 mol%), Zn (10 mol%) in *i*PrOH (0.6 mL) for 24 h. [b] Determined by ^{1}H NMR. [c] Determined by HPLC analysis.

Characterization data of compound 2

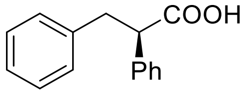

(R)-2,3-diphenylpropanoic acid (2a).[1] White solid, 99% yield, 97% ee, $[\alpha]_D^{25}$ = -77.62 (c = 0.84, $CHCl_3$). ^{1}H NMR (400 MHz, Chloroform-*d*) δ 7.29 – 7.25 (m, 4H), 7.24 – 7.10 (m, 4H), 7.09 – 7.04 (m, 2H), 3.82 (t, *J* = 7.7 Hz, 1H), 3.37 (dd, *J* = 13.8, 8.4 Hz, 1H), 2.99 (dd, *J* = 13.8, 7.0 Hz, 1H). ^{13}C NMR (101 MHz, Chloroform-*d*) δ 179.2, 138.7, 137.9, 128.9, 128.7, 128.3, 128.1, 127.6, 126.4, 53.5, 39.2. The enantiomeric excess of **2a** was determined by HPLC analysis on Chiralpak OJ-3 column. Conditions: hexane/isopropanol = 97/3, flow rate = 0.8 mL/min, uv-vis detection at λ = 210 nm, t_R = 19.9 min (major), 29.6 min (minor).

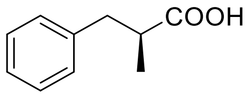

(S)-2-methyl-3-phenylpropanoic acid (2b).[2] Colorless oil, 99% yield, 96% ee, $[\alpha]_D^{25}$ = +12.13 (c = 0.78, $CHCl_3$). ^{1}H NMR (400 MHz, Chloroform-d) δ 7.30 (t, J = 7.3 Hz,

2H), 7.25 – 7.16 (m, 3H), 3.09 (dd, J = 13.4, 6.3 Hz, 1H), 2.88 – 2.75 (m, 1H), 2.68 (dd, J = 13.4, 8.0 Hz, 1H), 1.19 (d, J = 6.9 Hz, 3H). ^{13}C NMR (101 MHz, Chloroform-d) δ 182.3, 139.0, 129.0, 128.4, 126.4, 41.3, 39.3, 16.5. The enantiomeric excess of **2b** was determined by HPLC analysis on Chiralpak OJ-3 column. Conditions: hexane/isopropanol = 97/3, flow rate = 0.8 mL/min, uv-vis detection at λ = 210 nm, t_R = 9.5 min (minor), 10.5 min (major).

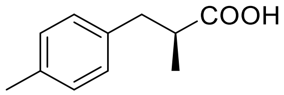

(S)-2-methyl-3-(p-tolyl)propanoic acid (2c).[3] Colorless oil, 99% yield, 97% ee, $[\alpha]_D^{25}$ = +49.23 (c = 0.39, $CHCl_3$). ^{1}H NMR (400 MHz, Chloroform-*d*) δ 7.13 – 7.06 (m, 4H), 3.04 (dd, *J* = 13.4, 6.3 Hz, 1H), 2.74 (dt, *J* = 13.7, 6.9 Hz, 1H), 2.64 (dd, *J* = 13.4, 8.0 Hz, 1H), 2.33 (s, 3H), 1.18 (d, *J* = 6.9 Hz, 3H). ^{13}C NMR (101 MHz, Chloroform-*d*) δ 182.4, 135.9, 129.1, 128.9, 41.3, 38.9, 21.0, 16.4. The enantiomeric excess of **2c** was determined by HPLC analysis on Chiralpak OJ-3 column. Conditions: hexane/isopropanol = 97/3, flow rate = 0.8 mL/min, uv-vis detection at λ = 220 nm, t_R = 12.8 min (minor), 13.9 min (major).

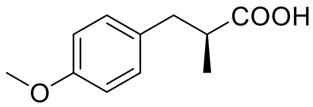

(S)-3-(4-methoxyphenyl)-2-methylpropanoic acid (2d).[4] Colorless oil, 93% yield, 99% ee, $[\alpha]_D^{25}$ = +20.44 (c = 0.91, $CHCl_3$). ^{1}H NMR (400 MHz, Chloroform-*d*) δ 7.10 (d, *J* = 8.6 Hz, 2H), 6.83 (d, *J* = 8.6 Hz, 2H), 3.79 (s, 3H), 3.01 (dd, *J* = 13.4, 6.4 Hz, 1H), 2.72 (h, *J* = 6.8 Hz, 1H), 2.62 (dd, *J* = 13.4, 7.9 Hz, 1H), 1.17 (d, *J* = 6.9 Hz, 3H). ^{13}C NMR (101 MHz, Chloroform-*d*) δ 182.3, 158.1, 131.1, 129.9, 113.8, 55.2, 41.4, 38.4, 16.4. The enantiomeric excess of **2d** was determined by HPLC analysis on Chiralpak OJ-3 column. Conditions: hexane/isopropanol = 97/3, flow rate = 0.8 mL/min, uv-vis detection at λ = 220 nm, t_R = 29.0 min (minor), 30.7 min (major).

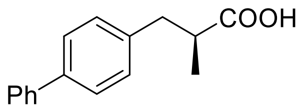

(S)-3-([1,1'-biphenyl]-4-yl)-2-methylpropanoic acid (2e). White solid, 98% yield, 94% ee, $[\alpha]_D^{25}$ = +7.09 (c = 1.03, $CHCl_3$). ^{1}H NMR (400 MHz, Chloroform-*d*) δ 7.60 – 7.55 (m, 2H), 7.52 (d, *J* = 8.1 Hz, 2H), 7.42 (t, *J* = 7.6 Hz, 2H), 7.33 (t, *J* = 7.3 Hz, 1H), 7.26 (d, *J* = 8.2 Hz, 2H), 3.12 (dd, *J* = 13.4, 6.3 Hz, 1H), 2.81 (h, *J* = 6.8 Hz, 1H), 2.72 (dd, *J* = 13.4, 8.0 Hz, 1H), 1.22 (d, *J* = 6.9 Hz, 3H). ^{13}C NMR (151 MHz, Chloroform-*d*) δ 182.2, 140.9, 139.3, 138.1, 129.4, 128.7, 127.1, 127.0, 41.2, 38.9, 16.5. HRMS calculated $[M-H]^-$ for $C_{16}H_{15}O_2^-$ = 239.1078, found: 239.1073. The enantiomeric excess of **2e** was determined by HPLC analysis on Chiralpak OJ-3 column. Conditions: hexane/isopropanol = 97/3, flow rate = 0.8 mL/min, uv-vis detection at λ = 210 nm, t_R = 30.5 min (minor), 31.8 min (major).

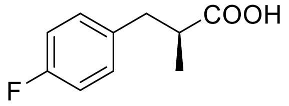

(S)-3-(4-fluorophenyl)-2-methylpropanoic acid (2f).[5] Colorless oil, 92% yield, 96% ee, $[\alpha]_D^{25}$ = +13.90 (c = 0.59, $CHCl_3$). ^{1}H NMR (400 MHz, Chloroform-*d*) δ 7.14 (dd, *J* = 8.4, 5.5 Hz, 2H), 6.97 (t, *J* = 8.7 Hz, 2H), 3.02 (dd, *J* = 13.3, 6.4 Hz, 1H), 2.79 – 2.70 (m, 1H), 2.66 (dd, *J* = 13.3, 7.6 Hz, 1H), 1.18 (d, *J* = 6.7 Hz, 3H). ^{13}C NMR (101 MHz, Chloroform-*d*) δ 182.1, 161.6 (d, *J* = 244.6 Hz), 134.6 (d, *J* = 3.5 Hz), 130.4 (d, *J* = 7.9 Hz), 115.2 (d, *J* = 21.1 Hz), 41.4, 38.5, 16.5. ^{19}F NMR (376 MHz, Chloroform-*d*) δ -116.7. The enantiomeric excess of **2f** was determined by HPLC analysis on Chiralpak OJ-3 column. Conditions: hexane/isopropanol = 97/3, flow rate = 0.8 mL/min, uv-vis detection at λ = 210 nm, t_R = 10.7 min (minor), 12.1 min (major).

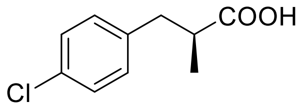

(S)-3-(4-chlorophenyl)-2-methylpropanoic acid (2g).[2] Colorless oil, 95% yield, 95% ee, $[\alpha]_D^{25}$ = +14.20 (c = 0.69, $CHCl_3$). ^{1}H NMR (600 MHz, Chloroform-*d*) δ 7.25 (d, *J* = 7.3 Hz, 2H), 7.12 (d, *J* = 8.1 Hz, 2H), 3.02 (dd, *J* = 13.6, 6.8 Hz, 1H), 2.73 (p, *J* =

7.1 Hz, 1H), 2.66 (dd, J = 13.6, 7.6 Hz, 1H), 1.18 (d, J = 6.9 Hz, 3H). ^{13}C NMR (151 MHz, Chloroform-*d*) δ 181.9, 137.4, 132.3, 130.3, 128.6, 41.3, 38.6, 16.6. The enantiomeric excess of **2g** was determined by HPLC analysis on Chiralpak OJ-3 column. Conditions: hexane/isopropanol = 97/3, flow rate = 0.8 mL/min, uv-vis detection at λ = 220 nm, t_R = 10.1 min (minor), 10.5 min (major).

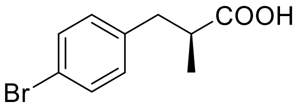

(S)-3-(4-bromophenyl)-2-methylpropanoic acid (2h).[2] Colorless oil, 91% yield, 93% ee, $[\alpha]_D^{25}$ = +16.88 (c = 0.96, $CHCl_3$). ^1H NMR (400 MHz, Chloroform-*d*) δ 7.41 (d, J = 8.3 Hz, 2H), 7.06 (d, J = 8.3 Hz, 2H), 3.01 (dd, J = 13.4, 6.7 Hz, 1H), 2.74 (h, J = 6.9 Hz, 1H), 2.64 (dd, J = 13.5, 7.6 Hz, 1H), 1.18 (d, J = 6.9 Hz, 3H). ^{13}C NMR (101 MHz, Chloroform-*d*) δ 182.0, 137.9, 131.5, 130.7, 120.3, 41.1, 38.6, 16.5. The enantiomeric excess of **2h** was determined by HPLC analysis on Chiralpak OJ-3 column. Conditions: hexane/isopropanol = 98/2, flow rate = 0.5 mL/min, uv-vis detection at λ = 230 nm, t_R = 19.4 min (minor), 20.2 min (major).

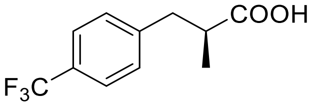

(S)-2-methyl-3-(4-(trifluoromethyl)phenyl)propanoic acid (2i).[3] Colorless oil, 87% yield, 87% ee, $[\alpha]_D^{25}$ = +7.16 (c = 0.95, $CHCl_3$). ^1H NMR (400 MHz, Chloroform-*d*) δ 7.55 (d, J = 8.0 Hz, 2H), 7.30 (d, J = 8.0 Hz, 2H), 3.12 (dd, J = 12.6, 5.9 Hz, 1H), 2.85 – 2.70 (m, 2H), 1.21 (d, J = 6.7 Hz, 3H). ^{13}C NMR (151 MHz, Chloroform-*d*) δ 181.7, 143.1, 129.3, 128.9 (q, J = 32.3 Hz), 125.4 (q, J = 3.9 Hz), 124.2 (q, J = 271.8 Hz), 40.9, 38.9, 16.6. ^{19}F NMR (376 MHz, Chloroform-*d*) δ -62.4. The enantiomeric excess of **2c** was determined by HPLC analysis on Chiralpak OJ-3 column. Conditions: hexane/isopropanol = 98/2, flow rate = 0.5 mL/min, uv-vis detection at λ = 220 nm, t_R = 13.0 min (minor), 13.6 min (major).

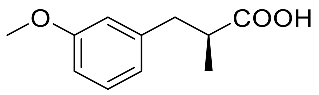

(S)-3-(3-methoxyphenyl)-2-methylpropanoic acid (2j).[3] Colorless oil, 89% yield, 94% ee, $[\alpha]_D^{25}$ = +5.53 (c = 0.85, $CHCl_3$). ^{1}H NMR (400 MHz, Chloroform-*d*) δ 7.21 (t, *J* = 7.8 Hz, 1H), 6.82 – 6.72 (m, 3H), 3.79 (s, 3H), 3.06 (dd, *J* = 13.5, 6.4 Hz, 1H), 2.77 (h, *J* = 6.8 Hz, 1H), 2.65 (dd, *J* = 13.5, 8.1 Hz, 1H), 1.19 (d, *J* = 6.9 Hz, 3H). ^{13}C NMR (101 MHz, Chloroform-*d*) δ 182.2, 159.6, 140.6, 129.4, 121.4, 114.7, 111.8, 55.1, 41.1, 39.3, 16.5. The enantiomeric excess of **2j** was determined by HPLC analysis on Chiralpak OJ-3 column. Conditions: hexane/isopropanol = 97/3, flow rate = 0.8 mL/min, uv-vis detection at λ = 220 nm, t_R = 15.1 min (minor), 18.5 min (major).

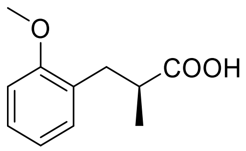

(S)-3-(2-methoxyphenyl)-2-methylpropanoic acid (2k).[3] Colorless oil, 97% yield, 97% ee, $[\alpha]_D^{25}$ = +27.62 (c = 0.21, $CHCl_3$). ^{1}H NMR (400 MHz, Chloroform-*d*) δ 7.24 – 7.18 (m, 1H), 7.16 – 7.10 (m, 1H), 6.91 – 6.81 (m, 2H), 3.81 (s, 3H), 3.05 (dd, *J* = 13.2, 6.7 Hz, 1H), 2.86 (h, *J* = 6.6 Hz, 1H), 2.71 (dd, *J* = 13.2, 7.6 Hz, 1H), 1.16 (d, *J* = 7.0 Hz, 3H). ^{13}C NMR (151 MHz, Chloroform-*d*) δ 182.8, 157.6, 130.9, 127.7, 127.4, 120.2, 110.2, 55.1, 39.3, 34.2, 16.7. The enantiomeric excess of **2k** was determined by HPLC analysis on Chiralpak OJ-3 column. Conditions: hexane/isopropanol = 97/3, flow rate = 0.8 mL/min, uv-vis detection at λ = 220 nm, t_R = 10.0 min (minor), 11.1 min (major).

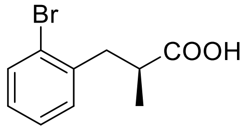

(S)-3-(2-bromophenyl)-2-methylpropanoic acid (2l).[3] Colorless oil, 92% yield, 92% ee, $[\alpha]_D^{25}$ = +14.94 (c = 0.79, $CHCl_3$). ^{1}H NMR (400 MHz, Chloroform-*d*) δ 7.55 (d, *J* = 7.9 Hz, 1H), 7.23 (d, *J* = 4.5 Hz, 2H), 7.09 (dt, *J* = 8.0, 4.5 Hz, 1H), 3.19 (dd, *J* = 13.5, 6.9 Hz, 1H), 2.93 (h, *J* = 6.8 Hz, 1H), 2.82 (dd, *J* = 13.5, 7.6 Hz, 1H), 1.23 (d, *J*

= 7.0 Hz, 3H). ^{13}C NMR (151 MHz, Chloroform-*d*) δ 182.0, 138.4, 132.9, 131.3, 128.2, 127.3, 124.7, 39.4, 39.3, 16.7. The enantiomeric excess of **2l** was determined by HPLC analysis on Chiralpak OJ-3 column. Conditions: hexane/isopropanol = 97/3, flow rate = 0.8 mL/min, uv-vis detection at λ = 220 nm, t_R = 8.7 min (minor), 9.2 min (major).

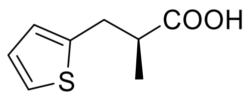

(S)-2-methyl-3-(thiophen-2-yl)propanoic acid (2m).[6] Colorless oil, 90% yield, 97% ee, $[\alpha]_D^{25}$ = +5.19 (c = 0.77, $CHCl_3$). ^{1}H NMR (400 MHz, Chloroform-*d*) δ 7.15 (dd, *J* = 5.1, 1.2 Hz, 1H), 6.93 (dd, *J* = 5.1, 3.4 Hz, 1H), 6.86 – 6.81 (m, 1H), 3.26 (dd, *J* = 14.7, 6.6 Hz, 1H), 2.94 (dd, *J* = 14.7, 7.4 Hz, 1H), 2.80 (h, *J* = 7.0 Hz, 1H), 1.24 (d, *J* = 7.0 Hz, 3H). ^{13}C NMR (151 MHz, Chloroform-*d*) δ 181.9, 141.3, 126.8, 125.7, 123.9, 41.6, 33.2, 16.6. The enantiomeric excess of **2m** was determined by HPLC analysis on Chiralpak OJ-3 column. Conditions: hexane/isopropanol = 97/3, flow rate = 0.8 mL/min, uv-vis detection at λ = 230 nm, t_R = 11.5 min (minor), 14.0 min (major).

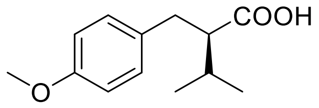

(R)-2-(4-methoxybenzyl)-3-methylbutanoic acid (2n).[7] White solid, 92% yield, 85% ee, $[\alpha]_D^{25}$ = +36.60 (c = 1.06, $CHCl_3$). ^{1}H NMR (400 MHz, Chloroform-*d*) δ 7.09 (d, *J* = 8.6 Hz, 2H), 6.80 (d, *J* = 8.6 Hz, 2H), 3.77 (s, 3H), 2.83 – 2.77 (m, 2H), 2.44 (dt, *J* = 9.1, 6.3 Hz, 1H), 1.95 (h, *J* = 6.8 Hz, 1H), 1.03 (dd, *J* = 11.5, 6.8 Hz, 6H). ^{13}C NMR (101 MHz, Chloroform-*d*) δ 181.0, 158.0, 131.6, 129.7, 113.8, 55.2, 54.6, 34.5, 30.3, 20.3, 20.0 . The enantiomeric excess of **2n** was determined by HPLC analysis on Chiralpak OJ-3 column. Conditions: hexane/isopropanol = 97/3, flow rate = 0.8 mL/min, uv-vis detection at λ = 210 nm, t_R = 16.2 min (major), 18.6 min (minor).

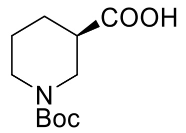

(R)-1-(tert-butoxycarbonyl)piperidine-3-carboxylic acid (2o).[8] White solid, 87% yield, 93% ee, $[\alpha]_D^{25}$ = -5.22 (c = 0.69, $CHCl_3$). ^1H NMR (400 MHz, Chloroform-*d*) δ 4.12 (brs, *J* = 7.1 Hz, 1H), 3.88 (dt, *J* = 13.3, 4.1 Hz, 1H), 3.03 (brs, 1H), 2.85 (ddd, *J* = 13.7, 10.9, 3.0 Hz, 1H), 2.48 (tt, *J* = 10.1, 3.9 Hz, 1H), 2.12 – 2.01 (m, 1H), 1.77 – 1.58 (m, 2H), 1.45 (s, 9H). ^{13}C NMR (151 MHz, Chloroform-*d*) δ 178.8, 154.7, 79.9, 45.6, 43.8, 41.1, 28.4, 27.2, 24.1. The enantiomeric excess of **2o** was determined by HPLC analysis on Chiralpak AD-3 column. Conditions: hexane/isopropanol = 97/3, flow rate = 0.8 mL/min, uv-vis detection at λ = 210 nm, t_R = 15.8 min (minor), 16.6 min (major).

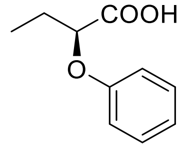

(S)-2-phenoxybutanoic acid (2p).[9] White solid, 80% yield, 93% ee, $[\alpha]_D^{25}$ = -15.57 (c = 0.61, $CHCl_3$). ^1H NMR (400 MHz, Chloroform-*d*) δ 8.80 (brs, 1H), 7.34 – 7.20 (m, 2H), 6.98 (t, *J* = 7.3 Hz, 1H), 6.88 (d, *J* = 7.9 Hz, 2H), 4.57 (t, *J* = 6.0 Hz, 1H), 2.06 – 1.90 (m, 2H), 1.07 (t, *J* = 7.3 Hz, 3H). ^{13}C NMR (101 MHz, Chloroform-*d*) δ 177.3, 157.6, 129.6, 121.8, 115.2, 25.9, 9.6. The enantiomeric excess of **2p** was determined by HPLC analysis on Chiralpak OJ-3 column. Conditions: hexane/isopropanol = 97/3, flow rate = 1.0 mL/min, uv-vis detection at λ = 220 nm, t_R = 12.1 min (minor), 16.5 min (major).

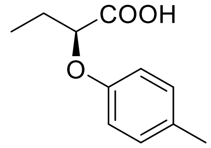

(S)-2-(p-tolyloxy)butanoic acid (2q).[9] White solid, 97% yield, 93% ee, $[\alpha]_D^{25}$ = -22.50 (c = 0.88, $CHCl_3$). ^1H NMR (600 MHz, Chloroform-*d*) δ 7.07 (d, *J* = 7.9 Hz, 2H), 6.80 (d, *J* = 8.1 Hz, 2H), 4.54 (t, *J* = 6.1 Hz, 1H), 2.28 (s, 3H), 1.98 (q, *J* = 7.8 Hz, 2H),

1.07 (t, J = 7.4 Hz, 3H). ^{13}C NMR (151 MHz, Chloroform-*d*) δ 177.3, 155.5, 131.2, 130.0, 115.2, 77.5, 25.9, 20.5, 9.5. The enantiomeric excess of **2q** was determined by HPLC analysis on Chiralpak AD-3 column. Conditions: hexane/isopropanol = 97/3, flow rate = 1.0 mL/min, uv-vis detection at λ = 220 nm, t_R = 8.5 min (major), 9.4 min (minor).

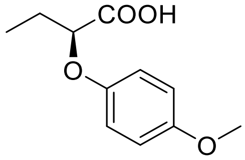

(S)-2-(4-methoxyphenoxy)butanoic acid (2r).[9] White solid, 92% yield, 95% ee, $[\alpha]_D^{25}$ = -35.87 (c = 0.92, $CHCl_3$). ^{1}H NMR (600 MHz, Chloroform-*d*) δ 8.43 (brs, 1H), 6.98 – 6.62 (m, 4H), 4.51 (t, J = 5.4 Hz, 1H), 3.76 (s, 3H), 2.00 (dq, J = 14.3, 6.8 Hz, 2H), 1.10 (t, J = 7.3 Hz, 3H). ^{13}C NMR (151 MHz, Chloroform-*d*) δ 177.2, 154.6, 151.7, 116.6, 114.7, 78.3, 55.7, 26.0, 9.5. The enantiomeric excess of **2r** was determined by HPLC analysis on Chiralpak AD-3 column. Conditions: hexane/isopropanol = 97/3, flow rate = 0.8 mL/min, uv-vis detection at λ = 230 nm, t_R = 20.5 min (major), 22.7 min (minor).

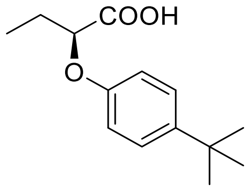

(S)-2-(4-(tert-butyl)phenoxy)butanoic acid (2s).[9] White solid, 93% yield, 93% ee, $[\alpha]_D^{25}$ = -25.56 (c = 0.63, $CHCl_3$). ^{1}H NMR (400 MHz, Chloroform-*d*) δ 8.74 (brs, 1H), 7.30 (d, J = 8.3 Hz, 2H), 6.84 (d, J = 8.3 Hz, 2H), 4.56 (t, J = 5.6 Hz, 1H), 2.08 – 1.91 (m, 2H), 1.29 (s, 9H), 1.09 (t, J = 7.2 Hz, 3H). ^{13}C NMR (101 MHz, Chloroform-*d*) δ 177.3, 155.3, 144.6, 126.4, 114.7, 77.4, 34.1, 31.4, 26.0, 9.6. The enantiomeric excess of **2s** was determined by HPLC analysis on Chiralpak AD-3 column. Conditions: hexane/isopropanol = 99/1, flow rate = 0.5 mL/min, uv-vis detection at λ = 220 nm, t_R = 26.4 min (major), 29.6 min (minor).

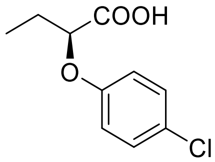

(S)-2-(4-chlorophenoxy)butanoic acid (2t).[9] White solid, 95% yield, 91% ee, $[\alpha]_D^{25}$ = -31.43 (c = 0.28, $CHCl_3$). ^{1}H NMR (600 MHz, Chloroform-*d*) δ 8.89 (brs, 1H), 7.26 – 7.21 (m, 2H), 6.83 (d, *J* = 8.9 Hz, 2H), 4.60 – 4.52 (m, 1H), 2.02 (dh, *J* = 14.3, 7.0 Hz, 2H), 1.10 (t, *J* = 7.4 Hz, 3H). ^{13}C NMR (151 MHz, Chloroform-*d*) δ 177.0, 156.2, 129.5, 126.8, 116.5, 77.4, 26.0, 9.5. The enantiomeric excess of **2t** was determined by HPLC analysis after esterification with $TMSCHN_2$ on Chiralpak OJ-3 column. Conditions: hexane/isopropanol = 99/1, flow rate = 0.5 mL/min, uv-vis detection at λ = 230 nm, t_R = 22.4 min (minor), 37.3 min (major).

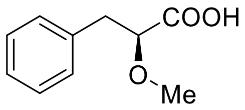

(S)-2-methoxy-3-phenylpropanoic acid (2u).[9] Colorless oil, 89% yield, 99% ee, $[\alpha]_D^{25}$ = -20.00 (c = 0.51, $CHCl_3$). ^{1}H NMR (400 MHz, Chloroform-*d*) δ 8.21 (brs, *J* = 95.3 Hz, 1H), 7.33 – 7.21 (m, 5H), 4.02 (dd, *J* = 8.0, 4.2 Hz, 1H), 3.38 (s, 3H), 3.14 (dd, *J* = 14.2, 4.2 Hz, 1H), 3.02 (dd, *J* = 14.2, 8.1 Hz, 1H). ^{13}C NMR (101 MHz, Chloroform-*d*) δ 176.5, 136.6, 129.3, 128.4, 126.8, 81.3, 58.6, 38.6. The enantiomeric excess of **2u** was determined by HPLC analysis on Chiralpak OJ-3 column. Conditions: hexane/isopropanol = 97/3, flow rate = 1.0 mL/min, uv-vis detection at λ = 210 nm, t_R = 14.8 min (minor), 24.3 min (major).

Asymmetric hydrogenation of *α*-substituted acrylic acids

Method C (for 3a-i).

In an argon-filled glovebox, $CoCl_2$ (0.025 M in THF, 0.2 mL, 0.005 mmol) and (*S,S*)-Ph-BPE (2.53 mg, 0.005 mmol) in HFIP (0.2 mL) were stirred in a vial at room temperature for 10 min. Then zinc dust (0.65 mg, 0.01 mmol) and HFIP (0.2 mL) were added and the mixture was stirred for 15 min. After that, substrate (0.1 mmol) was added to the reaction mixture. The vial was subsequently transferred into an autoclave and purged by three cycles of pressurization/venting with H_2. The reaction was then stirred under H_2 (60 atm) at 50 °C for 48 h. The hydrogen gas was released slowly and carefully. The resulting solution was concentrated in vacuum and the residue was purified by chromatography on silica gel. The ee values were determined by HPLC with a chiral column.

Supplementary Table 6. Solvent screening of **3a**[a]

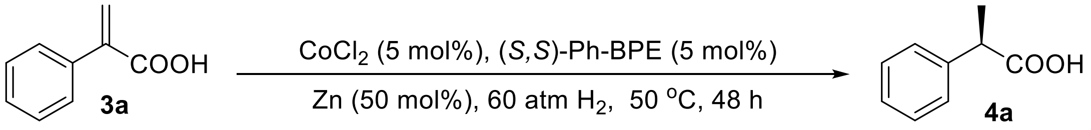

entry	solvent	conv. [%][b]	*ee* [%][c]
1	MeOH	98	73
2	*i*PrOH	21	81
3	*t*BuOH	22	84
4	TFE	>98	82
5	EtOH	69	76
6	*n*PrOH	15	50
7	*n*BuOH	<5	61
8	*i*BuOH	<5	48
9	2-methoxyethanol	ND	--
10	HFIP	>98	98

[a] Reaction conditions: **3a** (0.1 mmol), $CoCl_2$ (5 mol%), (*S,S*)-Ph-BPE (5 mol%) in solvent (0.4 mL) under 60 atm H_2 pressure at 50 °C for 48 h. [b] Determined by ^{1}H NMR. [c] Determined by HPLC analysis.

Characterization data of compound 4

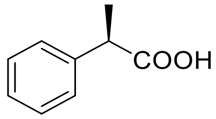

(R)-2-phenylpropanoic acid (4a).[10] Yellowish solid, 90% yield, 98% ee, $[\alpha]_D^{25}$ = -24.04 (c = 0.47, $CHCl_3$). ^{1}H NMR (400 MHz, Chloroform-*d*) δ 10.83 (brs, 1H), 7.35 – 7.22 (m, 5H), 3.72 (q, *J* = 7.2 Hz, 1H), 1.50 (d, *J* = 7.2 Hz, 3H). ^{13}C NMR (151 MHz, Chloroform-*d*) δ 180.9, 139.7, 128.6, 127.6, 127.4, 45.4, 18.1. The enantiomeric excess of **4a** was determined by HPLC analysis on Chiralpak AD-3 column. Conditions: hexane/isopropanol = 97/3, flow rate = 0.8 mL/min, uv-vis detection at λ = 210 nm, t_R = 12.3 min (major), 13.7 min (minor).

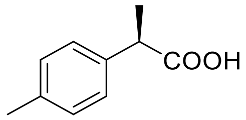

(R)-2-(p-tolyl)propanoic acid (4b).[10] Colorless oil, 98% yield, >99% ee, $[\alpha]_D^{25}$ = -26.91 (c = 0.81, $CHCl_3$). ^{1}H NMR (400 MHz, Chloroform-*d*) δ 7.21 (d, *J* = 8.1 Hz, 2H), 7.14 (d, *J* = 7.9 Hz, 2H), 3.70 (q, *J* = 7.2 Hz, 1H), 2.34 (s, 3H), 1.49 (d, *J* = 7.1 Hz, 3H). ^{13}C NMR (101 MHz, Chloroform-*d*) δ 180.8, 137.0, 136.8, 129.3, 127.4, 44.9, 21.0, 18.1. The enantiomeric excess of **4b** was determined by HPLC analysis on Chiralpak AD-3 column. Conditions: hexane/isopropanol = 97/3, flow rate = 0.8 mL/min, uv-vis detection at λ = 210 nm, t_R = 12.7 min (major), 14.4 min (minor).

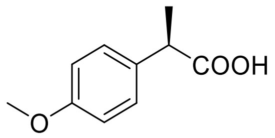

(R)-2-(4-methoxyphenyl)propanoic acid (4c).[10] White solid, 89% yield, >99% ee, $[\alpha]_D^{25}$ = -50.33 (c = 0.61, $CHCl_3$). ^{1}H NMR (400 MHz, Chloroform-*d*) δ 7.24 (d, *J* = 8.7 Hz, 2H), 6.86 (d, *J* = 8.7 Hz, 2H), 3.79 (s, 3H), 3.68 (q, *J* = 7.2 Hz, 1H), 1.48 (d, *J* = 7.2 Hz, 3H). ^{13}C NMR (101 MHz, Chloroform-*d*) δ 180.7, 158.8, 131.9, 128.6, 114.0, 55.2, 44.5, 18.1. The enantiomeric excess of **4c** was determined by HPLC analysis on

Chiralpak AD-3 column. Conditions: hexane/isopropanol = 97/3, flow rate = 0.8 mL/min, uv-vis detection at λ = 220 nm, t_R = 19.4 min (major), 21.5 min (minor).

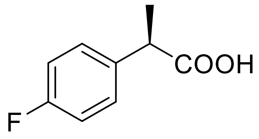

(R)-2-(4-fluorophenyl)propanoic acid (4d).[10] Yellowish oil, 96% yield, >99% ee, $[\alpha]_D^{25}$ = -33.47 (c = 0.75, $CHCl_3$). ^1H NMR (400 MHz, Chloroform-*d*) δ 7.26 (t, *J* = 4.3 Hz, 2H), 7.00 (t, *J* = 8.6 Hz, 2H), 3.69 (q, *J* = 7.0 Hz, 1H), 1.47 (d, *J* = 7.1 Hz, 3H). ^{13}C NMR (101 MHz, Chloroform-*d*) δ 180.3, 162.0 (d, *J* = 245.8 Hz), 135.6 (d, *J* = 3.0 Hz), 129.1 (d, *J* = 8.0 Hz), 115.5 (d, *J* = 21.7 Hz), 44.7, 18.2. ^{19}F NMR (376 MHz, Chloroform-*d*) δ -115.35. The enantiomeric excess of **4d** was determined by HPLC analysis on Chiralpak OJ-3 column. Conditions: hexane/isopropanol = 97/3, flow rate = 0.8 mL/min, uv-vis detection at λ = 210 nm, t_R = 16.3 min (major), 21.5 min (minor).

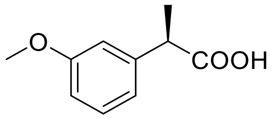

(R)-2-(3-methoxyphenyl)propanoic acid (4e).[11] Colorless oil, 95% yield, 97% ee, $[\alpha]_D^{25}$ = -45.47 (c = 0.53, $CHCl_3$). ^1H NMR (600 MHz, Chloroform-*d*) δ 7.26 – 7.23 (m, 1H), 6.92 – 6.86 (m, 2H), 6.81 (dd, *J* = 8.2, 2.2 Hz, 1H), 3.80 (s, 3H), 3.73 – 3.69 (m, 1H), 1.50 (d, *J* = 7.2 Hz, 3H). ^{13}C NMR (151 MHz, Chloroform-*d*) δ 180.3, 159.7, 141.2, 129.6, 112.0, 113.4, 112.7, 55.2, 45.3, 18.0. The enantiomeric excess of **4e** was determined by HPLC analysis on Chiralpak AD-3 column. Conditions: hexane/isopropanol = 97/3, flow rate = 0.8 mL/min, uv-vis detection at λ = 220 nm, t_R = 16.6 min (major), 20.3 min (minor).

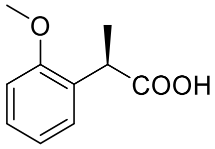

(R)-2-(2-methoxyphenyl)propanoic acid (4f).[12] White solid, 94% yield, 98% ee, [α]

$_D^{25}$ = -61.92 (c = 0.73, $CHCl_3$). ^1H NMR (400 MHz, Chloroform-*d*) δ 7.24 (ddd, *J* = 7.3, 4.0, 1.4 Hz, 2H), 6.94 (t, *J* = 7.1 Hz, 1H), 6.87 (d, *J* = 8.3 Hz, 1H), 4.07 (q, *J* = 7.2 Hz, 1H), 3.82 (s, 3H), 1.47 (d, *J* = 7.2 Hz, 3H). ^{13}C NMR (101 MHz, Chloroform-*d*) δ 180.7, 156.7, 128.8, 128.3, 128.0, 120.8, 110.7, 55.5, 39.1, 16.8. The enantiomeric excess of **4f** was determined by HPLC analysis on Chiralpak AD-3 column. Conditions: hexane/isopropanol = 97/3, flow rate = 0.8 mL/min, uv-vis detection at λ = 220 nm, t_R = 15.9 min (minor), 18.8 min (major).

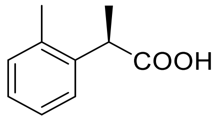

(R)-2-(o-tolyl)propanoic acid (4g).[13] Colorless oil, 89% yield, 90% ee, $[\alpha]_D^{25}$ = -57.27 (c = 0.63, $CHCl_3$). ^1H NMR (600 MHz, Chloroform-*d*) δ 7.28 (d, *J* = 7.2 Hz, 1H), 7.22 – 7.14 (m, 3H), 3.98 (q, *J* = 7.2 Hz, 1H), 2.37 (s, 3H), 1.48 (d, *J* = 7.1 Hz, 3H). ^{13}C NMR (151 MHz, Chloroform-*d*) δ 180.6, 138.4, 135.9, 130.5, 127.1, 126.5, 126.4, 41.2, 19.6, 17.6. The enantiomeric excess of **4g** was determined by HPLC analysis on Chiralpak OJ-3 column. Conditions: hexane/isopropanol = 97/3, flow rate = 0.8 mL/min, uv-vis detection at λ = 210 nm, t_R = 16.4 min (major), 20.8 min (minor).

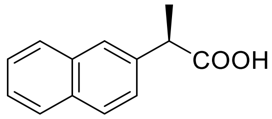

(R)-2-(naphthalen-2-yl)propanoic acid (4h).[13] White solid, 99% yield, 96% ee, $[\alpha]_D^{25}$ = -32.5 (c = 0.60, $CHCl_3$). ^1H NMR (400 MHz, Chloroform-*d*) δ 7.85 – 7.74 (m, 4H), 7.51 – 7.42 (m, 3H), 3.91 (q, *J* = 7.0 Hz, 1H), 1.60 (d, *J* = 7.1 Hz, 3H). ^{13}C NMR (151 MHz, Chloroform-*d*) δ 180.4, 137.2, 133.4, 132.7, 128.4, 127.8, 127.6, 126.3, 126.2, 125.9, 125.7, 45.5, 18.1. The enantiomeric excess of **4h** was determined by HPLC analysis on Chiralpak OJ-3 column. Conditions: hexane/isopropanol = 97/3, flow rate = 0.8 mL/min, uv-vis detection at λ = 230 nm, t_R = 41.5 min (minor), 44.5 min (major).

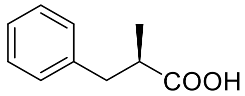

(R)-2-phenylpropanoic acid (4i).[14] Yellowish oil, 94% yield, 80% ee, $[\alpha]_D^{25}$ = -16.22 (c = 0.74, $CHCl_3$). ^{1}H NMR (400 MHz, Chloroform-*d*) δ 7.29 (t, *J* = 7.3 Hz, 2H), 7.21 (dd, *J* = 15.7, 7.2 Hz, 3H), 3.09 (dd, *J* = 13.4, 6.3 Hz, 1H), 2.78 (q, *J* = 7.1 Hz, 1H), 2.67 (dd, *J* = 13.3, 8.0 Hz, 1H), 1.18 (d, *J* = 6.8 Hz, 3H). ^{13}C NMR (101 MHz, Chloroform-*d*) δ 182.3, 139.0, 129.0, 128.4, 126.4, 41.4, 39.1, 16.5. The enantiomeric excess of **4i** was determined by HPLC analysis on Chiralpak OJ-3 column. Conditions: hexane/isopropanol = 97/3, flow rate = 0.8 mL/min, uv-vis detection at λ = 210 nm, t_R = 11.1 min (major), 12.6 min (minor).

Details of applications

Procedure for asymmetric hydrogenation of 1v.

In an argon-filled glovebox, $CoCl_2$ (0.005 M in THF, 0.2 mL, 0.001 mmol) and (*R,R*)-Ph-BPE (0.51 mg, 0.001 mmol) in MeOH (0.2 mL) were stirred in a vial at room temperature for 10 min. Then zinc dust (0.65 mg, 0.01 mmol), MeOH (0.2 mL) were added and the mixture was stirred for 15 min. After that, substrate **1v** (0.1 mmol) and THF (0.6 mL) were added to the reaction mixture. The vial was subsequently transferred into an autoclave and purged by three cycles of pressurization/venting with H_2. The reaction was then stirred under H_2 (60 atm) at 50 °C for 48 h. The hydrogen gas was released slowly and carefully. The resulting solution was concentrated in vacuum and the residue was purified by chromatography on silica gel. The ee values were determined by HPLC with a chiral column.

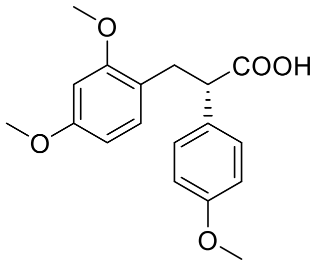

(S)-3-(2,4-dimethoxyphenyl)-2-(4-methoxyphenyl)propanoic acid (2v).[1] White solid, 90% yield, 94% ee, $[\alpha]_D^{25}$ = +76.53 (c = 0.95, $CHCl_3$). ^{1}H NMR (400 MHz, Chloroform-*d*) δ 7.22 (d, *J* = 8.6 Hz, 2H), 6.87 (d, *J* = 8.2 Hz, 1H), 6.82 (d, *J* = 8.6 Hz, 2H), 6.40 (d, *J* = 2.1 Hz, 1H), 6.29 (dd, *J* = 8.2, 2.2 Hz, 1H), 3.88 (t, *J* = 7.3 Hz, 1H), 3.78 (s, 3H), 3.76 (s, 3H), 3.76 (s, 3H), 3.26 (dd, *J* = 13.6, 8.4 Hz, 1H), 2.94 (dd, *J* = 13.6, 6.7 Hz, 1H). ^{13}C NMR (101 MHz, Chloroform-*d*) δ 179.4, 159.5, 158.7, 158.4, 131.0, 130.9, 129.1, 119.4, 113.9, 103.6, 98.3, 55.2, 55.2, 55.2, 50.5, 33.9. The enantiomeric excess of **2v** was determined by HPLC analysis on Chiralpak OD-3 column after esterification with $TMSCHN_2$. Conditions: hexane/isopropanol = 97/3, flow rate = 0.8 mL/min, uv-vis detection at λ = 210 nm, t_R = 11.5 min (minor), 14.8 min (major).

Procedure for asymmetric hydrogenation of 1x.

In an argon-filled glovebox, $CoCl_2$ (0.005 M in THF, 0.2 mL, 0.001 mmol) and (*R,R*)-Ph-BPE (0.51 mg, 0.001 mmol) in *i*BuOH (0.2 mL) were stirred in a vial at room temperature for 10 min. Then zinc dust (0.65 mg, 0.01 mmol), *i*BuOH (0.2 mL) were added and the mixture was stirred for 15 min. After that, substrate **1x** (0.1 mmol) was added to the reaction mixture. The vial was subsequently transferred into an autoclave and purged by three cycles of pressurization/venting with H_2. The reaction was then stirred under H_2 (60 atm) at 50 °C for 72 h. The hydrogen gas was released slowly and carefully. The resulting solution was concentrated in vacuum and the residue was purified by chromatography on silica gel. The ee values were determined by HPLC with a chiral column.

Supplementary Table 7. Solvent screening of **1x**[a]

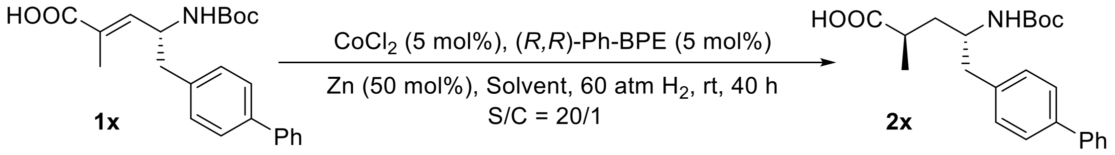

entry	solvent	conv. [%][b]	*dr* [%][c]
1	MeOH	>98	9.0/1
2	*i*PrOH	>98	11.3/1
3	TFE	>98	14.4/1
4	*t*BuOH	97	4.1/1
5	EtOH	>98	9.3/1
6	*n*PrOH	>98	10.5/1
7	*n*BuOH	>98	10.7/1
8	*i*BuOH	>98	17.0/1
9	2-Methoxyethanol	>98	10.0/1
10	HFIP	98	12.0/1

[a] Reaction conditions: **1x** (0.1 mmol), $CoCl_2$ (5 mol%), (*R,R*)-Ph-BPE (5 mol%) in solvent (0.4 mL) under 60 atm H_2 pressure at 50 °C for 40 h. [b] Determined by ^1H NMR. [c] Determined by HPLC analysis.

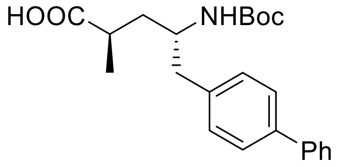

(2R,4S)-5-([1,1'-biphenyl]-4-yl)-4-((tert-butoxycarbonyl)amino)-2-methylpentanoic acid (2x). White solid, 97% yield, 17/1 dr, $[\alpha]_D^{25}$ = +1.49 (c = 0.94, $CHCl_3$). ^{1}H NMR (600 MHz, Chloroform-*d*) δ 7.57 (d, *J* = 7.6 Hz, 2H), 7.52 (t, *J* = 9.0 Hz, 2H), 7.42 (d, *J* = 7.9 Hz, 2H), 7.34 (d, *J* = 7.7 Hz, 1H), 7.26 – 7.22 (m, 2H), 6.33 (d, *J* = 9.5 Hz, 0.5H), 4.56 (d, *J* = 9.1 Hz, 0.5H), 4.09 – 3.88 (m, 1H), 2.95-2.45 (m, 2H), 2.70-2.55 (m, 1H), 1.84 (t, *J* = 11.3 Hz, 0.5H), 1.54 – 1.45 (m, 0.5H), 1.40 (s, 5H), 1.29 (s, 5H), 1.20 (d, *J* = 6.9 Hz, 3H). ^{13}C NMR (151 MHz, Chloroform-*d*) δ 180.8, 179.4, 157.1, 156.4, 141.1, 140.8, 139.5, 139.2, 137.7, 136.3, 129.8, 128.7, 127.2, 127.0, 127.0, 80.3, 80.2, 68.1, 50.8, 49.7, 42.1, 41.1, 39.5, 38.2, 36.5, 36.2, 31.5, 30.1, 29.7, 28.3, 28.0, 17.8, 15.9. HRMS calculated $[M-H]^-$ for $C_{23}H_{28}NO_4^-$ = 382.2024, found: 382.2022. The enantiomeric excess of **2x** was determined by HPLC analysis on Chiralpak AS-3 column. Conditions: hexane/isopropanol = 92/8, flow rate = 0.9 mL/min, uv-vis detection at λ = 210 nm, t_R = 11.2 min (major), 13.8 min (minor).

Procedure for asymmetric hydrogenation of 1w, 3j.

In an argon-filled glovebox, $CoCl_2$ (0.025 M in THF, 0.2 mL, 0.005 mmol) and (*R,R*)-Ph-BPE (2.53 mg, 0.005 mmol) in MeOH (0.2 mL) were stirred in a vial at room temperature for 10 min. Then zinc dust (0.65 mg, 0.01 mmol) and MeOH (0.2 mL) were added and the mixture was stirred for 15 min. After that, substrate (0.1 mmol) and HFIP (0.2 mL) were added to the reaction mixture. The vial was subsequently transferred into an autoclave and purged by three cycles of pressurization/venting with H_2. The reaction was then stirred under H_2 (60 atm) at 50 °C. The hydrogen gas was released slowly and carefully. The resulting solution was concentrated in vacuum and the residue was purified by chromatography on silica gel. The ee values were determined by HPLC with a chiral column.

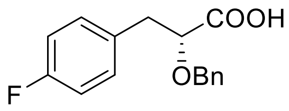

(R)-2-(benzyloxy)-3-(4-fluorophenyl)propanoic acid (2w).[9] White solid, 95% yield, >99% ee, $[\alpha]_D^{25}$ = +23.97 (c = 0.58, $CHCl_3$). ^1H NMR (400 MHz, Chloroform-*d*) δ 7.31 – 7.25 (m, 3H), 7.22 – 7.12 (m, 4H), 6.97 (t, *J* = 8.7 Hz, 2H), 4.67 (d, *J* = 11.7 Hz, 1H), 4.40 (d, *J* = 11.7 Hz, 1H), 4.14 (dd, *J* = 8.2, 3.9 Hz, 1H), 3.20 – 2.93 (m, 2H). ^{13}C NMR (101 MHz, Chloroform-*d*) δ 187.9, 161.9 (d, *J* = 245.0 Hz), 136.6, 132.3 (d, *J* = 3.0 Hz), 131.0 (d, *J* = 8.0 Hz), 128.5, 128.1, 127.9, 115.2 (d, *J* = 21.3 Hz), 78.5, 72.8, 38.0. ^{19}F NMR (565 MHz, Chloroform-*d*) δ -116.17. The enantiomeric excess of **2w** was determined by HPLC analysis on Chiralpak OJ-3 column. Conditions: hexane/isopropanol = 97/3, flow rate = 0.8 mL/min, uv-vis detection at λ = 210 nm, t_R = 22.6 min (major), 28.2 min (minor).

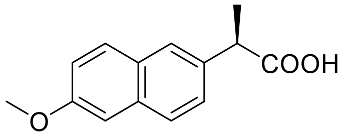

(R)-2-(6-methoxynaphthalen-2-yl)propanoic acid (4j).[14] White solid, 87% yield, 97% ee, $[\alpha]_D^{25}$ = -17.34 (c = 0.56, $CHCl_3$). ^1H NMR (400 MHz, Chloroform-d) δ 7.70 (d, J = 9.6 Hz, 3H), 7.41 (dd, J = 8.5, 1.7 Hz, 1H), 7.16 – 7.08 (m, 2H), 3.91 (s, 3H), 3.90 – 3.83 (m, 1H), 1.58 (d, J = 7.1 Hz, 3H). ^{13}C NMR (101 MHz, Chloroform-d) δ 180.4, 157.6, 134.9, 133.8, 129.3, 128.8, 127.2, 126.2, 126.1, 119.0, 105.5, 55.3, 45.3, 18.1. The enantiomeric excess of **4f** was determined by HPLC analysis on Chiralpak OJ-3 column. Conditions: hexane/isopropanol = 97/3, flow rate = 0.8 mL/min, uv-vis detection at λ = 230 nm, t_R = 83.0 min (minor), 97.2 min (major).

Procedure for asymmetric hydrogenation of 3k.

In an argon-filled glovebox, $CoCl_2$ (0.005 M in THF, 0.2 mL, 0.001 mmol) and (*R,R*)-Ph-BPE (0.51 mg, 0.001 mmol) in HFIP (0.2 mL) were stirred in a vial at room temperature for 10 min. Then zinc dust (0.65 mg, 0.01 mmol) and HFIP (0.2 mL) were added and the mixture was stirred for 15 min. After that, substrate (0.1 mmol) was

added to the reaction mixture. The vial was subsequently transferred into an autoclave and purged by three cycles of pressurization/venting with H_2. The reaction was then stirred under H_2 (60 atm) at 50 °C for 72 h. The hydrogen gas was released slowly and carefully. The resulting solution was concentrated in vacuum and the residue was purified by chromatography on silica gel. The ee values were determined by HPLC with a chiral column.

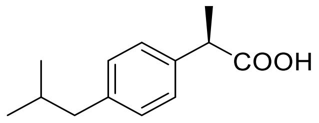

(R)-2-(4-isobutylphenyl)propanoic acid (4k).[10] White solid, 89% yield, 99% ee, $[\alpha]_D^{25}$ = -3.46 (c = 1.04, $CHCl_3$). ^{1}H NMR (400 MHz, Chloroform-*d*) δ 7.22 (d, *J* = 8.1 Hz, 2H), 7.10 (d, *J* = 8.1 Hz, 2H), 3.71 (q, *J* = 7.1 Hz, 1H), 2.45 (d, *J* = 7.2 Hz, 2H), 1.84 (dp, *J* = 13.6, 6.8 Hz, 1H), 1.50 (d, *J* = 7.2 Hz, 3H), 0.90 (d, *J* = 6.6 Hz, 6H). ^{13}C NMR (101 MHz, Chloroform-*d*) δ 180.6, 140.8, 137.0, 129.4, 127.3, 45.0, 44.9, 30.2, 22.4, 18.1. The enantiomeric excess of **4k** was determined by HPLC analysis after esterification with $TMSCHN_2$ on Chiralpak AD-3 column. Conditions: hexane/isopropanol = 99/1, flow rate = 0.5 mL/min, uv-vis detection at λ = 220 nm, t_R = 8.0 min (minor), 8.3 min (major).

Procedure for asymmetric hydrogenation of 5.

In an argon-filled glovebox, $CoCl_2$ (0.025 M in THF, 1.0 mL, 0.025 mmol) and (*S,S*)-Ph-BPE (12.7 mg, 0.025 mmol) in MeOH (1.0 mL) were stirred in a vial at room temperature for 10 min. Then zinc dust (16.4 mg, 0.25 mmol) was added and the mixture was stirred for 15 min. After that, substrate **5** (5 mmol), MeOH (11.0 mL) and HFIP (1.0 mL) were added to the reaction mixture. The vial was subsequently transferred into an autoclave and purged by three cycles of pressurization/venting with H_2. The reaction was then stirred under H_2 (30 atm) at 50 °C for 72 h. The hydrogen gas was released slowly and carefully. The resulting solution was concentrated in vacuum and the residue was purified by chromatography on silica gel. The ee values were determined by HPLC with a chiral column.

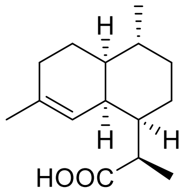

(R)-Dihydroartemisinic acid (6).[11] White solid, 98% yield, 97/3 dr, $[\alpha]_D^{25}$ = -8.84 (c = 0.85, $CHCl_3$). ^{1}H NMR (600 MHz, Chloroform-*d*) δ 5.12 (s, 1H), 2.53 – 2.46 (m, 2H), 1.97 – 1.87 (m, 2H), 1.84 – 1.77 (m, 1H), 1.66 – 1.51 (m, 6H), 1.47 – 1.38 (m, 2H), 1.28 – 1.24 (m, 1H), 1.19 (d, *J* = 6.9 Hz, 3H), 1.16 – 1.07 (m, 1H), 0.96 (qd, *J* = 12.9, 2.9 Hz, 1H), 0.87 (d, *J* = 6.5 Hz, 3H). ^{13}C NMR (151 MHz, Chloroform-*d*) δ 183.6, 136.0, 119.3, 43.6, 42.2, 41.7, 36.3, 35.2, 27.6, 27.4, 26.6, 25.7, 23.8, 19.7, 15.1. The diastereomeric ratio of compound **6** was determined by HPLC analysis with Agilent Poroshell 120, EC-C18 (2.7um) column. Conditions: gradient elution with ACN /H_2O (with 1.0% FA) (0-1 Min: ACN/H_2O=20/80; 1-35 Min: ACN/H_2O= 99/1 to 20/80; 35-40 Min: ACN/H_2O=20/80), flow rate = 0.5 mL/min, uv-vis detection at 210 nm, t_R = 32.2 min (major), 32.7 min (minor).

Supplementary Note 1

Control experiments

To provide insight into the possible catalyst activation mode and the mechanism of the asymmetric hydrogenation of *α,β*-unsaturated carboxylic acids, several control and catalytic experiments were conducted. Only starting material was observed suggesting that no reaction occurred for the corresponding ethyl ester **1b'** under standard conditions. Moreover, no hydrogenation product was observed when 1 mol% of CH_3COOH or **1b** was added to the reaction mixture as external carboxylic acid.

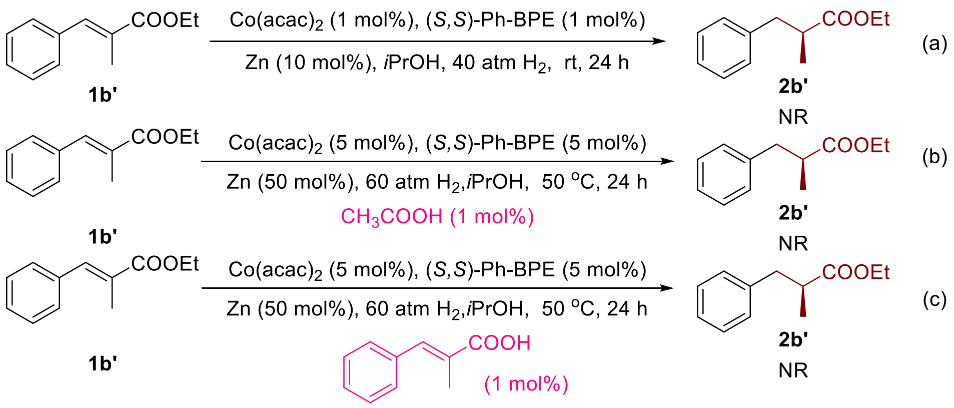

Supplementary Figure 1. Control experiments. (a): the asymmetric hydrogenation of ethyl ester **1b'** under standard condition; (b): with 1 mol% CH_3COOH as additives; (c) with 1 mol% of **1b** as additives.

Supplementary Note 2

Deuterium-Labeling experiment

Hydrogenation of 1b in isopropanol-d_8 and/or CH_3COOD.

The reactions using isopropanol-d_8 as solvent were conducted, and no deuteration was observed with or without Zn as activator. Addition of CH_3CO_2D (5 eq) as additive could not produce the deuterated product either, indicating that protonation of the Co-alkyl intermediate was probably not involved in the current reaction (**Supplementary Figure 2 and 3**). HRMS calculated [**2b**-H]$^-$ for $C_{10}H_{11}O_2^-$ = 163.0765, found 163.0755.

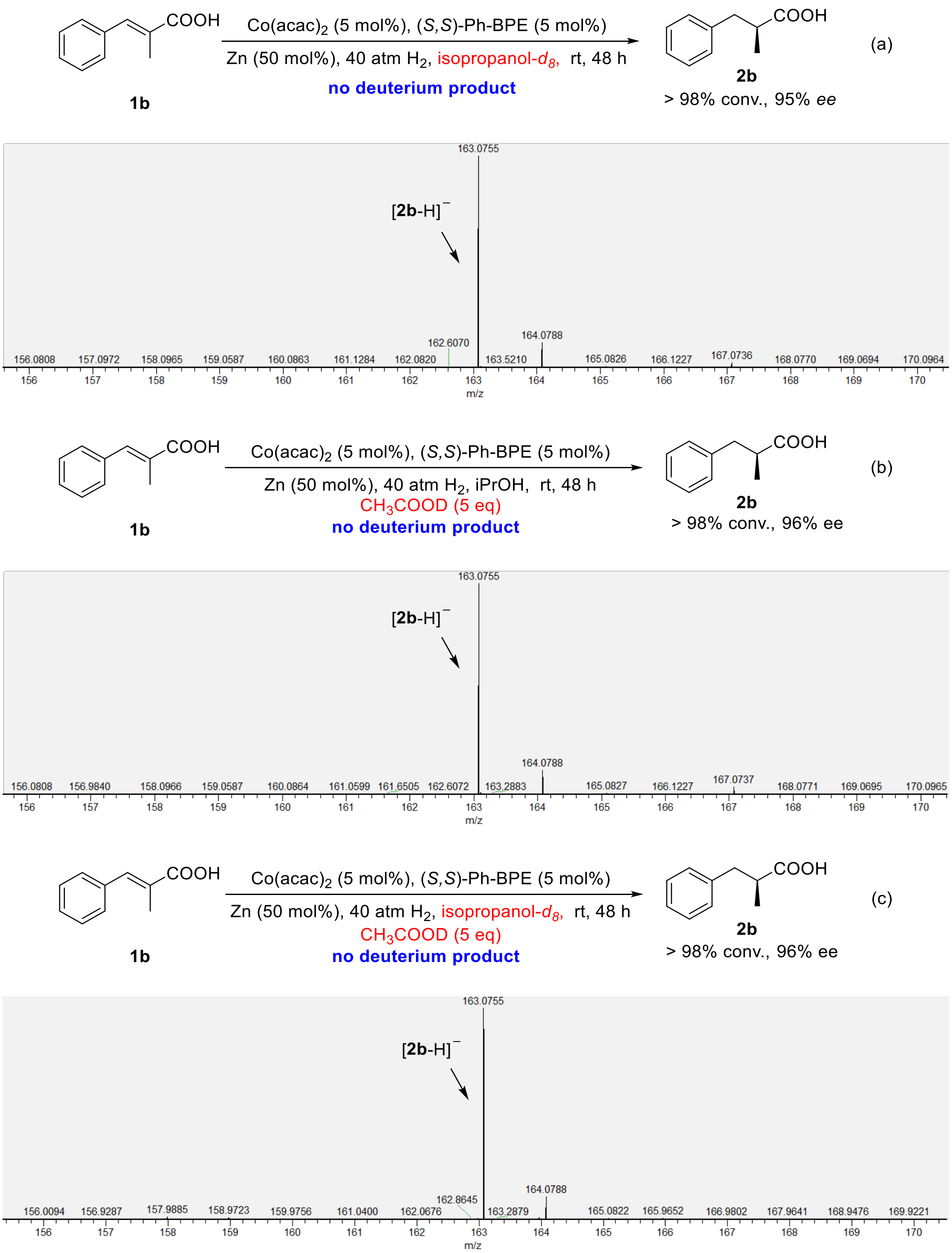

Supplementary Figure 2. Deuterium-labeling experiment in the presence of Zn and the corresponding HR-MS spectrums. (a): in isopropanol-d_8; (b): with 5 eq. CH_3COOD as additives; (c) with 5 eq. CH_3COOD as additives in isopropanol-d_8.

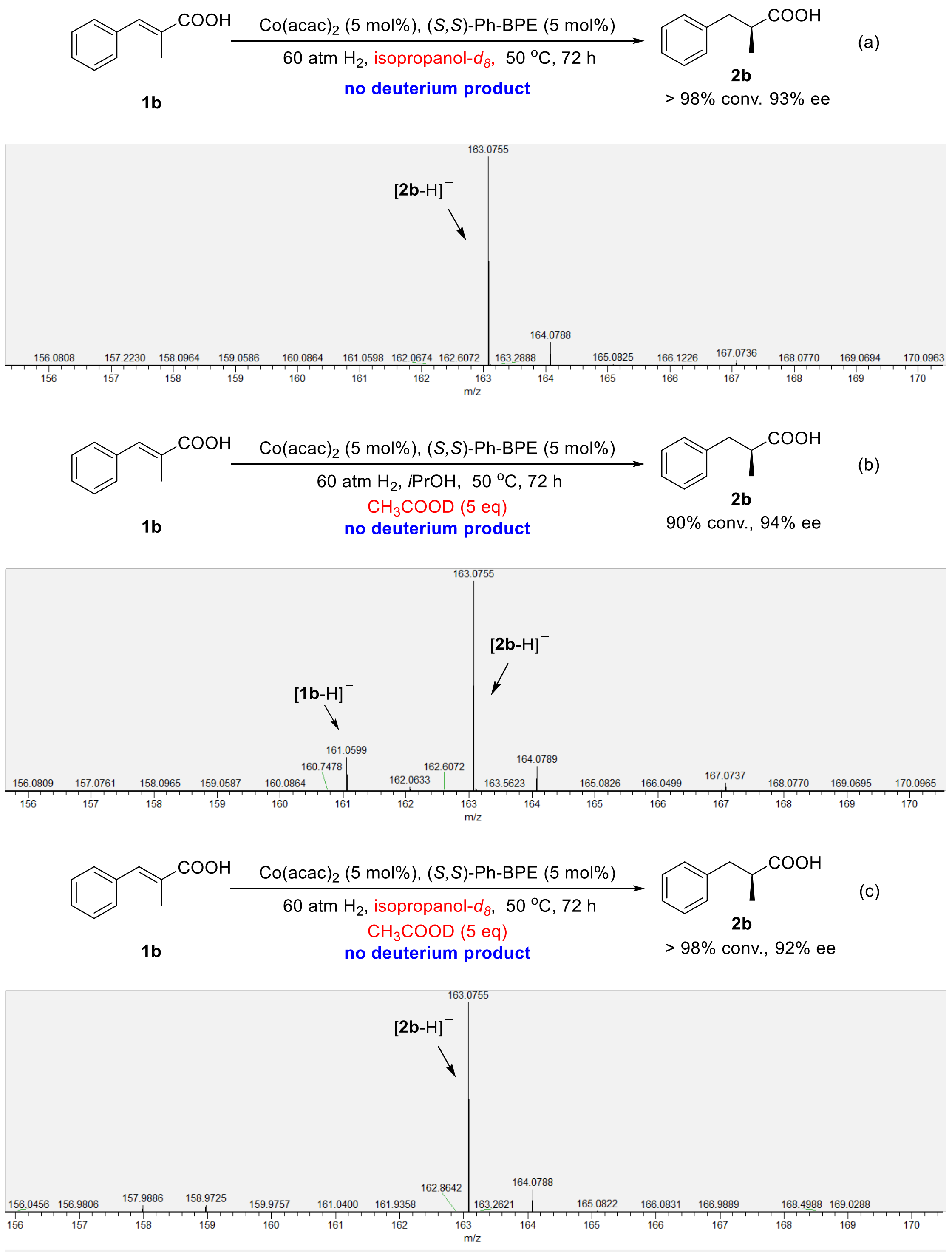

Supplementary Figure 3. Deuterium-labeling experiment in the absence of Zn and the corresponding HR-MS spectrums. (a): in isopropanol-d_8; (b): with 5 eq. CH_3COOD as additives; (c) with 5 eq. CH_3COOD as additives in isopropanol-d_8.

Hydrogenation of 1b with D_2.

In an argon-filled glovebox, $Co(acac)_2$ (0.050 M in *i*PrOH, 0.10 mL, 0.005 mmol) and (*S,S*)-Ph-BPE (0.050 M in THF, 0.10 mL, 0.005 mmol) were stirred in a vial at room temperature for 10 min. Then zinc dust (3.3 mg, 0.05 mmol) and *i*PrOH (0.50 mL) were added and the mixture was stirred for 15 min. After that, **1b** (0.1 mmol) was added to the reaction mixture. The vial was subsequently transferred into an autoclave and purged by three cycles of pressurization/venting with D_2. The reaction was then stirred under D_2 (40 atm) at room temperature for 48 h. The gas was released slowly and carefully. The resulting solution was concentrated in vacuum and the residue was purified by chromatography on silica gel. The deuterium-labeling experiment using D_2 gas indicates H_2 as the hydrogen donor. HRMS calculated {**2b-*d*$_2$**]-H}$^-$ for $C_{10}H_9D_2O_2^-$ = 165.0890, found 165.0877.

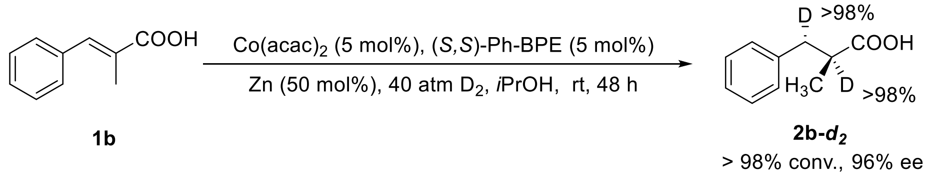

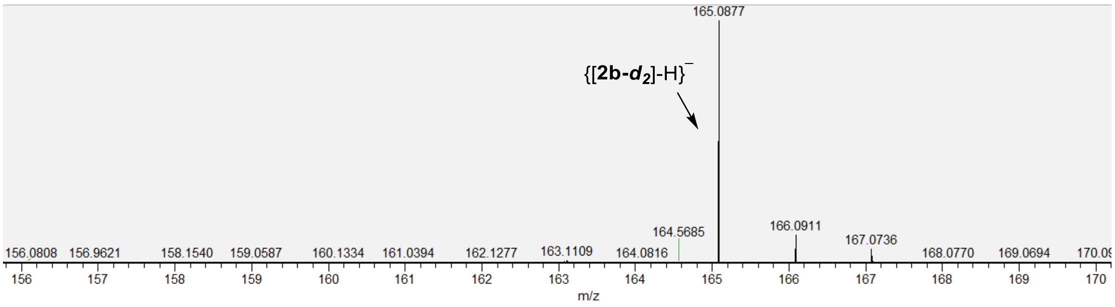

Supplementary Figure 4. HR-MS of Deuterium-Labeling Experiment of **1b** with D_2.

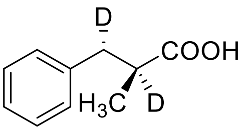

[2b-*d*$_2$][15] White solid, 94% yield, ^{1}H NMR (600 MHz, Chloroform-*d*) δ 7.29 (t, *J* = 7.4 Hz, 2H), 7.21 (dd, *J* = 23.0, 7.4 Hz, 3H), 2.66 (s, 1H), 1.18 (s, 3H).

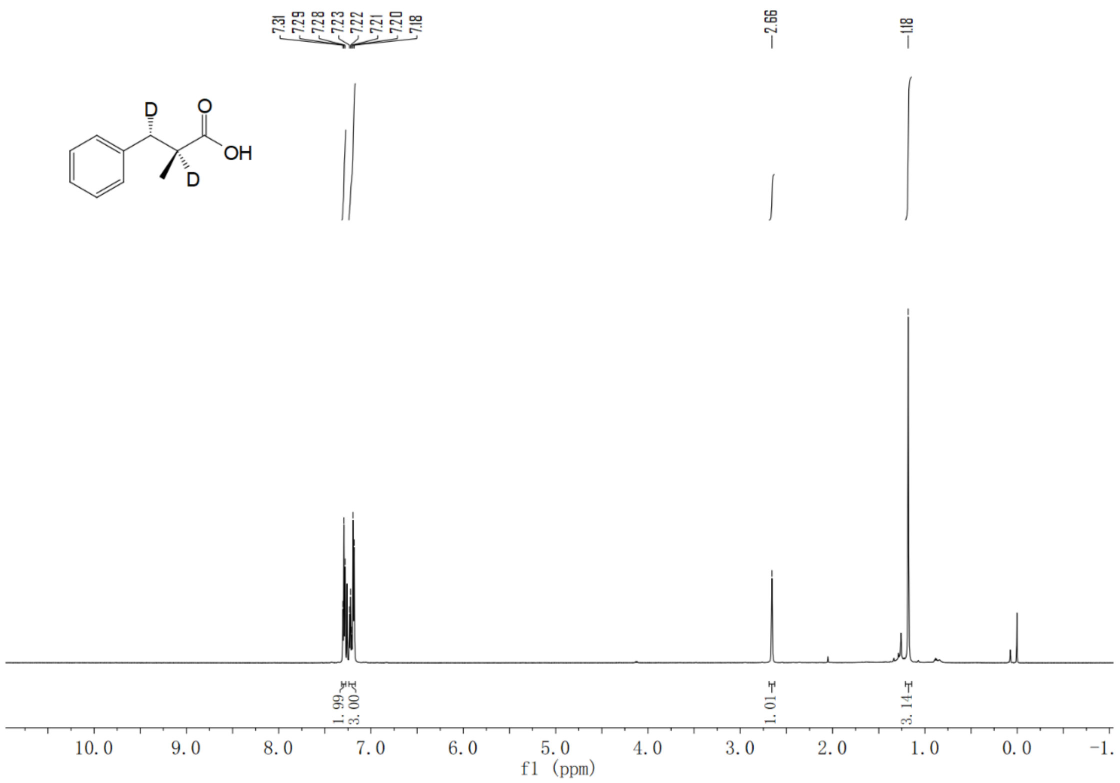

Supplementary Figure 5. ^{1}H NMR (600 MHz, $CDCl_3$) of **2b-*d*$_2$**

Hydrogenation of 1b with H_2/D_2 in the presence of Zn.

In an argon-filled glovebox, Co(acac)$_2$ (0.050 M in *i*PrOH, 0.10 mL, 0.005 mmol) and (*S,S*)-Ph-BPE (0.050 M in THF, 0.10 mL, 0.005 mmol) were stirred in a vial at room temperature for 10 min. Then zinc dust (3.3 mg, 0.05 mmol) and *i*PrOH (0.50 mL) were added and the mixture was stirred for 15 min. After that, **1b** (0.1 mmol) was added to the reaction mixture. The vial was subsequently transferred into an autoclave and purged by three cycles of pressurization/venting with H_2. The reaction was then stirred under H_2/D_2 (40 atm) at room temperature for 48 h. The gas was released slowly and carefully. The resulting solution was concentrated in vacuum and the residue was purified by chromatography on silica gel. HRMS calculated [**2b**-H]$^-$ for $C_{10}H_{11}O_2^-$ = 163.0765, found 163.0752. HRMS calculated {**2b-*d*$_1$**]-H}$^-$ for $C_{10}H_{10}DO_2^-$ = 164.0827, found 164.0815. HRMS calculated {**2b-*d*$_2$**]-H}$^-$ for $C_{10}H_9D_2O_2^-$ = 165.0890, found 165.0877.

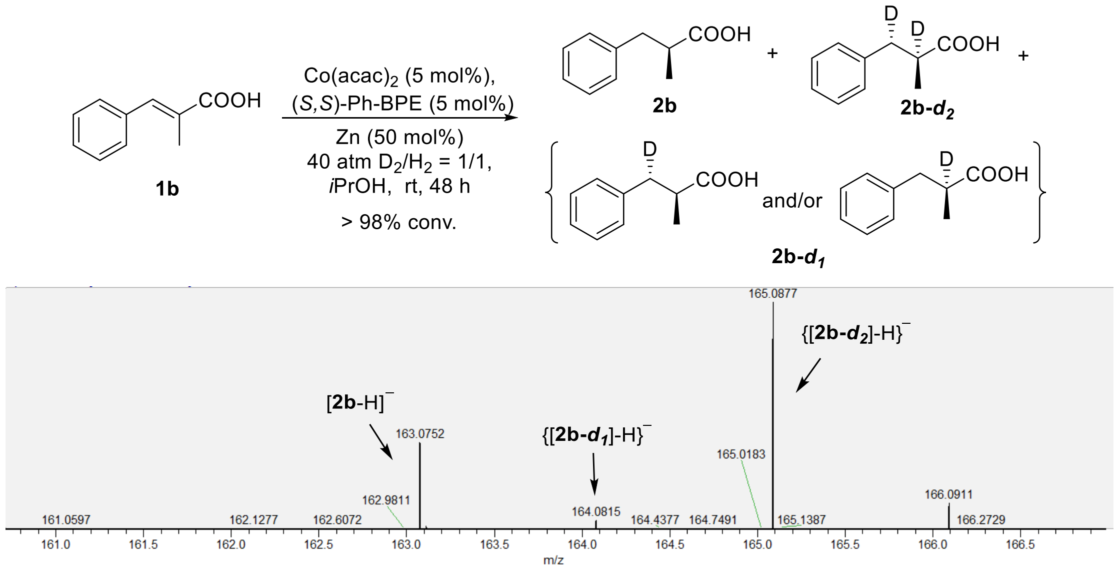

Supplementary Figure 6. The reaction of **1b** with H_2/D_2 in the presence of Zn.

Hydrogenation of 1b with H_2/D_2 in the absence of Zn.

In an argon-filled glovebox, $Co(acac)_2$ (0.050 M in *i*PrOH, 0.10 mL, 0.005 mmol) and (*S,S*)-Ph-BPE (0.050 M in THF, 0.10 mL, 0.005 mmol) were stirred in a vial at room temperature for 10 min. After that, **1b** (0.1 mmol) and *i*PrOH (0.50 mL) were added to the reaction mixture. The vial was subsequently transferred into an autoclave and purged by three cycles of pressurization/venting with H_2. The reaction was then stirred under H_2/D_2 (60 atm) at 50 °C for 72 h. The gas was released slowly and carefully. The resulting solution was concentrated in vacuum and the residue was purified by chromatography on silica gel. HRMS calculated [**2b**-H]$^-$ for $C_{10}H_{11}O_2^-$ = 163.0765, found 163.0753. HRMS calculated {**2b-*d*$_1$**]-H}$^-$ for $C_{10}H_{10}DO_2^-$ = 164.0827, found 164.0816. HRMS calculated {**2b-*d*$_2$**]-H}$^-$ for $C_{10}H_9D_2O_2^-$ = 165.0890, found 165.0878.

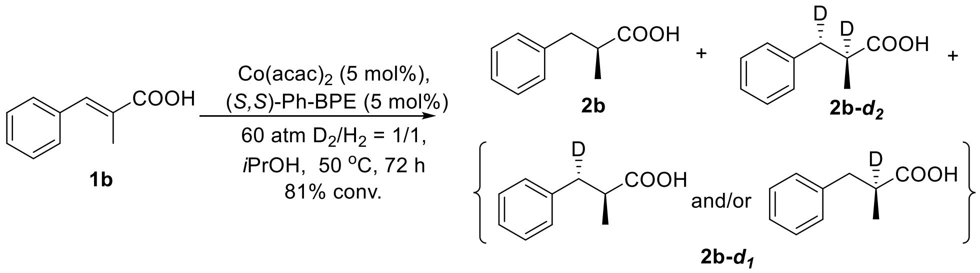

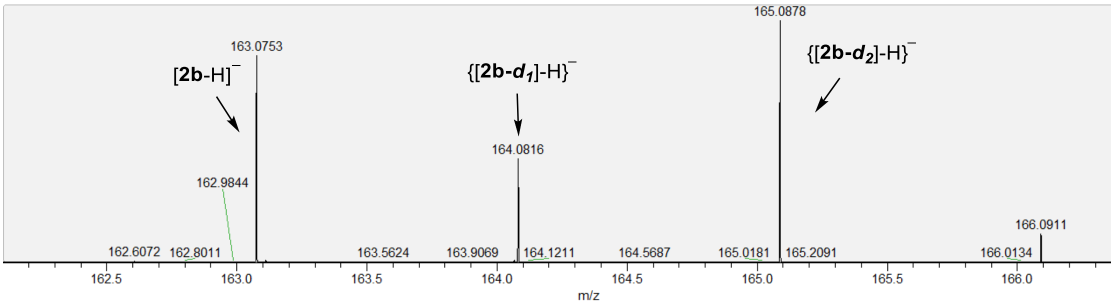

Supplementary Figure 7. The reaction of **1b** with H_2/D_2 in the absence of Zn.

Supplementary Note 3

EPR experiments

Procedures for EPR experiments.

(1) In an argon-filled glovebox, Co(acac)$_2$ (1.3 mg, 0.005 mmol), (*S,S*)-Ph-BPE (2.53 mg, 0.005 mmol) and THF (1 mL) were stirred in a vial at room temperature for 10 min. The reaction mixture (0.2 mL) and THF (0.2 mL) were added to a quartz tube. The quartz tube was then glassed in liquid helium and subjected to X-band EPR analysis (**Supplementary Figure 9**).

(2) In an argon-filled glovebox, Co(acac)$_2$ (1.3 mg, 0.005 mmol), (*S,S*)-Ph-BPE (2.53 mg, 0.005 mmol) and toluene (1 mL) were stirred in a vial at room temperature for 10 min. After that, **1b** (1.6 mg, 0.01 mmol) was added to the reaction mixture and stirred for 10 min. The reaction mixture (0.2 mL) and toluene (0.2 mL) were added to a quartz tube. The quartz tube was then glassed in liquid nitrogen and subjected to X-band EPR analysis (**Supplementary Figure 10**).

(3) In an argon-filled glovebox, Co(acac)$_2$ (0.050 M in *i*PrOH, 0.10 mL, 0.005 mmol) and (*S,S*)-Ph-BPE (0.050 M in THF, 0.10 mL, 0.005 mmol) were stirred in a vial at room temperature for 10 min. Then zinc dust (3.3 mg, 0.05 mmol) and *i*PrOH (0.50 mL) were added and the mixture was stirred for 15 min. After that, **1b** (0.1 mmol) was added to the reaction mixture. The vial was subsequently transferred into an autoclave and purged by three cycles of pressurization/venting with H_2. The reaction was then stirred under H_2 (40 atm) at room temperature for 30 min. The gas was released slowly and carefully. The solvent was removed under vacuum and the autoclave was then transformed to glovebox. The residue was dissolved in toluene (1 mL) and filtrated through celite to remove the Zn. After that the filtrate (0.2 mL) and toluene (0.2 mL) were added to a quartz tube. The quartz tube was then glassed in liquid nitrogen and subjected to X-band EPR analysis (**Supplementary Figure 11**).

(4) In an argon-filled glovebox, Co(acac)$_2$ (0.050 M in *i*PrOH, 0.10 mL, 0.005 mmol)

and (*S,S*)-Ph-BPE (0.050 M in THF, 0.10 mL, 0.005 mmol) were stirred in a vial at room temperature for 10 min. After that, **1b** (0.1 mmol) was added to the reaction mixture. The vial was subsequently transferred into an autoclave and purged by three cycles of pressurization/venting with H_2. The reaction was then stirred under H_2 (60 atm) at 50 °C for 30 min. The gas was released slowly and carefully. The solvent was removed under vacuum and the autoclave was then transformed to glovebox. The residue was dissolved in toluene (1 mL). The solution (0.2 mL) and toluene (0.2 mL) were added to a quartz tube. The quartz tube was then glassed in liquid nitrogen and subjected to X-band EPR analysis (**Supplementary Figure 12**).

(5) In an argon-filled glovebox, bis(2-ethylhexanoate)cobalt (2.7 mg, 0.005 mmol, 65 wt. % in mineral spirits), (*S,S*)-Ph-BPE (2.56 mg, 0.005 mmol) and THF (1 mL) were stirred in a vial at room temperature for 10 min. The reaction mixture (0.2 mL) and THF (0.2 mL) were added to a quartz tube. The quartz tube was then glassed in liquid helium and subjected to X-band EPR analysis (**Supplementary Figure 13**).

The EPR spectrum of $Co(acac)_2$, $Co(acac)_2$+BPE and $Co(acac)_2$+BPE+substrate were recorded, and the EPR signal changed greatly after the addition of BPE and the substrate. The spectrum of $Co(acac)_2$+BPE+substrate [$Co(BPE)(O_2CR)_2$] was very similar to that of BPE+Cobalt(II)(2-ethylhexanoate)$_2$, indicating that coordination of BPE to $Co(acac)_2$ and substitution of acac by the substrate probably happened. The reaction mixture in the presence of H_2 after 30 min was also monitored with EPR, and the EPR spectra were very similar to that of [$Co(BPE)(O_2CR)_2$], indicating that the formation of $Co(BPE)(O_2CR)_2$ species during hydrogenation reaction and which was probably an off-cycle resting state in the current reaction.

Field strength: 500-4500 G

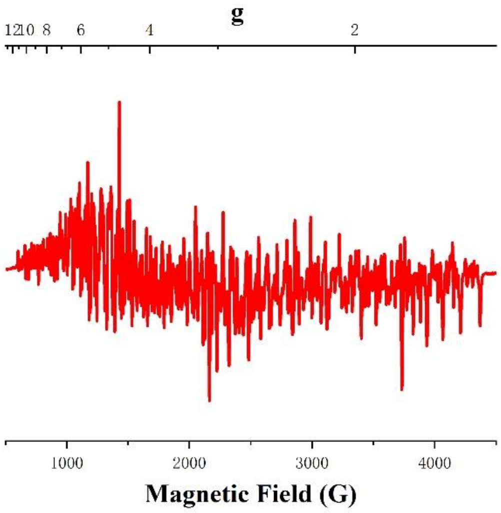

Supplementary Figure 8. EPR spectrum of $Co(acac)_2$ in THF glass at 4.8 K. Field strength window = 500-4500 G. Frequency = 9.374998 GHz; modulation frequency = 100 kHz; modulation amplitude = 5 G; microwave power = 0.04 mW.

Field strength: 500-4500 G

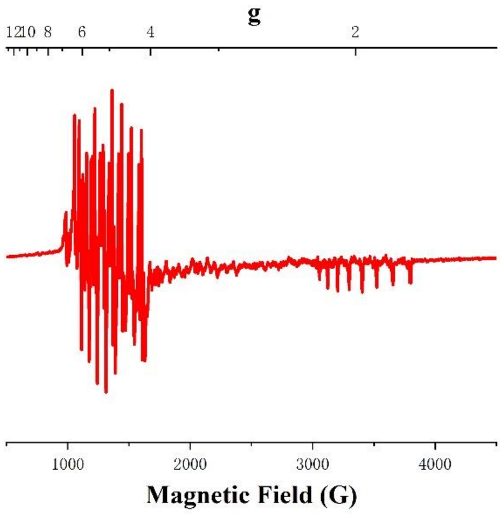

Supplementary Figure 9. EPR spectrum of (1) in THF glass at 4.8 K. Field strength window = 500-4500 G. Frequency = 9.373563 GHz; modulation frequency = 100 kHz; modulation amplitude = 5 G; microwave power = 0.006 mW.

(a) Field strength window = 500-4500 G. (b) Field strength window = 2600-3700 G.

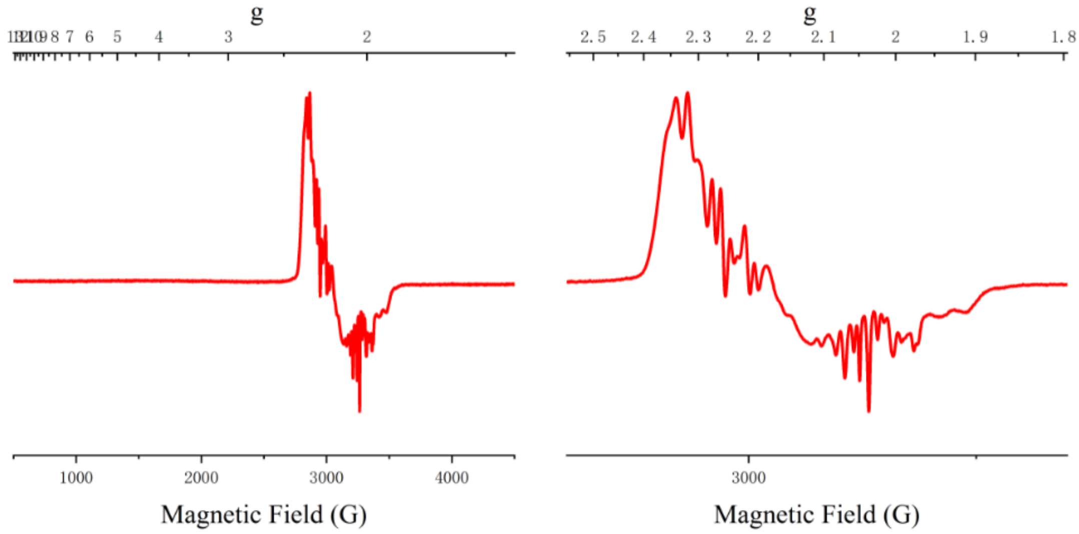

Supplementary Figure 10. EPR spectrum of (2) in toluene glass at 100 K. (a): Field strength window = 500-4500 G; (b): Field strength window = 2600-3700 G. Frequency = 9.301772 GHz; modulation frequency = 100 kHz; modulation amplitude = 4 G; microwave power = 2.0 mW.

(a) Field strength window = 500-4500 G. (b) Field strength window = 2600-3700 G.

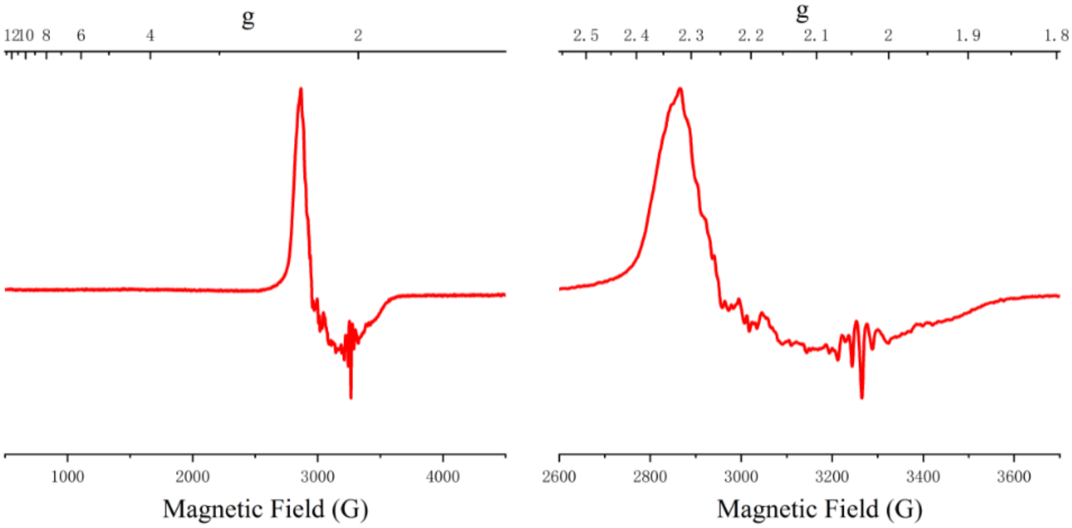

Supplementary Figure 11. EPR spectrum of (3) in toluene glass at 100 K. (a): Field strength window = 500-4500 G; (b): Field strength window = 2600-3700 G. Frequency = 9.301210 GHz; modulation frequency = 100 kHz; modulation amplitude = 4 G; microwave power = 2.0 mW.

(a) Field strength window = 500-4500 G. (b) Field strength window = 2600-3700 G.

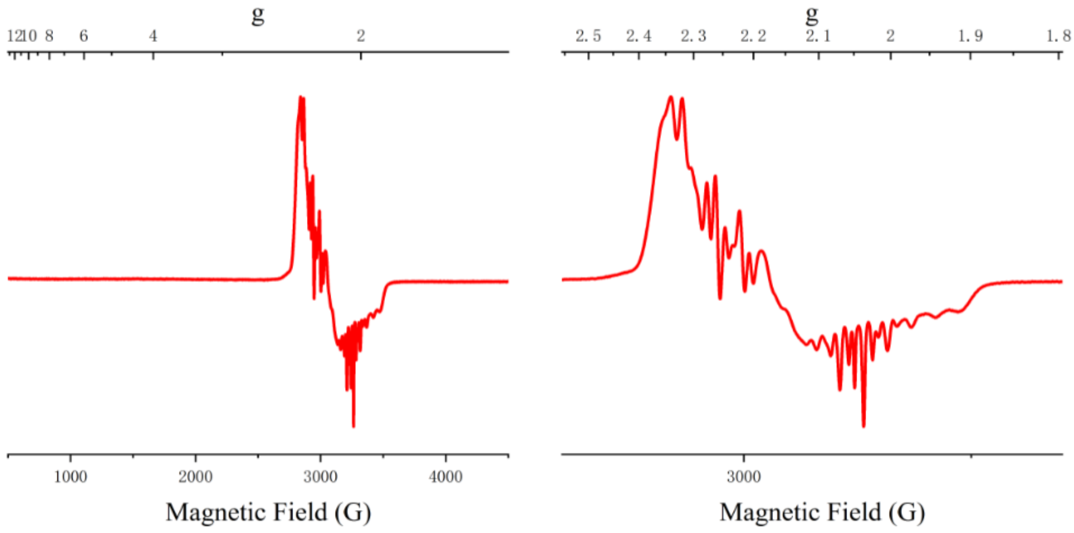

Supplementary Figure 12. EPR spectrum of (4) in toluene glass at 100 K. (a): Field strength window = 500-4500 G; (b): Field strength window = 2600-3700 G. Frequency = 9.303780 GHz; modulation frequency = 100 kHz; modulation amplitude = 4 G; microwave power = 2.0 mW.

(a) Field strength window = 500-4500 G. (b) Field strength window = 2600-3700 G.

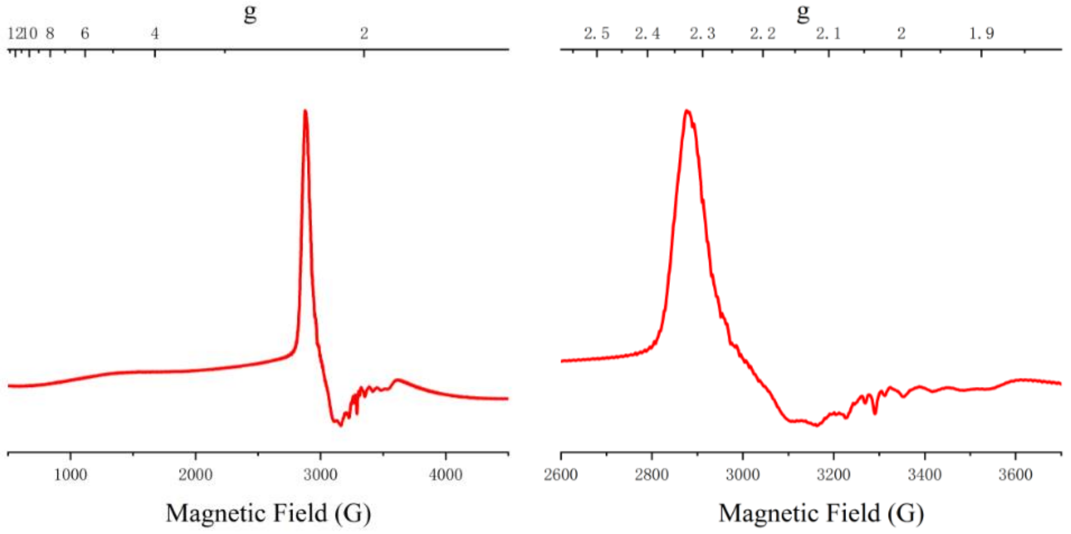

Supplementary Figure 13. EPR spectrum of (5) in THF glass at 4.8 K. (a): Field strength window = 500-4500 G; (b): Field strength window = 2600-3700 G. Frequency = 9.373040 GHz; modulation frequency = 100 kHz; modulation amplitude = 5 G; microwave power = 0.025 mW.

Supplementary Note 4

Plausible mechanism

Based on experimental observations, the plausible mechanism was proposed in **Supplementary Figure 14**. The key catalytic intermediate **E** could be generated through two pathways. In the absence of Zn, complex **E** can be generated through carboxy group mediated H_2 heterolytic process. Coordination of $Co(acac)_2$ with (*S*,*S*)-Ph-BPE generates Co(II) complex **A**, which then undergoes ligand exchange with more acidic substrate **1b** to produce complex **B**, complex **B** and **B'** are in equilibrium with each other. Heterolysis of H_2 by complex **B'** produces the key catalytic species **E** via transition state **C**. However, **E** may be produced through protonation of dihydride complex **D** with **1b** when employing one-electron reductant. The key intermediate **E** then enters the catalytic cycle (Figure S12, left). Intramolecular migratory insertion of complex **E** produces five-membered intermediate **F**. Coordination of H_2 to **F** forms complex **G**, which undergoes subsequent sigma-bond metathesis to give complex **H**. The ligand exchange of intermediate **H** with unsaturated carboxylate substrate releases the hydrogenation product **2b** and regenerates the cobalt hydride complex **D**. Chirik and coworkers reported an alternative mechanism which involved the migratory insertion of the dihydride complex and the subsequent reductive elimination as key step (Figure S12, right).[15] The cobalt dihydride species **D** generated in situ first underwent migratory insertion to form cobalt-alkyl intermediate **I** and the product was produced after subsequent reductive elimination. The cobalt(0) species **J** then underwent H_2 oxidative addition and regenerated the cobalt dihydride species **D**.

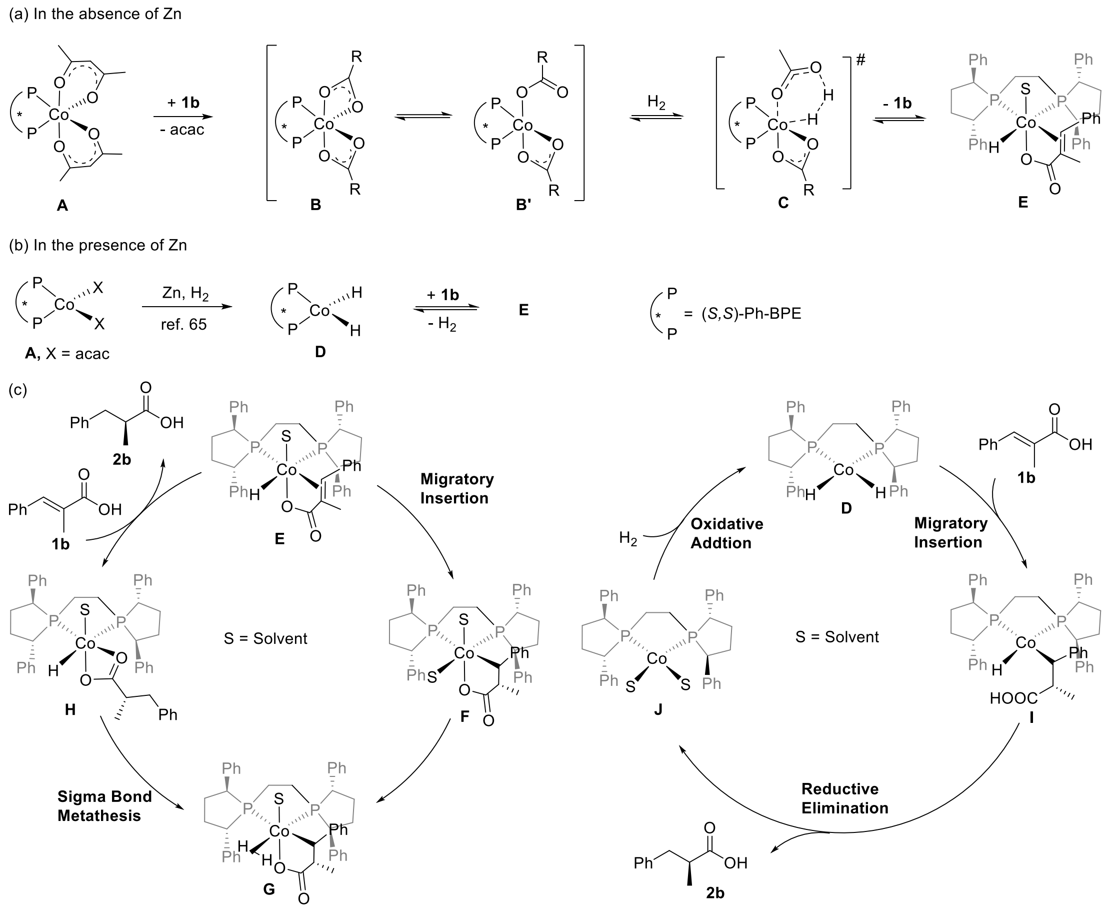

Supplementary Figure 14. Plausible mechanism. (a): the activation of pre-catalyst in the absence of Zn; (b) the activation of pre-catalyst in the presence of Zn; (c) plausible catalytic cycle.

Supplementary Figures

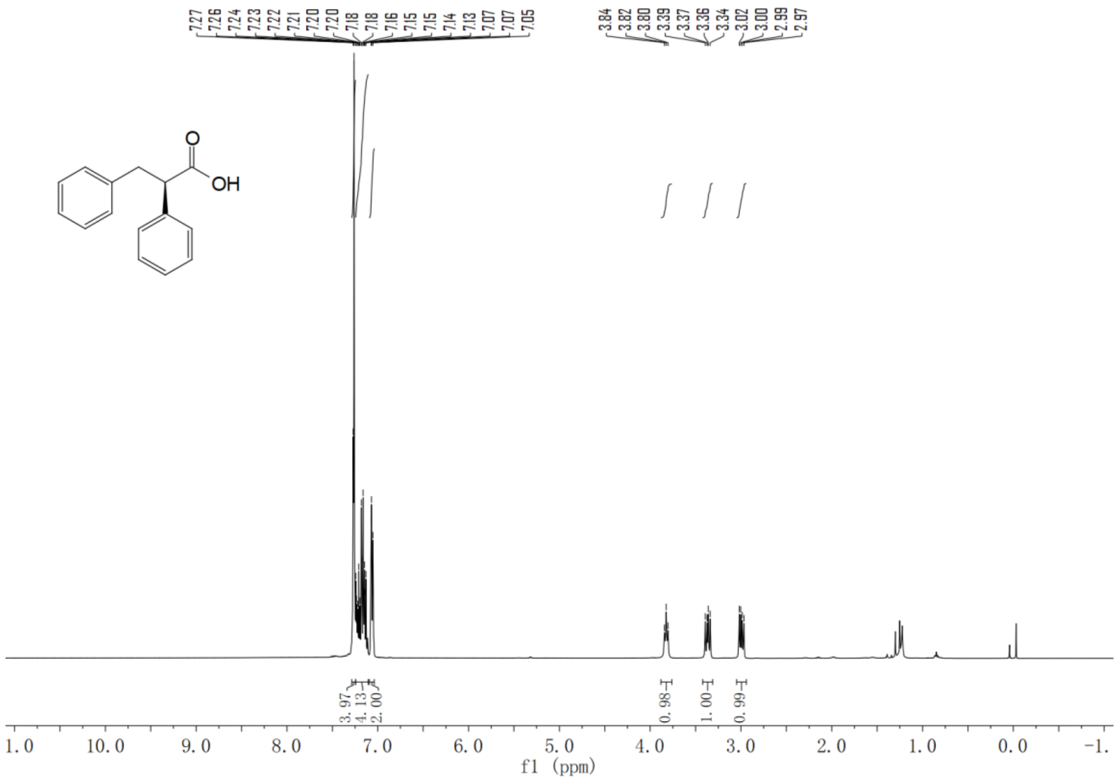

Supplementary Figure 15. ^{1}H NMR (400 MHz, $CDCl_3$) spectrum of **2a**

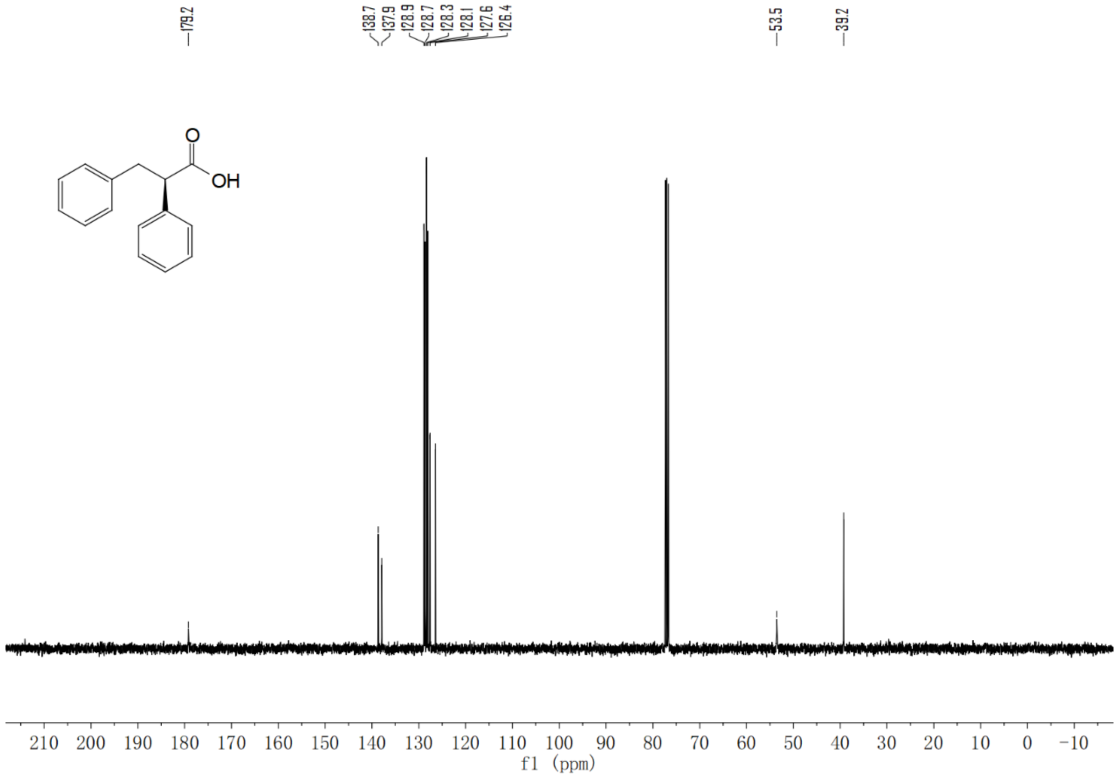

Supplementary Figure 16. ^{13}C NMR (101 MHz, $CDCl_3$) spectrum of **2a**

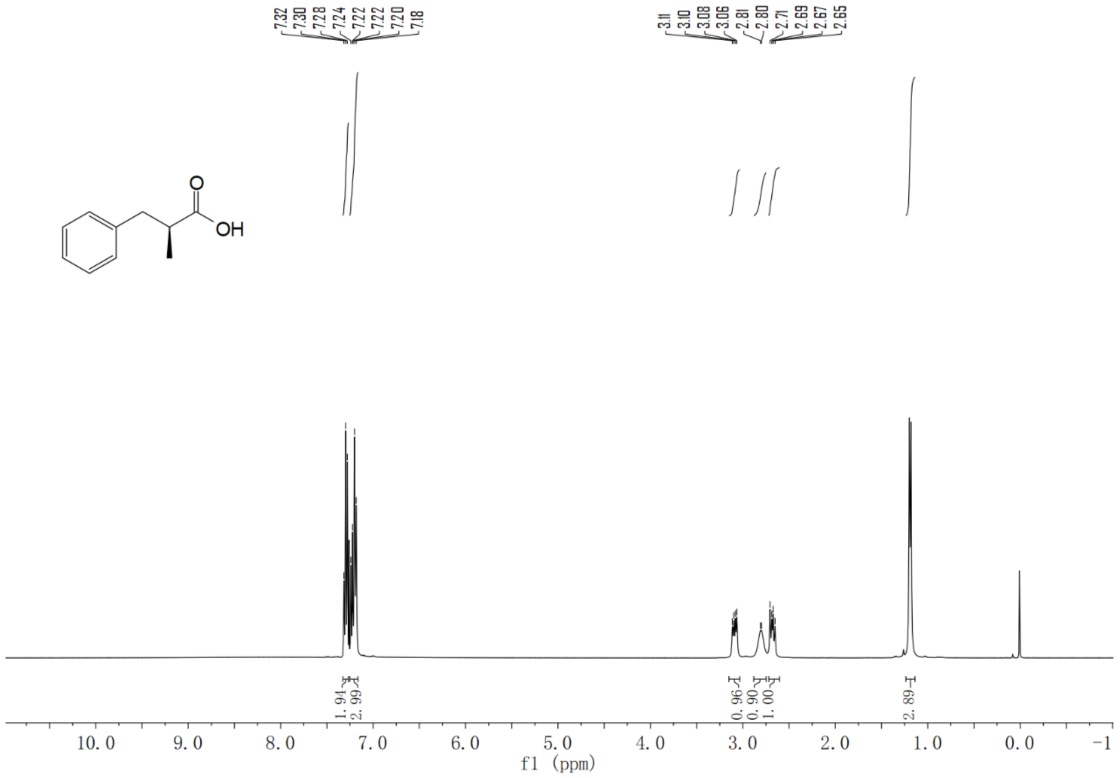

Supplementary Figure 17. ^{1}H NMR (400 MHz, $CDCl_3$) spectrum of **2b**

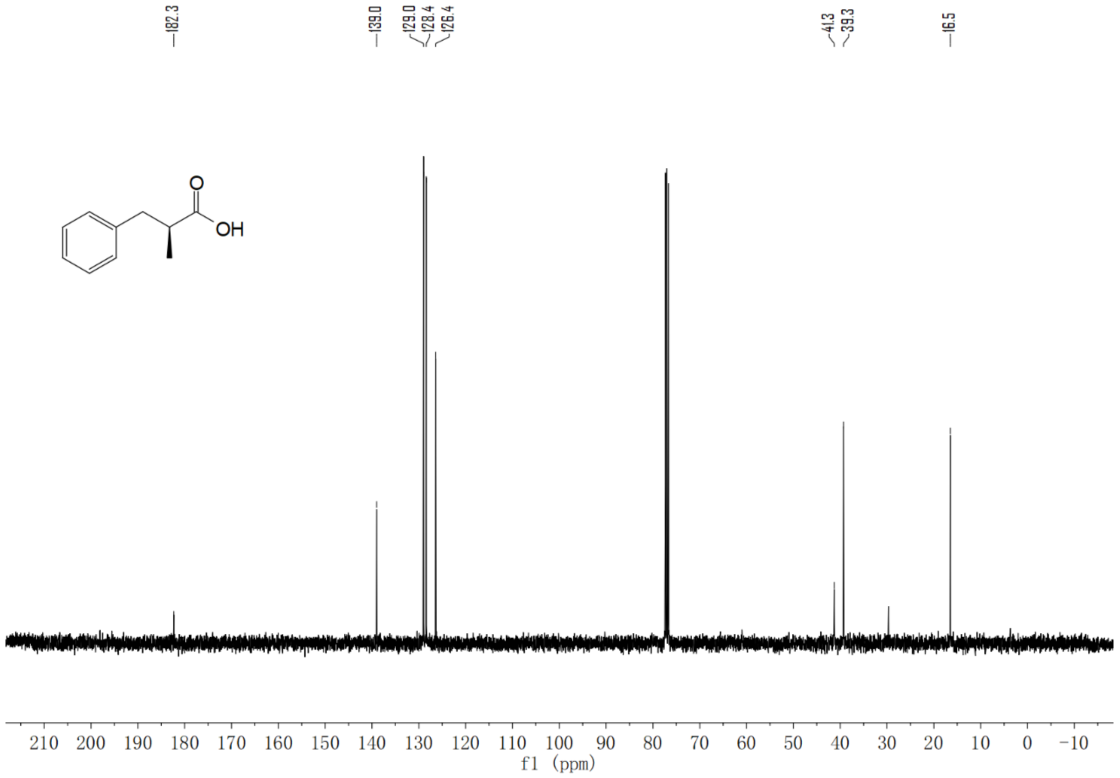

Supplementary Figure 18. ^{13}C NMR (101 MHz, $CDCl_3$) spectrum of **2b**

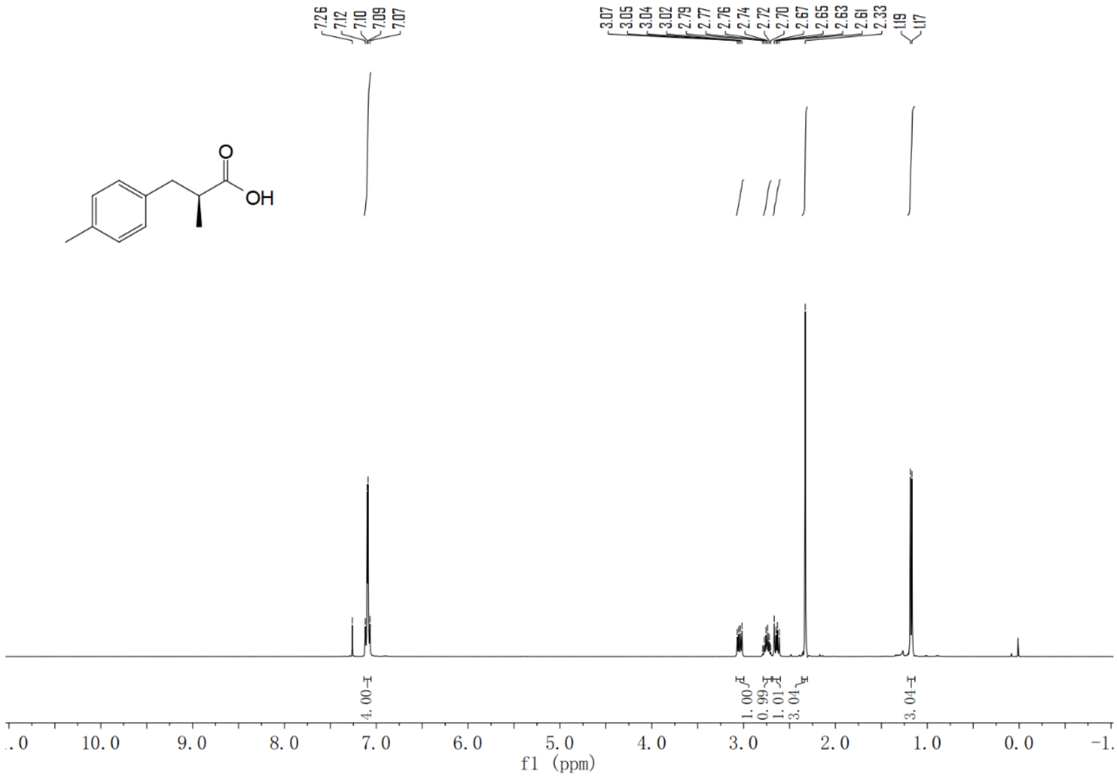

Supplementary Figure 19. ^{1}H NMR (400 MHz, $CDCl_3$) spectrum of **2c**

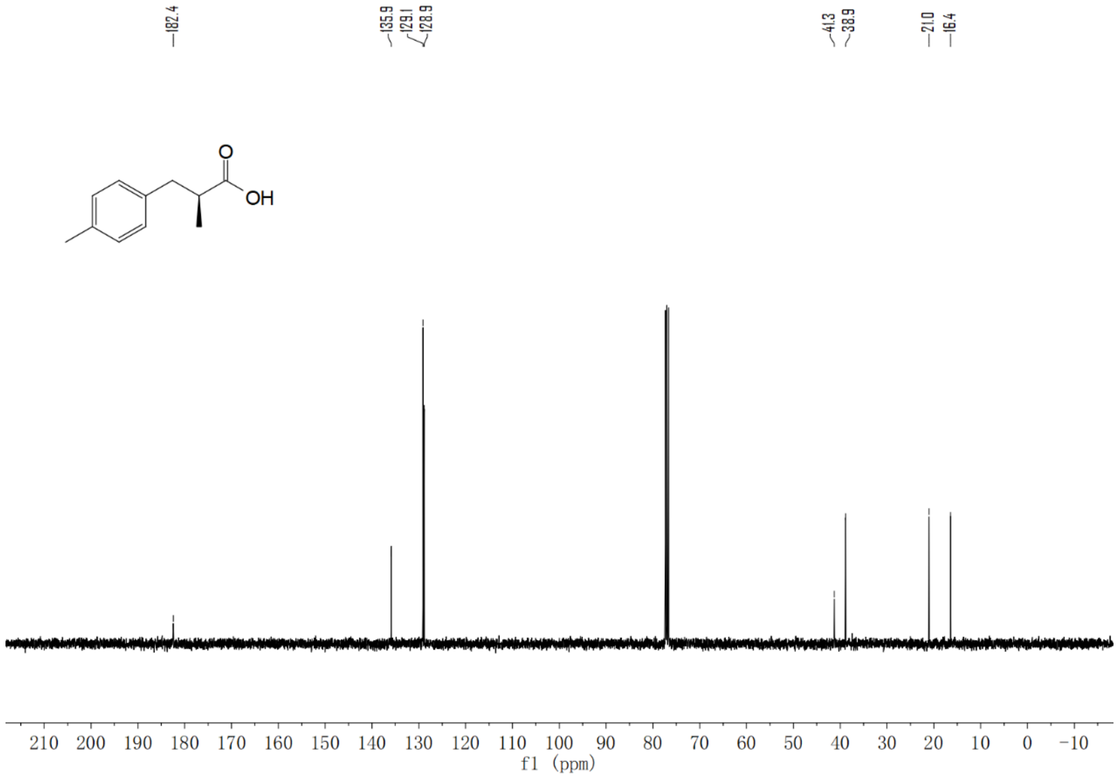

Supplementary Figure 20. ^{13}C NMR (101 MHz, $CDCl_3$) spectrum of **2c**

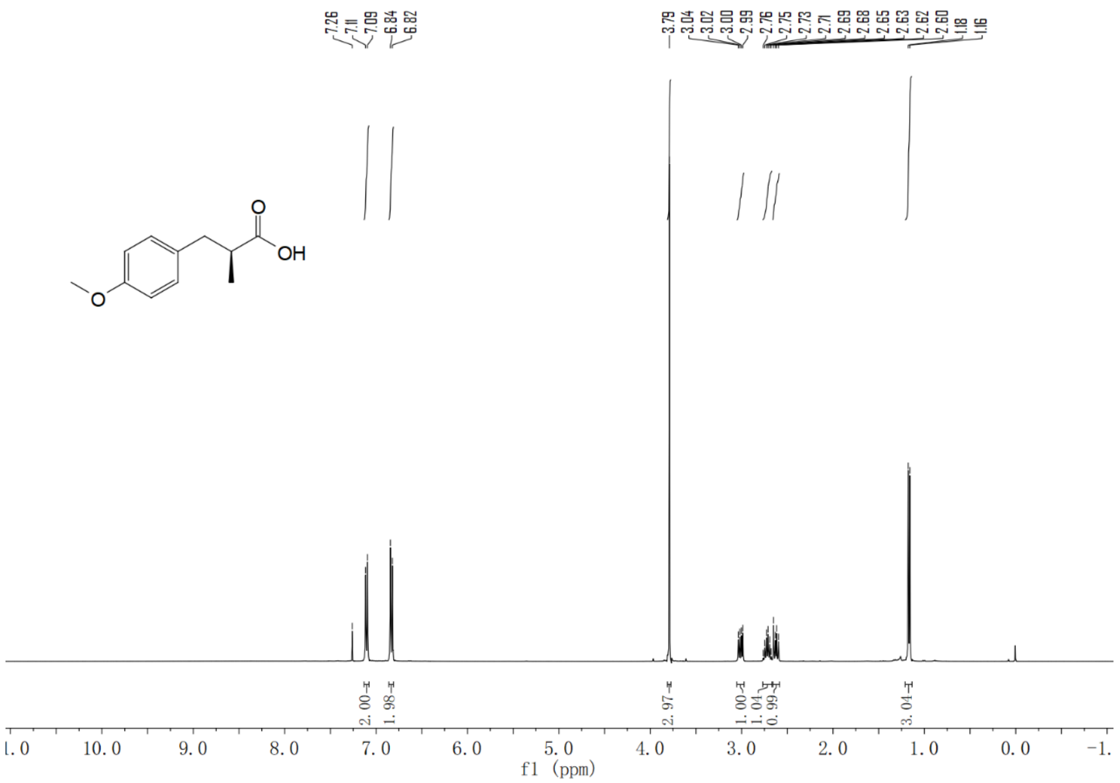

Supplementary Figure 21. ^{1}H NMR (400 MHz, $CDCl_3$) spectrum of **2d**

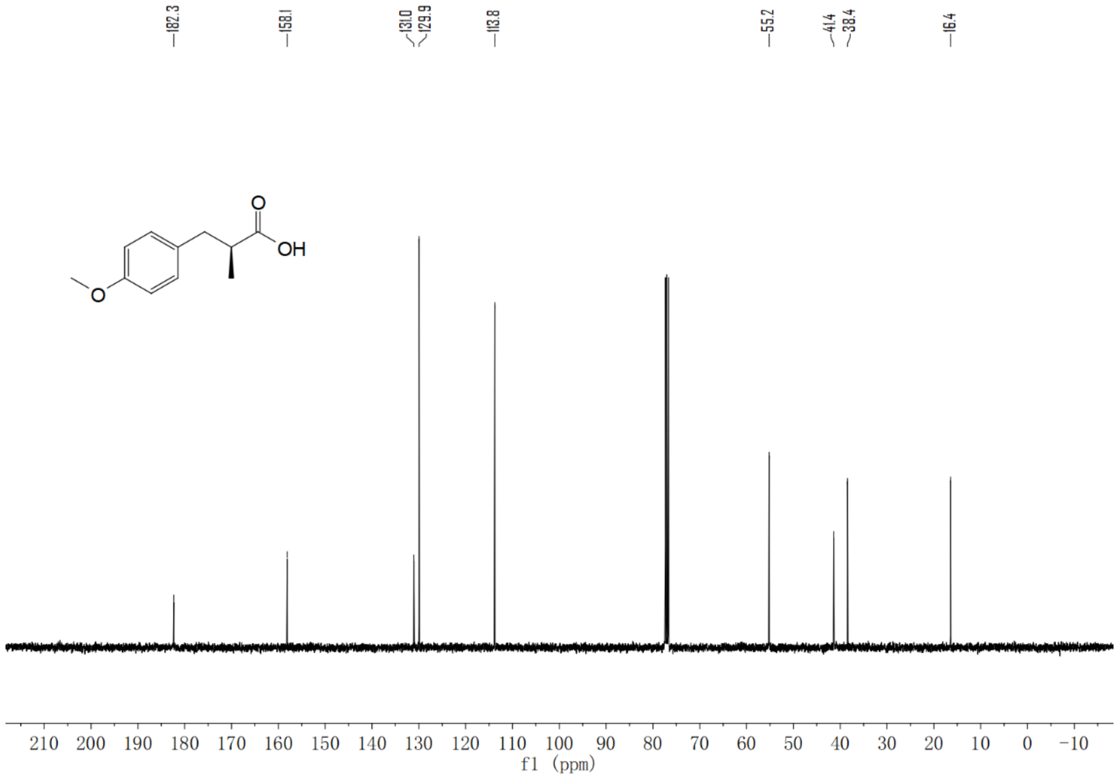

Supplementary Figure 22. ^{13}C NMR (101 MHz, $CDCl_3$) spectrum of **2d**

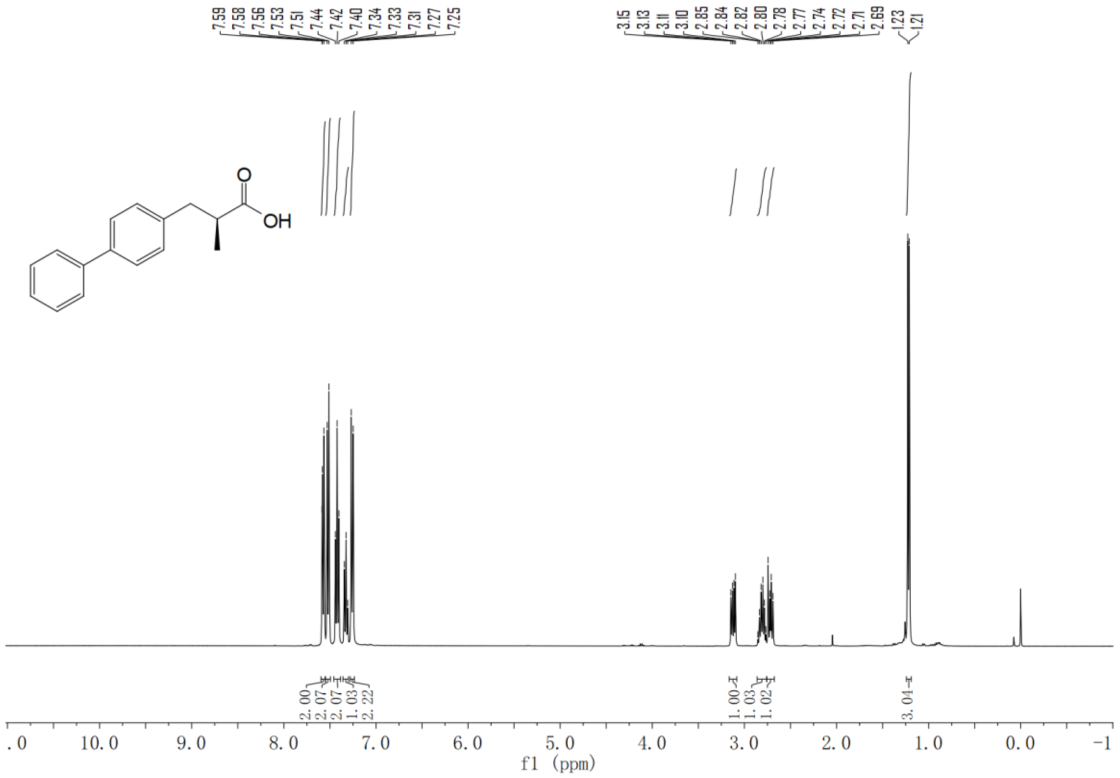

Supplementary Figure 23. ^{1}H NMR (400 MHz, $CDCl_3$) spectrum of **2e**

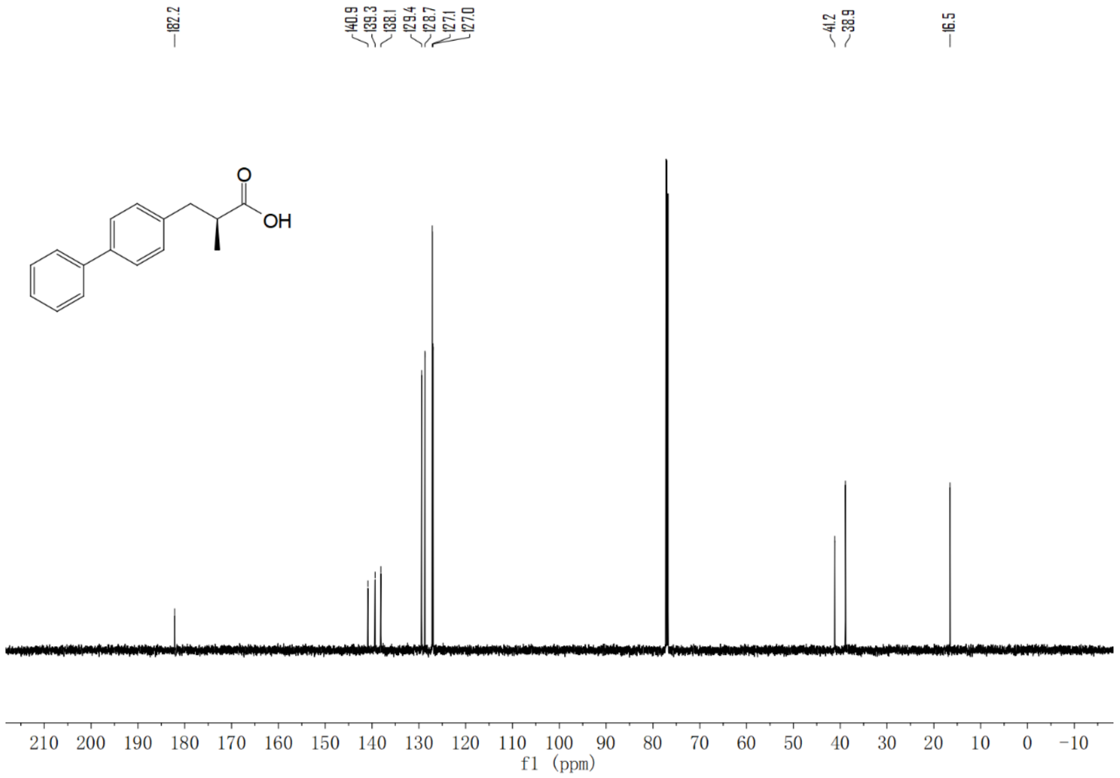

Supplementary Figure 24. ^{13}C NMR (151 MHz, $CDCl_3$) spectrum of **2e**

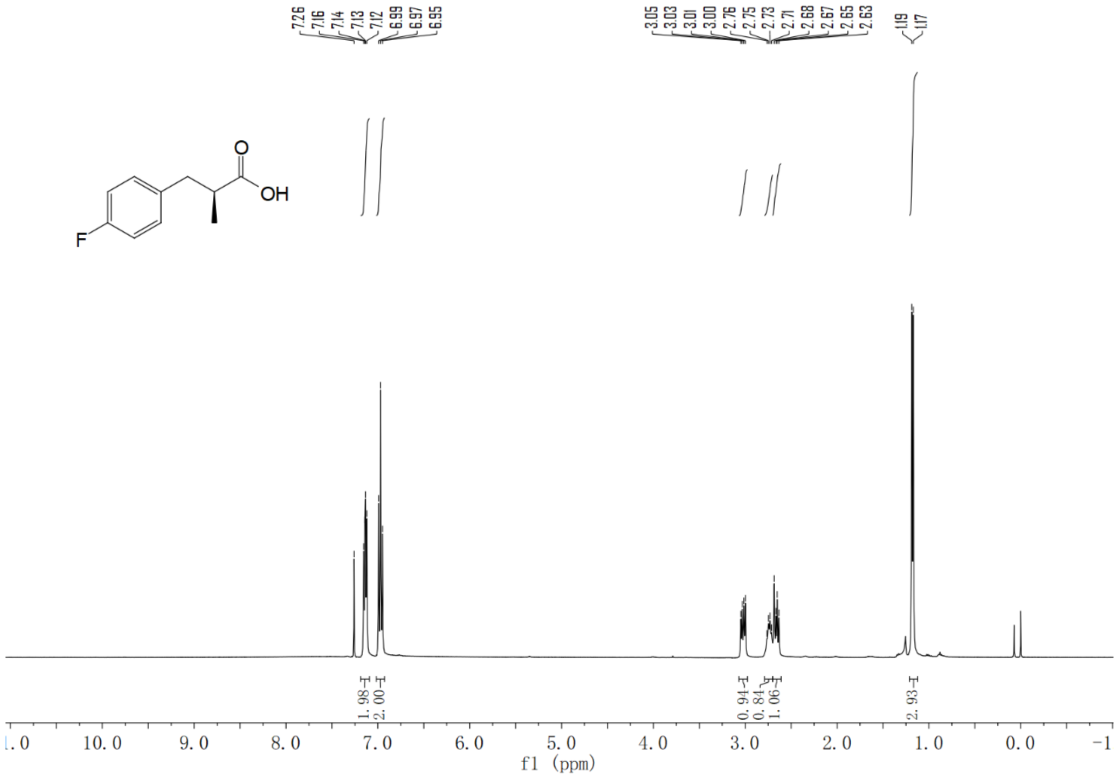

Supplementary Figure 25. ^{1}H NMR (400 MHz, $CDCl_3$) spectrum of **2f**

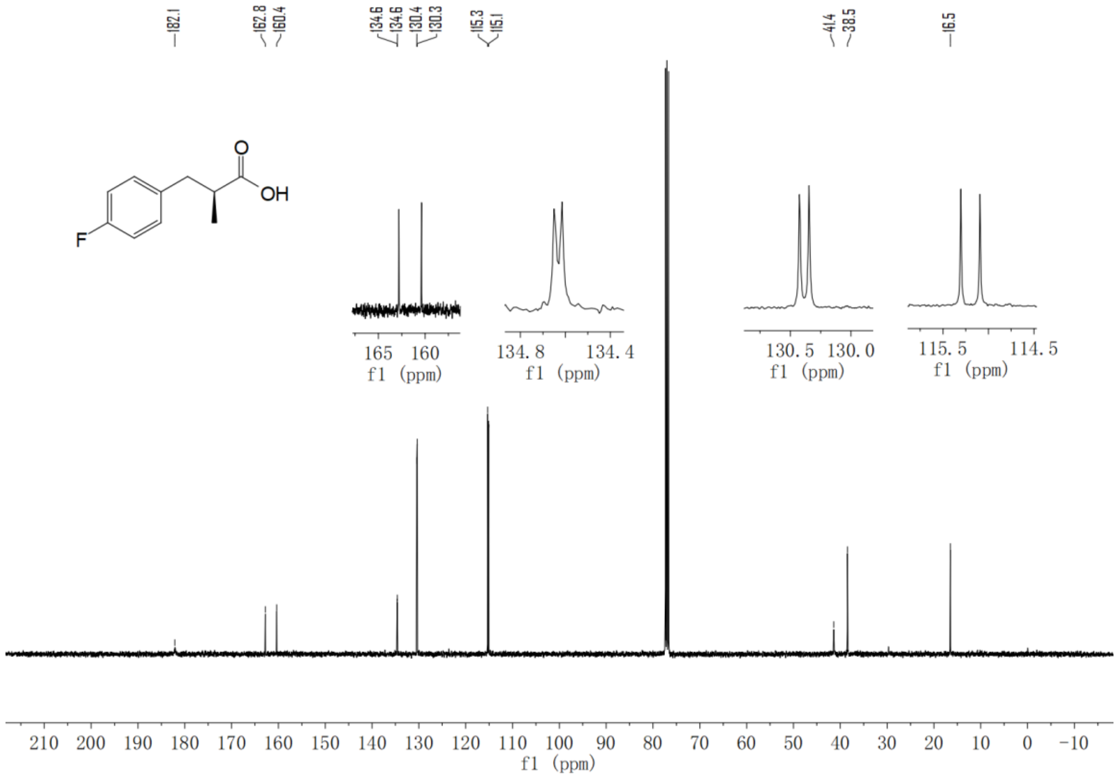

Supplementary Figure 26. ^{13}C NMR (101 MHz, $CDCl_3$) spectrum of **2f**

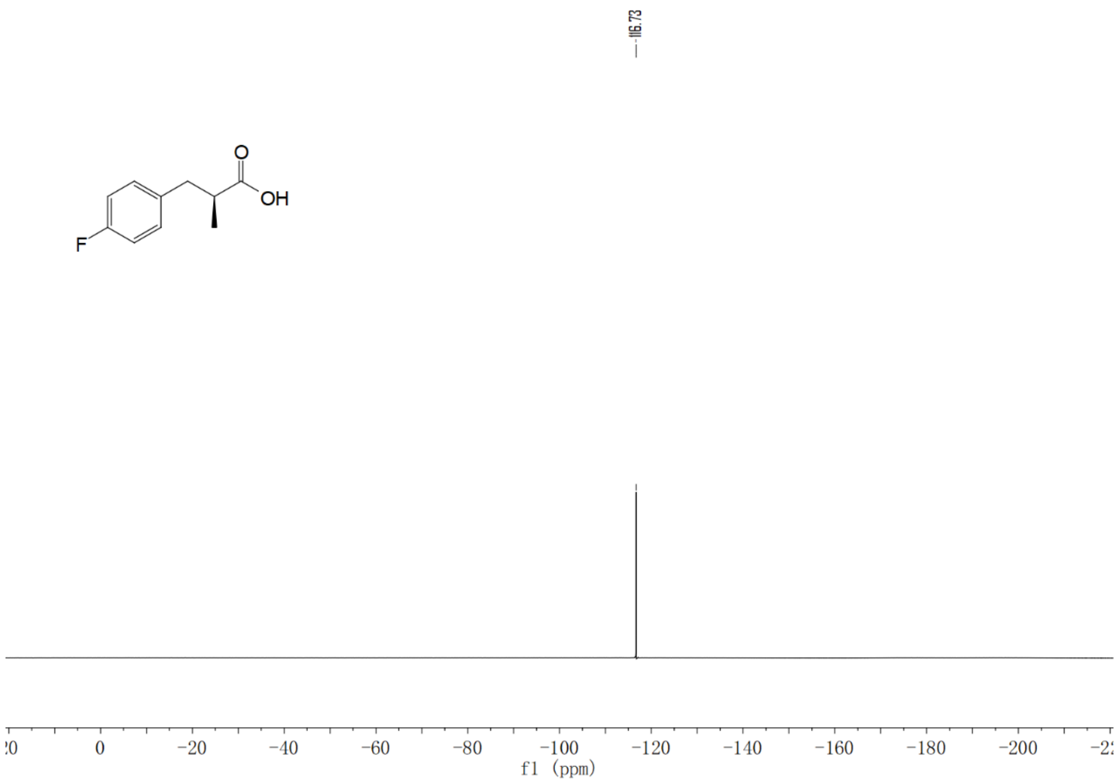

Supplementary Figure 27. ^{19}F NMR (376 MHz, $CDCl_3$) spectrum of **2f**

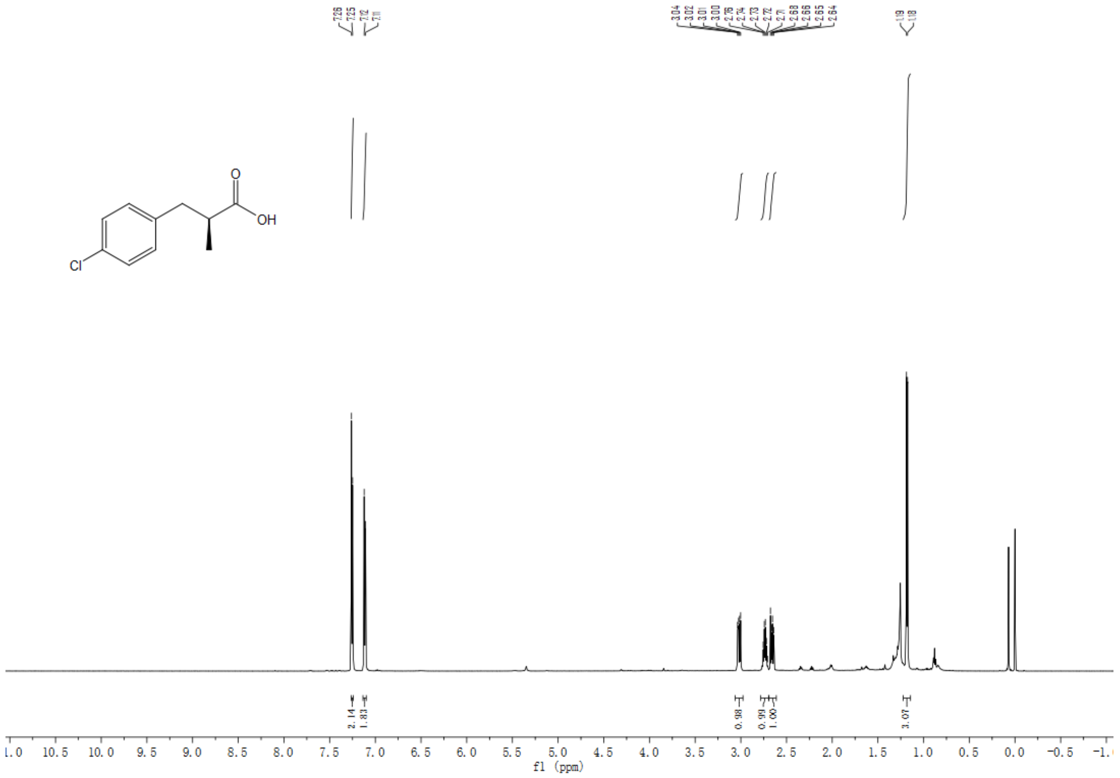

Supplementary Figure 28. ^{1}H NMR (600 MHz, $CDCl_3$) spectrum of **2g**

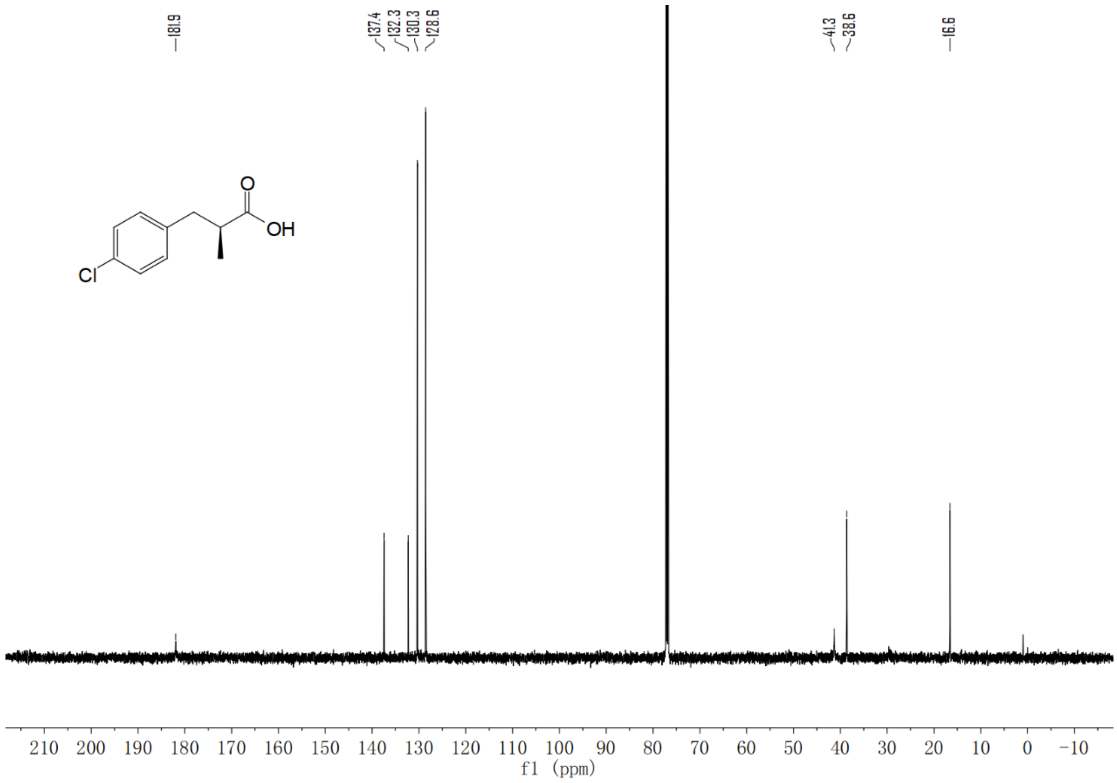

Supplementary Figure 29. ^{13}C NMR (151 MHz, $CDCl_3$) spectrum of **2g**

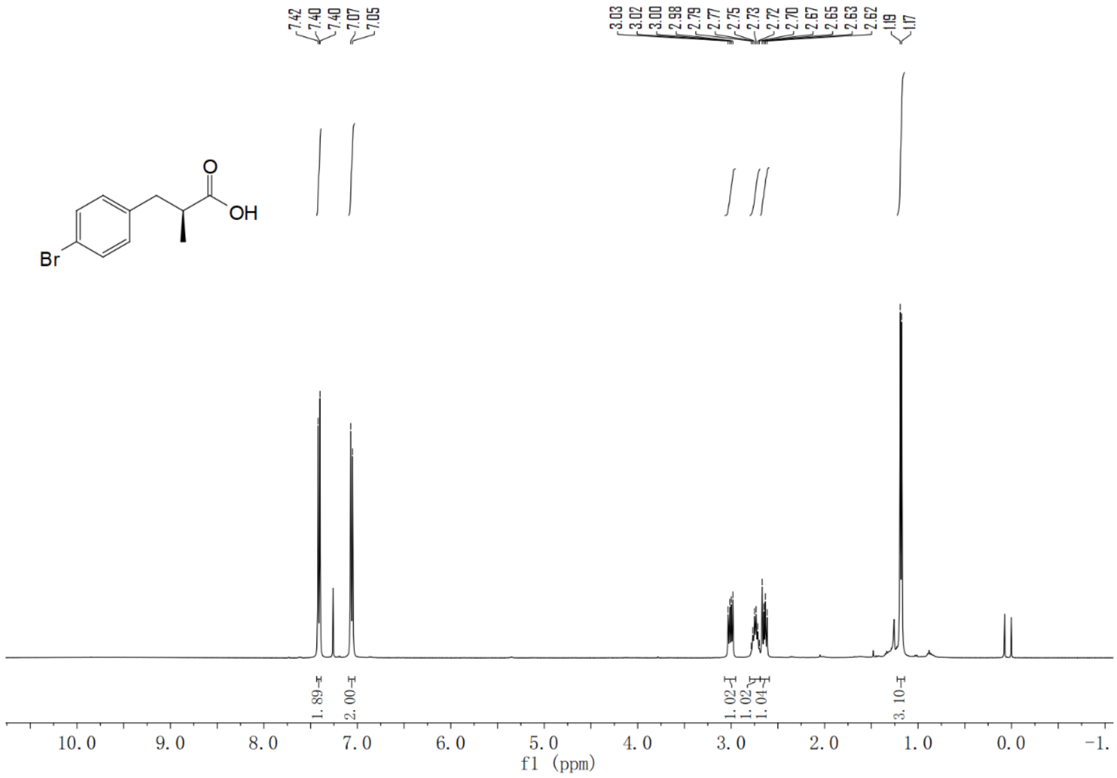

Supplementary Figure 30. ^{1}H NMR (400 MHz, $CDCl_3$) spectrum of **2h**

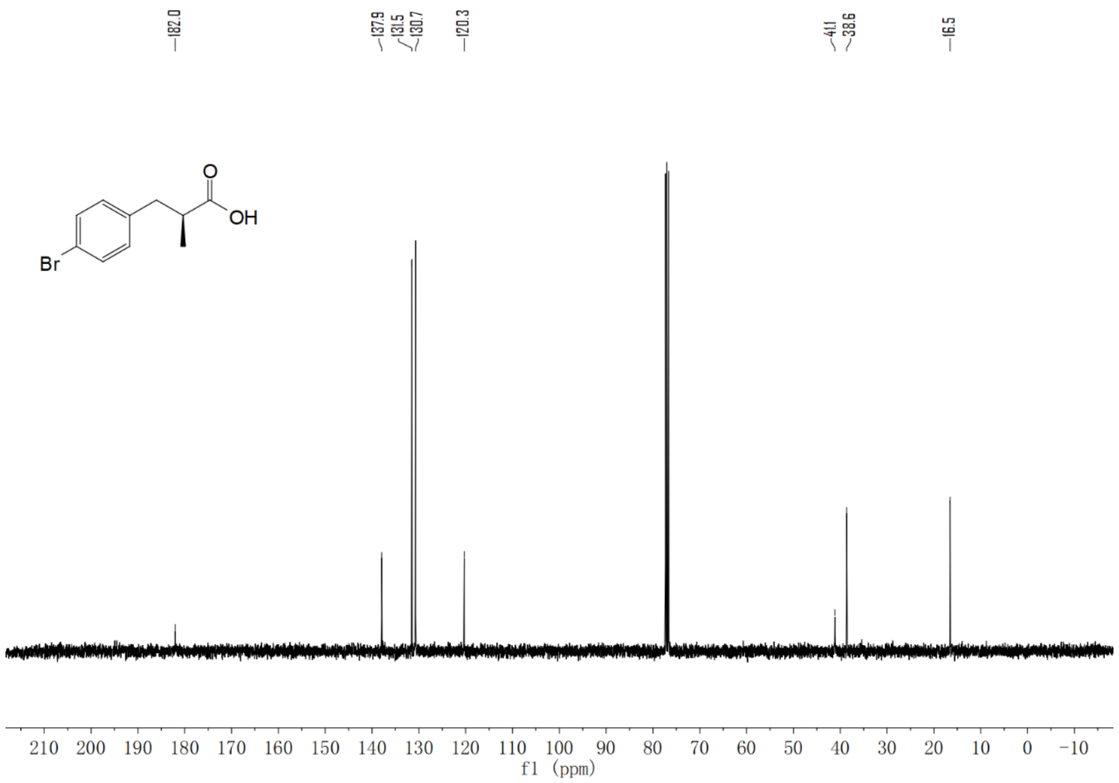

Supplementary Figure 31. ^{13}C NMR (101 MHz, $CDCl_3$) spectrum of **2h**

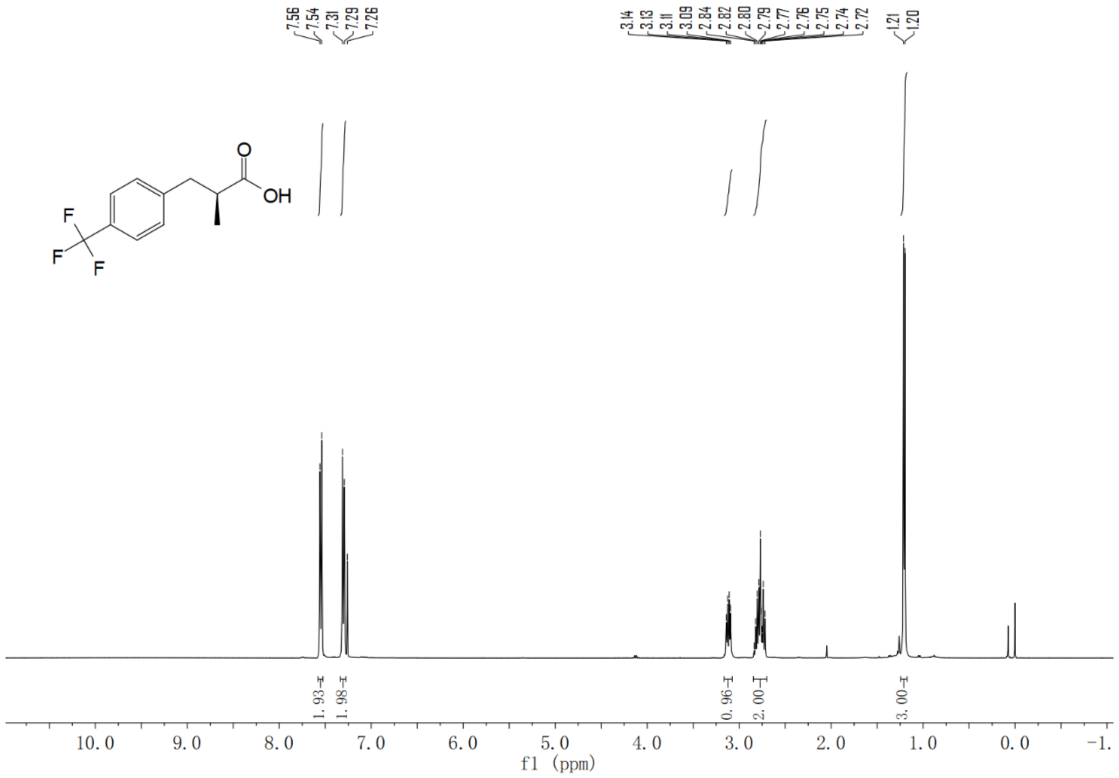

Supplementary Figure 32. ^{1}H NMR (400 MHz, $CDCl_3$) spectrum of **2i**

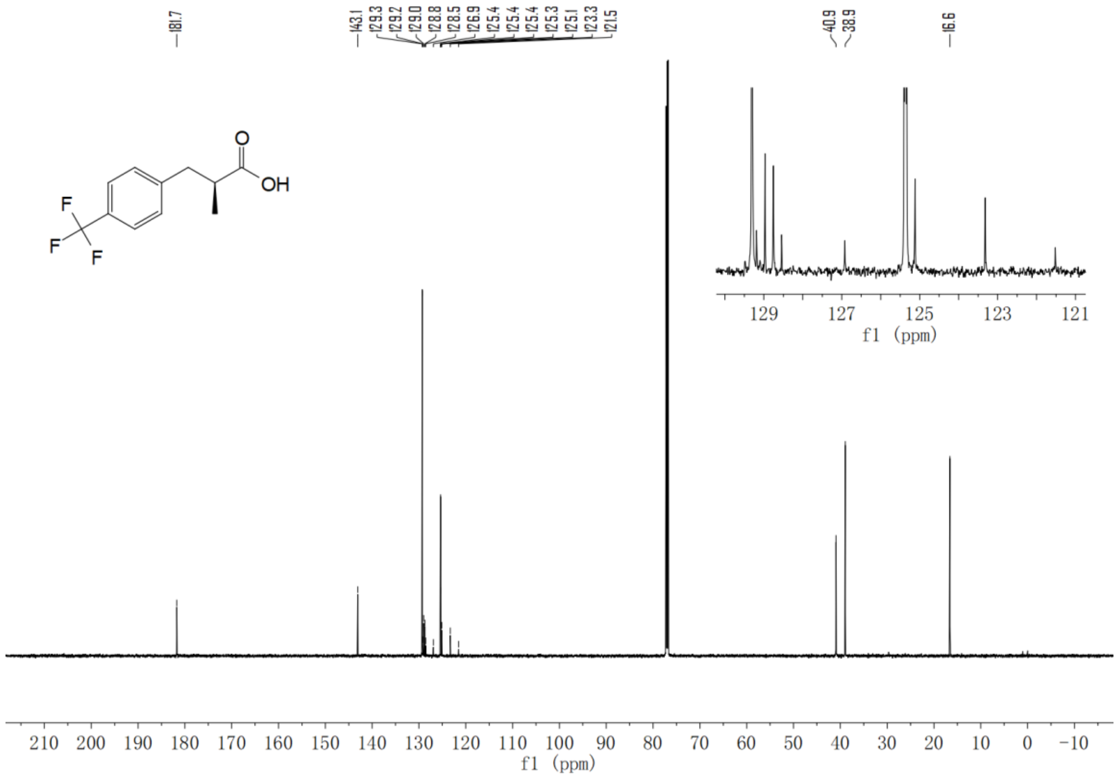

Supplementary Figure 33. ^{13}C NMR (151 MHz, $CDCl_3$) spectrum of **2i**

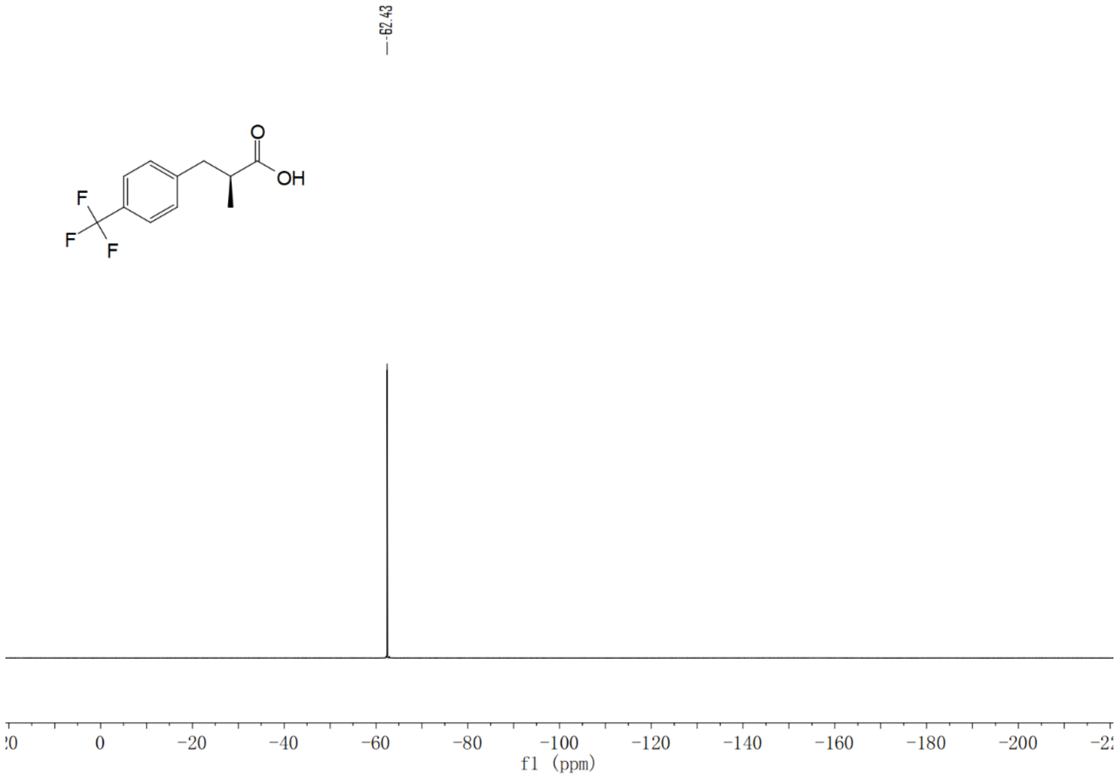

Supplementary Figure 34. ^{19}F NMR (376 MHz, $CDCl_3$) spectrum of **2i**

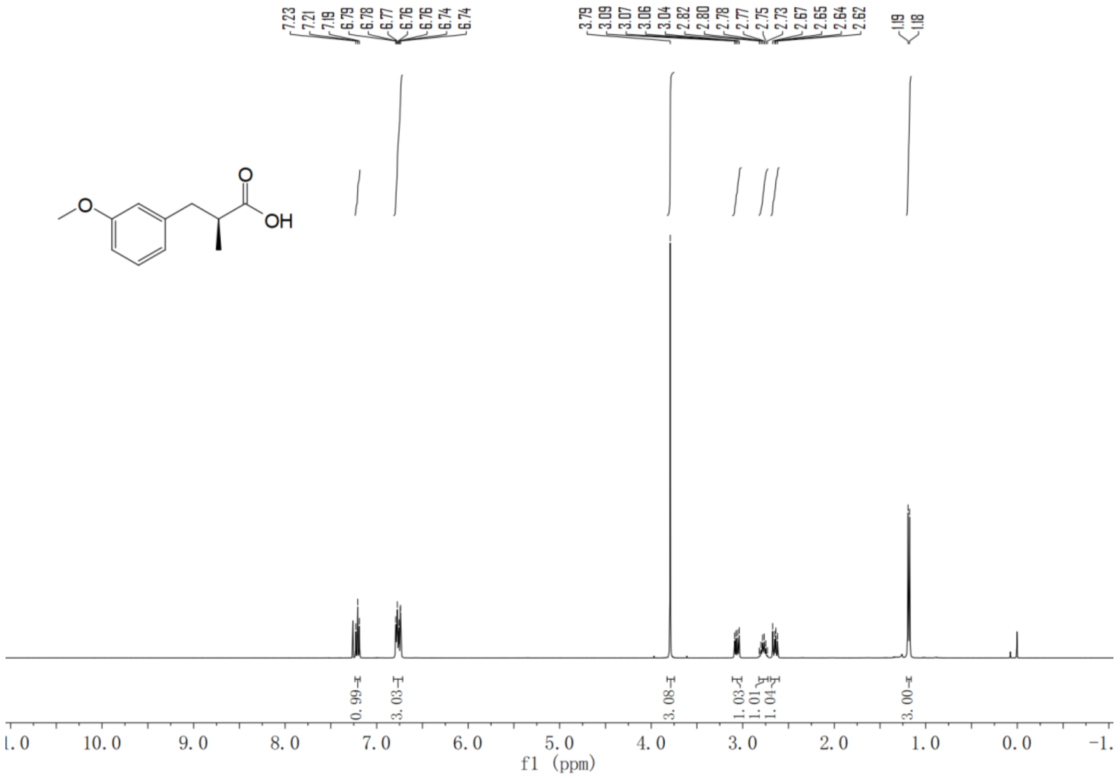

Supplementary Figure 35. ^{1}H NMR (400 MHz, $CDCl_3$) spectrum of **2j**

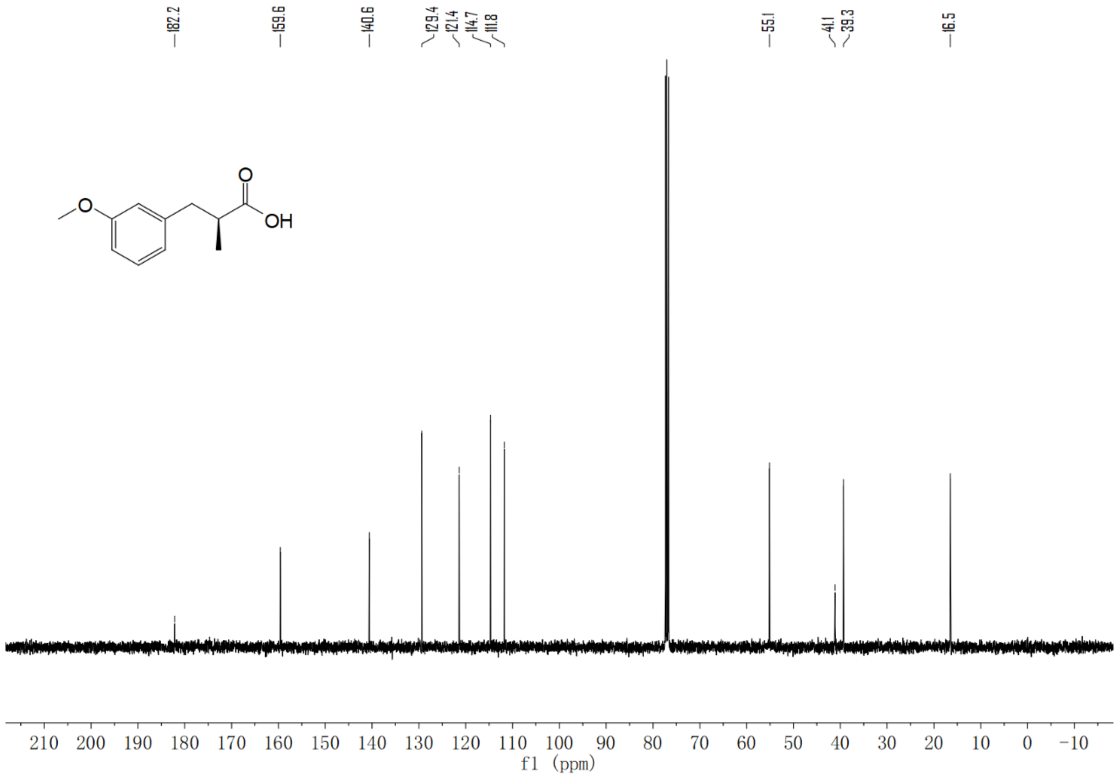

Supplementary Figure 36. ^{13}C NMR (101 MHz, $CDCl_3$) spectrum of **2j**

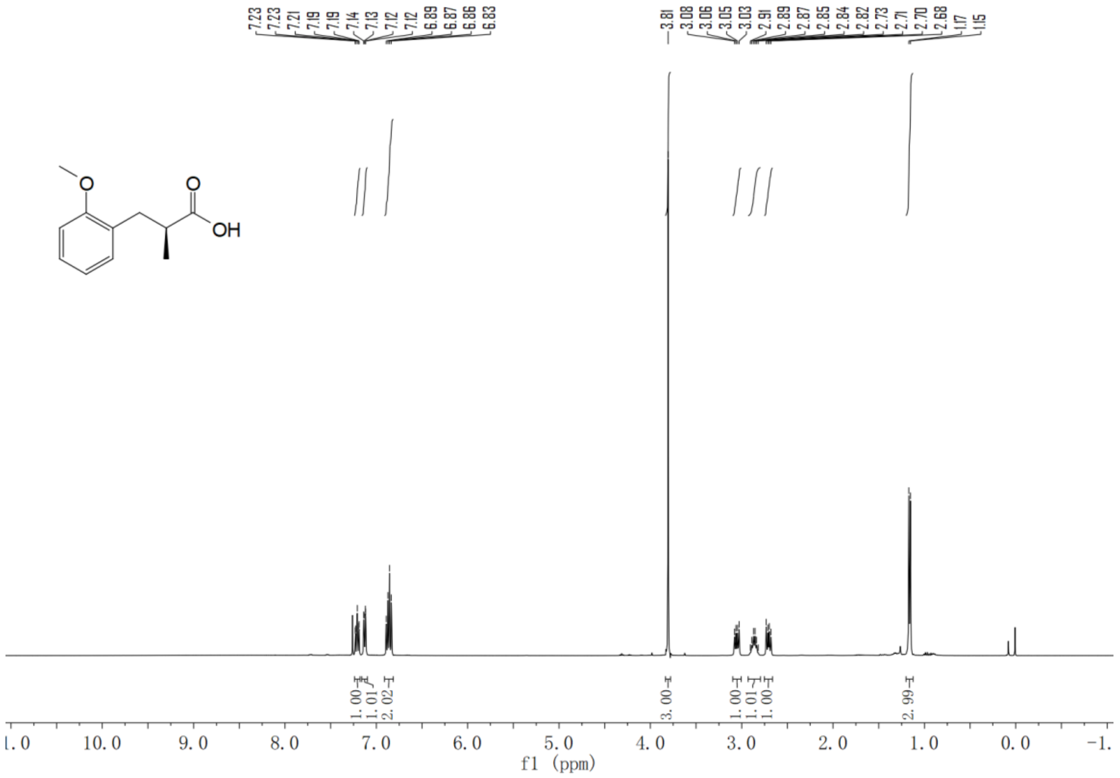

Supplementary Figure 37. ^{1}H NMR (400 MHz, $CDCl_3$) spectrum of **2k**

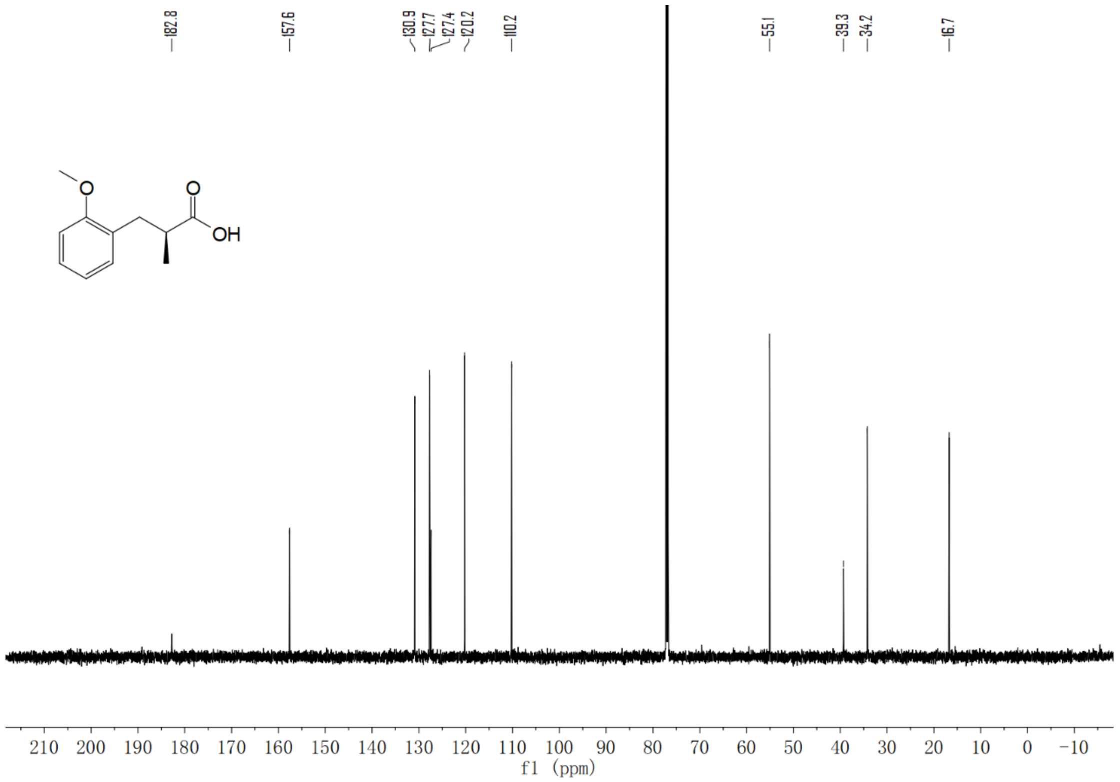

Supplementary Figure 38. ^{13}C NMR (151 MHz, $CDCl_3$) spectrum of **2k**

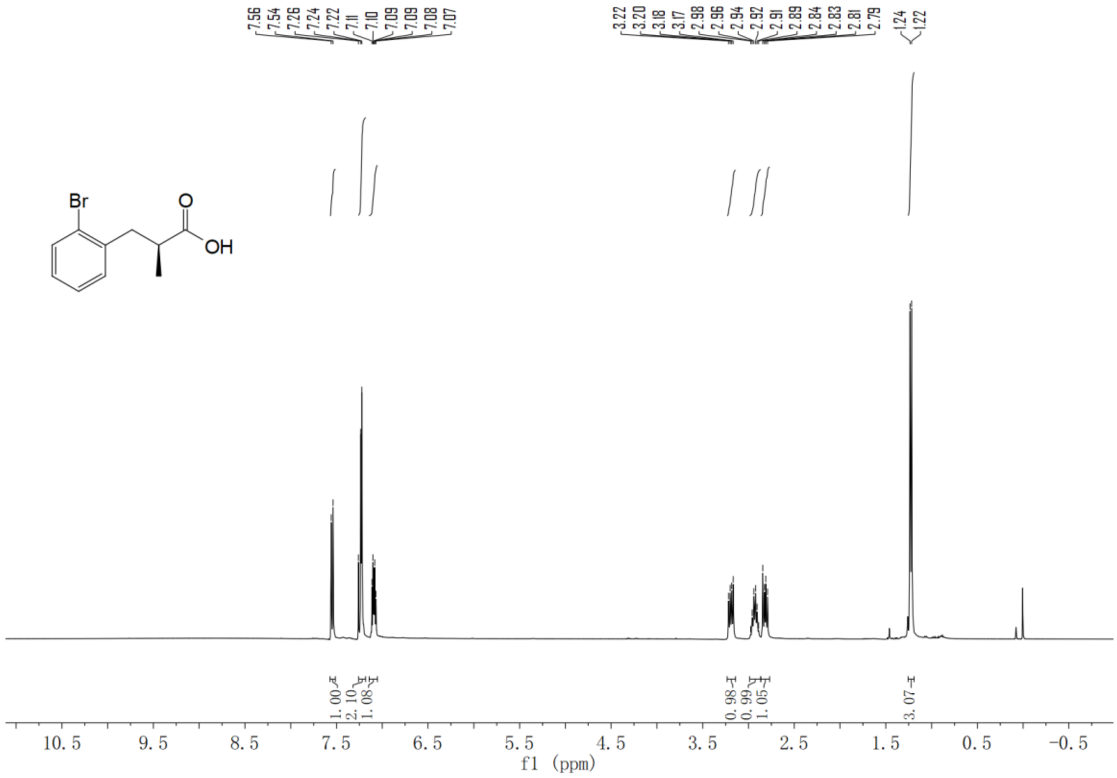

Supplementary Figure 39. ^{1}H NMR (400 MHz, $CDCl_3$) spectrum of **2l**

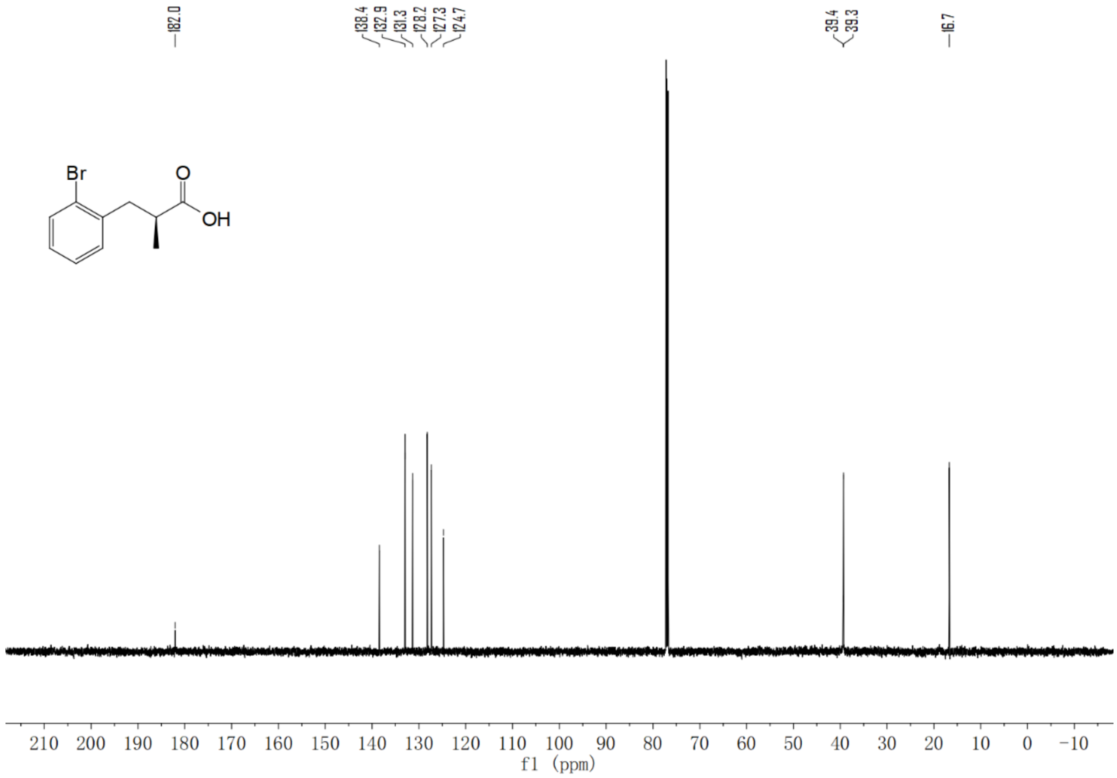

Supplementary Figure 40. ^{13}C NMR (151 MHz, $CDCl_3$) spectrum of **2l**

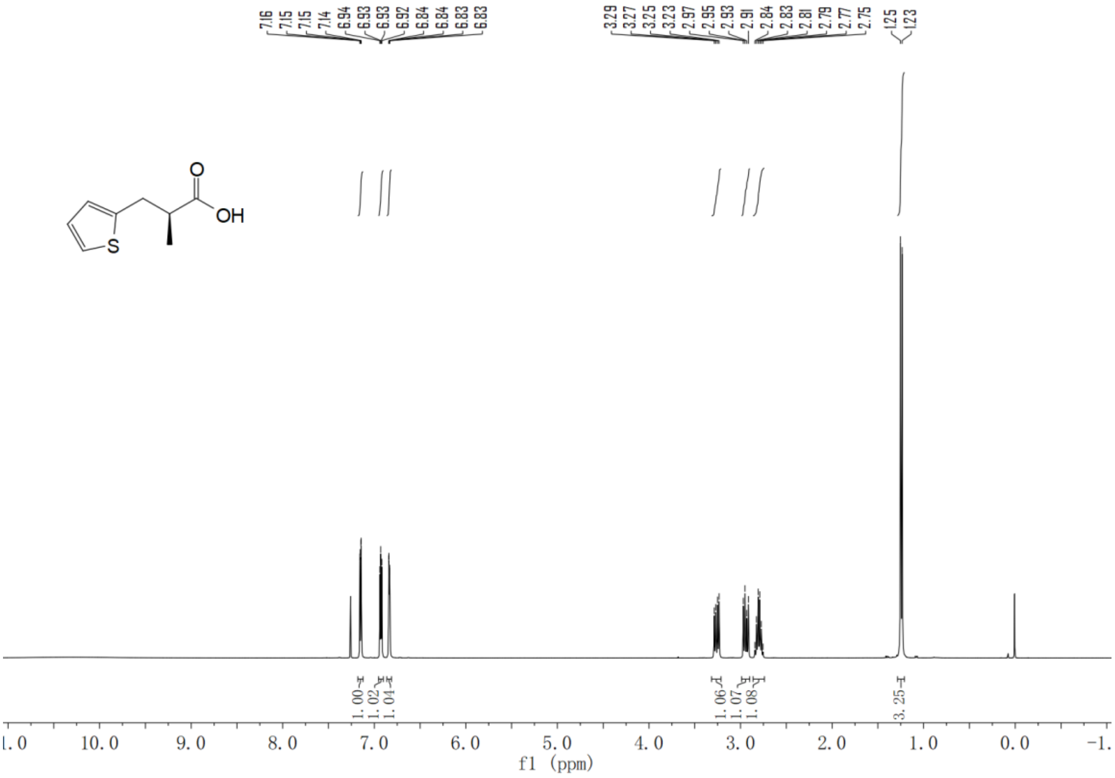

Supplementary Figure 41. ^{1}H NMR (400 MHz, $CDCl_3$) spectrum of **2m**

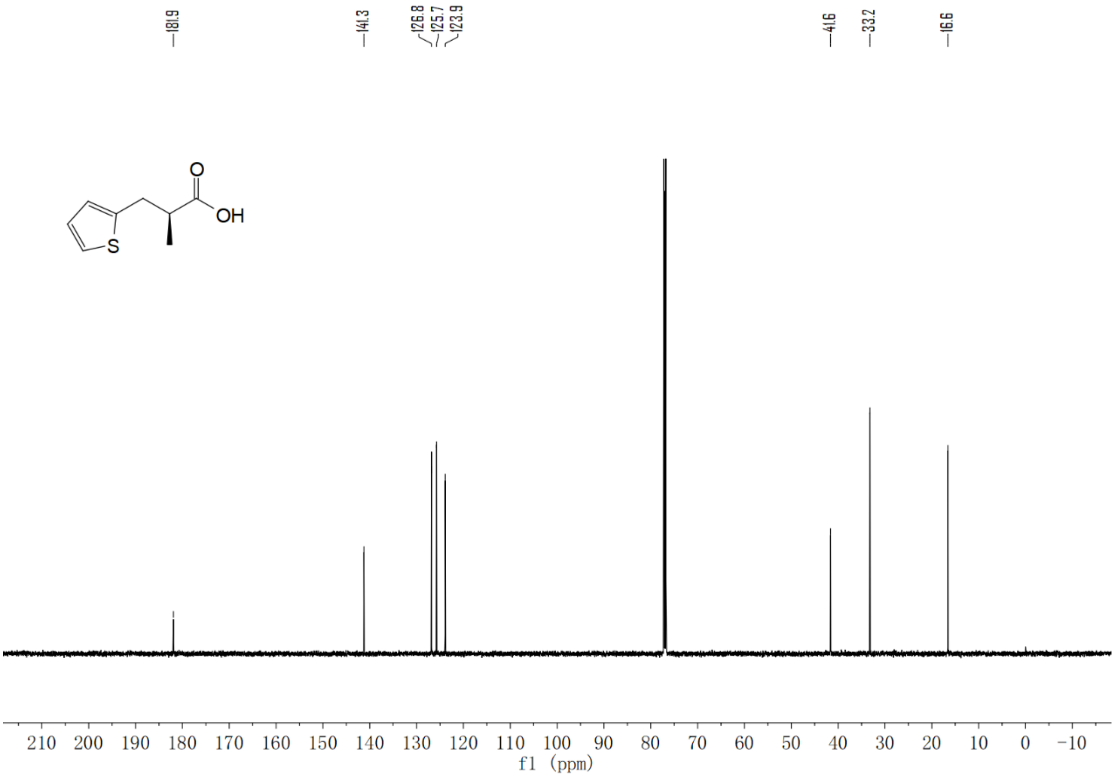

Supplementary Figure 42. ^{13}C NMR (151 MHz, $CDCl_3$) spectrum of **2m**

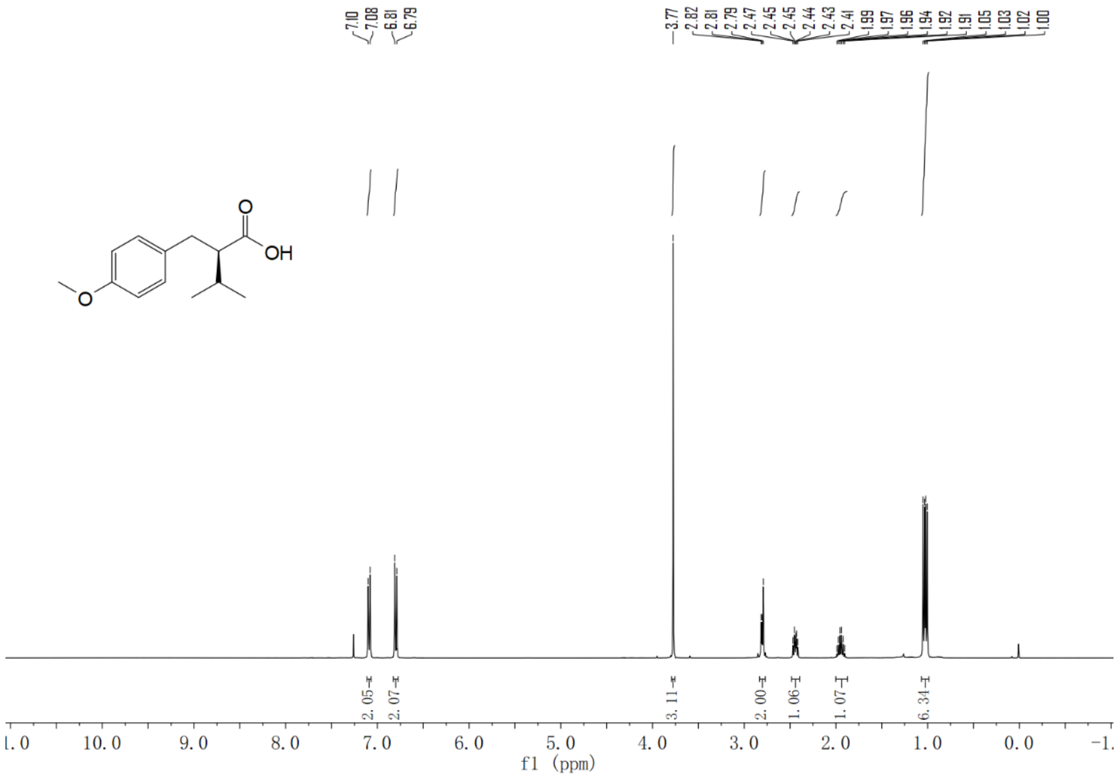

Supplementary Figure 43. ^{1}H NMR (400 MHz, $CDCl_3$) spectrum of **2n**

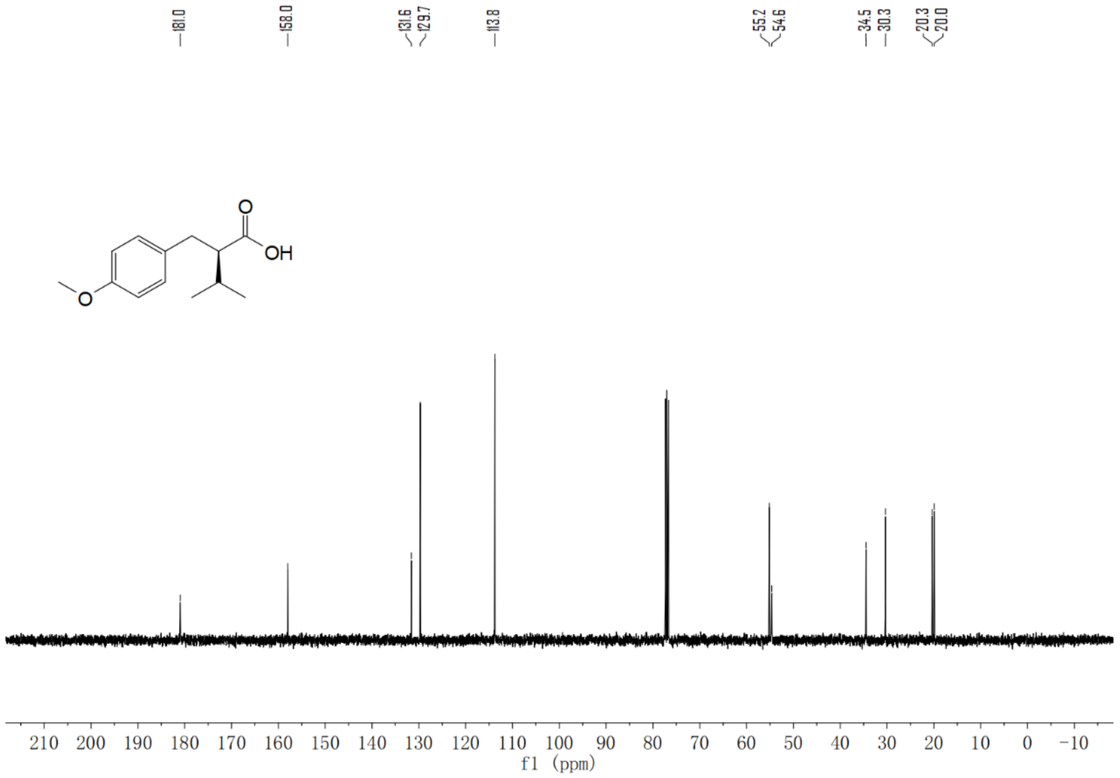

Supplementary Figure 44. ^{13}C NMR (101 MHz, $CDCl_3$) spectrum of **2n**

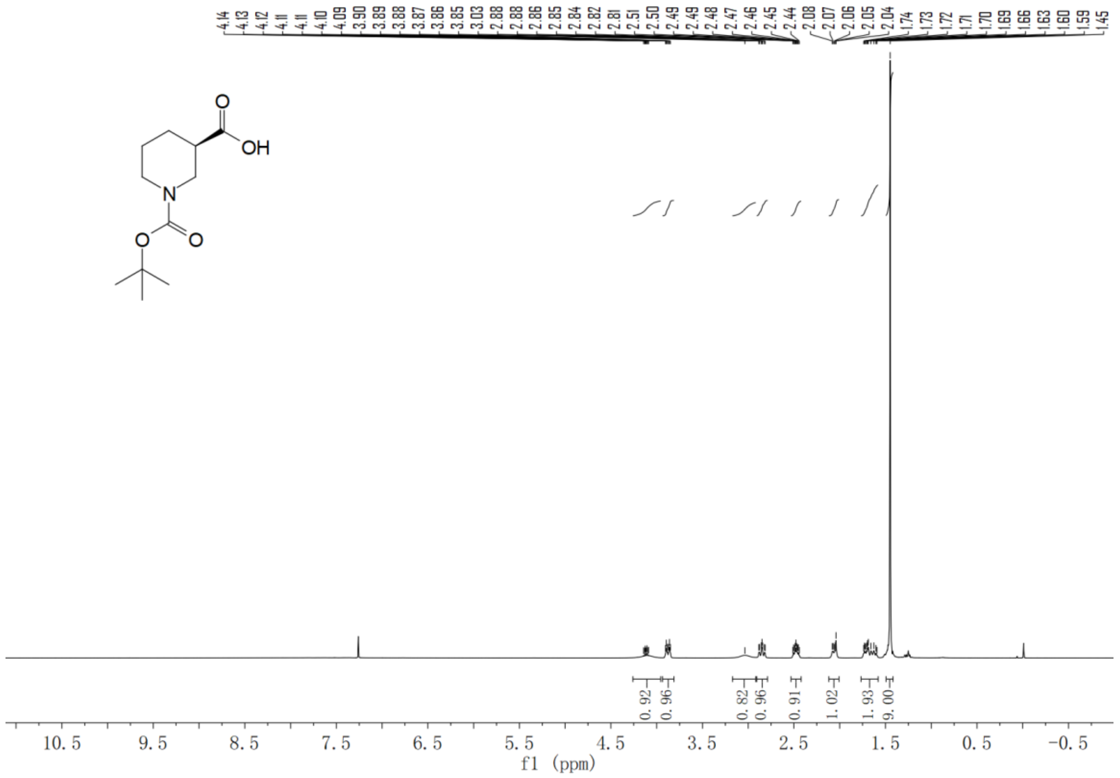

Supplementary Figure 45. ^{1}H NMR (400 MHz, $CDCl_3$) spectrum of **2o**

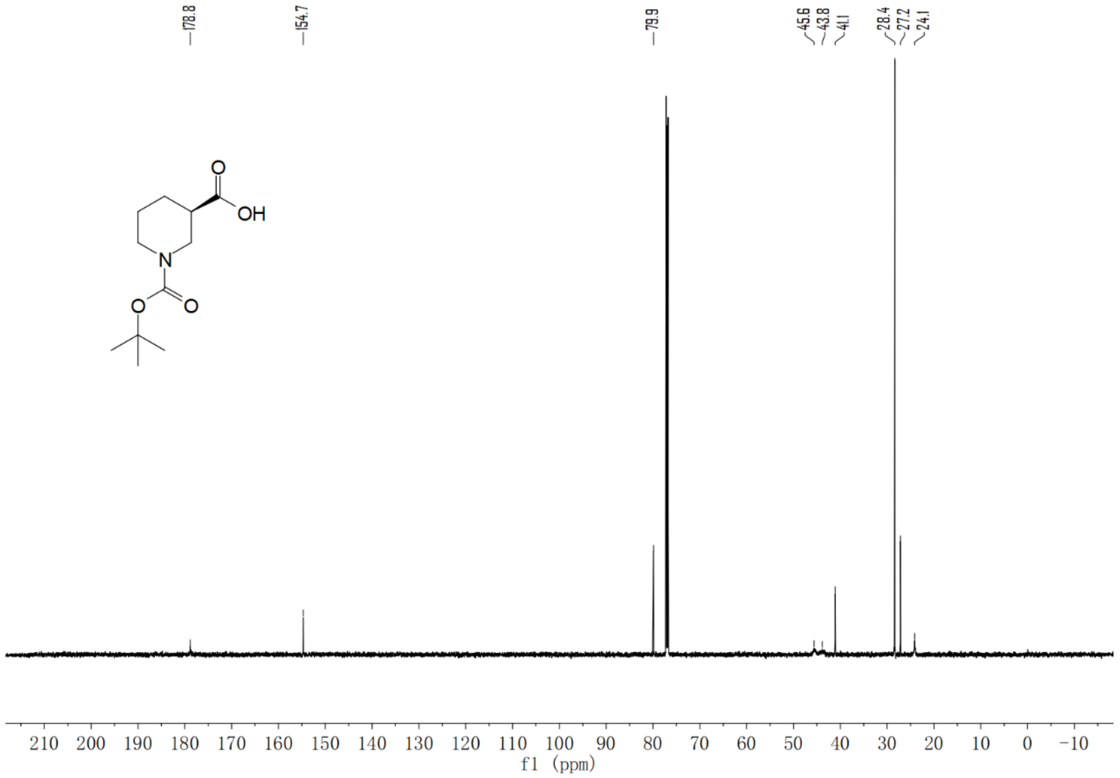

Supplementary Figure 46. ^{13}C NMR (151 MHz, $CDCl_3$) spectrum of **2o**

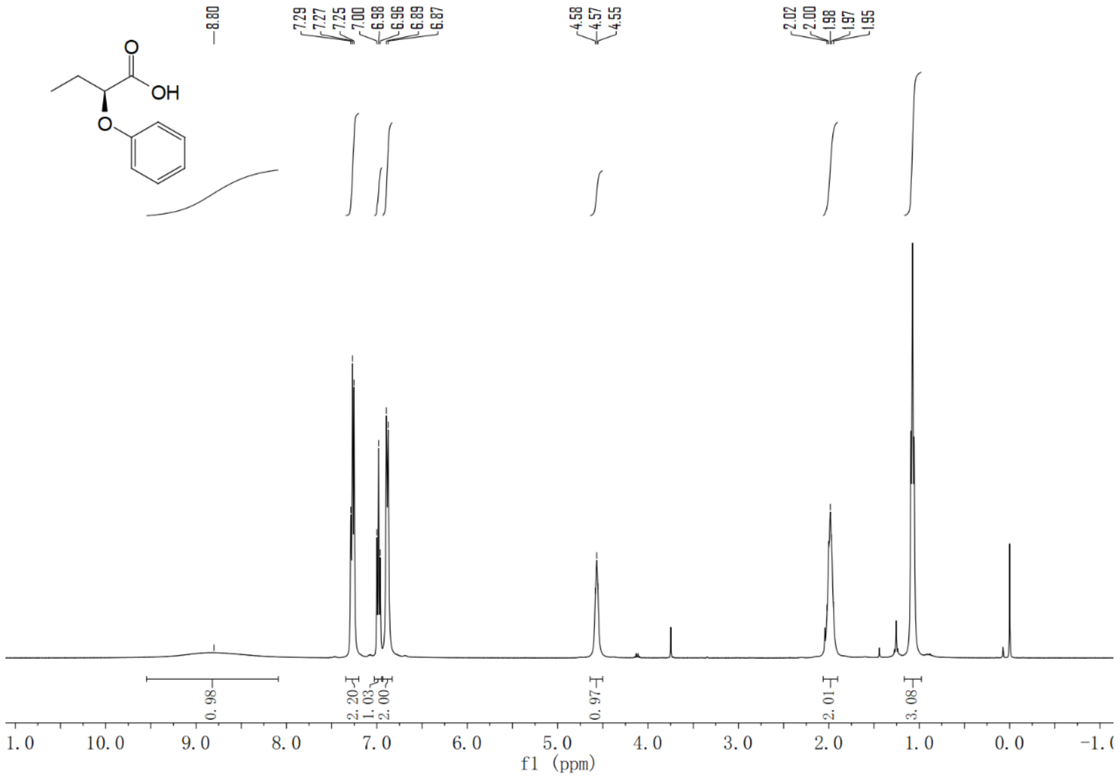

Supplementary Figure 47. ^{1}H NMR (400 MHz, $CDCl_3$) spectrum of **2p**

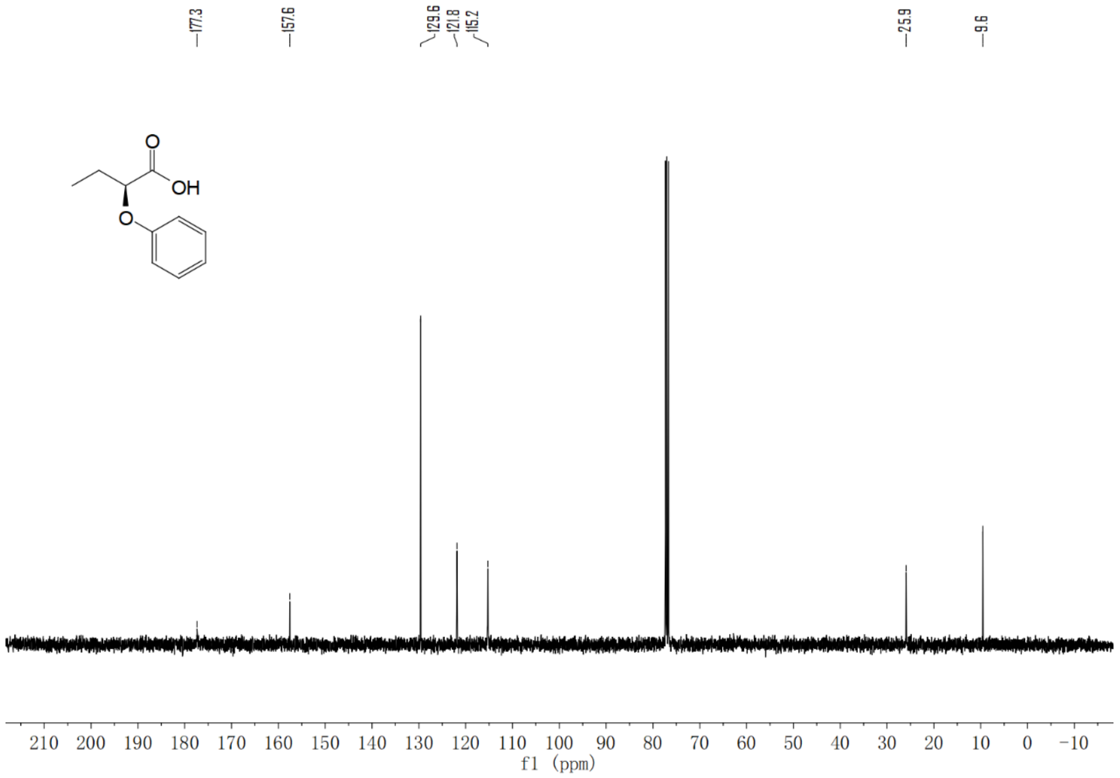

Supplementary Figure 48. ^{13}C NMR (101 MHz, $CDCl_3$) spectrum of **2p**

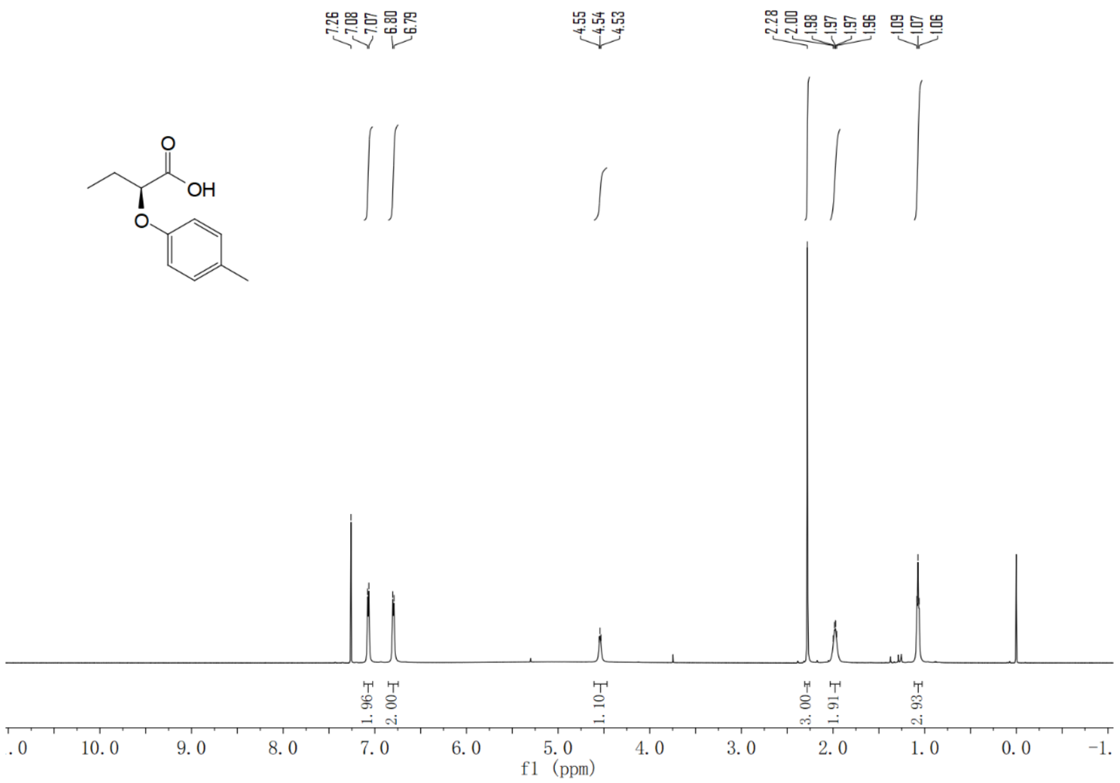

Supplementary Figure 49. ^{1}H NMR (600 MHz, $CDCl_3$) spectrum of **2q**

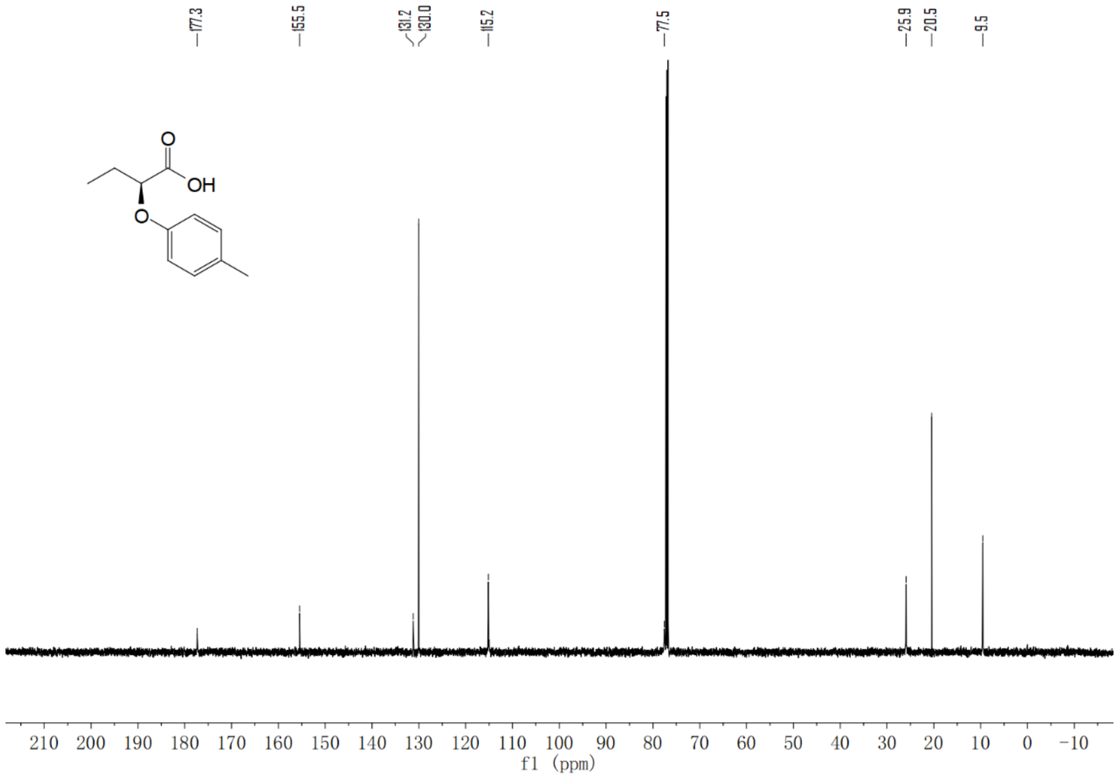

Supplementary Figure 50. ^{13}C NMR (151 MHz, $CDCl_3$) spectrum of **2q**

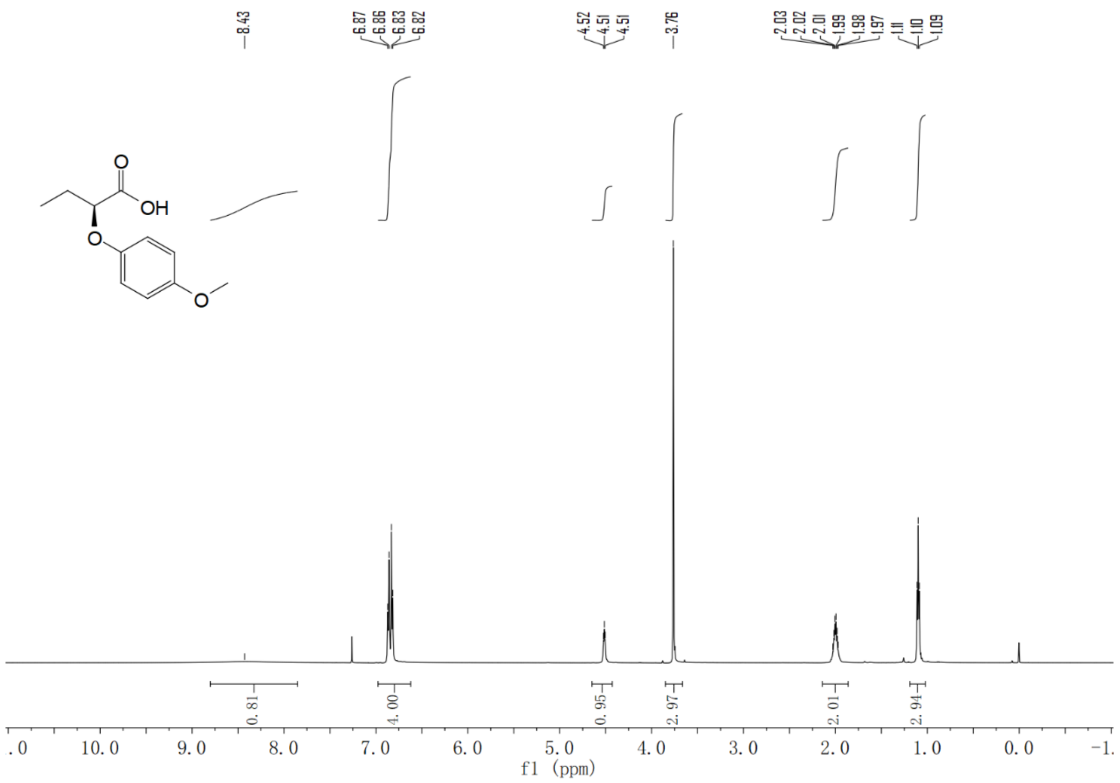

Supplementary Figure 51. ^{1}H NMR (600 MHz, $CDCl_3$) spectrum of **2r**

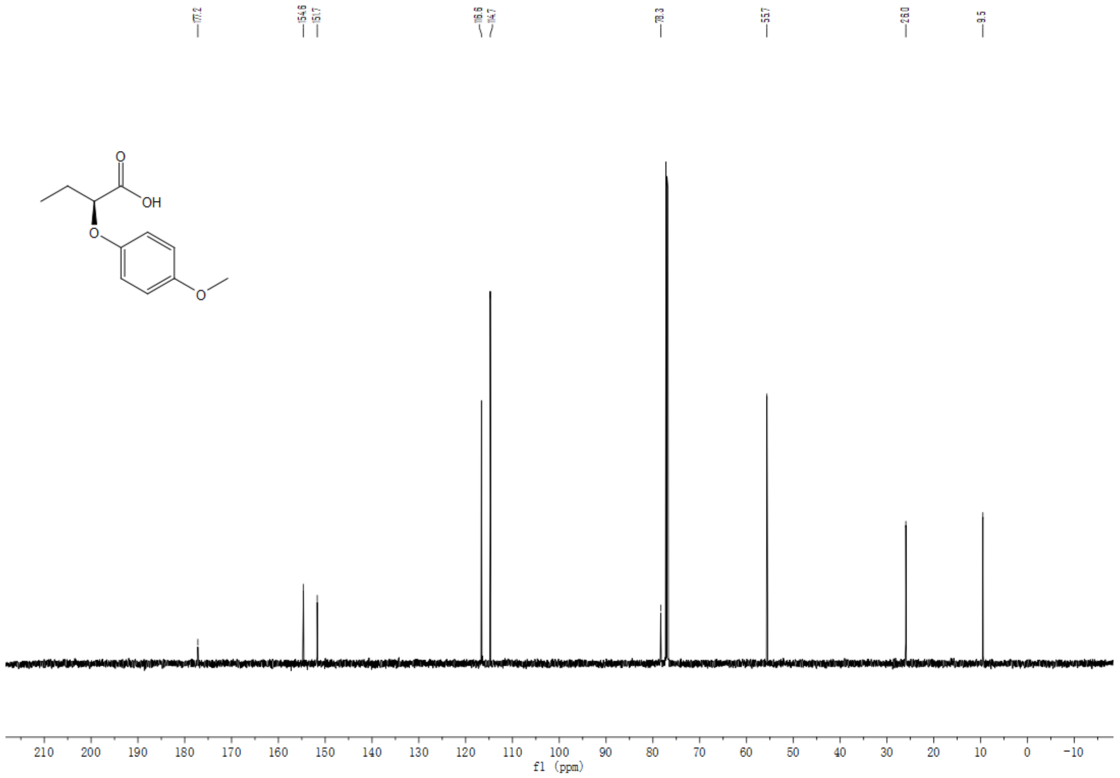

Supplementary Figure 52. ^{13}C NMR (151 MHz, $CDCl_3$) spectrum of **2r**

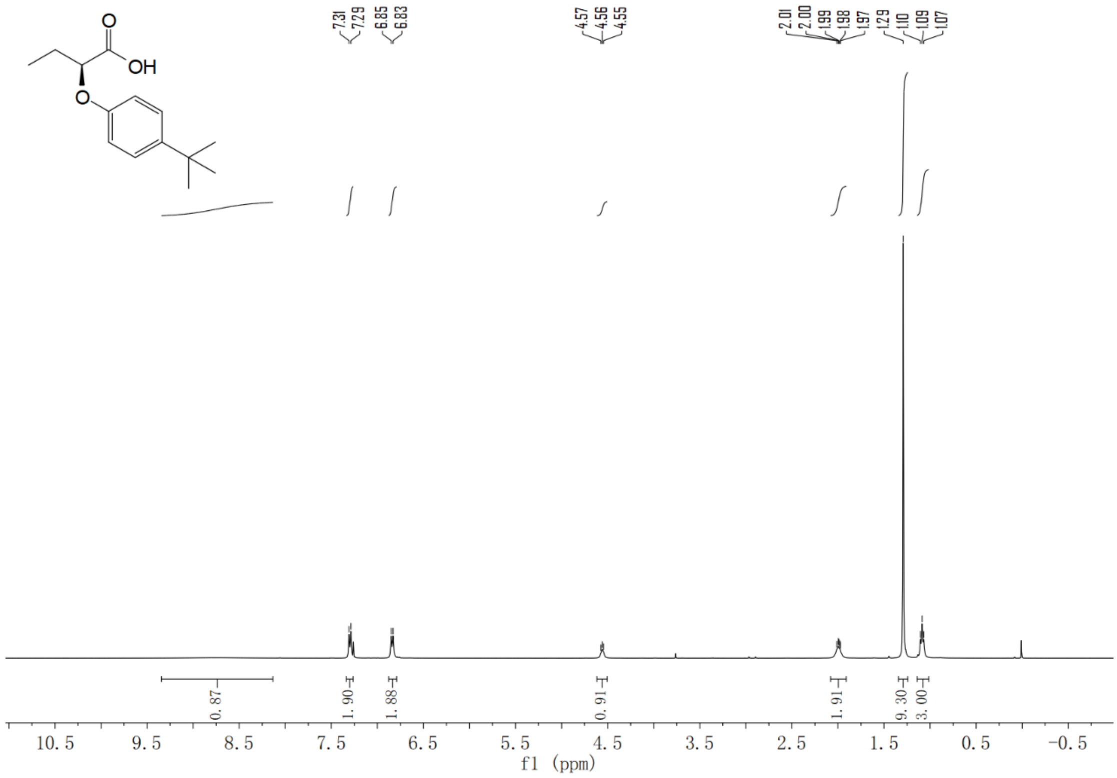

Supplementary Figure 53. ^{1}H NMR (400 MHz, CDCl$_3$) spectrum of **2s**

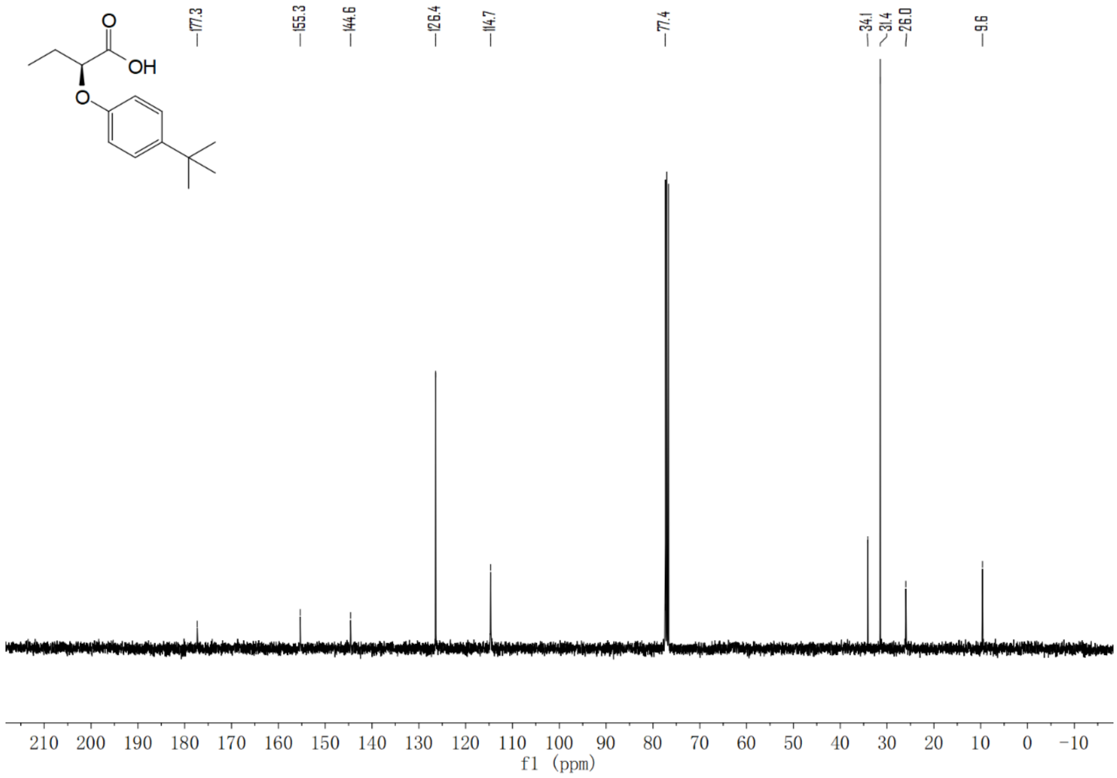

Supplementary Figure 54. ^{13}C NMR (101 MHz, CDCl$_3$) spectrum of **2s**

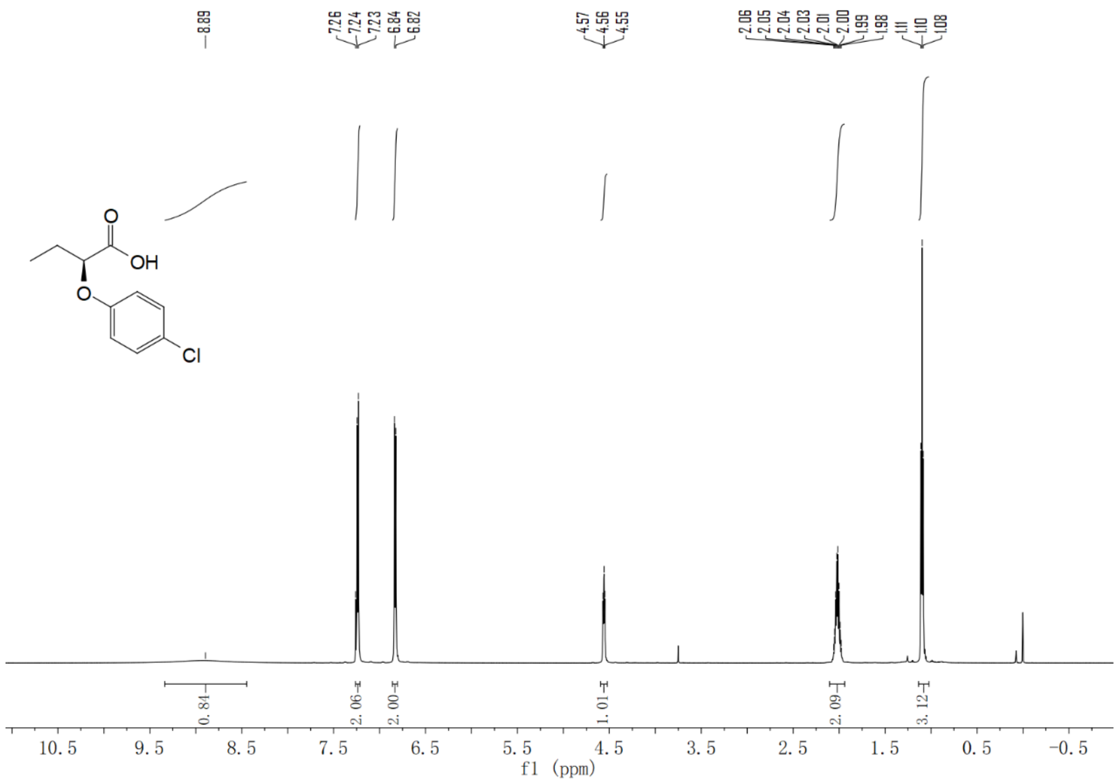

Supplementary Figure 55. ^{1}H NMR (600 MHz, $CDCl_3$) spectrum of **2t**

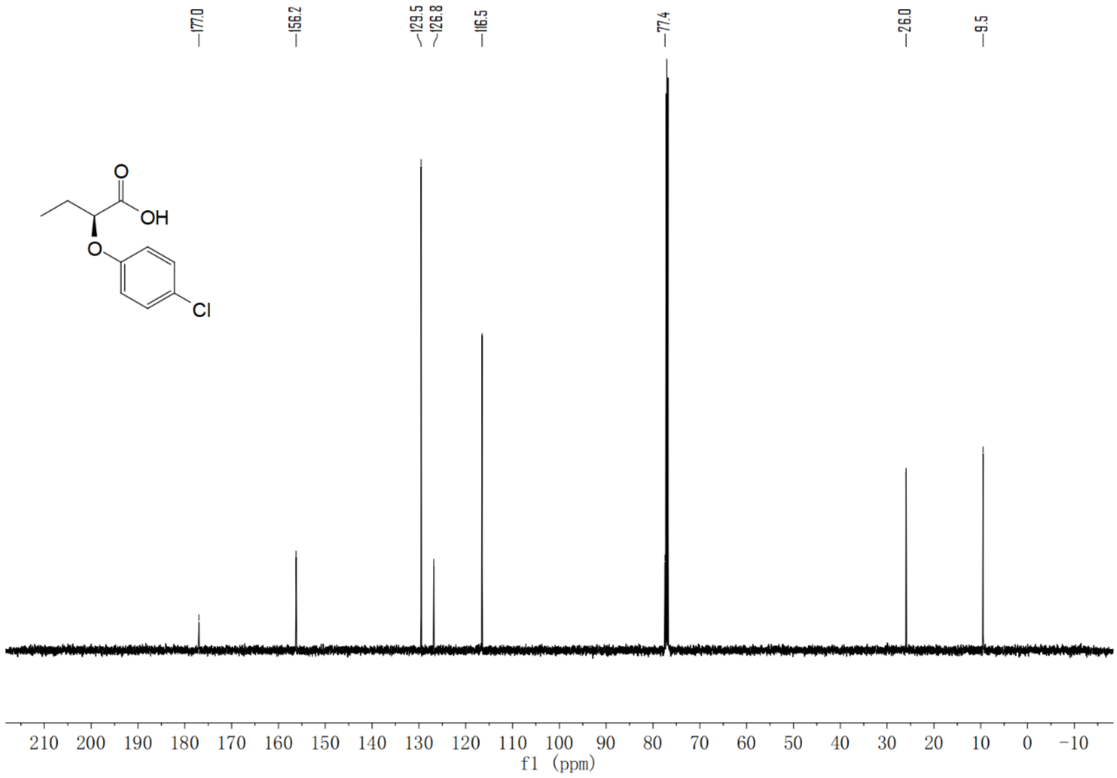

Supplementary Figure 56. ^{13}C NMR (151 MHz, $CDCl_3$) spectrum of **2t**

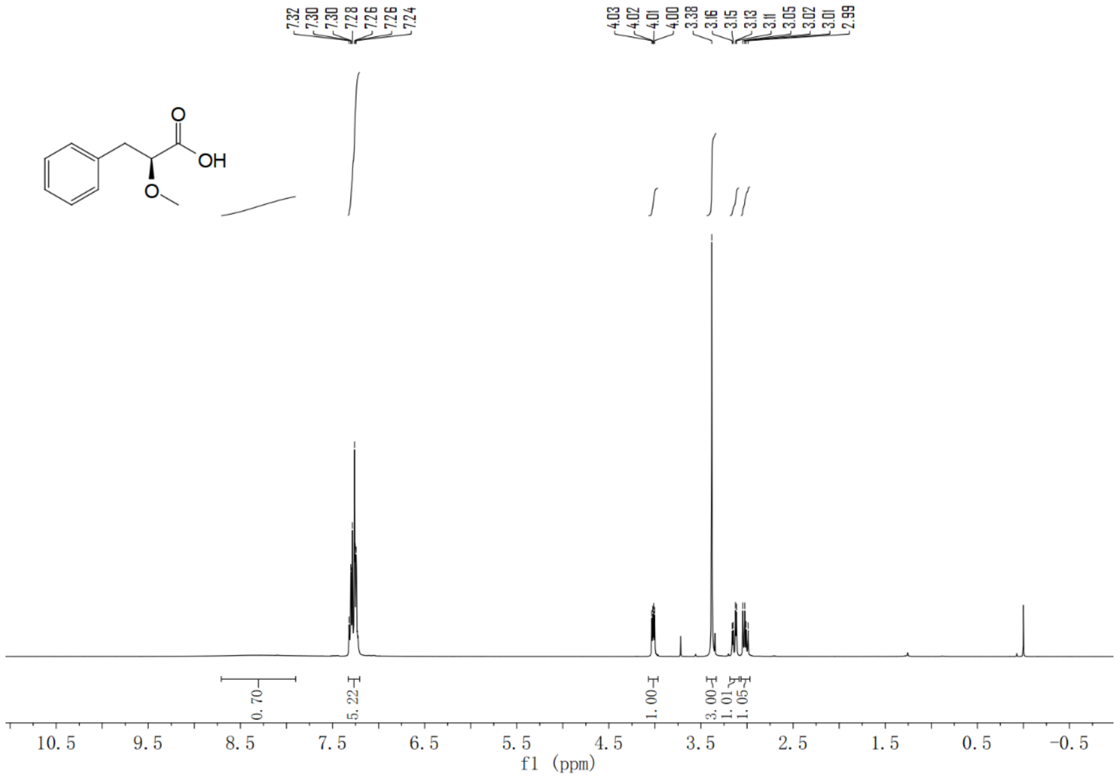

Supplementary Figure 57. ^{1}H NMR (400 MHz, $CDCl_3$) spectrum of **2u**

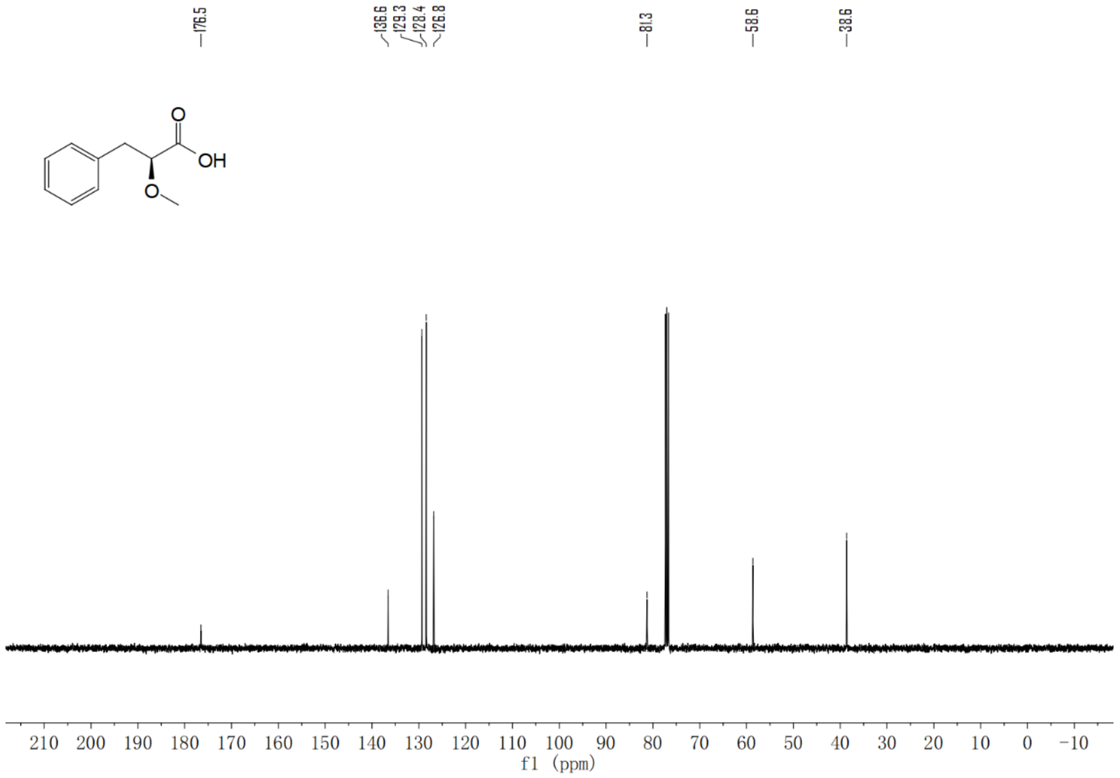

Supplementary Figure 58. ^{13}C NMR (101 MHz, $CDCl_3$) spectrum of **2u**

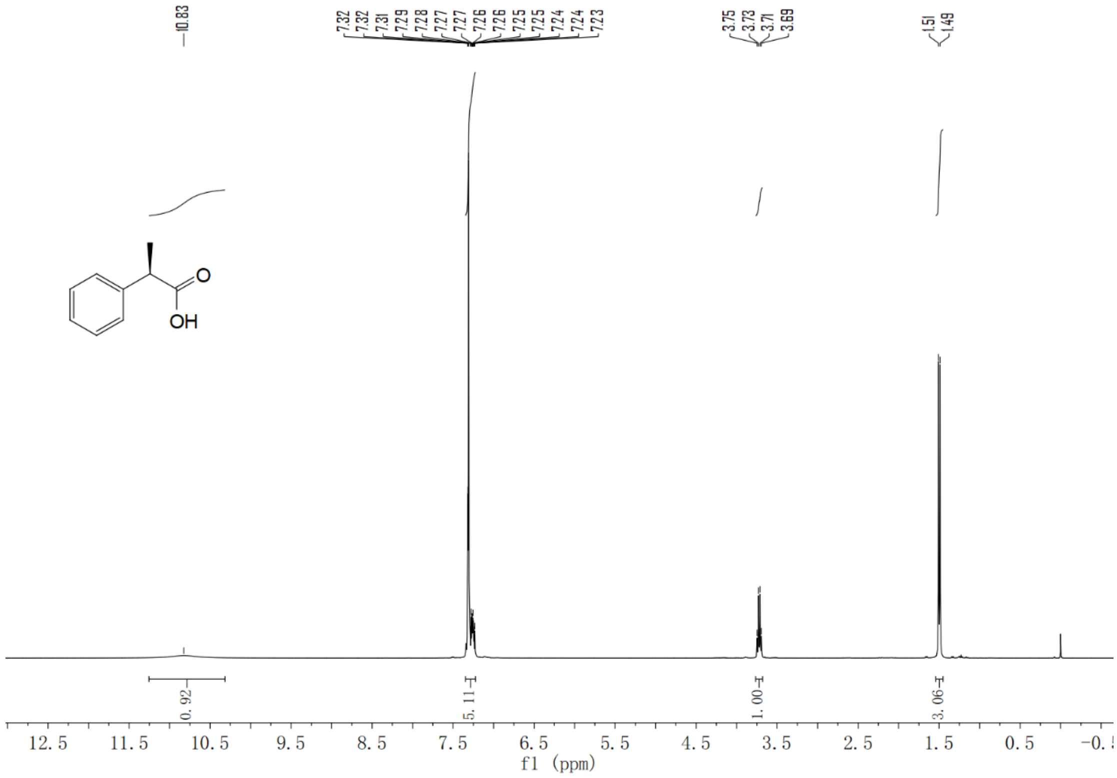

Supplementary Figure 59. ^{1}H NMR (400 MHz, $CDCl_3$) spectrum of **4a**

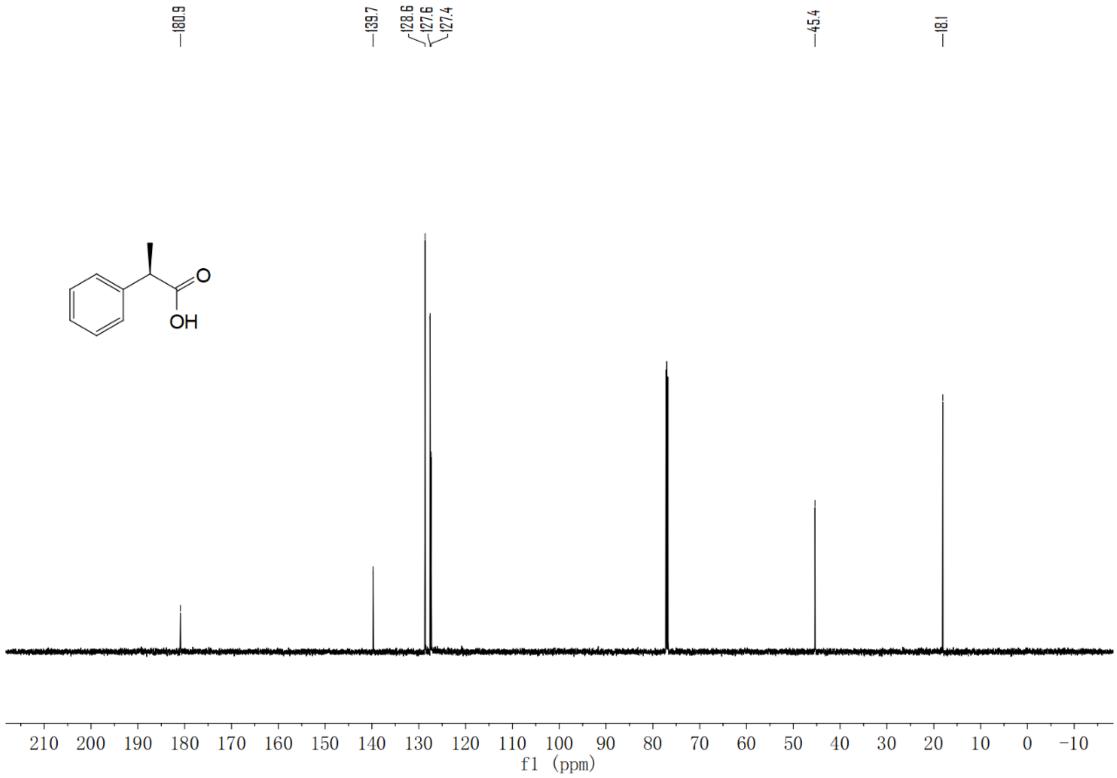

Supplementary Figure 60. ^{13}C NMR (151 MHz, $CDCl_3$) spectrum of **4a**

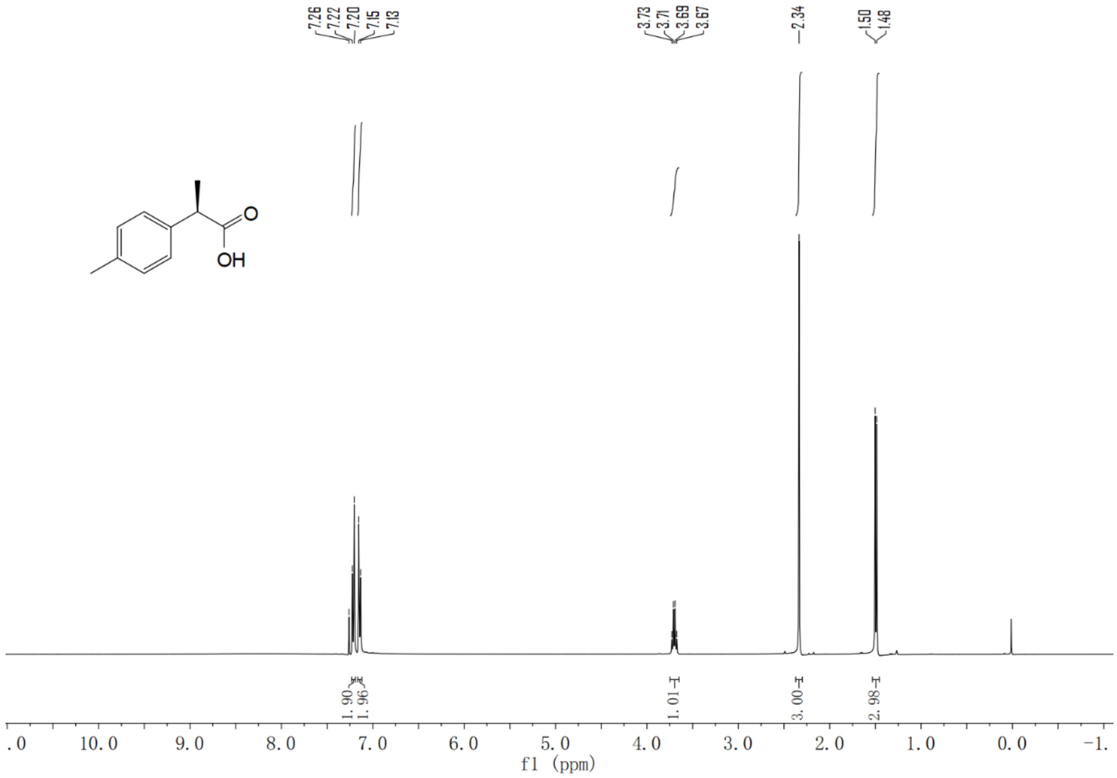

Supplementary Figure 61. ^{1}H NMR (400 MHz, $CDCl_3$) spectrum of **4b**

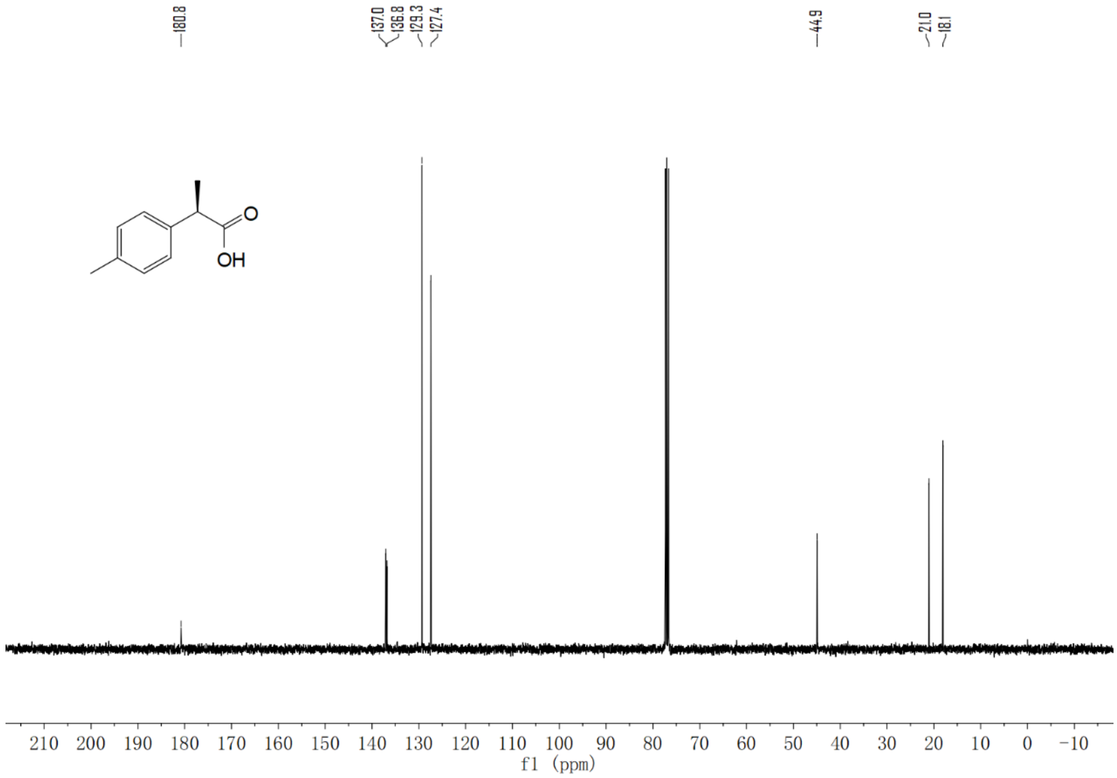

Supplementary Figure 62. ^{13}C NMR (101 MHz, $CDCl_3$) spectrum of **4b**

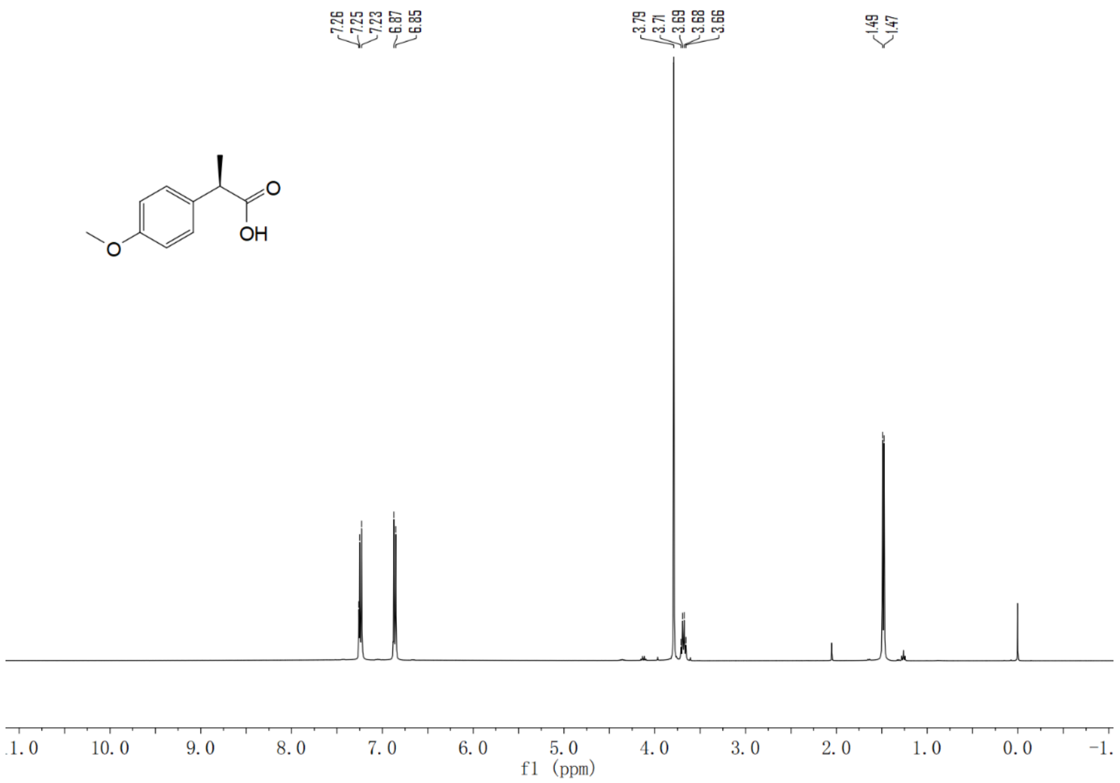

Supplementary Figure 63. ^{1}H NMR (400 MHz, $CDCl_3$) spectrum of **4c**

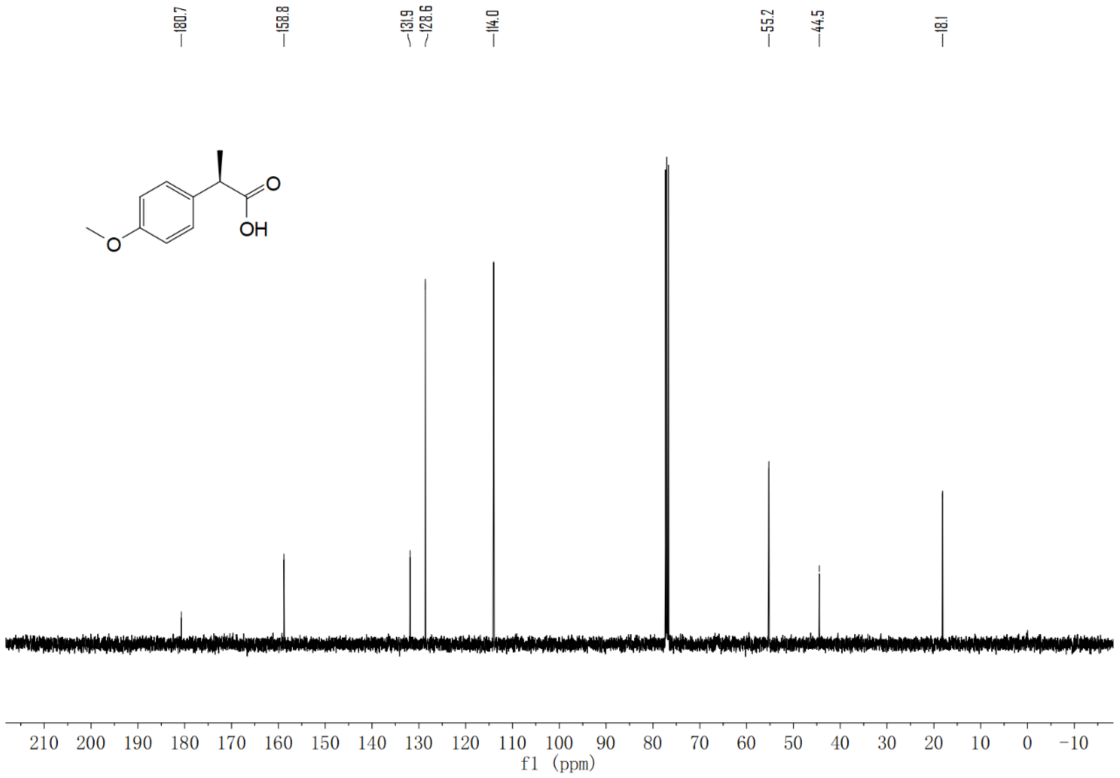

Supplementary Figure 64. ^{13}C NMR (101 MHz, $CDCl_3$) spectrum of **4c**

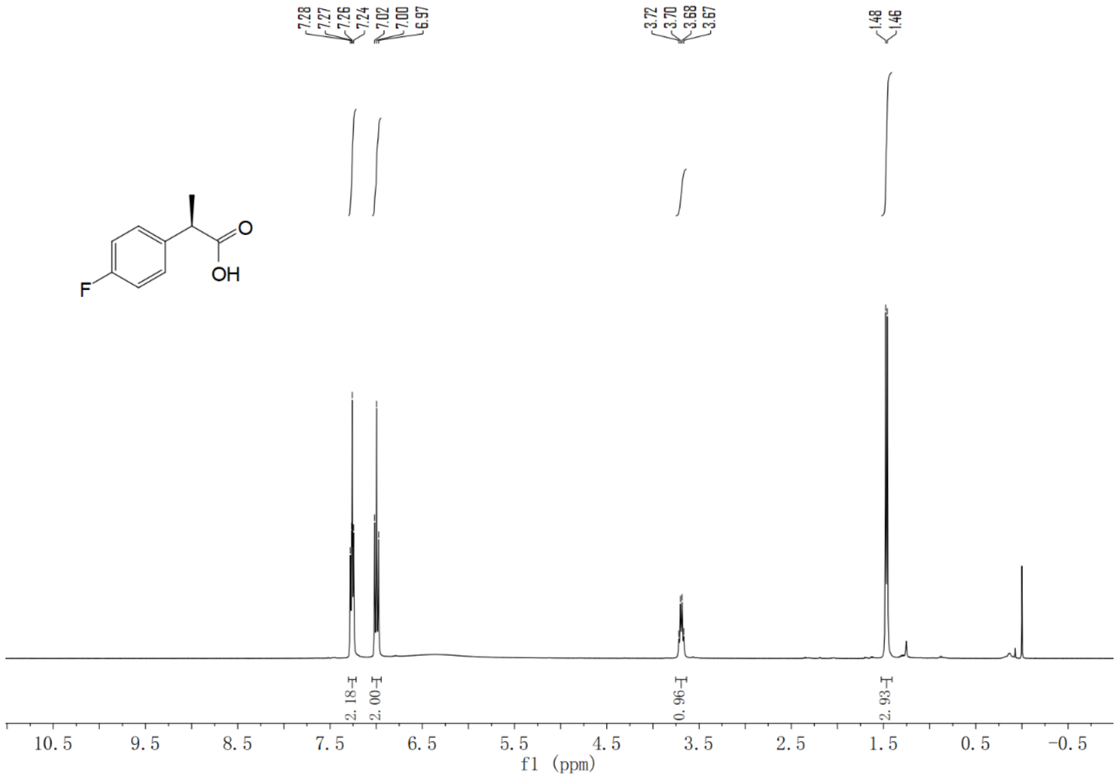

Supplementary Figure 65. ^{1}H NMR (400 MHz, $CDCl_3$) spectrum of **4d**

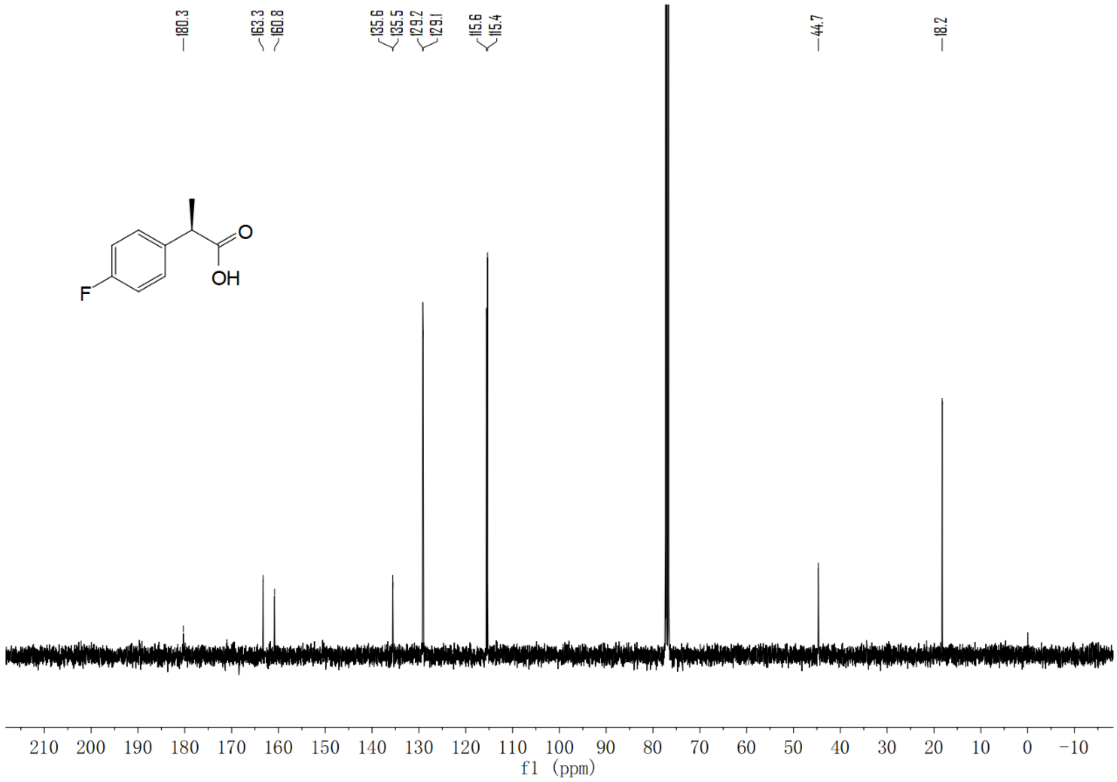

Supplementary Figure 66. ^{13}C NMR (101 MHz, $CDCl_3$) spectrum of **4d**

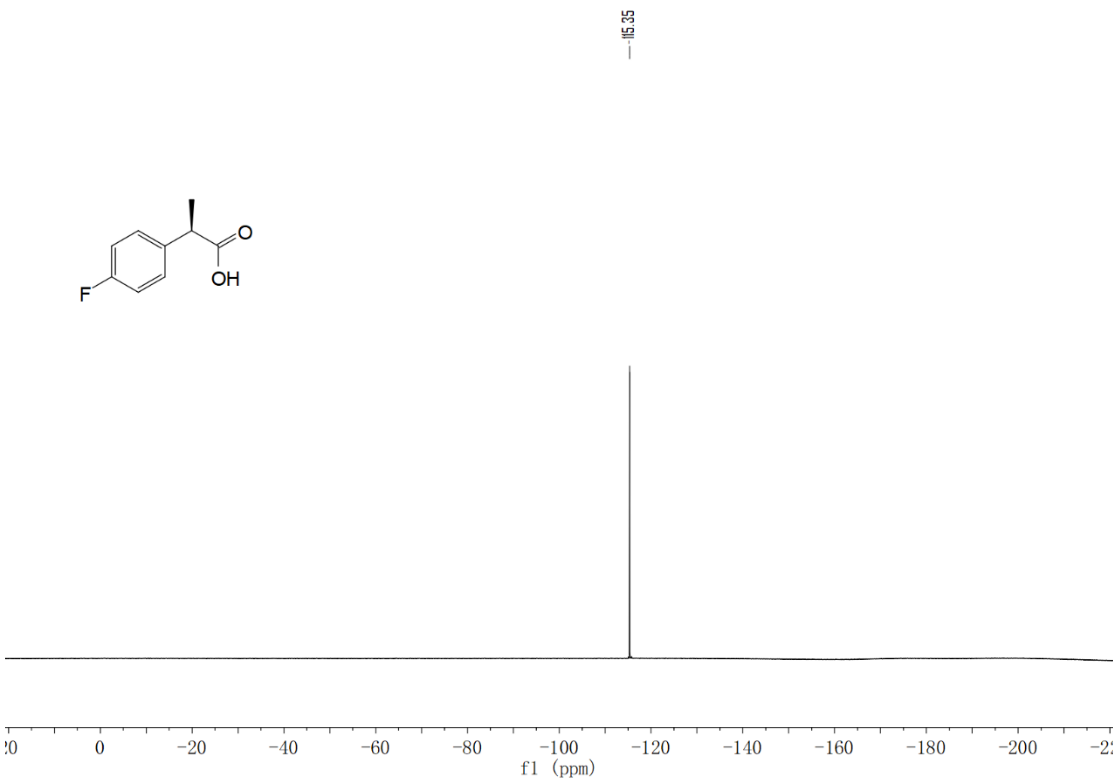

Supplementary Figure 67. ^{19}F NMR (376 MHz, $CDCl_3$) spectrum of **4d**

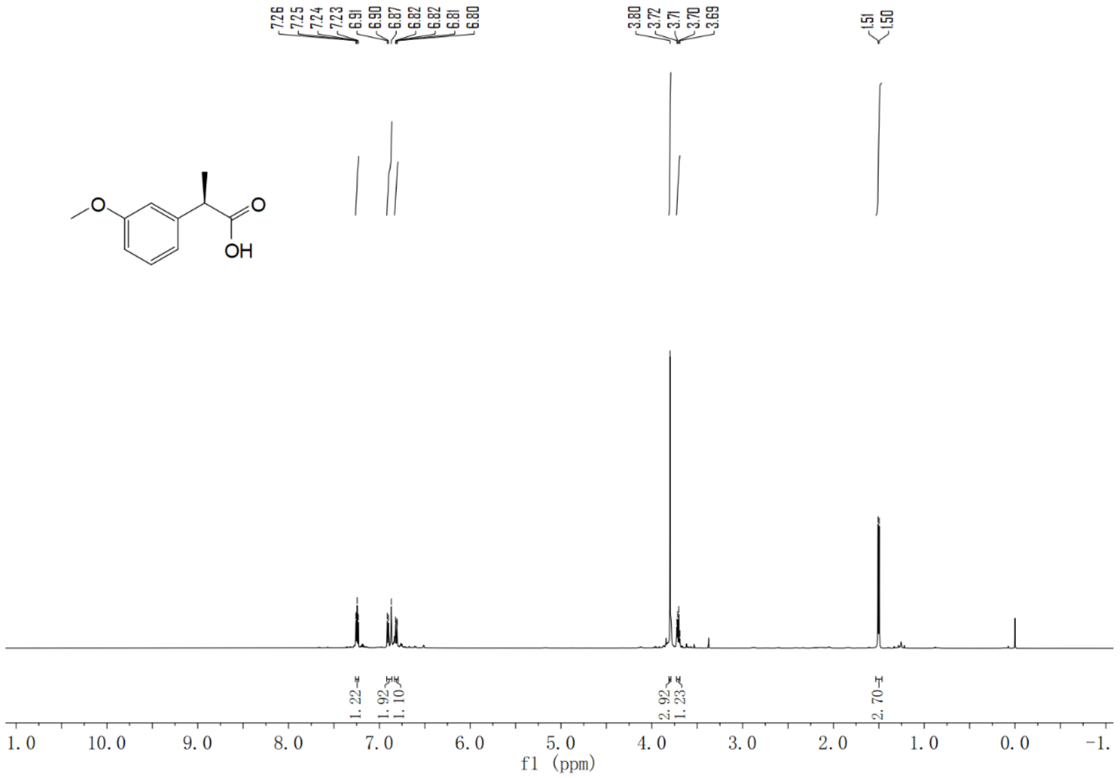

Supplementary Figure 68. ^{1}H NMR (600 MHz, $CDCl_3$) spectrum of **4e**

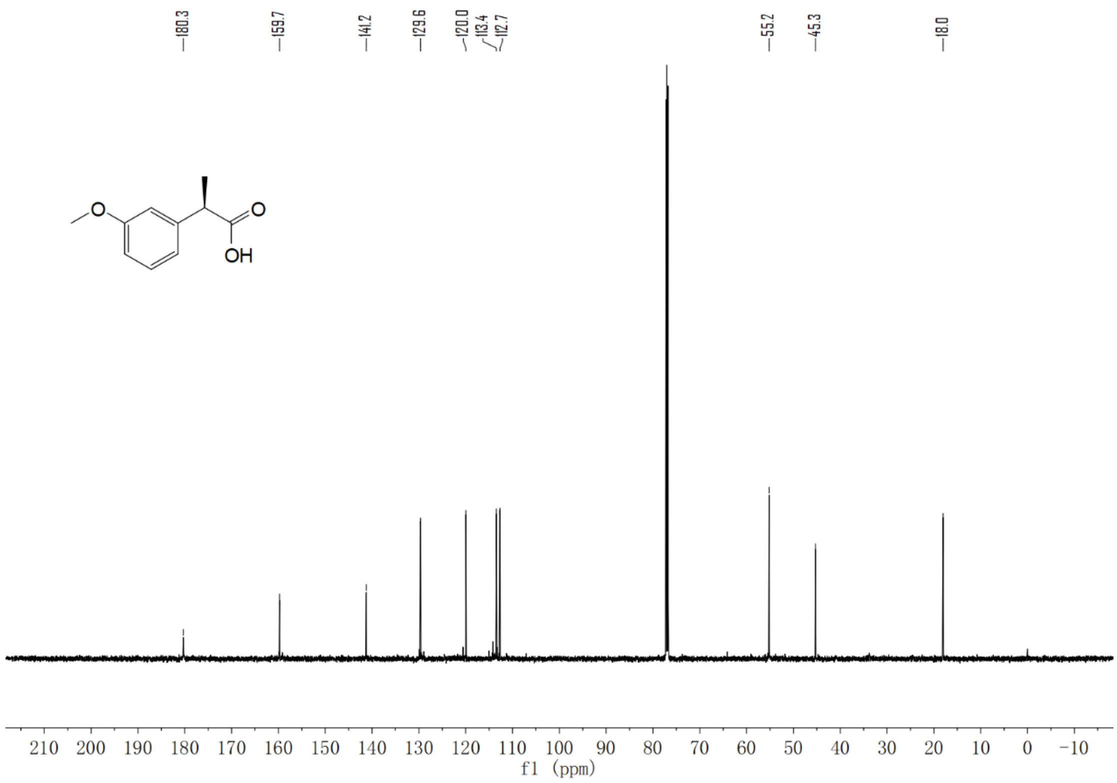

Supplementary Figure 69. ^{13}C NMR (151 MHz, $CDCl_3$) spectrum of **4e**

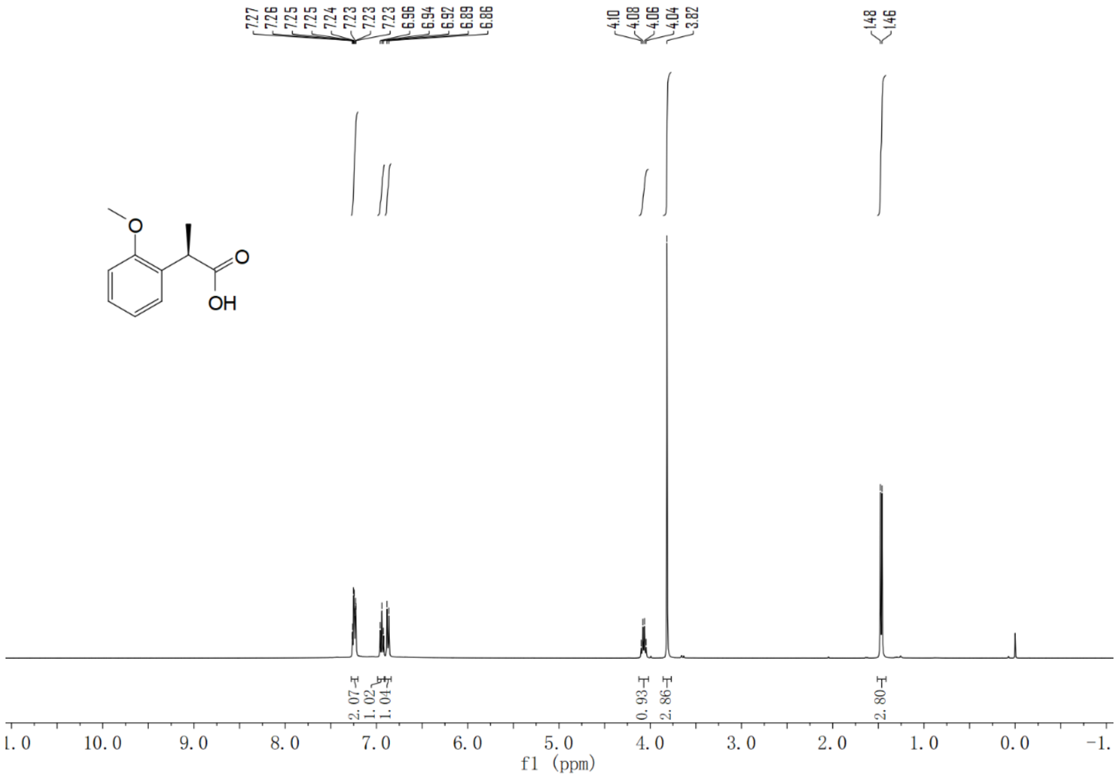

Supplementary Figure 70. ^{1}H NMR (400 MHz, $CDCl_3$) spectrum of **4f**

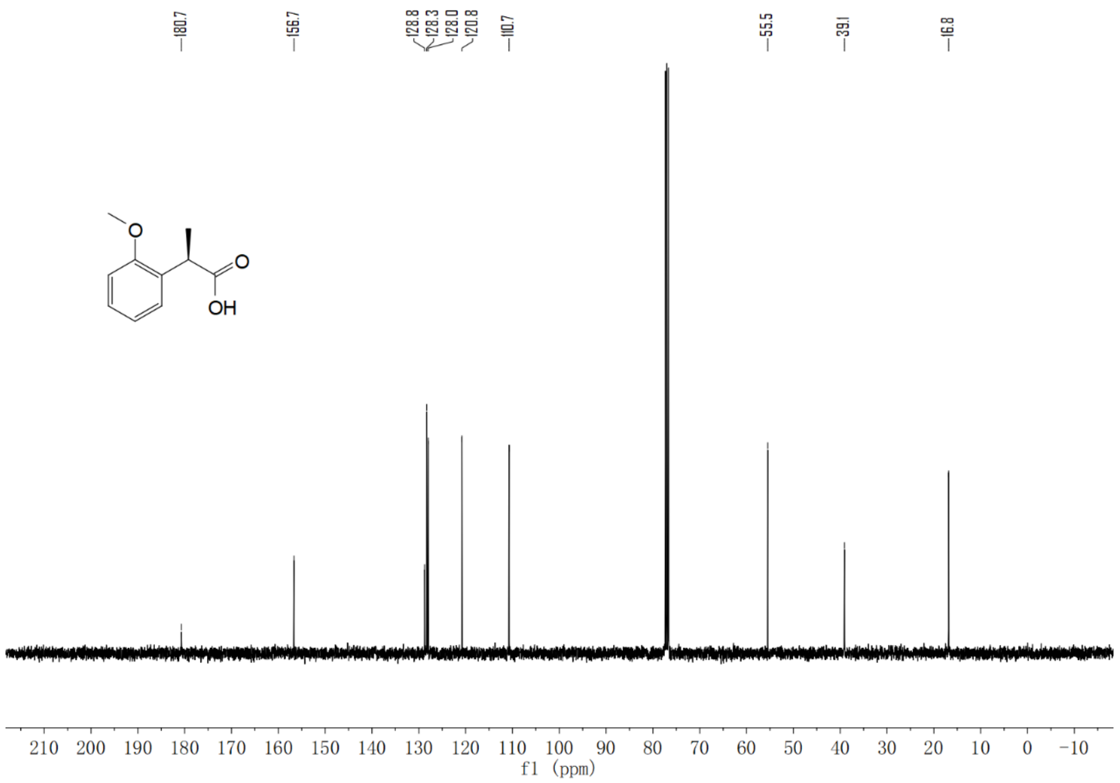

Supplementary Figure 71. ^{13}C NMR (101 MHz, $CDCl_3$) spectrum of **4f**

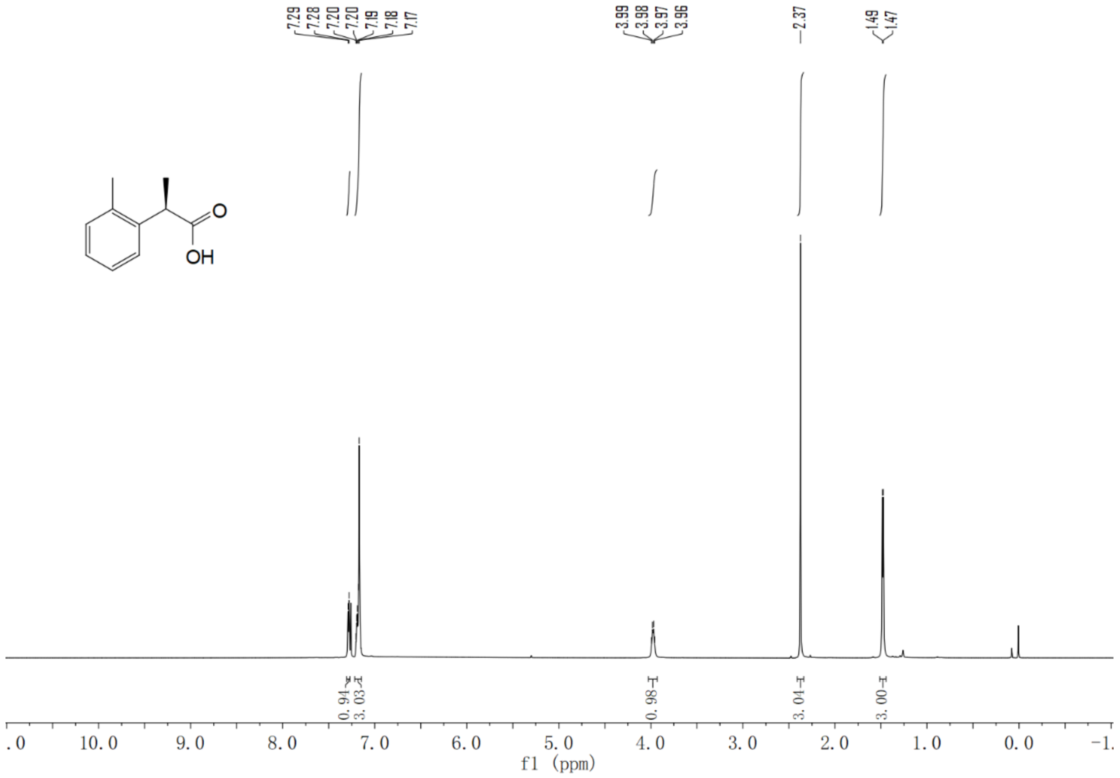

Supplementary Figure 72. ^{1}H NMR (600 MHz, $CDCl_3$) spectrum of **4g**

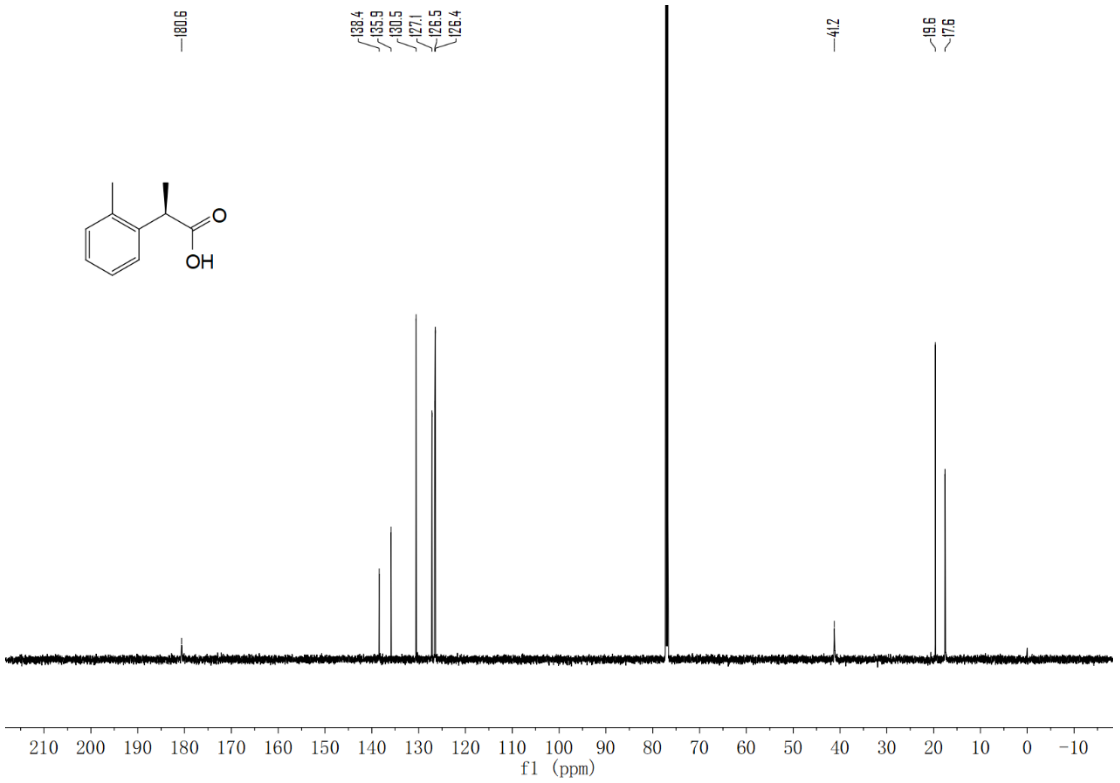

Supplementary Figure 73. ^{13}C NMR (151 MHz, $CDCl_3$) spectrum of **4g**

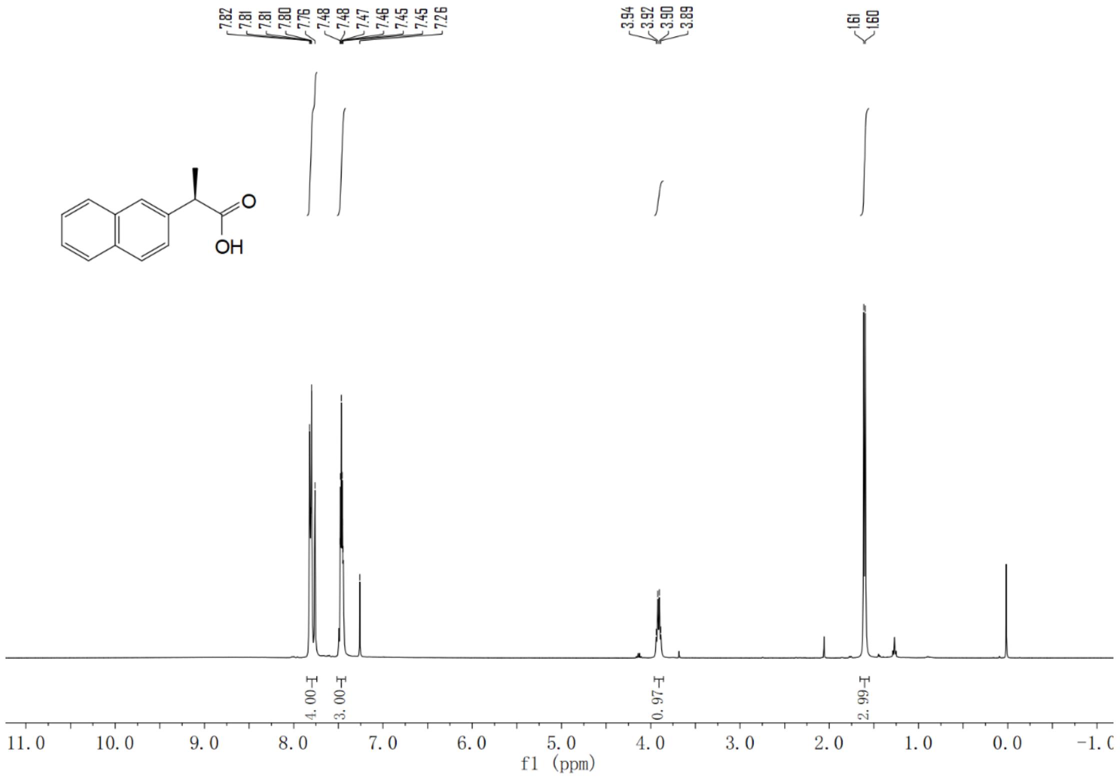

Supplementary Figure 74. ^{1}H NMR (400 MHz, $CDCl_3$) spectrum of **4h**

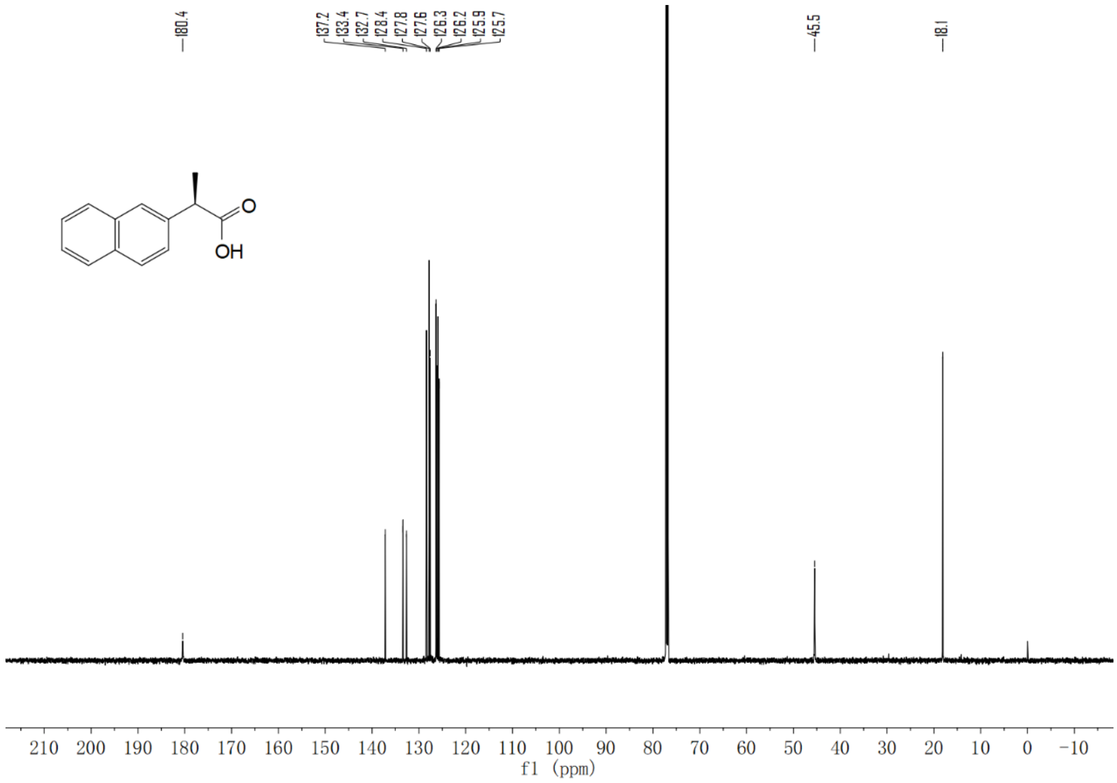

Supplementary Figure 75. ^{13}C NMR (151 MHz, $CDCl_3$) spectrum of **4h**

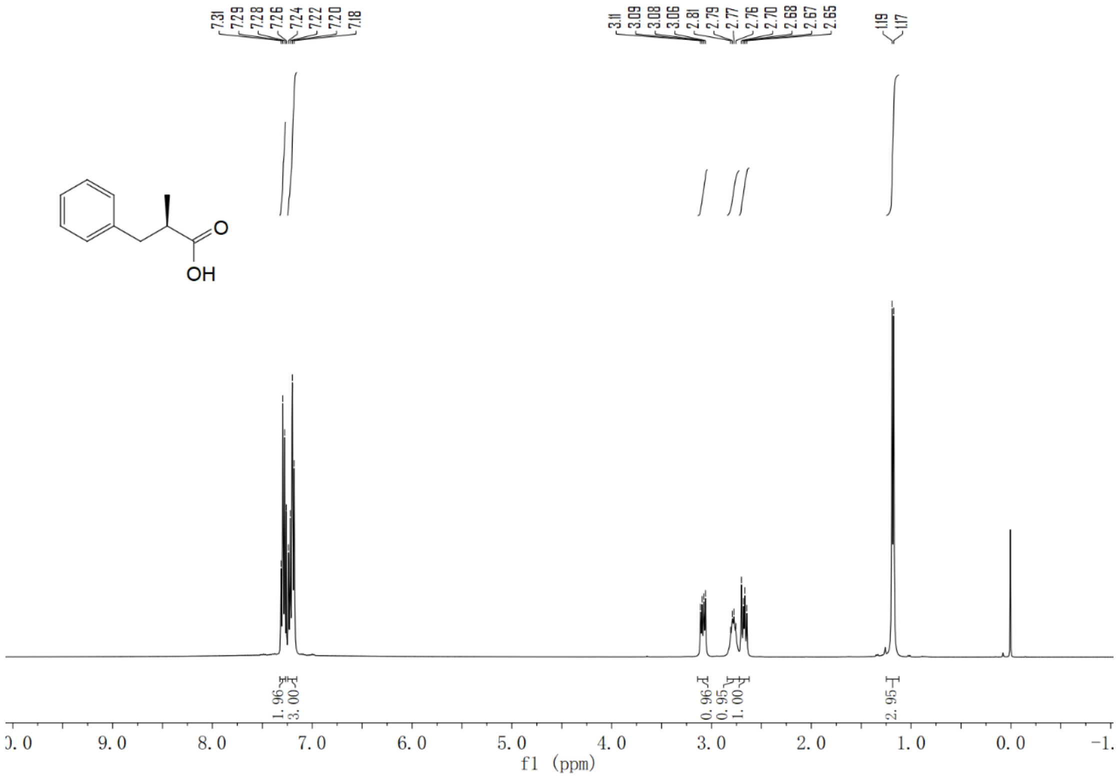

Supplementary Figure 76. ^{1}H NMR (400 MHz, $CDCl_3$) spectrum of **4i**

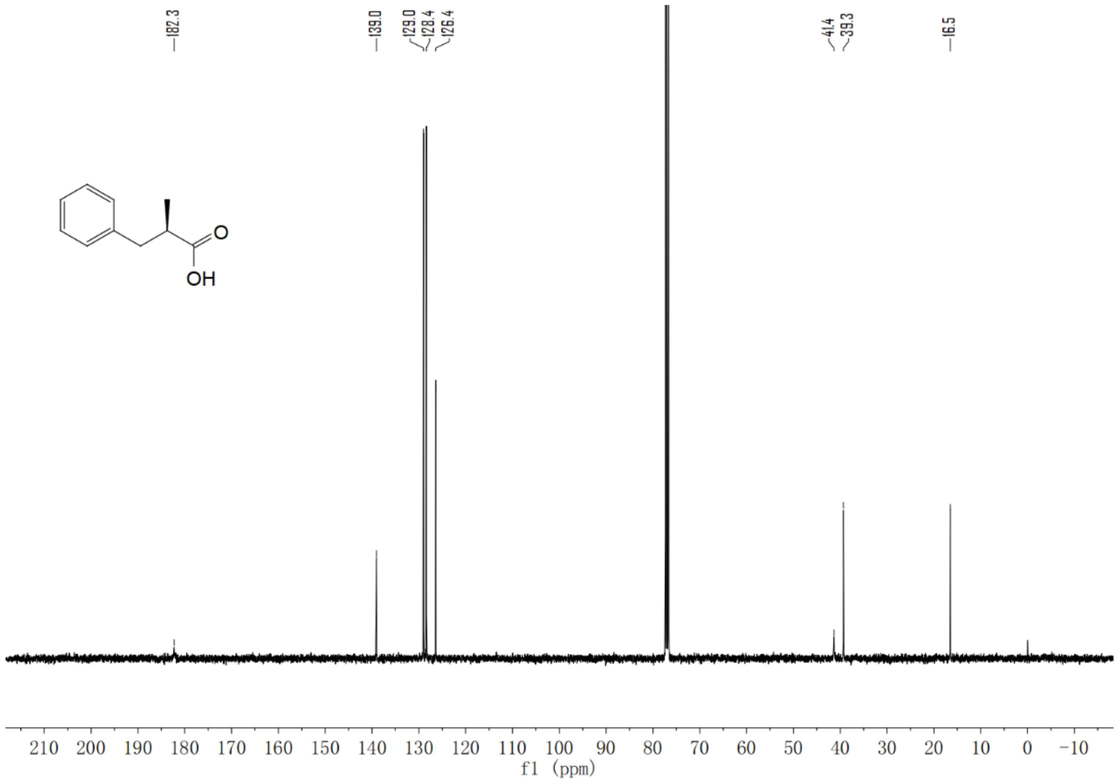

Supplementary Figure 77. ^{13}C NMR (101 MHz, $CDCl_3$) spectrum of **4i**

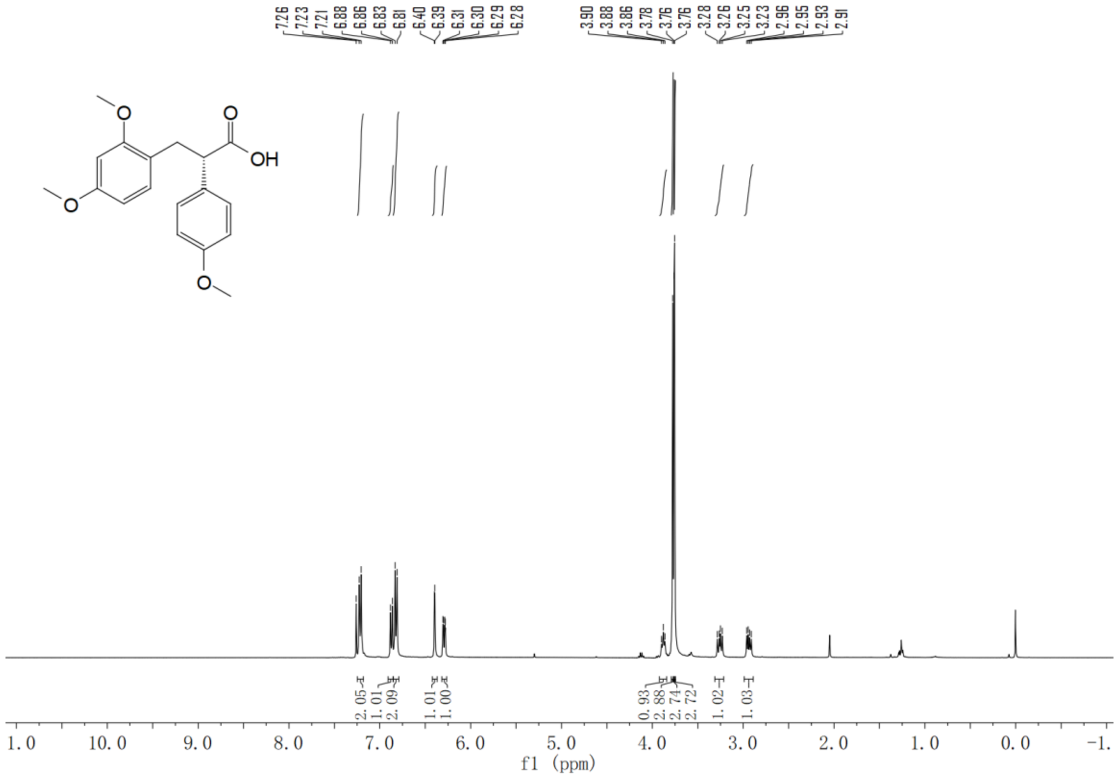

Supplementary Figure 78. ^{1}H NMR (400 MHz, $CDCl_3$) spectrum of **2v**

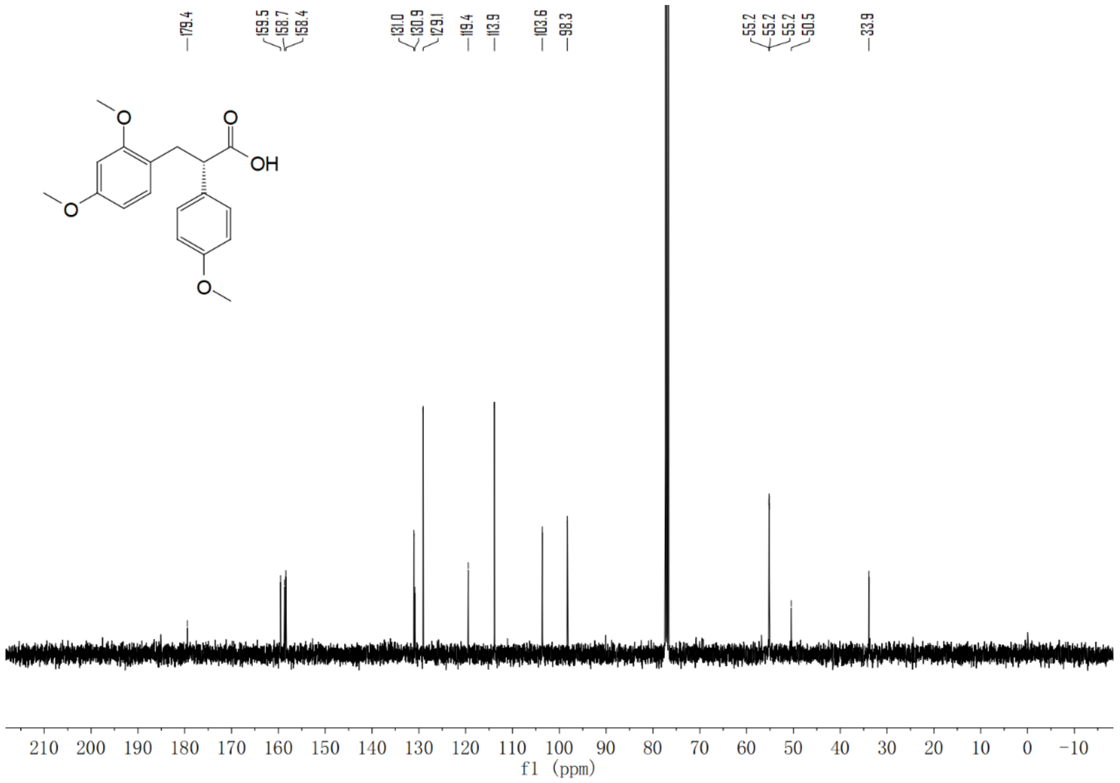

Supplementary Figure 79. ^{13}C NMR (101 MHz, $CDCl_3$) spectrum of **2v**

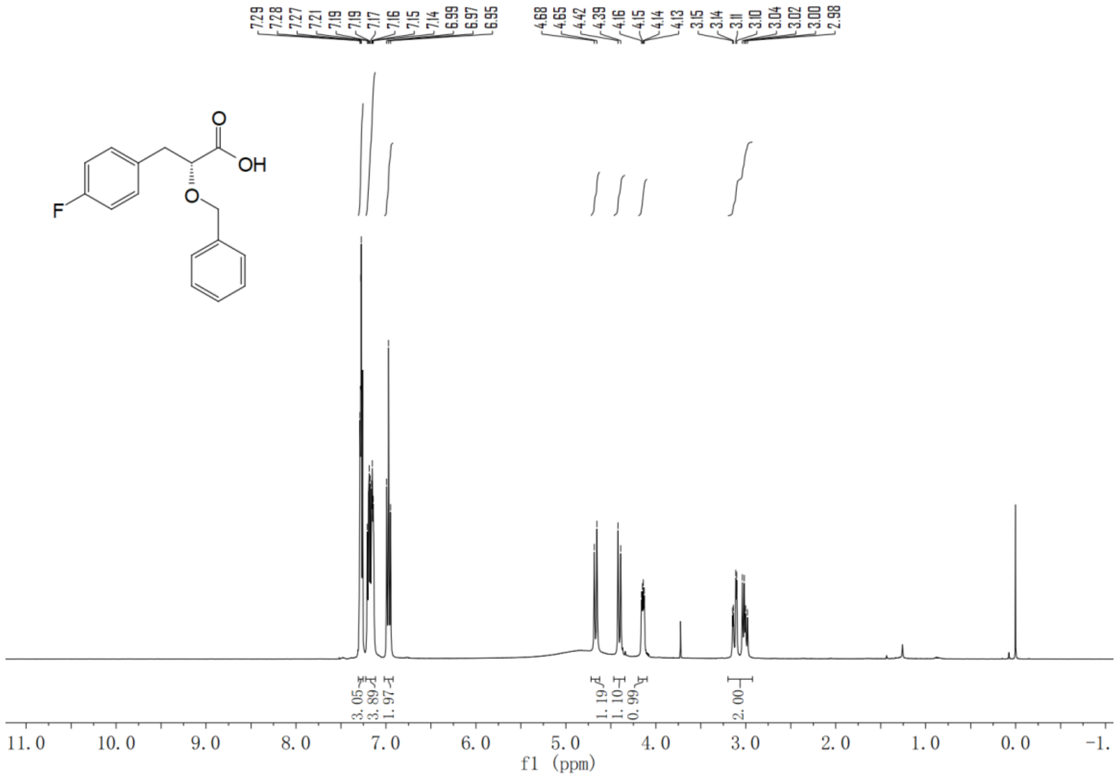

Supplementary Figure 80. ^{1}H NMR (400 MHz, $CDCl_3$) spectrum of **2w**

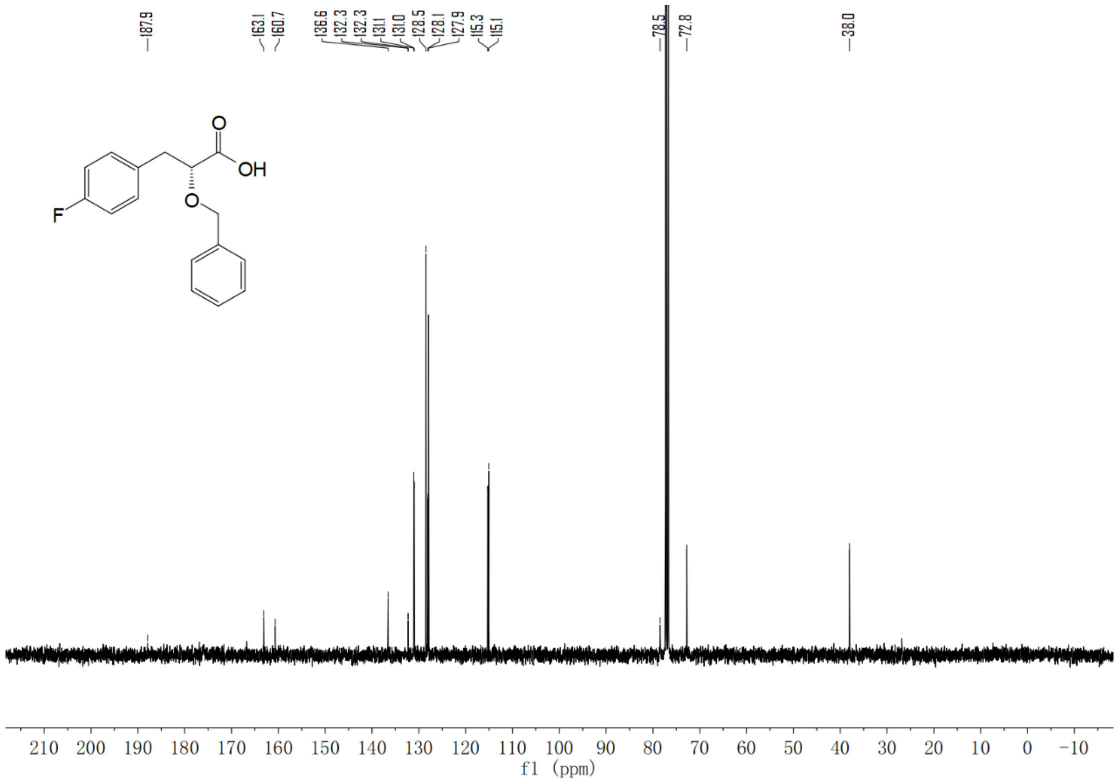

Supplementary Figure 81. ^{13}C NMR (101 MHz, $CDCl_3$) spectrum of **2w**

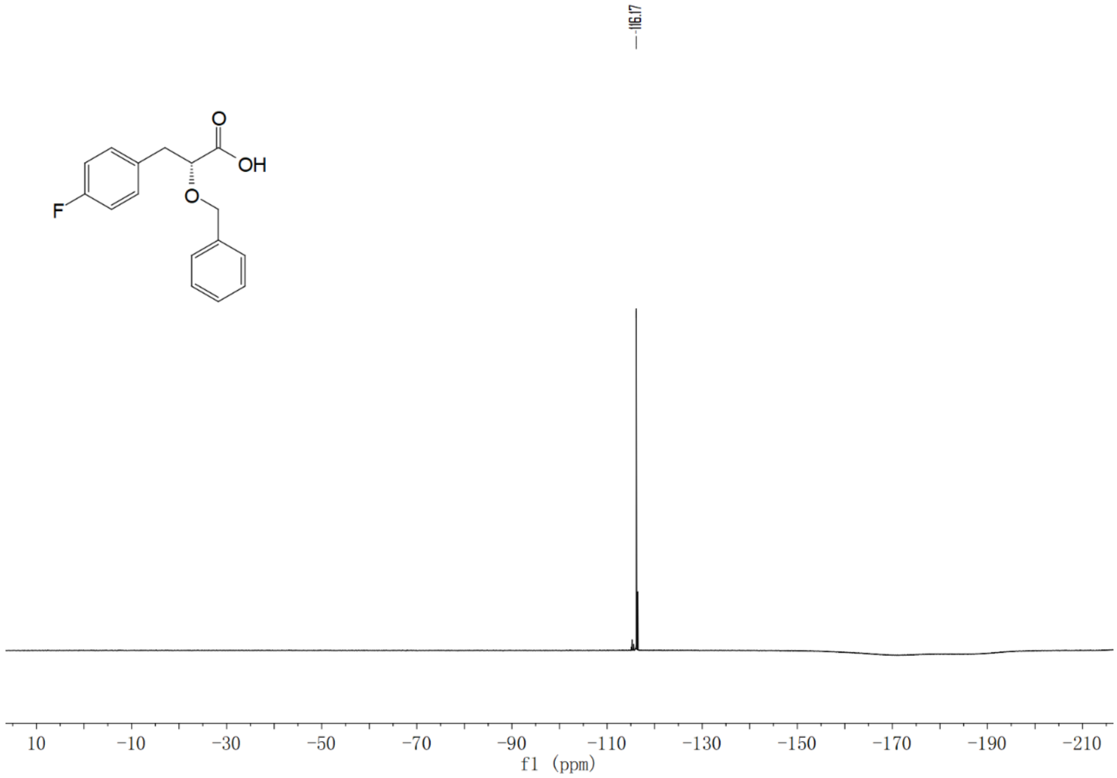

Supplementary Figure 82. ^{19}F NMR (565 MHz, $CDCl_3$) spectrum of **2w**

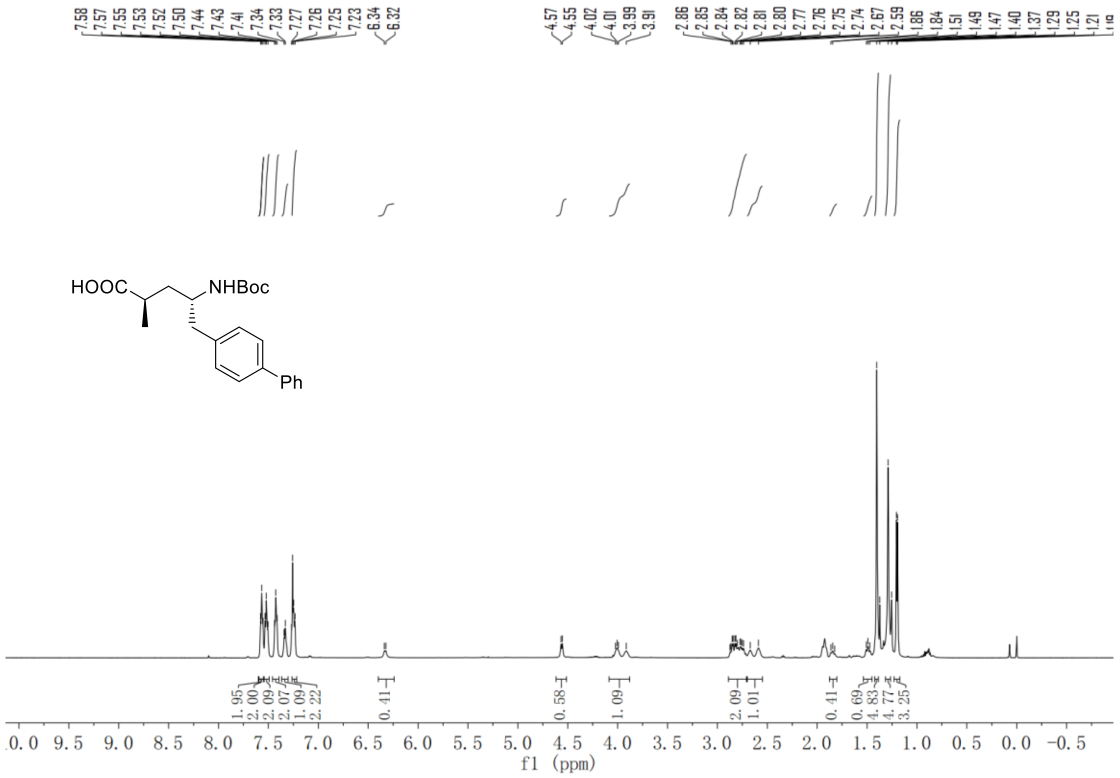

Supplementary Figure 83. ^{1}H NMR (600 MHz, $CDCl_3$) spectrum of **2x**

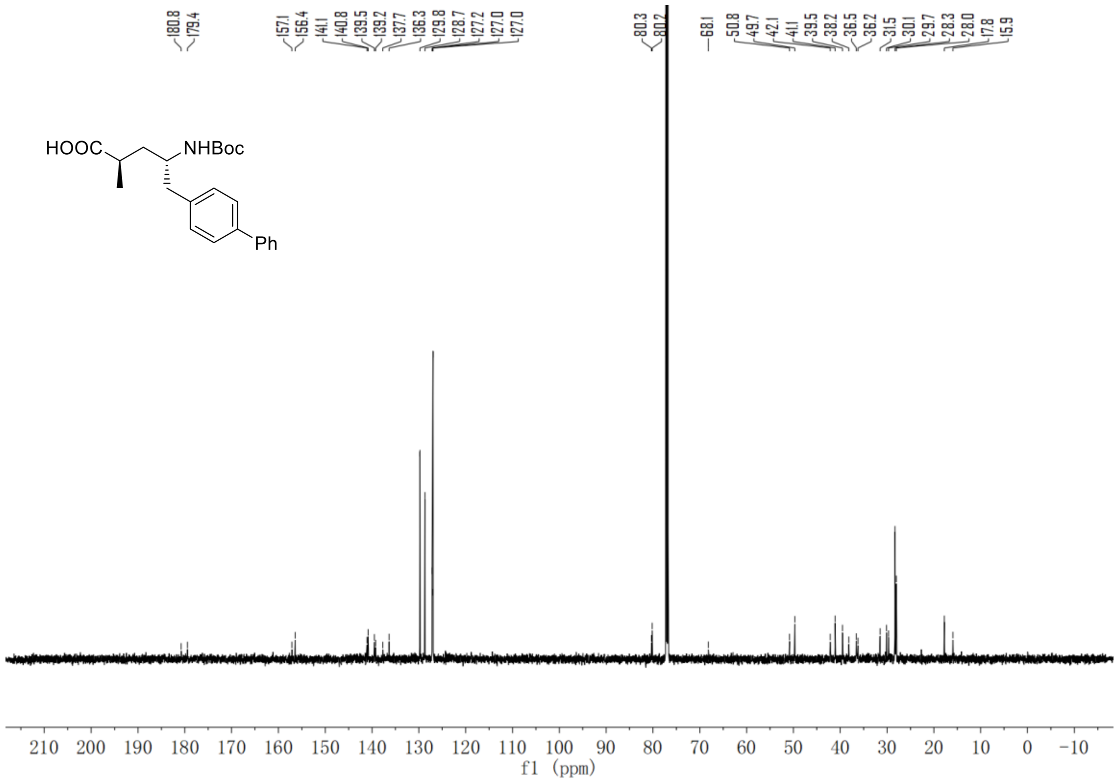

Supplementary Figure 84. ^{13}C NMR (151 MHz, $CDCl_3$) spectrum of **2x**

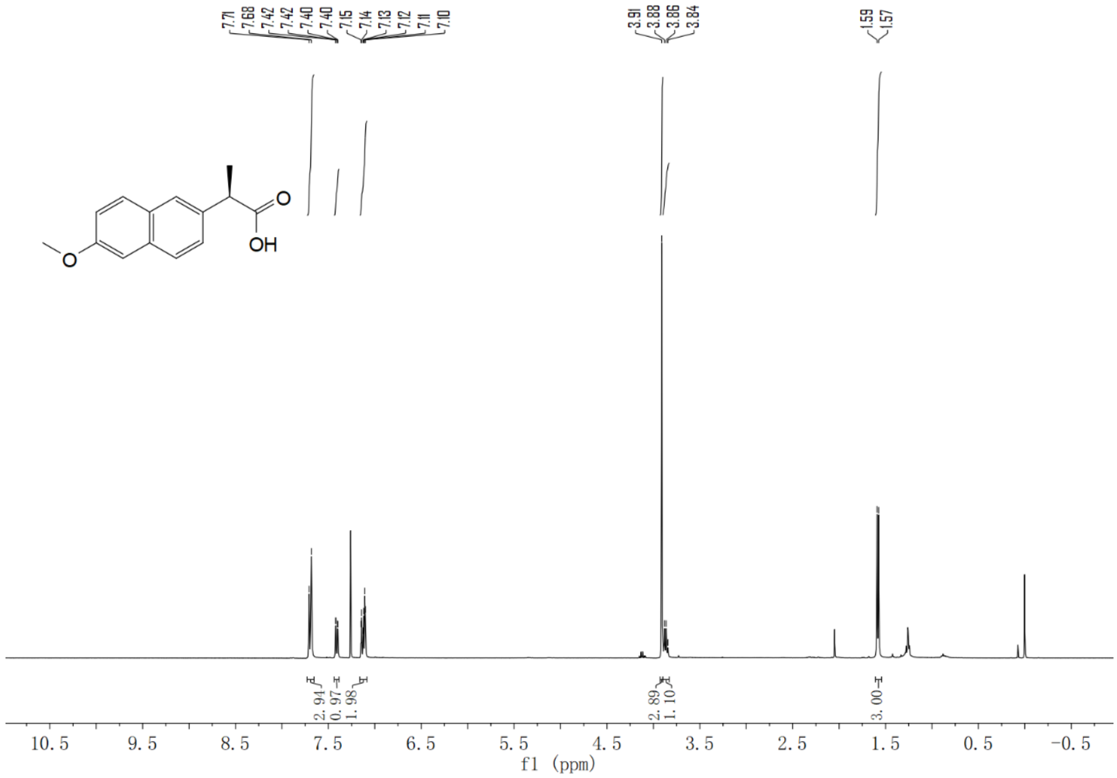

Supplementary Figure 85. ^{1}H NMR (400 MHz, $CDCl_3$) spectrum of **4j**

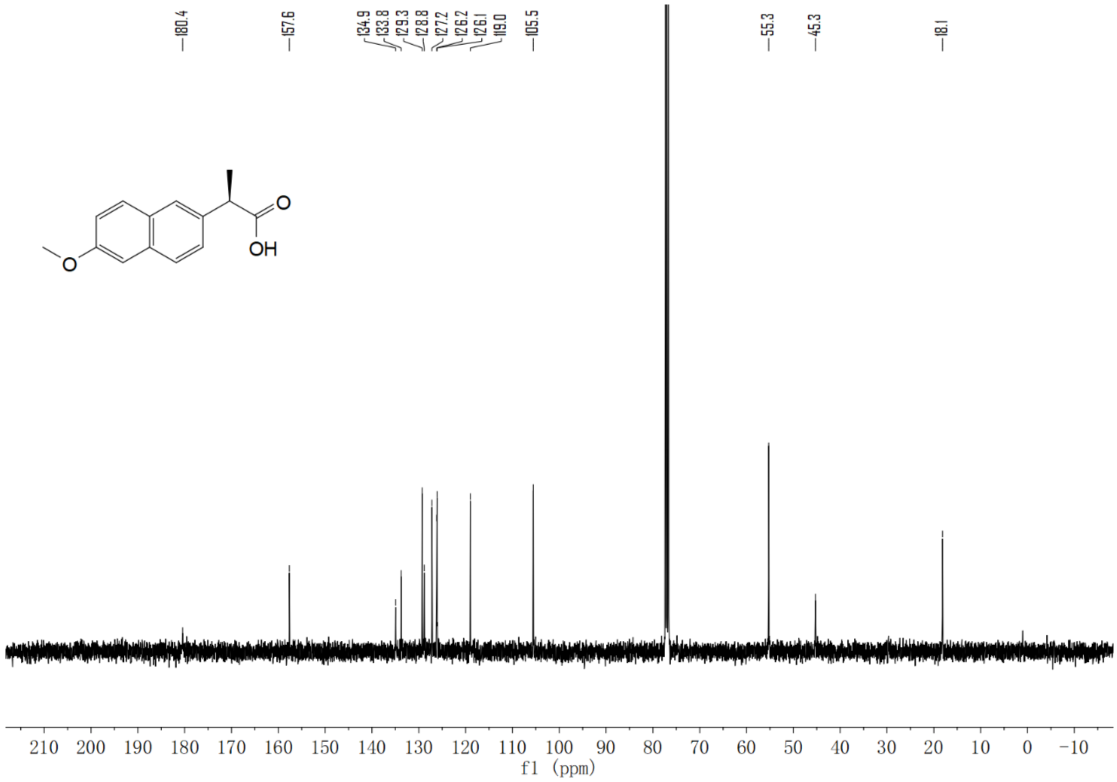

Supplementary Figure 86. ^{13}C NMR (101 MHz, $CDCl_3$) spectrum of **4j**

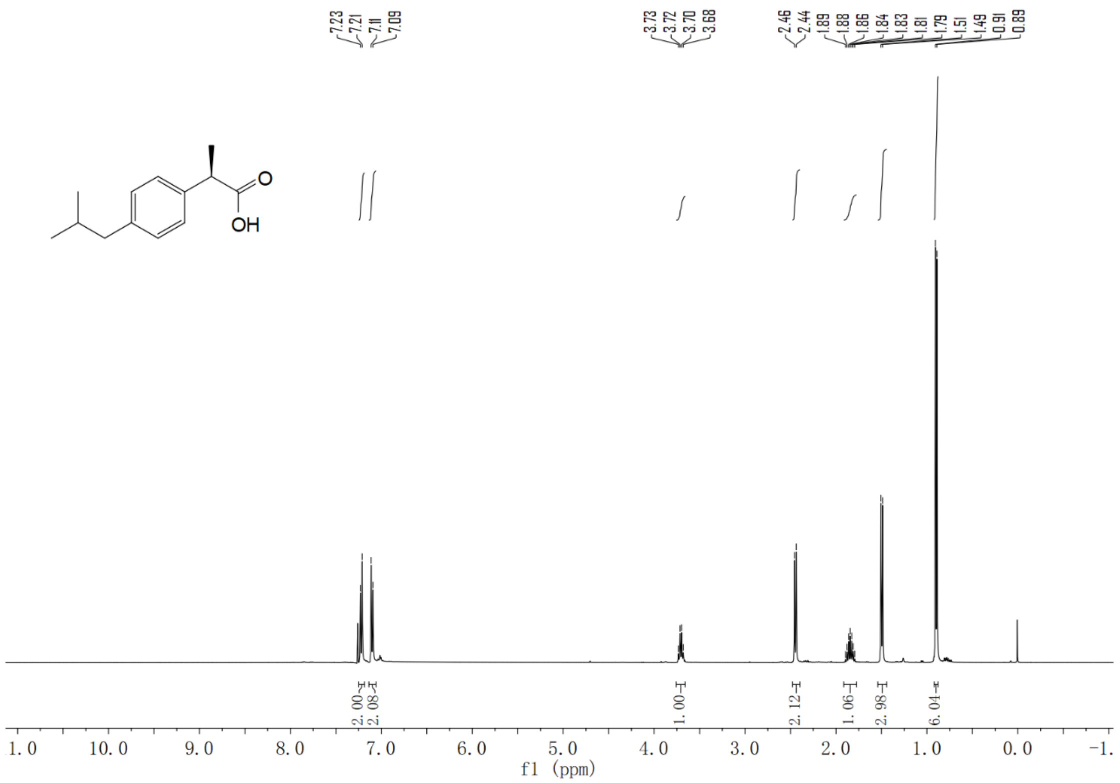

Supplementary Figure 87. ^{1}H NMR (400 MHz, $CDCl_3$) spectrum of **4k**

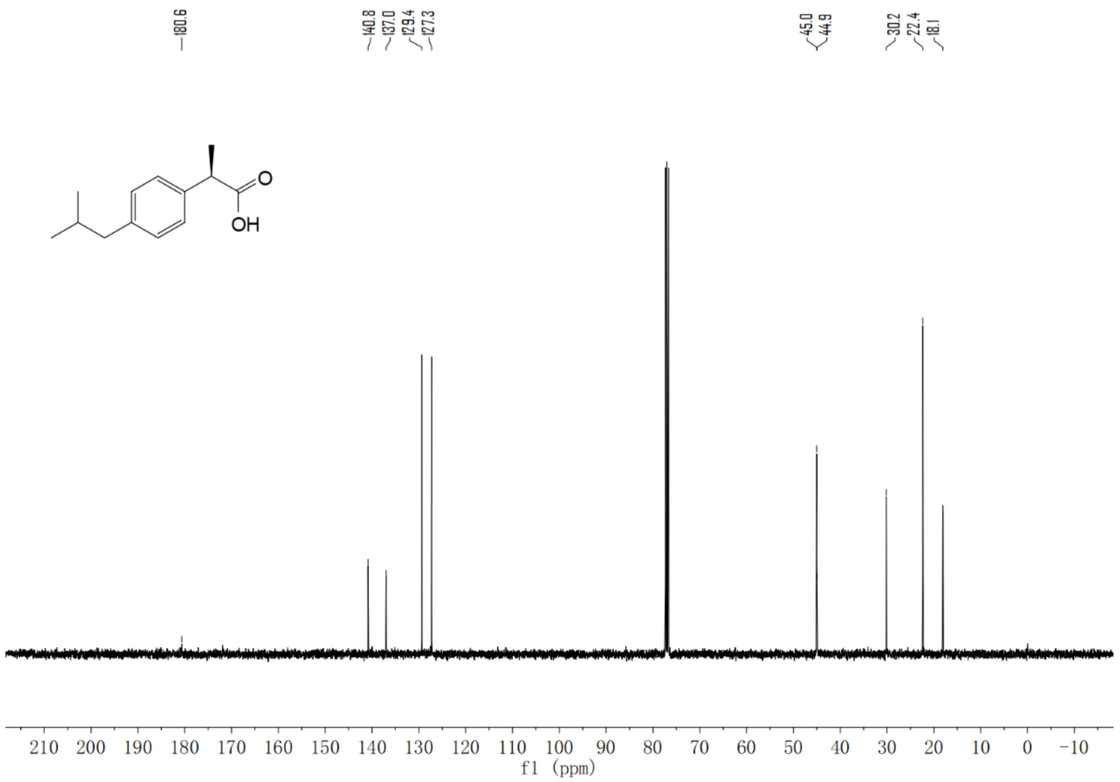

Supplementary Figure 88. ^{13}C NMR (101 MHz, $CDCl_3$) spectrum of **4k**

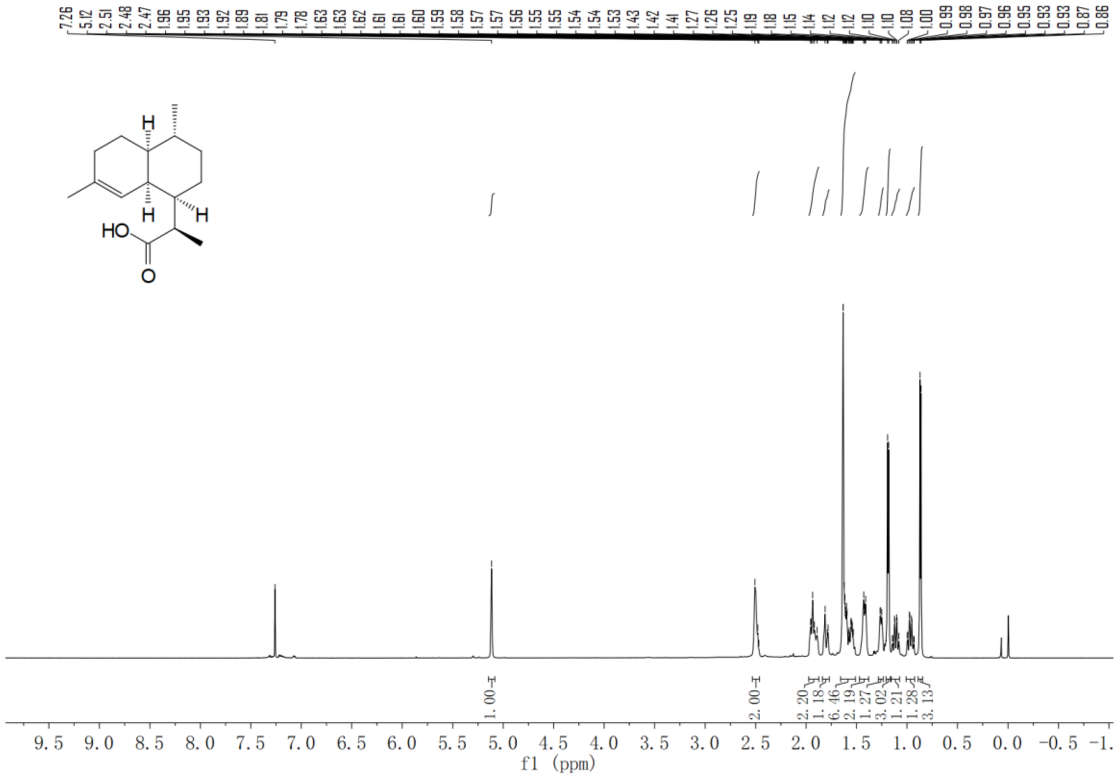

Supplementary Figure 89. ^{1}H NMR (400 MHz, $CDCl_3$) spectrum of **6**

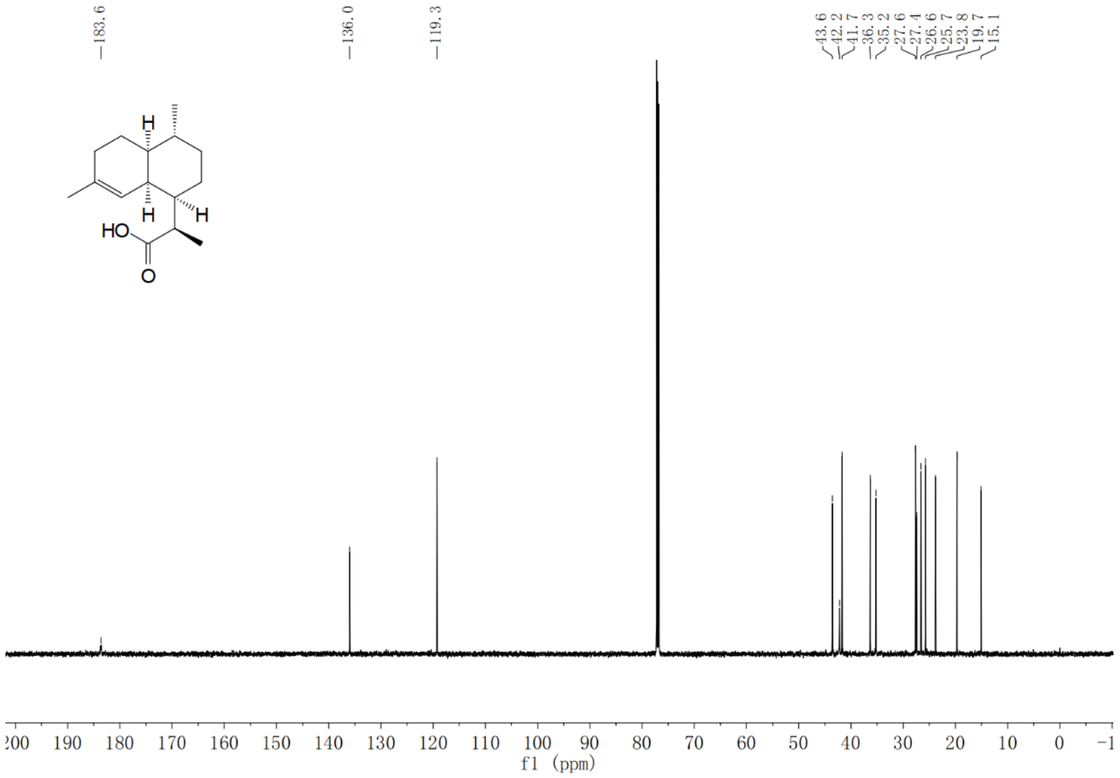

Supplementary Figure 90. ^{13}C NMR (101 MHz, $CDCl_3$) spectrum of **6**

Data File D:\CHEMSTA...TA\DXY\XY-1-76 77-1 2018.12.6 2018-12-06 12-19-03\relacation00002.D
Sample Name: xy-1-35-1-rac

===

Acq. Operator : SYSTEM Seq. Line : 2
Sample Operator : SYSTEM
Acq. Instrument : LC Location : P1-F-01
Injection Date : 12/6/2018 12:31:55 PM Inj : 1
Inj Volume : 1.000 µl
Acq. Method : D:\ChemStation\1\Data\DXY\xy-1-76 77-1 2018.12.6 2018-12-06 12-19-03\OJ3-97-3-0.8ML-60min.M
Last changed : 10/29/2018 10:29:50 AM by SYSTEM
Analysis Method : D:\ChemStation\1\Data\DXY\xy-1-76 77-1 2018.12.6 2018-12-06 12-19-03\OJ3-97-3-0.8ML-60min.M (Sequence Method)
Last changed : 10/23/2019 6:24:32 PM by SYSTEM
(modified after loading)
Additional Info : Peak(s) manually integrated

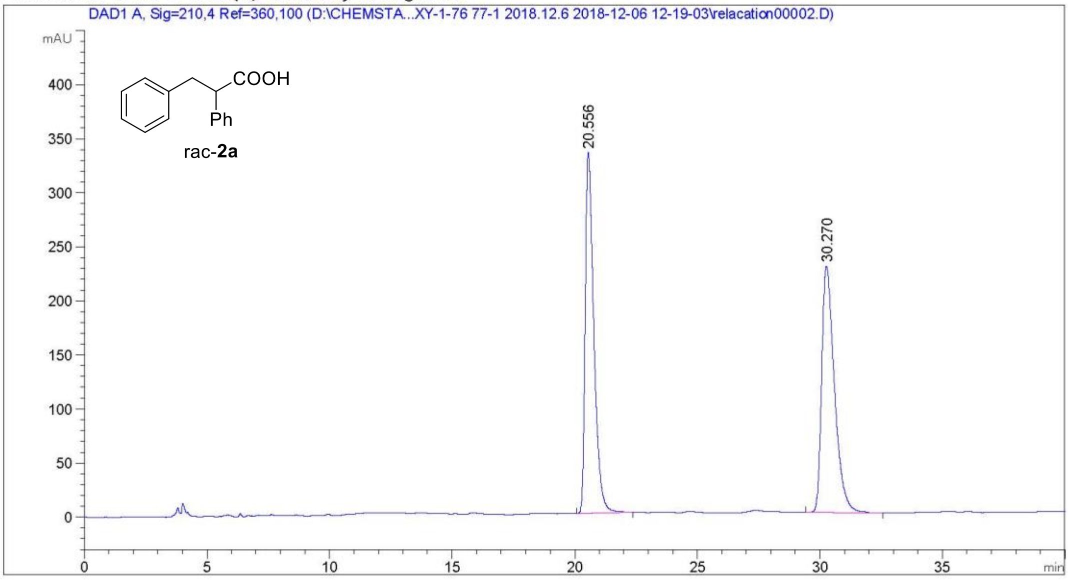

===

Area Percent Report

===

Sorted By : Signal
Multiplier : 1.0000
Dilution : 1.0000
Use Multiplier & Dilution Factor with ISTDs

Signal 1: DAD1 A, Sig=210,4 Ref=360,100

Peak #	RetTime [min]	Type	Width [min]	Area [mAU*s]	Height [mAU]	Area %
1	20.556	BB	0.3694	8160.38135	333.58524	49.7693
2	30.270	BB	0.5476	8236.02344	227.89218	50.2307

Totals : 1.63964e4 561.47742

Supplementary Figure 91. HPLC spectrum of rac-**2a**

Data File D:\CHEMSTA...TA\DXY\XY-1-76 77-1 2018.12.6 2018-12-06 12-19-03\relacation00003.D
Sample Name: xy-1-76-1

===

Acq. Operator : SYSTEM Seq. Line : 3
Sample Operator : SYSTEM
Acq. Instrument : LC Location : P1-F-02
Injection Date : 12/6/2018 1:32:49 PM Inj : 1
Inj Volume : 1.000 µl
Acq. Method : D:\ChemStation\1\Data\DXY\xy-1-76 77-1 2018.12.6 2018-12-06 12-19-03\OJ3-97-3-0.8ML-60min.M
Last changed : 10/29/2018 10:29:50 AM by SYSTEM
Analysis Method : D:\ChemStation\1\Data\DXY\xy-1-76 77-1 2018.12.6 2018-12-06 12-19-03\OJ3-97-3-0.8ML-60min.M (Sequence Method)
Last changed : 10/24/2019 4:09:16 PM by SYSTEM
(modified after loading)
Additional Info : Peak(s) manually integrated

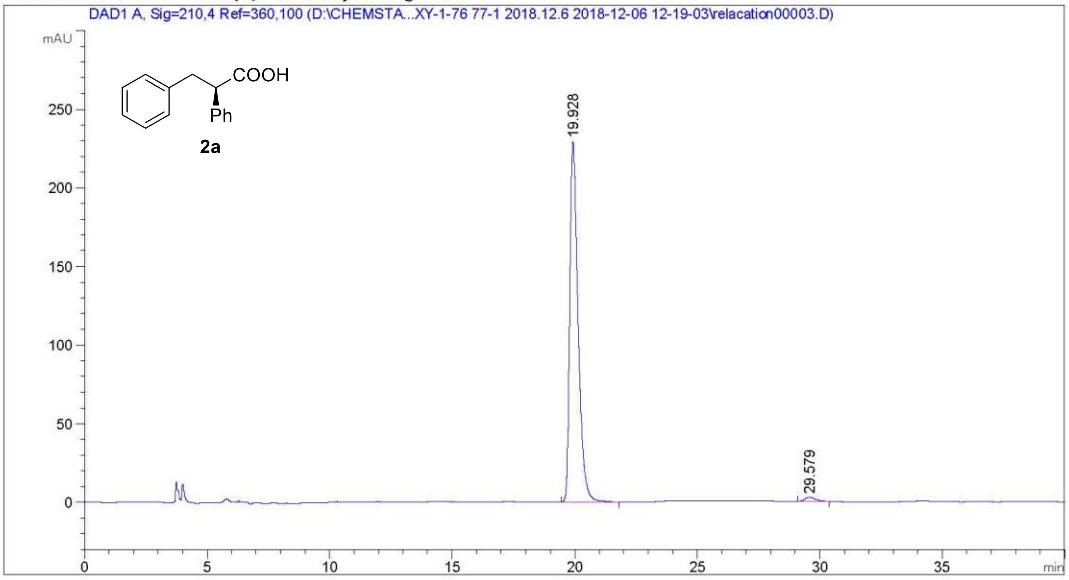

===

Area Percent Report

===

Sorted By : Signal
Multiplier : 1.0000
Dilution : 1.0000
Use Multiplier & Dilution Factor with ISTDs

Signal 1: DAD1 A, Sig=210,4 Ref=360,100

Peak #	RetTime [min]	Type	Width [min]	Area [mAU*s]	Height [mAU]	Area %
1	19.928	BB	0.3543	5389.30225	229.19775	98.4429
2	29.579	BB	0.4772	85.24332	2.70821	1.5571

Totals : 5474.54556 231.90596

Supplementary Figure 92. HPLC spectrum of **2a**

Data File D:\ChemSta...ta\DXY\xy-1-76 77-1 2018.12.6 2018-12-06 12-19-03\relacation00004.D
Sample Name: xy-1-77-1-rac

===

Acq. Operator : SYSTEM Seq. Line : 4
Sample Operator : SYSTEM
Acq. Instrument : LC Location : P1-F-03
Injection Date : 12/6/2018 2:33:43 PM Inj : 1
Inj Volume : 1.000 µl
Acq. Method : D:\ChemStation\1\Data\DXY\xy-1-76 77-1 2018.12.6 2018-12-06 12-19-03\OJ3-97-3-0.8ML-60min.M
Last changed : 10/29/2018 10:29:50 AM by SYSTEM
Analysis Method : D:\ChemStation\1\Data\DXY\xy-1-76 77-1 2018.12.6 2018-12-06 12-19-03\OJ3-97-3-0.8ML-60min.M (Sequence Method)
Last changed : 10/23/2019 6:31:57 PM by SYSTEM
(modified after loading)
Additional Info : Peak(s) manually integrated

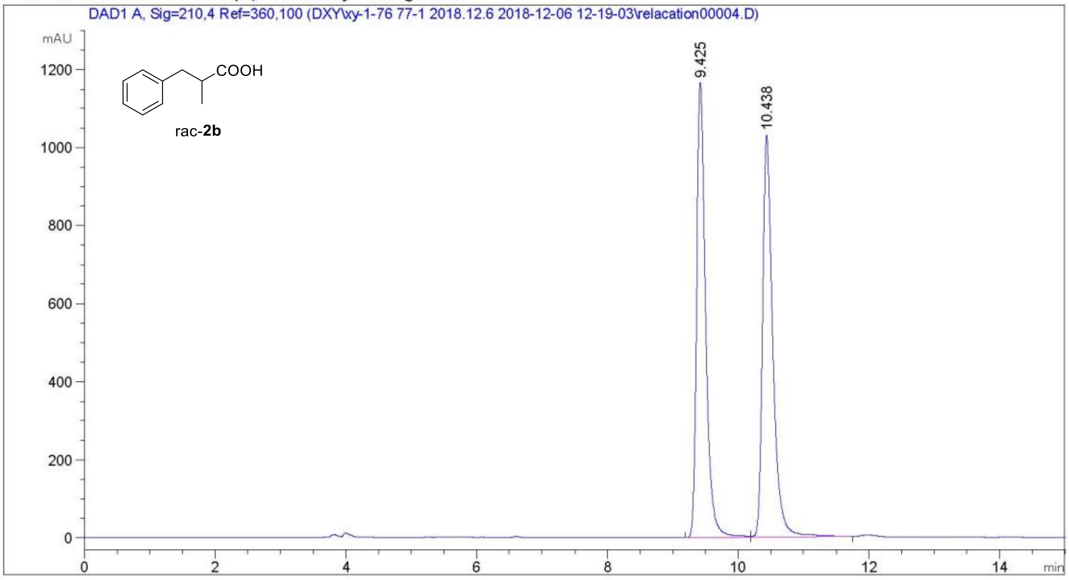

===

Area Percent Report

===

Sorted By : Signal
Multiplier : 1.0000
Dilution : 1.0000
Use Multiplier & Dilution Factor with ISTDs

Signal 1: DAD1 A, Sig=210,4 Ref=360,100

Peak #	RetTime [min]	Type	Width [min]	Area [mAU*s]	Height [mAU]	Area %
1	9.425	BV	0.1431	1.11229e4	1167.72766	49.5868
2	10.438	VB	0.1658	1.13083e4	1032.50208	50.4132

Totals : 2.24312e4 2200.22974

Supplementary Figure 93. HPLC spectrum of rac-**2b**

Data File D:\ChemSta...ta\DXY\xy-1-76 77-1 2018.12.6 2018-12-06 12-19-03\relacation00005.D
Sample Name: xy-1-77-1

===

Acq. Operator : SYSTEM Seq. Line : 5
Sample Operator : SYSTEM
Acq. Instrument : LC Location : P1-F-04
Injection Date : 12/6/2018 3:34:36 PM Inj : 1
Inj Volume : 1.000 µl
Acq. Method : D:\ChemStation\1\Data\DXY\xy-1-76 77-1 2018.12.6 2018-12-06 12-19-03\OJ3-97-3-0.8ML-60min.M
Last changed : 12/6/2018 4:21:42 PM by SYSTEM
(modified after loading)
Analysis Method : D:\ChemStation\1\Data\DXY\xy-1-76 77-1 2018.12.6 2018-12-06 12-19-03\OJ3-97-3-0.8ML-60min.M (Sequence Method)
Last changed : 10/23/2019 6:33:45 PM by SYSTEM
(modified after loading)
Additional Info : Peak(s) manually integrated

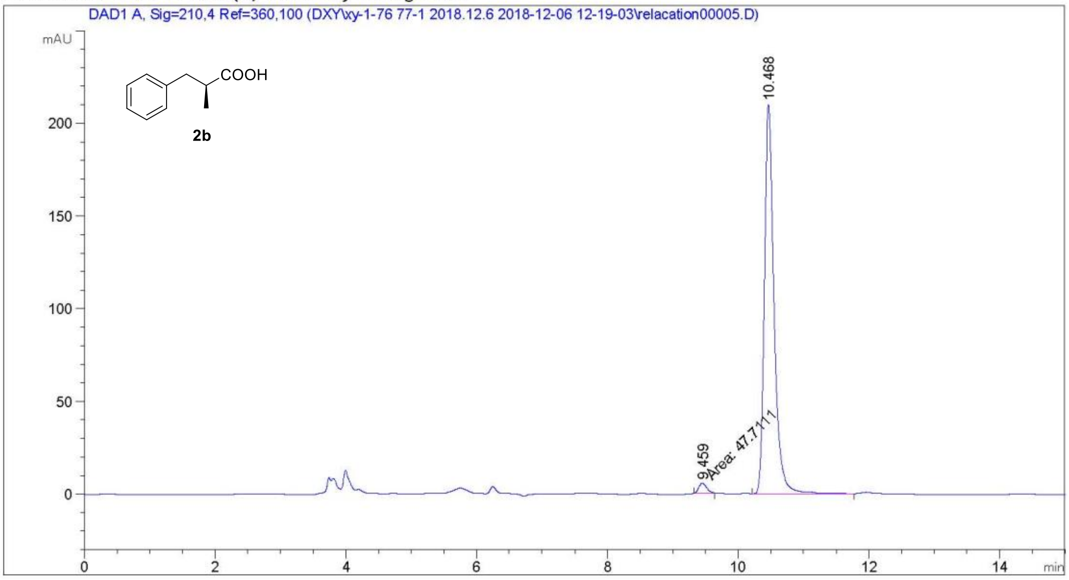

===

Area Percent Report

===

Sorted By : Signal
Multiplier : 1.0000
Dilution : 1.0000
Use Multiplier & Dilution Factor with ISTDs

Signal 1: DAD1 A, Sig=210,4 Ref=360,100

Peak #	RetTime [min]	Type	Width [min]	Area [mAU*s]	Height [mAU]	Area %
1	9.459	MM	0.1428	47.71107	5.56733	2.1558
2	10.468	BB	0.1562	2165.41870	210.10663	97.8442

Supplementary Figure 94. HPLC spectrum of **2b**

Data File D:\CHEM32\...2018.12.3 XY-1-73 74 75-1 2018-12-03 21-28-25\002-P1-F1-xy-1-80-1.D
Sample Name: xy-1-80-1

===

Acq. Operator : SYSTEM Seq. Line : 2
Acq. Instrument : 1260-DAD Location : P1-F-01
Injection Date : 12/3/2018 21:50:27 Inj : 1
Inj Volume : 5.000 µl
Acq. Method : d:\Chem32\1\Data\Dxy\2018.12.3 xy-1-73 74 75-1 2018-12-03 21-28-25\DXY-OJ-3-97-3-0.8ML-60MIN.M
Last changed : 12/3/2018 21:15:49 by SYSTEM
Analysis Method : d:\Chem32\1\Data\Dxy\2018.12.3 xy-1-73 74 75-1 2018-12-03 21-28-25\DXY-OJ-3-97-3-0.8ML-60MIN.M (Sequence Method)
Last changed : 12/18/2019 09:53:26 by SYSTEM
(modified after loading)
Additional Info : Peak(s) manually integrated

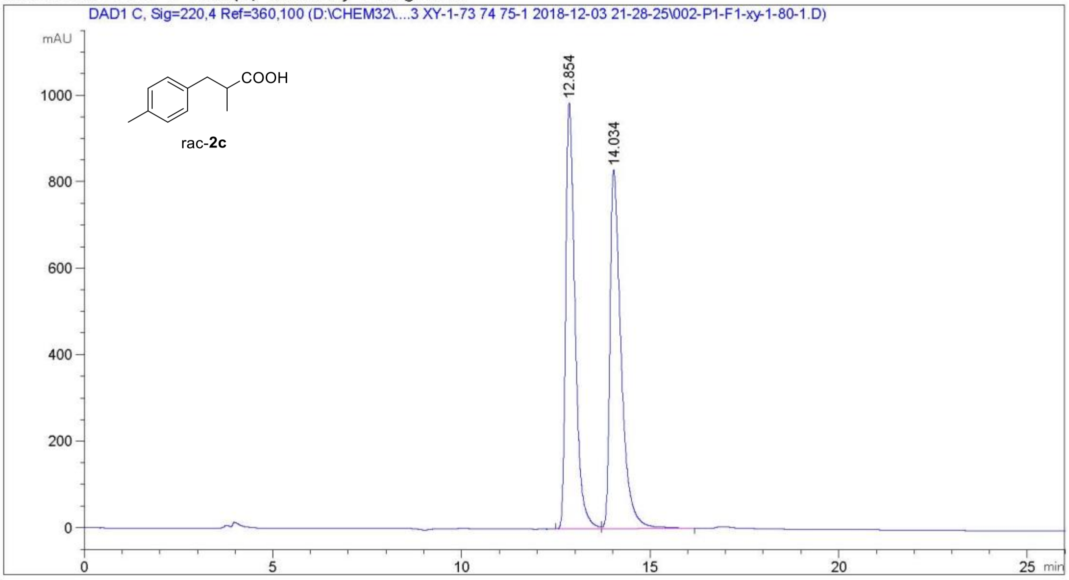

===

Area Percent Report

===

Sorted By : Signal
Multiplier : 1.0000
Dilution : 1.0000
Use Multiplier & Dilution Factor with ISTDs

Signal 1: DAD1 C, Sig=220,4 Ref=360,100

Peak #	RetTime [min]	Type	Width [min]	Area [mAU*s]	Height [mAU]	Area %
1	12.854	BV	0.2616	1.68539e4	985.19116	49.8329
2	14.034	VB	0.3066	1.69669e4	829.52667	50.1671
Totals :				3.38208e4	1814.71783	

Supplementary Figure 95. HPLC spectrum of rac-**2c**

Data File d:\Chem32\...2018.12.3 xy-1-73 74 75-1 2018-12-03 21-28-25\003-P1-F2-xy-1-73-1.D
Sample Name: xy-1-73-1

===

Acq. Operator	: SYSTEM	Seq. Line :	3
Acq. Instrument	: 1260-DAD	Location :	P1-F-02
Injection Date	: 12/3/2018 22:51:22	Inj :	1
		Inj Volume :	5.000 µl

Acq. Method : d:\Chem32\1\Data\Dxy\2018.12.3 xy-1-73 74 75-1 2018-12-03 21-28-25\DXY-OJ-3-97-3-0.8ML-60MIN.M
Last changed : 12/3/2018 21:15:49 by SYSTEM
Analysis Method : d:\Chem32\1\Data\Dxy\2018.12.3 xy-1-73 74 75-1 2018-12-03 21-28-25\DXY-OJ-3-97-3-0.8ML-60MIN.M (Sequence Method)
Last changed : 10/24/2019 15:57:18 by SYSTEM
(modified after loading)
Additional Info : Peak(s) manually integrated

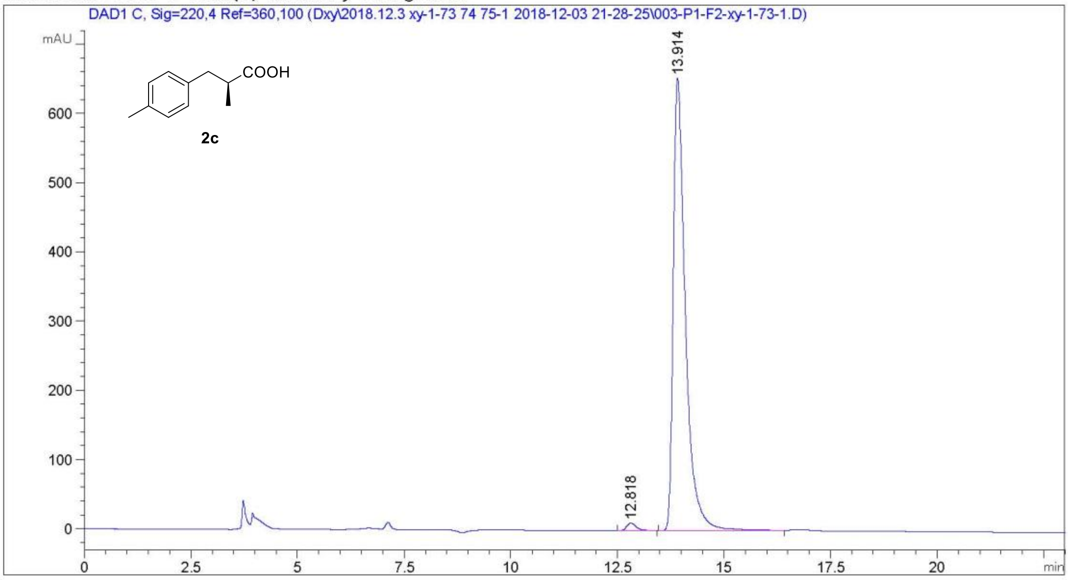

===
Area Percent Report
===

Sorted By : Signal
Multiplier : 1.0000
Dilution : 1.0000
Use Multiplier & Dilution Factor with ISTDs

Signal 1: DAD1 C, Sig=220,4 Ref=360,100

Peak #	RetTime [min]	Type	Width [min]	Area [mAU*s]	Height [mAU]	Area %
1	12.818	BB	0.2453	172.71449	10.87475	1.3199
2	13.914	BB	0.2943	1.29127e4	654.18231	98.6801

Totals : 1.30854e4 665.05706

Supplementary Figure 96. HPLC spectrum of **2c**

Data File d:\Chem32\...2018.12.3 xy-1-73 74 75-1 2018-12-03 21-28-25\004-P1-F3-xy-1-80-2.D
Sample Name: xy-1-80-2

===

Acq. Operator : SYSTEM　　Seq. Line : 4
Acq. Instrument : 1260-DAD　　Location : P1-F-03
Injection Date : 12/3/2018 23:52:17　　Inj : 1
Inj Volume : 5.000 µl
Acq. Method : d:\Chem32\1\Data\Dxy\2018.12.3 xy-1-73 74 75-1 2018-12-03 21-28-25\DXY-OJ-3-97-3-0.8ML-60MIN.M
Last changed : 12/3/2018 21:15:49 by SYSTEM
Analysis Method : d:\Chem32\1\Data\Dxy\2018.12.3 xy-1-73 74 75-1 2018-12-03 21-28-25\DXY-OJ-3-97-3-0.8ML-60MIN.M (Sequence Method)
Last changed : 10/23/2019 18:55:49 by SYSTEM
(modified after loading)
Additional Info : Peak(s) manually integrated

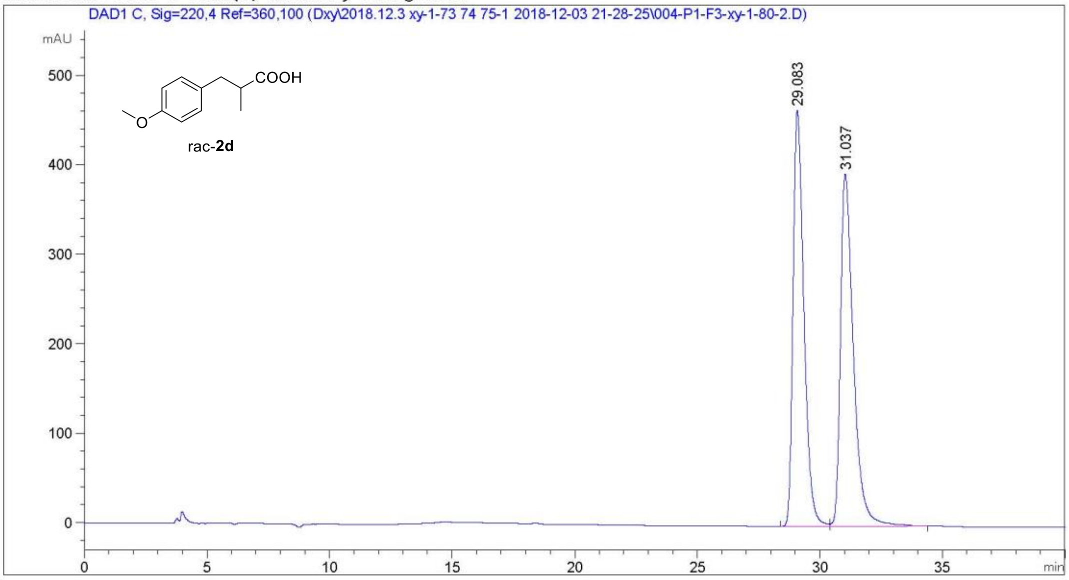

===

Area Percent Report

===

Sorted By : Signal
Multiplier : 1.0000
Dilution : 1.0000
Use Multiplier & Dilution Factor with ISTDs

Signal 1: DAD1 C, Sig=220,4 Ref=360,100

Peak #	RetTime [min]	Type	Width [min]	Area [mAU*s]	Height [mAU]	Area %
1	29.083	BV	0.4702	1.42745e4	464.97992	49.7482
2	31.037	VB	0.5534	1.44190e4	393.57562	50.2518

Totals : 2.86935e4 858.55554

Supplementary Figure 97. HPLC spectrum of rac-**2d**

Data File D:\CHEM32\...2018.12.3 XY-1-73 74 75-1 2018-12-03 21-28-25\005-P1-F4-xy-1-74-1.D
Sample Name: xy-1-74-1

===

```
Acq. Operator   : SYSTEM                        Seq. Line :   5
Acq. Instrument : 1260-DAD                       Location :   P1-F-04
Injection Date  : 12/4/2018 00:53:11                  Inj :   1
                                               Inj Volume : 5.000 µl
Acq. Method     : d:\Chem32\1\Data\Dxy\2018.12.3 xy-1-73 74 75-1 2018-12-03 21-28-25\DXY-OJ-3
                  -97-3-0.8ML-60MIN.M
Last changed    : 12/3/2018 21:15:49 by SYSTEM
Analysis Method : d:\Chem32\1\Data\Dxy\2018.12.3 xy-1-73 74 75-1 2018-12-03 21-28-25\DXY-OJ-3
                  -97-3-0.8ML-60MIN.M (Sequence Method)
Last changed    : 10/24/2019 15:52:03 by SYSTEM
                  (modified after loading)
Additional Info : Peak(s) manually integrated
```

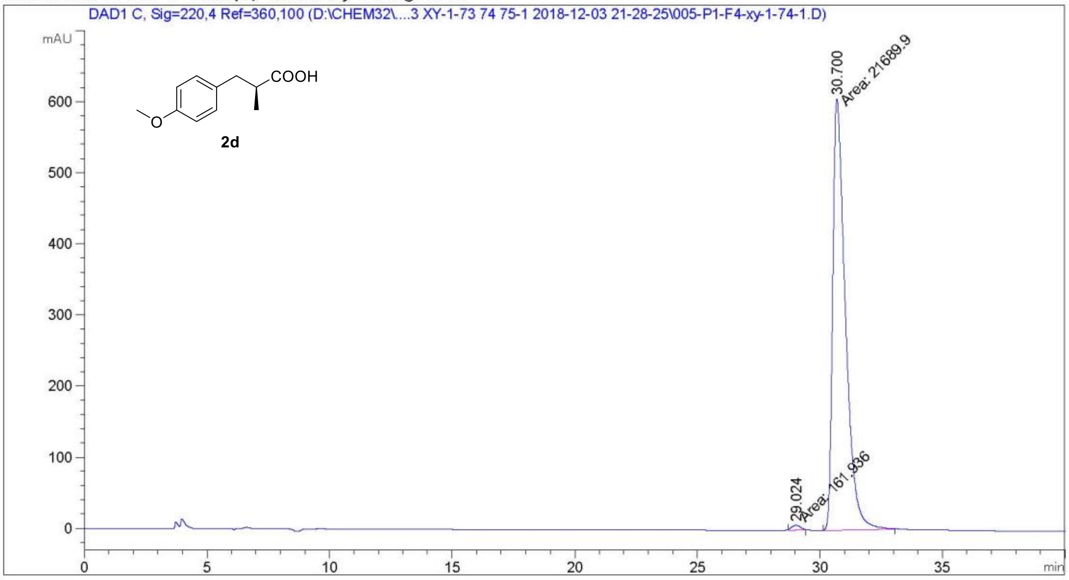

===

Area Percent Report

===

```
Sorted By             :      Signal
Multiplier            :      1.0000
Dilution              :      1.0000
Use Multiplier & Dilution Factor with ISTDs
```

Signal 1: DAD1 C, Sig=220,4 Ref=360,100

Peak #	RetTime [min]	Type	Width [min]	Area [mAU*s]	Height [mAU]	Area %
1	29.024	MM	0.4021	161.93555	6.71287	0.7411
2	30.700	MM	0.5961	2.16899e4	606.44678	99.2589

Totals : 2.18518e4 613.15965

Supplementary Figure 98. HPLC spectrum of **2d**

Data File D:\CHEMSTA...05 106-1 DXY-1-104-1 2019.1.2 2019-01-02 22-25-36\relacation00005.D
Sample Name: xy-1-100-1

===

Acq. Operator : SYSTEM Seq. Line : 5
Sample Operator : SYSTEM
Acq. Instrument : LC Location : P1-D-03
Injection Date : 1/2/2019 11:49:03 PM Inj : 1
Inj Volume : 1.000 µl
Acq. Method : D:\ChemStation\1\Data\DXY\xy-1-94 105 106-1 dxy-1-104-1 2019.1.2 2019-01-02 22-25-36\OJ3-97-3-0.8ML-55min.M
Last changed : 10/30/2018 4:44:46 PM by SYSTEM
Analysis Method : D:\ChemStation\1\Data\DXY\xy-1-94 105 106-1 dxy-1-104-1 2019.1.2 2019-01-02 22-25-36\OJ3-97-3-0.8ML-55min.M (Sequence Method)
Last changed : 10/24/2019 3:00:22 PM by SYSTEM
(modified after loading)
Additional Info : Peak(s) manually integrated

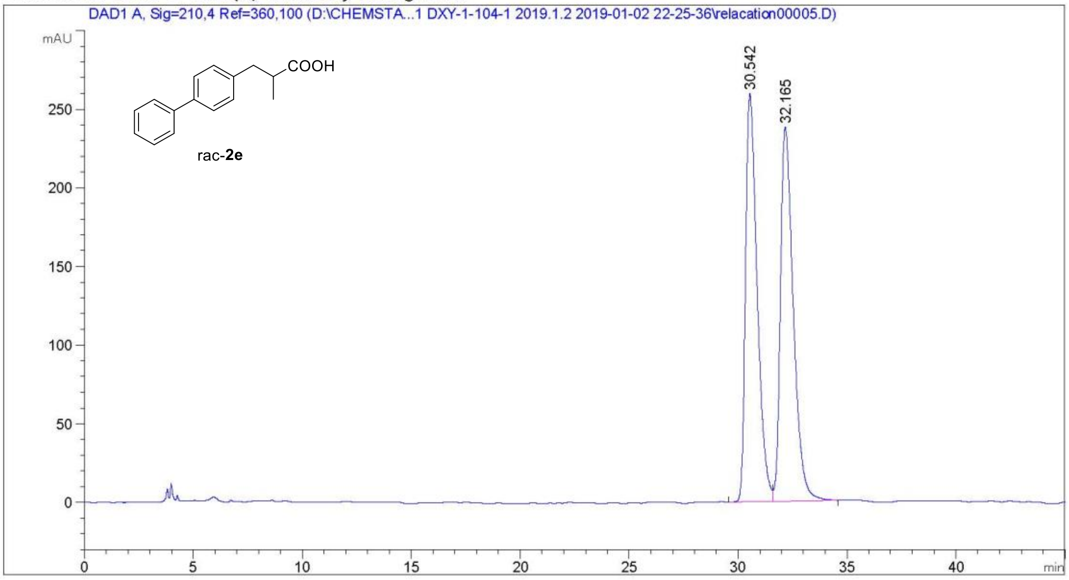

===

Area Percent Report

===

Sorted By : Signal
Multiplier : 1.0000
Dilution : 1.0000
Use Multiplier & Dilution Factor with ISTDs

Signal 1: DAD1 A, Sig=210,4 Ref=360,100

Peak #	RetTime [min]	Type	Width [min]	Area [mAU*s]	Height [mAU]	Area %
1	30.542	BV	0.5650	9580.72461	259.33481	49.5705
2	32.165	VB	0.6224	9746.74414	237.68053	50.4295

Totals : 1.93275e4 497.01534

Supplementary Figure 99. HPLC spectrum of rac-**2e**

Data File D:\ChemSta...05 106-1 dxy-1-104-1 2019.1.2 2019-01-02 22-25-36\relacation00006.D
Sample Name: xy-1-104-1

===

Acq. Operator : SYSTEM Seq. Line : 6
Sample Operator : SYSTEM
Acq. Instrument : LC Location : P1-D-04
Injection Date : 1/3/2019 12:44:57 AM Inj : 1
Inj Volume : 1.000 µl
Acq. Method : D:\ChemStation\1\Data\DXY\xy-1-94 105 106-1 dxy-1-104-1 2019.1.2 2019-01-02 22-25-36\OJ3-97-3-0.8ML-55min.M
Last changed : 10/30/2018 4:44:46 PM by SYSTEM
Analysis Method : D:\ChemStation\1\Data\DXY\xy-1-94 105 106-1 dxy-1-104-1 2019.1.2 2019-01-02 22-25-36\OJ3-97-3-0.8ML-55min.M (Sequence Method)
Last changed : 10/24/2019 3:01:25 PM by SYSTEM
(modified after loading)
Additional Info : Peak(s) manually integrated

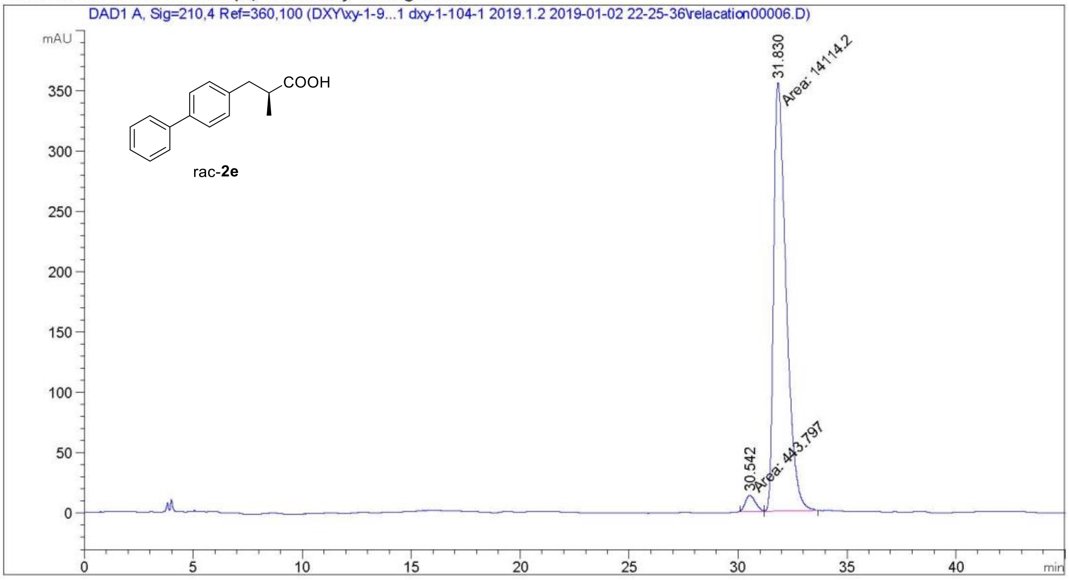

===

Area Percent Report

===

Sorted By : Signal
Multiplier : 1.0000
Dilution : 1.0000
Use Multiplier & Dilution Factor with ISTDs

Signal 1: DAD1 A, Sig=210,4 Ref=360,100

Peak #	RetTime [min]	Type	Width [min]	Area [mAU*s]	Height [mAU]	Area %
1	30.542	MF	0.5473	443.79681	13.51531	3.0485
2	31.830	FM	0.6626	1.41142e4	355.01944	96.9515

Totals : 1.45580e4 368.53475

Supplementary Figure 100. HPLC spectrum of **2e**

Data File D:\CHEMSTA...1\DATA\DXY\XY-1-75 2019.10.27 2019-10-27 16-54-21\relacation00002.D
Sample Name: xy-1-80-3

===

Acq. Operator : SYSTEM Seq. Line : 2
Sample Operator : SYSTEM
Acq. Instrument : LC Location : P1-A-02
Injection Date : 10/27/2019 5:15:50 PM Inj : 1
Inj Volume : 1.000 µl
Acq. Method : D:\ChemStation\1\Data\DXY\xy-1-75 2019.10.27 2019-10-27 16-54-21\OJ3-97-3-0.8ML-55min.M
Last changed : 10/30/2018 4:44:46 PM by SYSTEM
Analysis Method : D:\ChemStation\1\Data\DXY\xy-1-75 2019.10.27 2019-10-27 16-54-21\OJ3-97-3-0.8ML-55min.M (Sequence Method)
Last changed : 10/29/2019 11:15:34 AM by SYSTEM
(modified after loading)
Additional Info : Peak(s) manually integrated

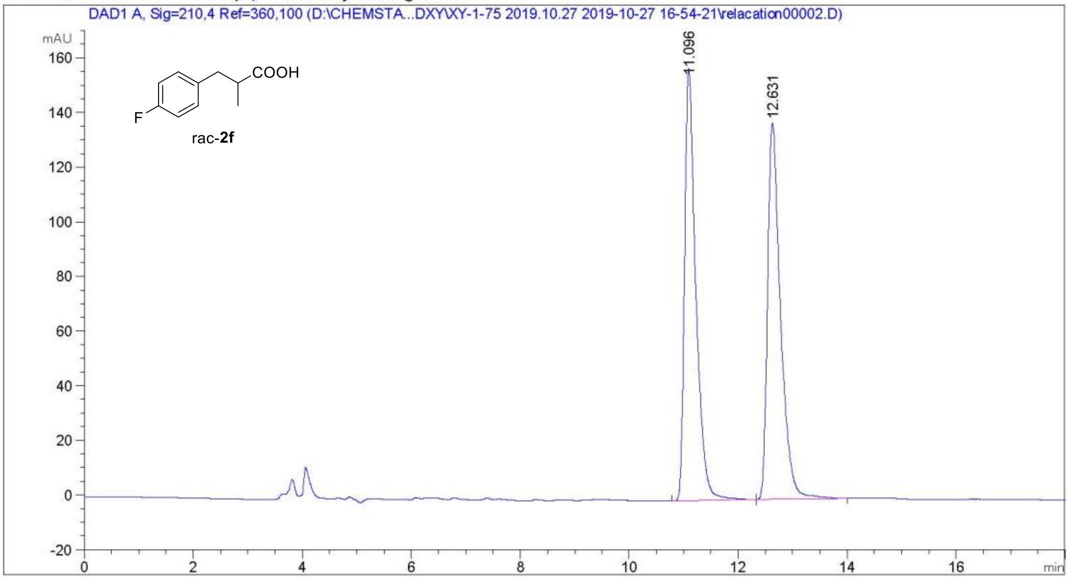

===

Area Percent Report

===

Sorted By : Signal
Multiplier : 1.0000
Dilution : 1.0000
Use Multiplier & Dilution Factor with ISTDs

Signal 1: DAD1 A, Sig=210,4 Ref=360,100

Peak #	RetTime [min]	Type	Width [min]	Area [mAU*s]	Height [mAU]	Area %
1	11.096	BB	0.2106	2260.20361	158.24481	49.9690
2	12.631	BB	0.2415	2263.00439	137.84248	50.0310

Totals : 4523.20801 296.08730

Supplementary Figure 101. HPLC spectrum of rac-**2f**

Data File D:\ChemSta...1\Data\DXY\xy-1-75 2019.10.27 2019-10-27 16-54-21\relacation00003.D
Sample Name: xy-1-75-1

===

Acq. Operator : SYSTEM Seq. Line : 3
Sample Operator : SYSTEM
Acq. Instrument : LC Location : P1-A-03
Injection Date : 10/27/2019 6:11:41 PM Inj : 1
Inj Volume : 1.000 µl
Acq. Method : D:\ChemStation\1\Data\DXY\xy-1-75 2019.10.27 2019-10-27 16-54-21\OJ3-97-3-0.8ML-55min.M
Last changed : 10/30/2018 4:44:46 PM by SYSTEM
Analysis Method : D:\ChemStation\1\Data\DXY\xy-1-75 2019.10.27 2019-10-27 16-54-21\OJ3-97-3-0.8ML-55min.M (Sequence Method)
Last changed : 10/29/2019 11:17:29 AM by SYSTEM
(modified after loading)
Additional Info : Peak(s) manually integrated

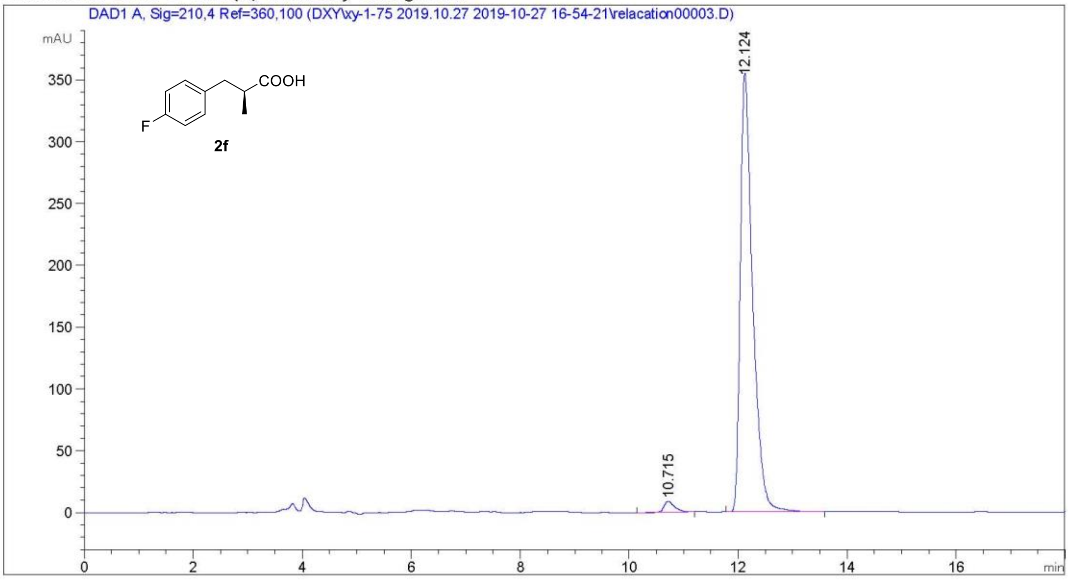

===

Area Percent Report

===

Sorted By : Signal
Multiplier : 1.0000
Dilution : 1.0000
Use Multiplier & Dilution Factor with ISTDs

Signal 1: DAD1 A, Sig=210,4 Ref=360,100

Peak #	RetTime [min]	Type	Width [min]	Area [mAU*s]	Height [mAU]	Area %
1	10.715	BB	0.1973	122.17621	8.95044	2.1432
2	12.124	BB	0.2313	5578.50928	355.07358	97.8568

Totals : 5700.68549 364.02402

Supplementary Figure 102. HPLC spectrum of **2f**

Data File D:\ChemSta...05 106-1 dxy-1-104-1 2019.1.2 2019-01-02 22-25-36\relacation00003.D
Sample Name: xy-1-99-1

===

Acq. Operator : SYSTEM Seq. Line : 3
Sample Operator : SYSTEM
Acq. Instrument : LC Location : P1-D-01
Injection Date : 1/2/2019 10:47:15 PM Inj : 1
Inj Volume : 1.000 µl
Acq. Method : D:\ChemStation\1\Data\DXY\xy-1-94 105 106-1 dxy-1-104-1 2019.1.2 2019-01-02 22-25-36\OJ3-97-3-0.8ML-30min.M
Last changed : 1/2/2019 10:18:19 PM by SYSTEM
Analysis Method : D:\ChemStation\1\Data\DXY\xy-1-94 105 106-1 dxy-1-104-1 2019.1.2 2019-01-02 22-25-36\OJ3-97-3-0.8ML-30min.M (Sequence Method)
Last changed : 10/23/2019 7:00:16 PM by SYSTEM
(modified after loading)
Additional Info : Peak(s) manually integrated

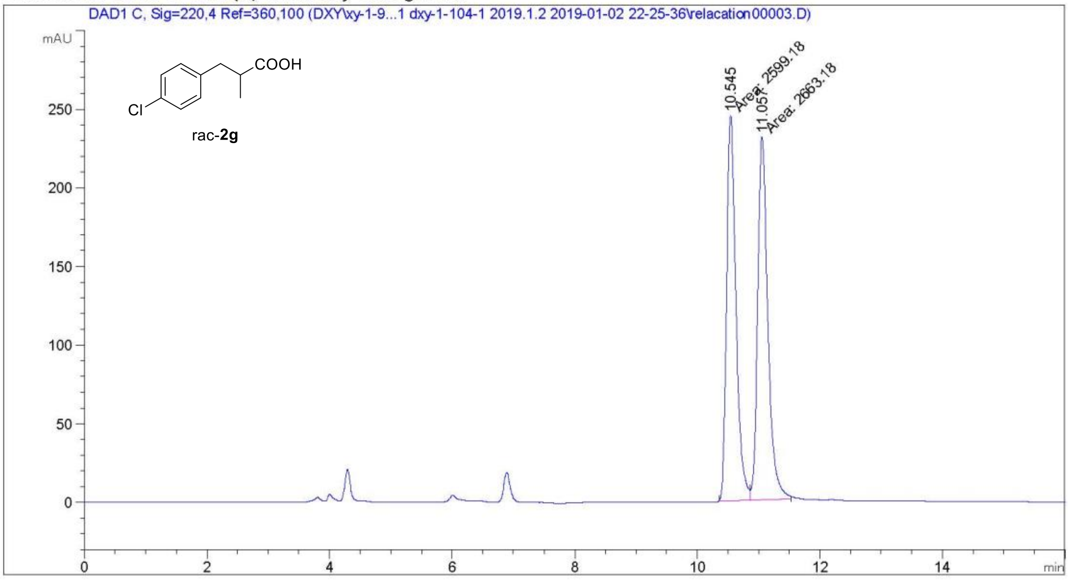

===

Area Percent Report

===

Sorted By : Signal
Multiplier : 1.0000
Dilution : 1.0000
Use Multiplier & Dilution Factor with ISTDs

Signal 1: DAD1 C, Sig=220,4 Ref=360,100

Peak #	RetTime [min]	Type	Width [min]	Area [mAU*s]	Height [mAU]	Area %
1	10.545	MF	0.1767	2599.17993	245.21254	49.3919
2	11.057	FM	0.1921	2663.17896	231.01855	50.6081

Totals : 5262.35889 476.23109

Supplementary Figure 103. HPLC spectrum of rac-**2g**

Data File D:\CHEMSTA...05 106-1 DXY-1-104-1 2019.1.2 2019-01-02 22-25-36\relacation00004.D
Sample Name: xy-1-94-1

===

Acq. Operator : SYSTEM Seq. Line : 4
Sample Operator : SYSTEM
Acq. Instrument : LC Location : P1-D-02
Injection Date : 1/2/2019 11:18:07 PM Inj : 1
Inj Volume : 1.000 µl
Acq. Method : D:\ChemStation\1\Data\DXY\xy-1-94 105 106-1 dxy-1-104-1 2019.1.2 2019-01-02 22-25-36\OJ3-97-3-0.8ML-30min.M
Last changed : 1/2/2019 10:18:19 PM by SYSTEM
Analysis Method : D:\ChemStation\1\Data\DXY\xy-1-94 105 106-1 dxy-1-104-1 2019.1.2 2019-01-02 22-25-36\OJ3-97-3-0.8ML-30min.M (Sequence Method)
Last changed : 10/23/2019 6:58:11 PM by SYSTEM
(modified after loading)
Additional Info : Peak(s) manually integrated

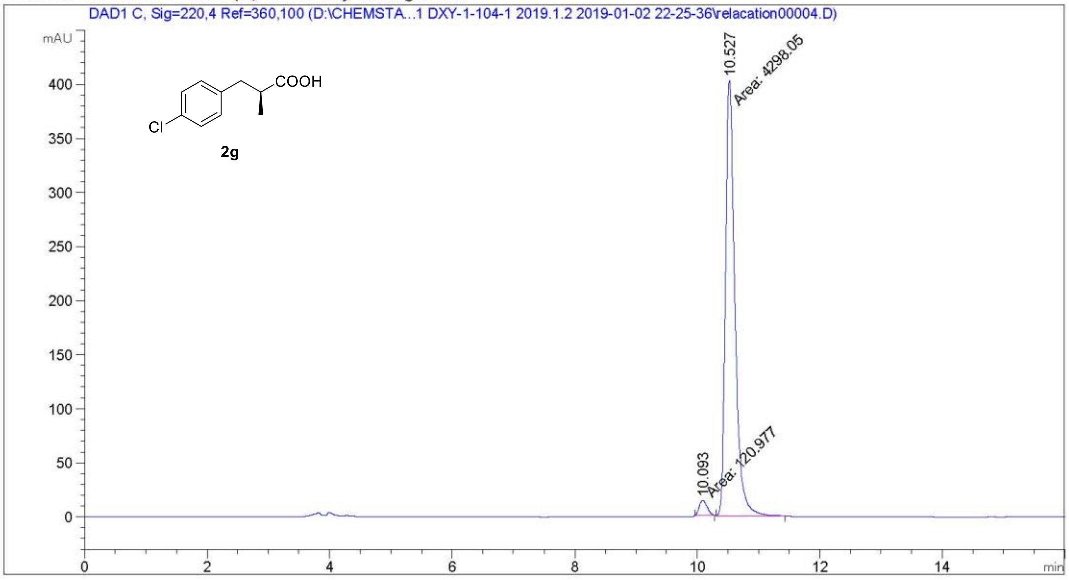

===

Area Percent Report

===

Sorted By : Signal
Multiplier : 1.0000
Dilution : 1.0000
Use Multiplier & Dilution Factor with ISTDs

Signal 1: DAD1 C, Sig=220,4 Ref=360,100

Peak #	RetTime [min]	Type	Width [min]	Area [mAU*s]	Height [mAU]	Area %
1	10.093	MM	0.1467	120.97746	13.74354	2.7376
2	10.527	MM	0.1778	4298.05371	402.96558	97.2624

Totals : 4419.03117 416.70911

Supplementary Figure 104. HPLC spectrum of **2g**

Data File D:\CHEMSTA...83 84 85 86-1 2018.12.16 2018-12-16 19-28-49\relacation00008--009.D
Sample Name: xy-1-87-1

===

Acq. Operator : SYSTEM   Seq. Line : 9
Sample Operator : SYSTEM
Acq. Instrument : LC   Location : P1-F-05
Injection Date : 12/17/2018 12:04:53 AM   Inj : 1
Inj Volume : 1.000 µl
Acq. Method : D:\ChemStation\1\Data\DXY\xy-1-83 84 85 86-1 2018.12.16 2018-12-16 19-28-49 \OJ3-98-2-0.5ML-60min.M
Last changed : 12/16/2018 2:12:34 PM by SYSTEM
Analysis Method : D:\ChemStation\1\Data\DXY\xy-1-83 84 85 86-1 2018.12.16 2018-12-16 19-28-49 \OJ3-98-2-0.5ML-60min.M (Sequence Method)
Last changed : 10/23/2019 7:11:02 PM by SYSTEM
(modified after loading)
Additional Info : Peak(s) manually integrated

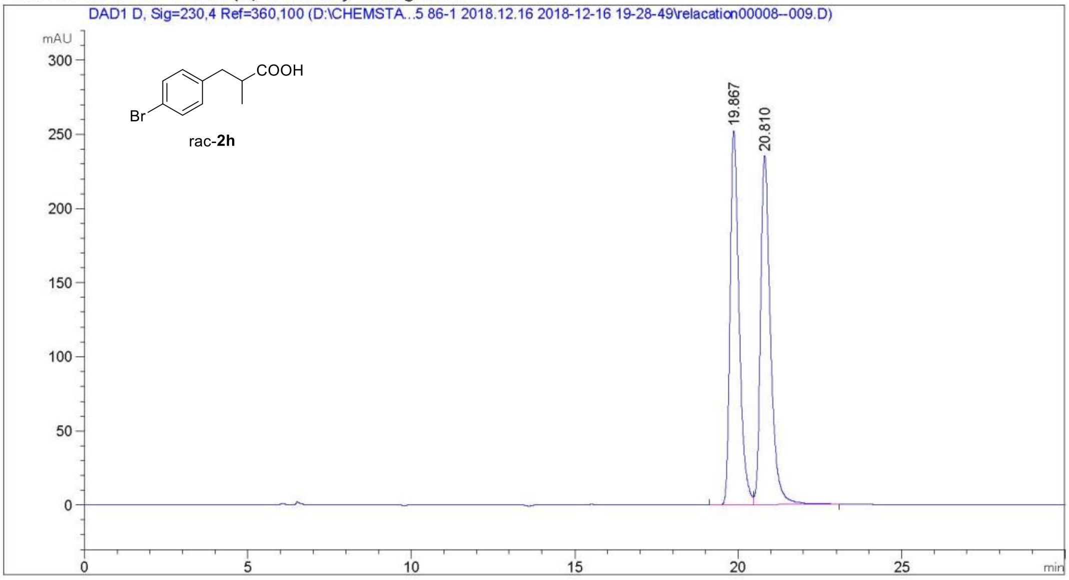

===

Area Percent Report

===

Sorted By : Signal
Multiplier : 1.0000
Dilution : 1.0000
Use Multiplier & Dilution Factor with ISTDs

Signal 1: DAD1 D, Sig=230,4 Ref=360,100

Peak #	RetTime [min]	Type	Width [min]	Area [mAU*s]	Height [mAU]	Area %
1	19.867	BV	0.2916	4834.78955	252.30565	49.2861
2	20.810	VB	0.3209	4974.85303	235.06357	50.7139

Totals : 9809.64258 487.36922

Supplementary Figure 105. HPLC spectrum of rac-**2h**

```
Data File D:\ChemSta...83 84 85 86-1 2018.12.16 2018-12-16 19-28-49\relacation00009--010.D
Sample Name: xy-1-83-1

=====================================================================
Acq. Operator   : SYSTEM                          Seq. Line :  10
Sample Operator : SYSTEM
Acq. Instrument : LC                               Location :   P1-F-06
Injection Date  : 12/17/2018 1:05:47 AM                 Inj :   1
                                              Inj Volume : 1.000 µl
Acq. Method     : D:\ChemStation\1\Data\DXY\xy-1-83 84 85 86-1 2018.12.16 2018-12-16 19-28-49
                  \OJ3-98-2-0.5ML-60min.M
Last changed    : 12/16/2018 2:12:34 PM by SYSTEM
Analysis Method : D:\ChemStation\1\Data\DXY\xy-1-83 84 85 86-1 2018.12.16 2018-12-16 19-28-49
                  \OJ3-98-2-0.5ML-60min.M (Sequence Method)
Last changed    : 10/23/2019 7:11:55 PM by SYSTEM
                  (modified after loading)
Additional Info : Peak(s) manually integrated
```

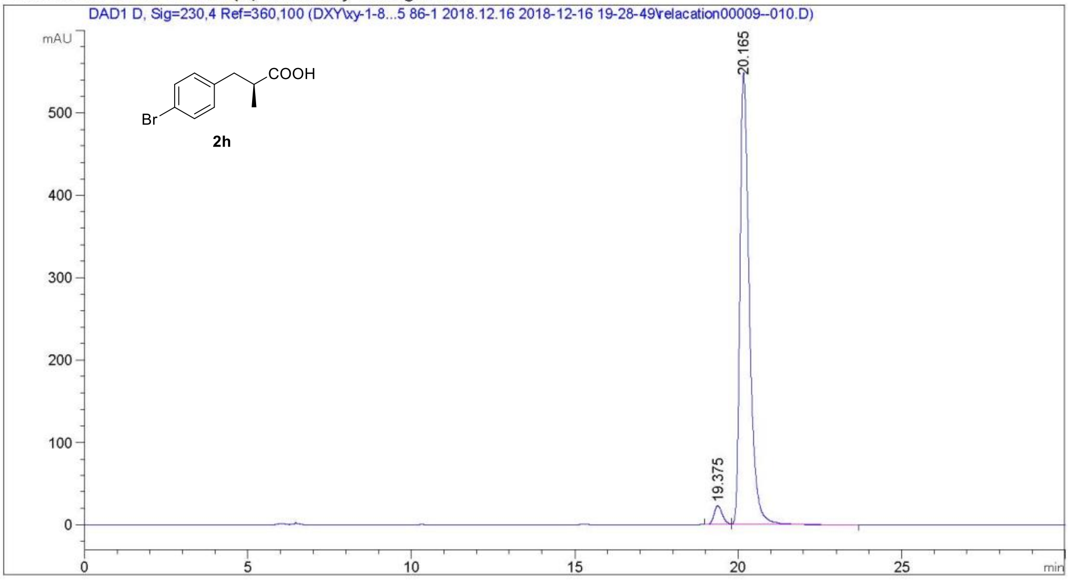


```
=====================================================================
                         Area Percent Report
=====================================================================

Sorted By             :      Signal
Multiplier            :      1.0000
Dilution              :      1.0000
Use Multiplier & Dilution Factor with ISTDs


Signal 1: DAD1 D, Sig=230,4 Ref=360,100
```

Peak #	RetTime [min]	Type	Width [min]	Area [mAU*s]	Height [mAU]	Area %
1	19.375	BV	0.2691	399.62656	22.73603	3.5119
2	20.165	VB	0.3035	1.09797e4	548.52478	96.4881

Totals : 1.13793e4 571.26081

Supplementary Figure 106. HPLC spectrum of **2h**

```
Data File D:\CHEMSTA...83 84 85 86-1 2018.12.16 2018-12-16 19-28-49\relacation00010--011.D
Sample Name: xy-1-88-1

    =====================================================================
    Acq. Operator   : SYSTEM                       Seq. Line :  11
    Sample Operator : SYSTEM
    Acq. Instrument : LC                            Location :   P1-F-07
    Injection Date  : 12/17/2018 2:06:41 AM              Inj :   1
                                                  Inj Volume : 1.000 µl
    Acq. Method     : D:\ChemStation\1\Data\DXY\xy-1-83 84 85 86-1 2018.12.16 2018-12-16 19-28-49
                      \OJ3-98-2-0.5ML-60min.M
    Last changed    : 12/16/2018 2:12:34 PM by SYSTEM
    Analysis Method : D:\ChemStation\1\Data\DXY\xy-1-83 84 85 86-1 2018.12.16 2018-12-16 19-28-49
                      \OJ3-98-2-0.5ML-60min.M (Sequence Method)
    Last changed    : 10/23/2019 7:18:24 PM by SYSTEM
                      (modified after loading)
    Additional Info : Peak(s) manually integrated
```

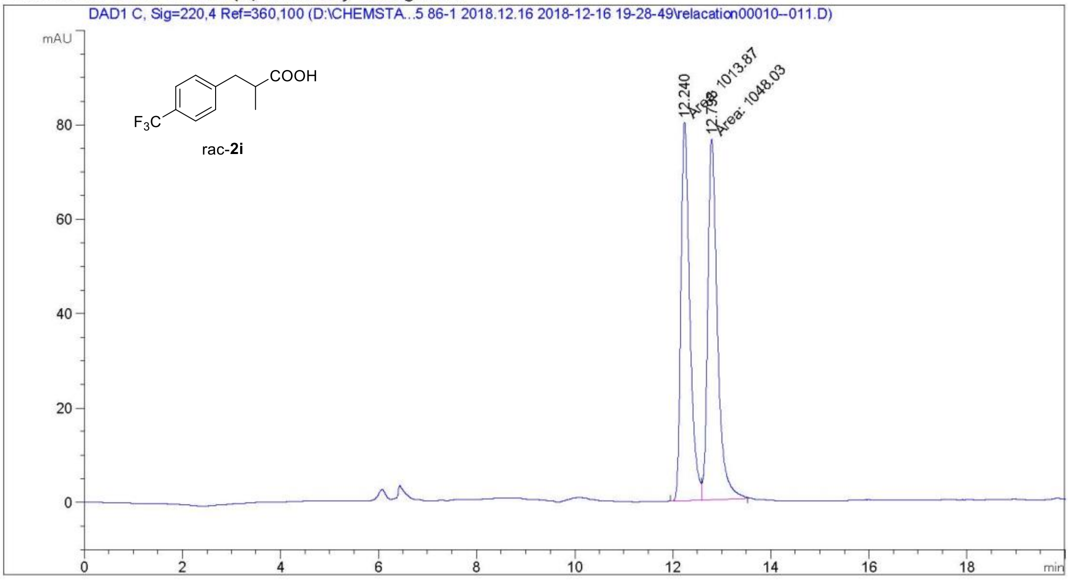


```
    =====================================================================
                             Area Percent Report
    =====================================================================

    Sorted By             :      Signal
    Multiplier            :      1.0000
    Dilution              :      1.0000
    Use Multiplier & Dilution Factor with ISTDs


    Signal 1: DAD1 C, Sig=220,4 Ref=360,100

    Peak RetTime Type  Width     Area      Height     Area
      #   [min]        [min]   [mAU*s]     [mAU]        %
    ----|-------|----|-------|----------|----------|--------|
       1  12.240 MF    0.2104 1013.87378   80.29807  49.1718
       2  12.793 FM    0.2284 1048.02820   76.46355  50.8282

    Totals :                  2061.90198  156.76163
```


Supplementary Figure 107. HPLC spectrum of rac-**2i**

Data File D:\ChemSta...83 84 85 86-1 2018.12.16 2018-12-16 19-28-49\relacation00011--012.D
Sample Name: xy-1-84-1

===

Acq. Operator : SYSTEM Seq. Line : 12
Sample Operator : SYSTEM
Acq. Instrument : LC Location : P1-F-08
Injection Date : 12/17/2018 3:07:36 AM Inj : 1
Inj Volume : 1.000 µl
Acq. Method : D:\ChemStation\1\Data\DXY\xy-1-83 84 85 86-1 2018.12.16 2018-12-16 19-28-49 \OJ3-98-2-0.5ML-60min.M
Last changed : 12/16/2018 2:12:34 PM by SYSTEM
Analysis Method : D:\ChemStation\1\Data\DXY\xy-1-83 84 85 86-1 2018.12.16 2018-12-16 19-28-49 \OJ3-98-2-0.5ML-60min.M (Sequence Method)
Last changed : 10/23/2019 7:20:52 PM by SYSTEM
(modified after loading)
Additional Info : Peak(s) manually integrated

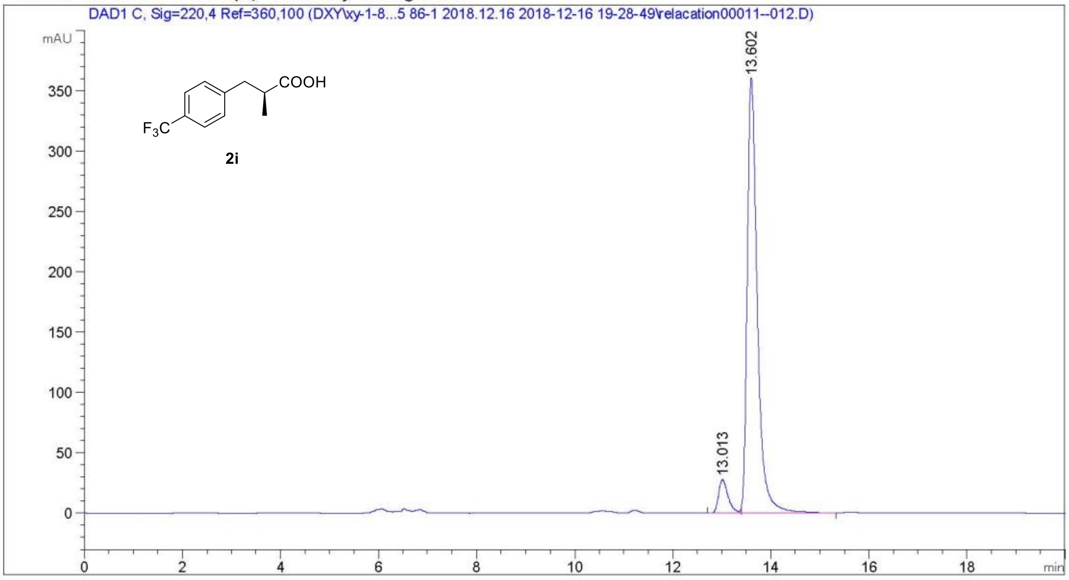

===

Area Percent Report

===

Sorted By : Signal
Multiplier : 1.0000
Dilution : 1.0000
Use Multiplier & Dilution Factor with ISTDs

Signal 1: DAD1 C, Sig=220,4 Ref=360,100

Peak #	RetTime [min]	Type	Width [min]	Area [mAU*s]	Height [mAU]	Area %
1	13.013	BV E	0.2006	366.94022	27.69195	6.6671
2	13.602	VB R	0.2143	5136.77002	360.45105	93.3329

Totals : 5503.71024 388.14300

Supplementary Figure 108. HPLC spectrum of rac-**2i**

Data File D:\CHEMSTA...83 84 85 86-1 2018.12.16 2018-12-16 19-28-49\relacation00002--003.D
Sample Name: xy-1-89-1

===

Acq. Operator : SYSTEM Seq. Line : 3
Sample Operator : SYSTEM
Acq. Instrument : LC Location : P1-F-01
Injection Date : 12/16/2018 7:50:27 PM Inj : 1
Inj Volume : 1.000 µl
Acq. Method : D:\ChemStation\1\Data\DXY\xy-1-83 84 85 86-1 2018.12.16 2018-12-16 19-28-49 \OJ3-97-3-0.8ML-55min.M
Last changed : 10/30/2018 4:44:46 PM by SYSTEM
Analysis Method : D:\ChemStation\1\Data\DXY\xy-1-83 84 85 86-1 2018.12.16 2018-12-16 19-28-49 \OJ3-97-3-0.8ML-55min.M (Sequence Method)
Last changed : 10/23/2019 7:26:58 PM by SYSTEM
(modified after loading)
Additional Info : Peak(s) manually integrated

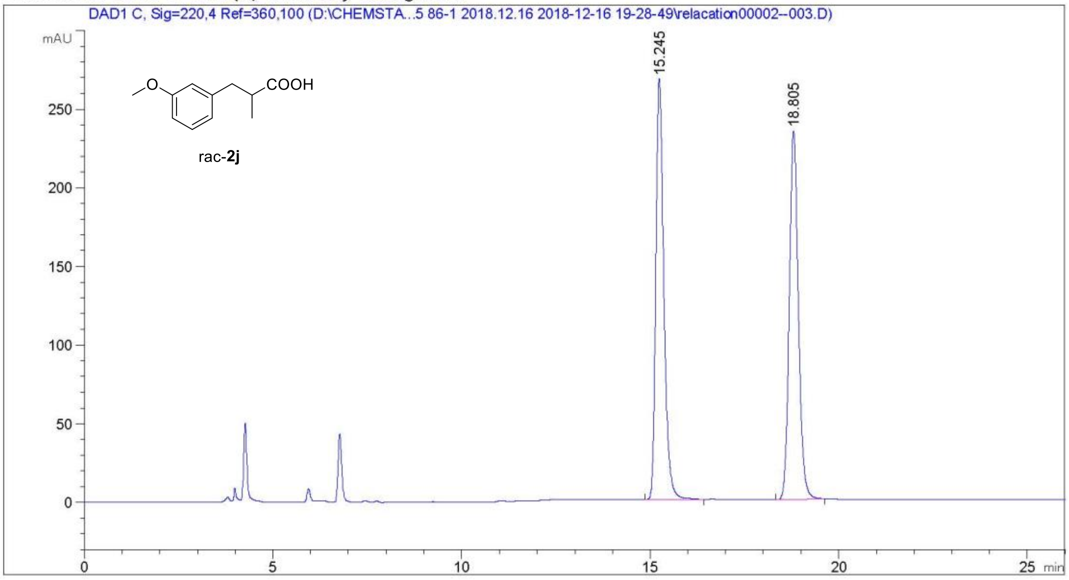

===

Area Percent Report

===

Sorted By : Signal
Multiplier : 1.0000
Dilution : 1.0000
Use Multiplier & Dilution Factor with ISTDs

Signal 1: DAD1 C, Sig=220,4 Ref=360,100

Peak #	RetTime [min]	Type	Width [min]	Area [mAU*s]	Height [mAU]	Area %
1	15.245	BB	0.2233	3930.35059	267.65866	50.1616
2	18.805	BB	0.2545	3905.02612	234.19073	49.8384

Totals : 7835.37671 501.84940

Supplementary Figure 109. HPLC spectrum of rac-**2j**

Data File D:\ChemSta...83 84 85 86-1 2018.12.16 2018-12-16 19-28-49\relacation00003--004.D
Sample Name: xy-1-85-1

===

```
Acq. Operator   : SYSTEM                        Seq. Line :   4
Sample Operator : SYSTEM
Acq. Instrument : LC                             Location :   P1-F-02
Injection Date  : 12/16/2018 8:46:21 PM               Inj :   1
                                               Inj Volume : 1.000 µl
Acq. Method     : D:\ChemStation\1\Data\DXY\xy-1-83 84 85 86-1 2018.12.16 2018-12-16 19-28-49
                  \OJ3-97-3-0.8ML-55min.M
Last changed    : 10/30/2018 4:44:46 PM by SYSTEM
Analysis Method : D:\ChemStation\1\Data\DXY\xy-1-83 84 85 86-1 2018.12.16 2018-12-16 19-28-49
                  \OJ3-97-3-0.8ML-55min.M (Sequence Method)
Last changed    : 10/23/2019 7:28:49 PM by SYSTEM
                  (modified after loading)
Additional Info : Peak(s) manually integrated
```

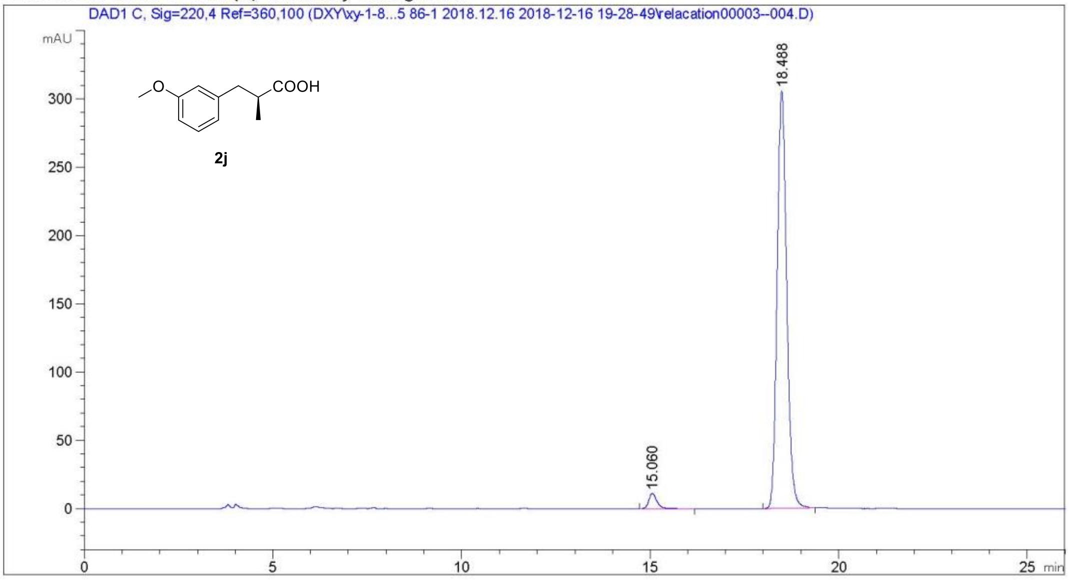

===

Area Percent Report

===

```
Sorted By             :      Signal
Multiplier            :      1.0000
Dilution              :      1.0000
Use Multiplier & Dilution Factor with ISTDs
```

Signal 1: DAD1 C, Sig=220,4 Ref=360,100

Peak #	RetTime [min]	Type	Width [min]	Area [mAU*s]	Height [mAU]	Area %
1	15.060	BB	0.2288	165.57222	11.05037	2.9944
2	18.488	BB	0.2710	5363.88770	305.30130	97.0056

Totals : 5529.45992 316.35167

Supplementary Figure 110. HPLC spectrum of **2j**

Data File D:\CHEMSTA...DATA\DXY\DXY-1-103-1 2019.1.4 2019-01-04 22-01-49\relacation00003.D
Sample Name: xy-1-98-1

===

Acq. Operator : SYSTEM Seq. Line : 3
Sample Operator : SYSTEM
Acq. Instrument : LC Location : P1-D-09
Injection Date : 1/4/2019 10:23:28 PM Inj : 1
Inj Volume : 1.000 µl
Acq. Method : D:\ChemStation\1\Data\DXY\dxy-1-103-1 2019.1.4 2019-01-04 22-01-49\OJ3-97-3-0.8ML-30min.M
Last changed : 1/2/2019 10:18:19 PM by SYSTEM
Analysis Method : D:\ChemStation\1\Data\DXY\dxy-1-103-1 2019.1.4 2019-01-04 22-01-49\OJ3-97-3-0.8ML-30min.M (Sequence Method)
Last changed : 10/24/2019 2:49:02 PM by SYSTEM
(modified after loading)
Additional Info : Peak(s) manually integrated

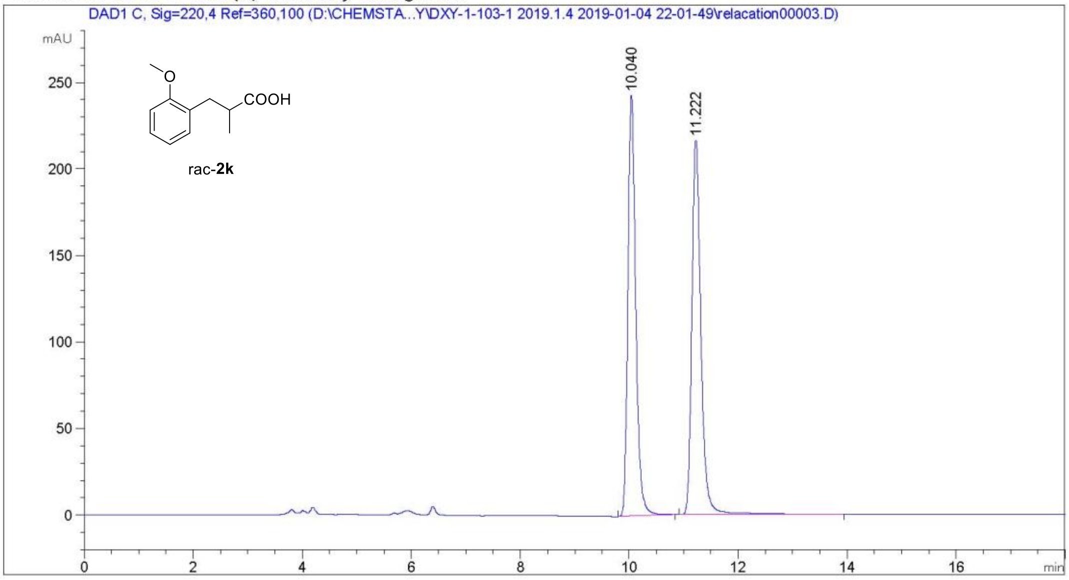

===

Area Percent Report

===

Sorted By : Signal
Multiplier : 1.0000
Dilution : 1.0000
Use Multiplier & Dilution Factor with ISTDs

Signal 1: DAD1 C, Sig=220,4 Ref=360,100

Peak #	RetTime [min]	Type	Width [min]	Area [mAU*s]	Height [mAU]	Area %
1	10.040	BB	0.1523	2422.96313	242.99103	49.2396
2	11.222	BB	0.1746	2497.79810	216.44078	50.7604

Totals : 4920.76123 459.43181

Supplementary Figure 111. HPLC spectrum of rac-**2k**

Data File D:\ChemSta...Data\DXY\dxy-1-103-1 2019.1.4 2019-01-04 22-01-49\relacation00004.D
Sample Name: dxy-1-103-1

===

Acq. Operator : SYSTEM   Seq. Line : 4
Sample Operator : SYSTEM
Acq. Instrument : LC   Location : P1-D-10
Injection Date : 1/4/2019 10:54:20 PM   Inj : 1
Inj Volume : 1.000 µl
Acq. Method : D:\ChemStation\1\Data\DXY\dxy-1-103-1 2019.1.4 2019-01-04 22-01-49\OJ3-97-3-0.8ML-30min.M
Last changed : 1/2/2019 10:18:19 PM by SYSTEM
Analysis Method : D:\ChemStation\1\Data\DXY\dxy-1-103-1 2019.1.4 2019-01-04 22-01-49\OJ3-97-3-0.8ML-30min.M (Sequence Method)
Last changed : 10/24/2019 2:50:00 PM by SYSTEM
(modified after loading)
Additional Info : Peak(s) manually integrated

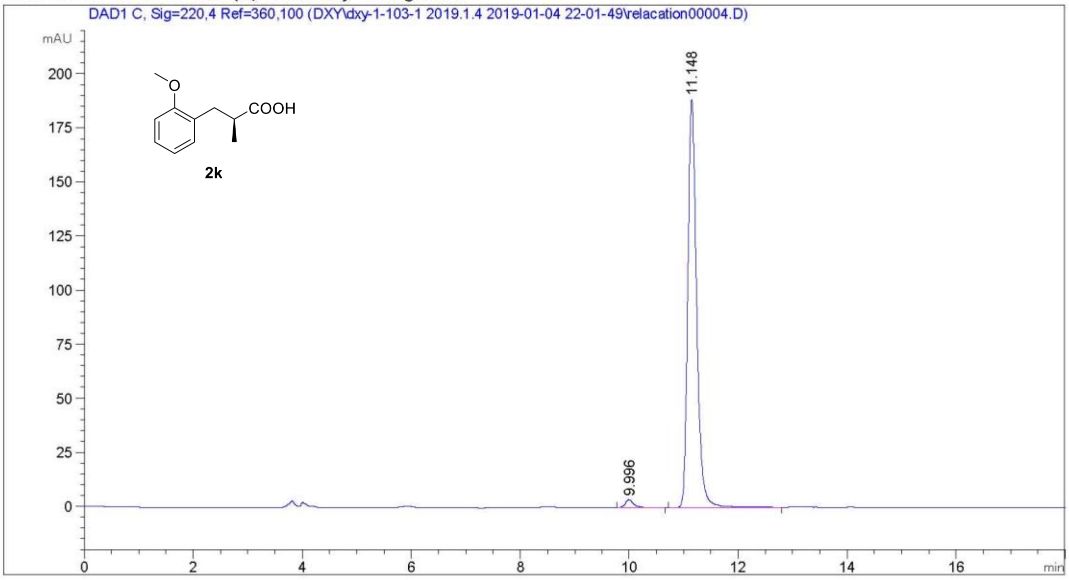

===

Area Percent Report

===

Sorted By : Signal
Multiplier : 1.0000
Dilution : 1.0000
Use Multiplier & Dilution Factor with ISTDs

Signal 1: DAD1 C, Sig=220,4 Ref=360,100

Peak #	RetTime [min]	Type	Width [min]	Area [mAU*s]	Height [mAU]	Area %
1	9.996	BB	0.1521	34.96122	3.45368	1.6356
2	11.148	BB	0.1701	2102.58887	188.60707	98.3644

Totals : 2137.55009 192.06075

Supplementary Figure 112. HPLC spectrum of **2k**

Data File D:\ChemStation\1\Data\DXY\2019.1.22-2 2019-01-22 22-01-13\relacation00002.D
Sample Name: XY-1-102-1

===

```
Acq. Operator   : SYSTEM                        Seq. Line :   2
Sample Operator : SYSTEM
Acq. Instrument : LC                             Location :   P1-D-01
Injection Date  : 1/22/2019 10:12:27 PM               Inj :   1
                                              Inj Volume : 1.000 µl
Acq. Method     : D:\ChemStation\1\Data\DXY\2019.1.22-2 2019-01-22 22-01-13\OJ3-97-3-0.8ML-
                  30min.M
Last changed    : 1/2/2019 10:18:19 PM by SYSTEM
Analysis Method : D:\ChemStation\1\Data\DXY\2019.1.22-2 2019-01-22 22-01-13\OJ3-97-3-0.8ML-
                  30min.M (Sequence Method)
Last changed    : 10/24/2019 3:19:13 PM by SYSTEM
                  (modified after loading)
Additional Info : Peak(s) manually integrated
```

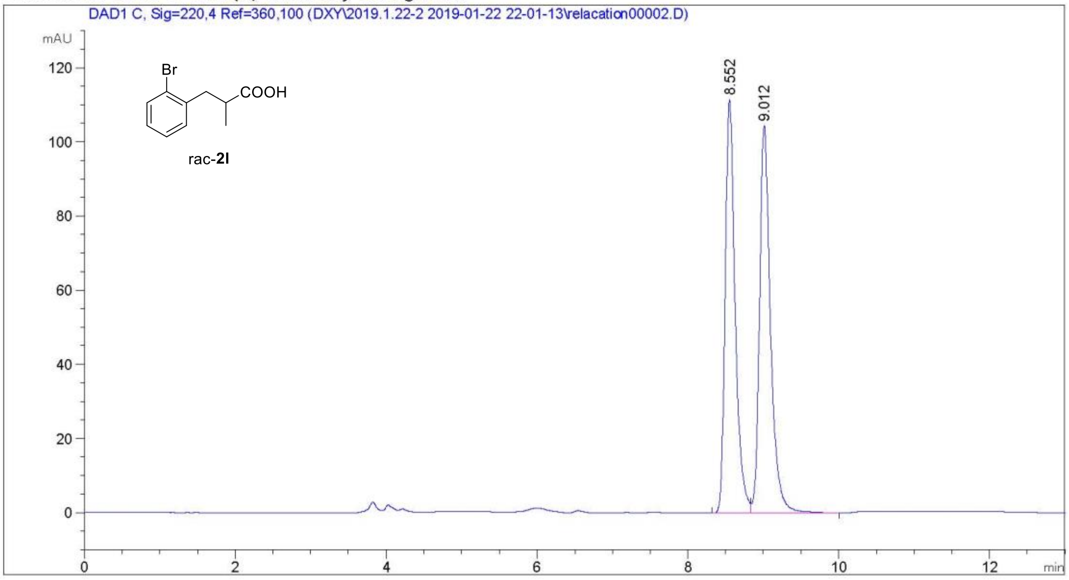

===

Area Percent Report

===

```
Sorted By             :      Signal
Multiplier            :      1.0000
Dilution              :      1.0000
Use Multiplier & Dilution Factor with ISTDs
```

Signal 1: DAD1 C, Sig=220,4 Ref=360,100

Peak #	RetTime [min]	Type	Width [min]	Area [mAU*s]	Height [mAU]	Area %
1	8.552	BV	0.1388	1020.83771	111.48037	49.2776
2	9.012	VB	0.1493	1050.76794	104.48247	50.7224

Totals : 2071.60565 215.96284

Supplementary Figure 113. HPLC spectrum of rac-**2l**

Data File D:\ChemStation\1\Data\DXY\2019.1.22-2 2019-01-22 22-01-13\relacation00003.D
Sample Name: XY-1-106-1

===

Acq. Operator : SYSTEM   Seq. Line : 3
Sample Operator : SYSTEM
Acq. Instrument : LC   Location : P1-D-02
Injection Date : 1/22/2019 10:43:19 PM   Inj : 1
Inj Volume : 1.000 µl
Acq. Method : D:\ChemStation\1\Data\DXY\2019.1.22-2 2019-01-22 22-01-13\OJ3-97-3-0.8ML-30min.M
Last changed : 1/2/2019 10:18:19 PM by SYSTEM
Analysis Method : D:\ChemStation\1\Data\DXY\2019.1.22-2 2019-01-22 22-01-13\OJ3-97-3-0.8ML-30min.M (Sequence Method)
Last changed : 10/24/2019 3:18:36 PM by SYSTEM
(modified after loading)
Additional Info : Peak(s) manually integrated

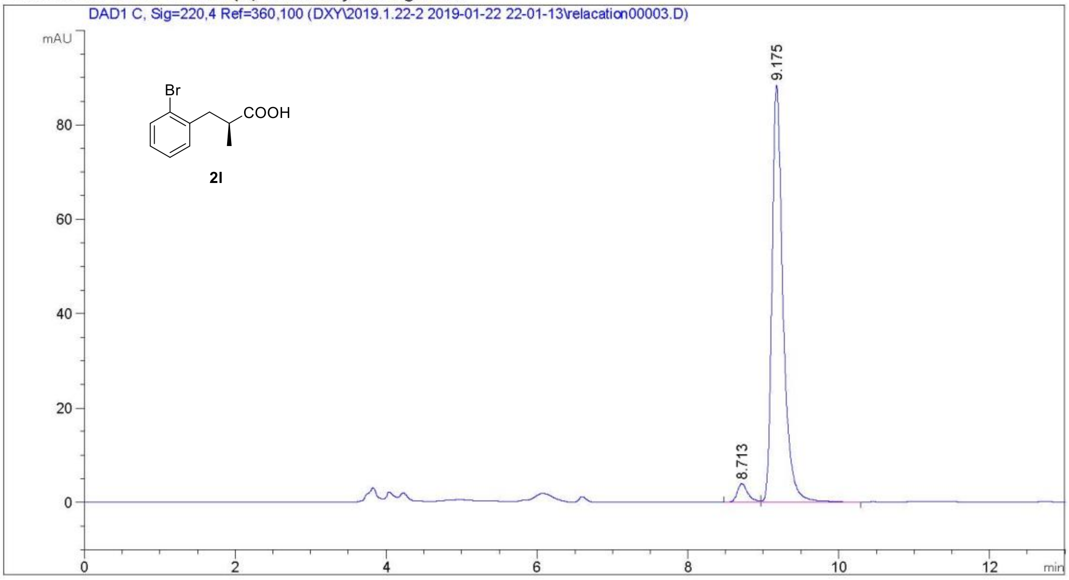

===

Area Percent Report

===

Sorted By : Signal
Multiplier : 1.0000
Dilution : 1.0000
Use Multiplier & Dilution Factor with ISTDs

Signal 1: DAD1 C, Sig=220,4 Ref=360,100

Peak #	RetTime [min]	Type	Width [min]	Area [mAU*s]	Height [mAU]	Area %
1	8.713	BV	0.1408	37.00693	3.89830	3.9889
2	9.175	VB	0.1515	890.73456	88.40613	96.0111

Totals : 927.74149 92.30443

Supplementary Figure 114. HPLC spectrum of **2l**

Data File D:\CHEMSTATION\1\DATA\DXY\2019.1.22-2 2019-01-22 22-01-13\relacation00004.D
Sample Name: XY-1-135-1

===

Acq. Operator : SYSTEM Seq. Line : 4
Sample Operator : SYSTEM
Acq. Instrument : LC Location : P1-D-03
Injection Date : 1/22/2019 11:14:12 PM Inj : 1
Inj Volume : 1.000 µl
Acq. Method : D:\ChemStation\1\Data\DXY\2019.1.22-2 2019-01-22 22-01-13\OJ3-97-3-0.8ML-30min.M
Last changed : 1/2/2019 10:18:19 PM by SYSTEM
Analysis Method : D:\ChemStation\1\Data\DXY\2019.1.22-2 2019-01-22 22-01-13\OJ3-97-3-0.8ML-30min.M (Sequence Method)
Last changed : 10/24/2019 3:29:29 PM by SYSTEM
(modified after loading)
Additional Info : Peak(s) manually integrated

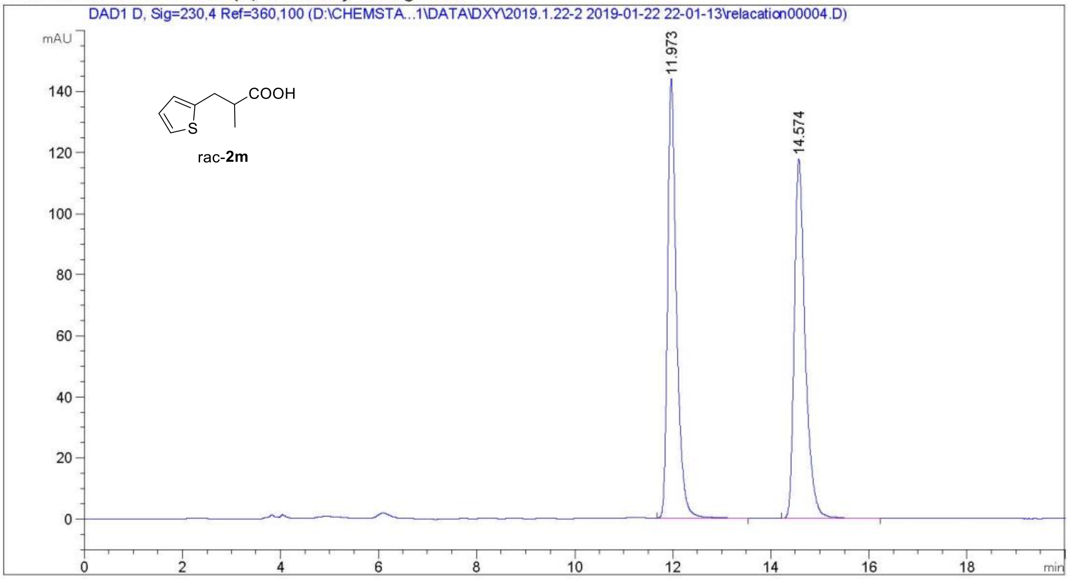

===

Area Percent Report

===

Sorted By : Signal
Multiplier : 1.0000
Dilution : 1.0000
Use Multiplier & Dilution Factor with ISTDs

Signal 1: DAD1 D, Sig=230,4 Ref=360,100

Peak #	RetTime [min]	Type	Width [min]	Area [mAU*s]	Height [mAU]	Area %
1	11.973	BB	0.1888	1811.52527	144.00896	50.0395
2	14.574	BB	0.2312	1808.66296	117.74893	49.9605

Totals : 3620.18823 261.75789

Supplementary Figure 115. HPLC spectrum of rac-**2m**

Data File D:\ChemStation\1\Data\DXY\2019.1.22-2 2019-01-22 22-01-13\relacation00005.D
Sample Name: XY-1-103-1

===

Acq. Operator : SYSTEM Seq. Line : 5
Sample Operator : SYSTEM
Acq. Instrument : LC Location : P1-D-04
Injection Date : 1/22/2019 11:45:04 PM Inj : 1
Inj Volume : 1.000 µl
Acq. Method : D:\ChemStation\1\Data\DXY\2019.1.22-2 2019-01-22 22-01-13\OJ3-97-3-0.8ML-30min.M
Last changed : 1/2/2019 10:18:19 PM by SYSTEM
Analysis Method : D:\ChemStation\1\Data\DXY\2019.1.22-2 2019-01-22 22-01-13\OJ3-97-3-0.8ML-30min.M (Sequence Method)
Last changed : 10/24/2019 3:30:30 PM by SYSTEM
(modified after loading)
Additional Info : Peak(s) manually integrated

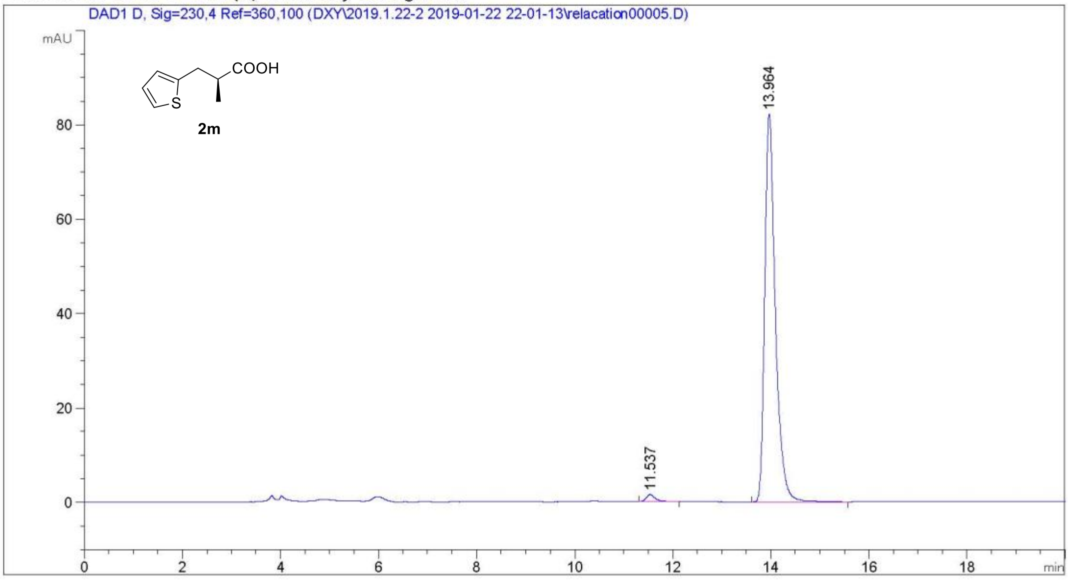

===

Area Percent Report

===

Sorted By : Signal
Multiplier : 1.0000
Dilution : 1.0000
Use Multiplier & Dilution Factor with ISTDs

Signal 1: DAD1 D, Sig=230,4 Ref=360,100

Peak #	RetTime [min]	Type	Width [min]	Area [mAU*s]	Height [mAU]	Area %
1	11.537	BB	0.1799	17.90438	1.47200	1.4398
2	13.964	BB	0.2220	1225.64258	82.15739	98.5602

Totals : 1243.54696 83.62939

Supplementary Figure 116. HPLC spectrum of **2m**

```
Data File D:\CHEMSTA...83 84 85 86-1 2018.12.16 2018-12-16 19-28-49\relacation00004--005.D
Sample Name: xy-1-90-1

    =====================================================================
    Acq. Operator   : SYSTEM                         Seq. Line :    5
    Sample Operator : SYSTEM
    Acq. Instrument : LC                              Location :    P1-F-03
    Injection Date  : 12/16/2018 9:42:15 PM                Inj :    1
                                                    Inj Volume : 1.000 µl
    Acq. Method     : D:\ChemStation\1\Data\DXY\xy-1-83 84 85 86-1 2018.12.16 2018-12-16 19-28-49
                      \OJ3-97-3-0.8ML-55min.M
    Last changed    : 10/30/2018 4:44:46 PM by SYSTEM
    Analysis Method : D:\ChemStation\1\Data\DXY\xy-1-83 84 85 86-1 2018.12.16 2018-12-16 19-28-49
                      \OJ3-97-3-0.8ML-55min.M (Sequence Method)
    Last changed    : 10/23/2019 7:32:37 PM by SYSTEM
                      (modified after loading)
    Additional Info : Peak(s) manually integrated
```

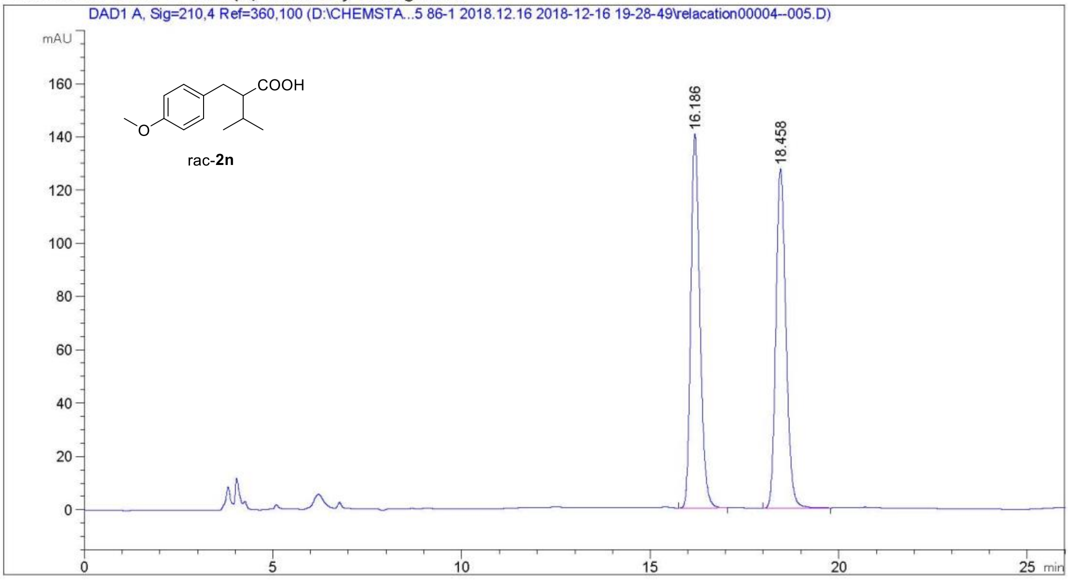


```
    =====================================================================
                             Area Percent Report
    =====================================================================

    Sorted By             :      Signal
    Multiplier            :      1.0000
    Dilution              :      1.0000
    Use Multiplier & Dilution Factor with ISTDs


    Signal 1: DAD1 A, Sig=210,4 Ref=360,100

    Peak RetTime Type  Width     Area      Height     Area
      #   [min]        [min]   [mAU*s]     [mAU]        %
    ----|-------|----|-------|----------|----------|--------|
       1  16.186 BB    0.2569 2348.12280  140.57016  49.8520
       2  18.458 BB    0.2860 2362.06348  127.56287  50.1480

    Totals :                  4710.18628  268.13303
```


Supplementary Figure 117. HPLC spectrum of rac-**2n**

Data File D:\ChemSta...83 84 85 86-1 2018.12.16 2018-12-16 19-28-49\relacation00005--006.D
Sample Name: xy-1-86-1

===

Acq. Operator : SYSTEM   Seq. Line : 6
Sample Operator : SYSTEM
Acq. Instrument : LC   Location : P1-F-04
Injection Date : 12/16/2018 10:38:09 PM   Inj : 1
Inj Volume : 1.000 µl
Acq. Method : D:\ChemStation\1\Data\DXY\xy-1-83 84 85 86-1 2018.12.16 2018-12-16 19-28-49 \OJ3-97-3-0.8ML-55min.M
Last changed : 10/30/2018 4:44:46 PM by SYSTEM
Analysis Method : D:\ChemStation\1\Data\DXY\xy-1-83 84 85 86-1 2018.12.16 2018-12-16 19-28-49 \OJ3-97-3-0.8ML-55min.M (Sequence Method)
Last changed : 10/24/2019 2:41:52 PM by SYSTEM
(modified after loading)
Additional Info : Peak(s) manually integrated

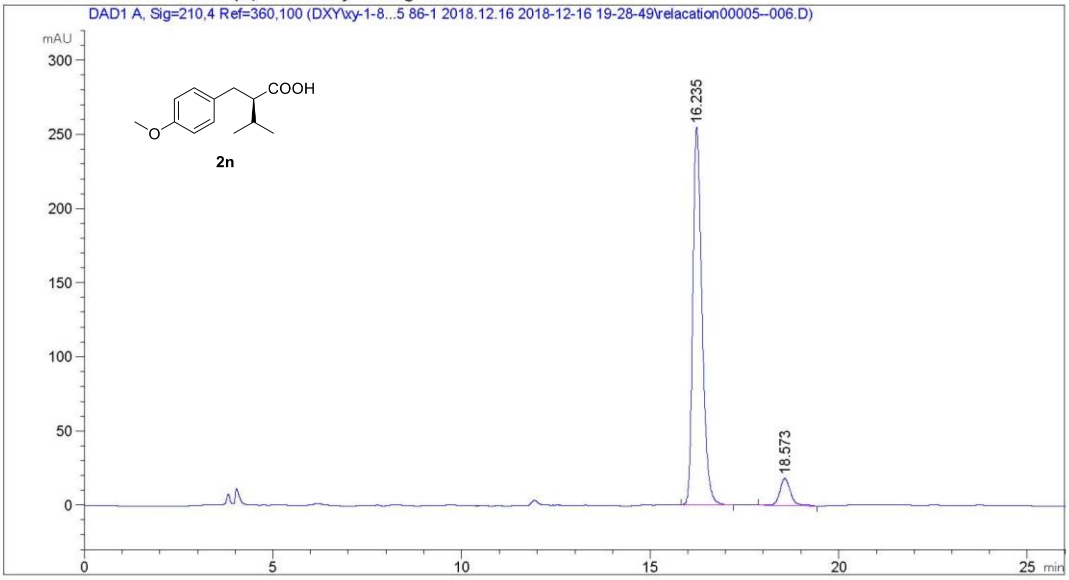

===

Area Percent Report

===

Sorted By : Signal
Multiplier : 1.0000
Dilution : 1.0000
Use Multiplier & Dilution Factor with ISTDs

Signal 1: DAD1 A, Sig=210,4 Ref=360,100

Peak #	RetTime [min]	Type	Width [min]	Area [mAU*s]	Height [mAU]	Area %
1	16.235	BB	0.2571	4299.36475	254.45367	92.2646
2	18.573	BB	0.2996	360.45657	18.31259	7.7354

Totals : 4659.82132 272.76626

Supplementary Figure 118. HPLC spectrum of **2n**

Data File D:\ChemSta...05 106-1 dxy-1-104-1 2019.1.2 2019-01-02 22-25-36\relacation00012.D
Sample Name: xy-1-101-1

===

Acq. Operator : SYSTEM Seq. Line : 12
Sample Operator : SYSTEM
Acq. Instrument : LC Location : P1-D-07
Injection Date : 1/3/2019 3:08:53 AM Inj : 1
Inj Volume : 2.000 µl
Acq. Method : D:\ChemStation\1\Data\DXY\xy-1-94 105 106-1 dxy-1-104-1 2019.1.2 2019-01-02 22-25-36\AD3-97-3-0.8ML-50min.M
Last changed : 9/12/2018 9:16:06 PM by SYSTEM
Analysis Method : D:\ChemStation\1\Data\DXY\xy-1-94 105 106-1 dxy-1-104-1 2019.1.2 2019-01-02 22-25-36\AD3-97-3-0.8ML-50min.M (Sequence Method)
Last changed : 10/24/2019 3:59:44 PM by SYSTEM
(modified after loading)
Additional Info : Peak(s) manually integrated

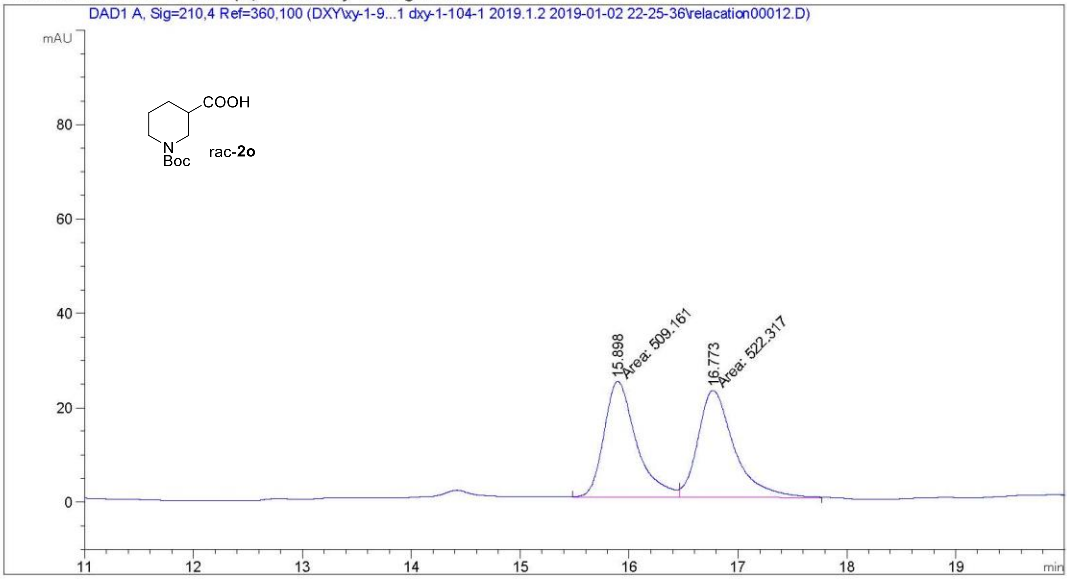

===

Area Percent Report

===

Sorted By : Signal
Multiplier : 1.0000
Dilution : 1.0000
Use Multiplier & Dilution Factor with ISTDs

Signal 1: DAD1 A, Sig=210,4 Ref=360,100

Peak #	RetTime [min]	Type	Width [min]	Area [mAU*s]	Height [mAU]	Area %
1	15.898	MF	0.3457	509.16071	24.54621	49.3623
2	16.773	FM	0.3837	522.31677	22.68739	50.6377

Totals : 1031.47748 47.23359

Supplementary Figure 119. HPLC spectrum of rac-**2o**

Data File D:\ChemSta...05 106-1 dxy-1-104-1 2019.1.2 2019-01-02 22-25-36\relacation00013.D
Sample Name: xy-1-105-1

===

Acq. Operator : SYSTEM Seq. Line : 13
Sample Operator : SYSTEM
Acq. Instrument : LC Location : P1-D-08
Injection Date : 1/3/2019 3:59:46 AM Inj : 1
Inj Volume : 2.000 µl
Acq. Method : D:\ChemStation\1\Data\DXY\xy-1-94 105 106-1 dxy-1-104-1 2019.1.2 2019-01-02 22-25-36\AD3-97-3-0.8ML-50min.M
Last changed : 9/12/2018 9:16:06 PM by SYSTEM
Analysis Method : D:\ChemStation\1\Data\DXY\xy-1-94 105 106-1 dxy-1-104-1 2019.1.2 2019-01-02 22-25-36\AD3-97-3-0.8ML-50min.M (Sequence Method)
Last changed : 10/24/2019 3:36:50 PM by SYSTEM
(modified after loading)
Additional Info : Peak(s) manually integrated

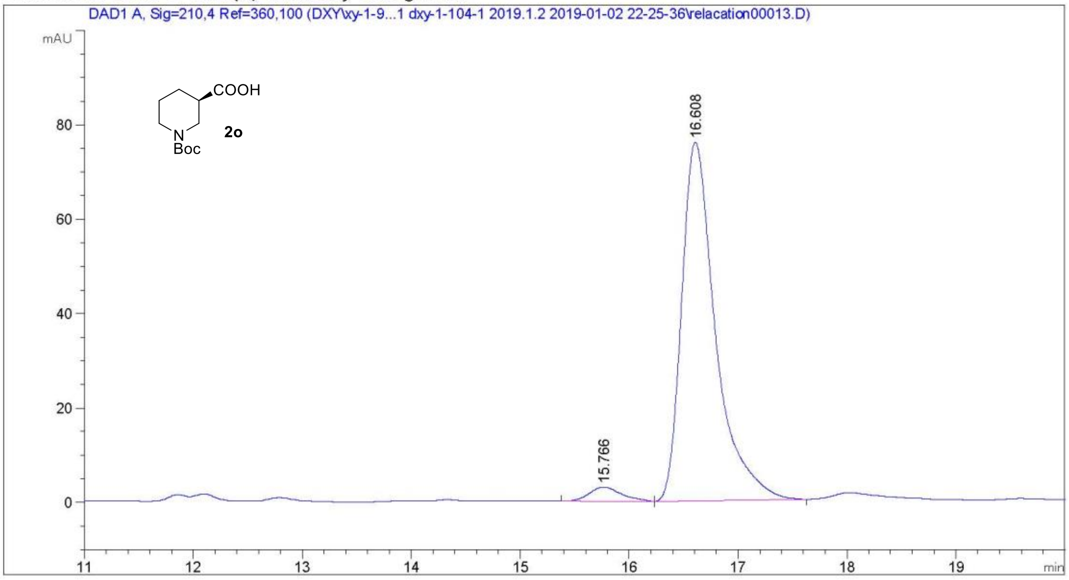

===

Area Percent Report

===

Sorted By : Signal
Multiplier : 1.0000
Dilution : 1.0000
Use Multiplier & Dilution Factor with ISTDs

Signal 1: DAD1 A, Sig=210,4 Ref=360,100

Peak #	RetTime [min]	Type	Width [min]	Area [mAU*s]	Height [mAU]	Area %
1	15.766	BB	0.3002	61.28635	3.00226	3.6032
2	16.608	BB	0.3258	1639.61218	75.97534	96.3968

Totals : 1700.89854 78.97761

Supplementary Figure 120. HPLC spectrum of **2o**

Data File D:\ChemSta...\XY-1-139 140 141-1 2017.2.27 2019-02-27 21-28-30\relacation00003.D
Sample Name: XY-1-142-1

===

Acq. Operator : SYSTEM Seq. Line : 3
Sample Operator : SYSTEM
Acq. Instrument : LC Location : P1-F-01
Injection Date : 2/27/2019 9:51:35 PM Inj : 1
Inj Volume : 1.000 µl
Acq. Method : D:\ChemStation\1\Data\DXY\XY-1-139 140 141-1 2017.2.27 2019-02-27 21-28-30 \OJ3-97-3-1ML-50min.M
Last changed : 9/20/2018 7:08:47 PM by SYSTEM
Analysis Method : D:\ChemStation\1\Data\DXY\XY-1-139 140 141-1 2017.2.27 2019-02-27 21-28-30 \OJ3-97-3-1ML-50min.M (Sequence Method)
Last changed : 10/24/2019 5:04:18 PM by SYSTEM
(modified after loading)
Additional Info : Peak(s) manually integrated

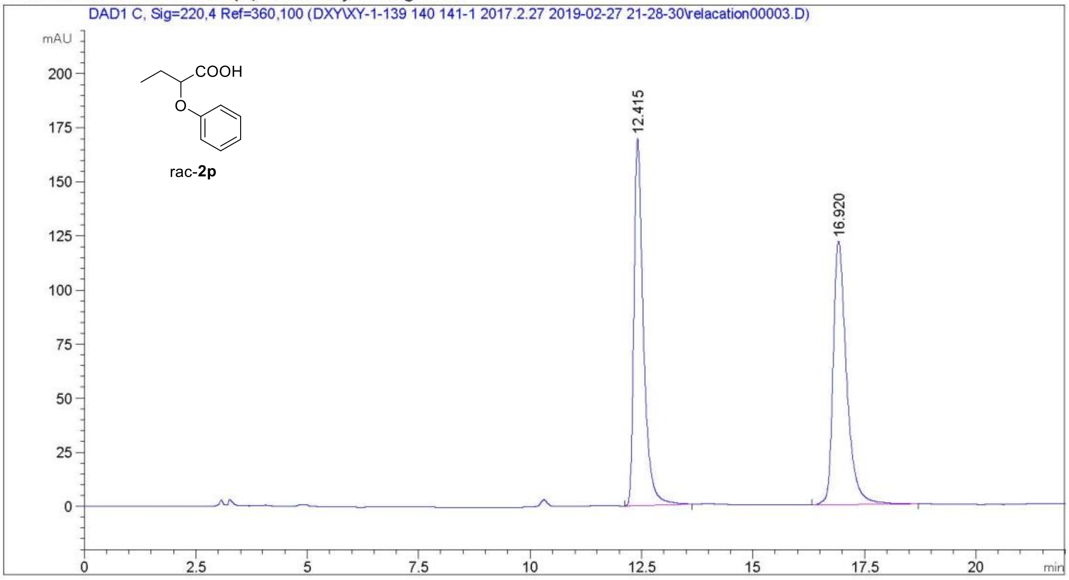

===

Area Percent Report

===

Sorted By : Signal
Multiplier : 1.0000
Dilution : 1.0000
Use Multiplier & Dilution Factor with ISTDs

Signal 1: DAD1 C, Sig=220,4 Ref=360,100

Peak #	RetTime [min]	Type	Width [min]	Area [mAU*s]	Height [mAU]	Area %
1	12.415	BB	0.2194	2492.04736	169.65915	49.3941
2	16.920	BB	0.3146	2553.18872	121.79343	50.6059

Totals : 5045.23608 291.45258

Supplementary Figure 121. HPLC spectrum of rac-**2p**

Data File D:\ChemSta...\XY-1-139 140 141-1 2017.2.27 2019-02-27 21-28-30\relacation00004.D
Sample Name: XY-1-139-1

===

Acq. Operator : SYSTEM Seq. Line : 4
Sample Operator : SYSTEM
Acq. Instrument : LC Location : P1-F-02
Injection Date : 2/27/2019 10:42:27 PM Inj : 1
Inj Volume : 1.000 µl
Acq. Method : D:\ChemStation\1\Data\DXY\XY-1-139 140 141-1 2017.2.27 2019-02-27 21-28-30 \OJ3-97-3-1ML-50min.M
Last changed : 9/20/2018 7:08:47 PM by SYSTEM
Analysis Method : D:\ChemStation\1\Data\DXY\XY-1-139 140 141-1 2017.2.27 2019-02-27 21-28-30 \OJ3-97-3-1ML-50min.M (Sequence Method)
Last changed : 10/24/2019 5:04:18 PM by SYSTEM
(modified after loading)
Additional Info : Peak(s) manually integrated

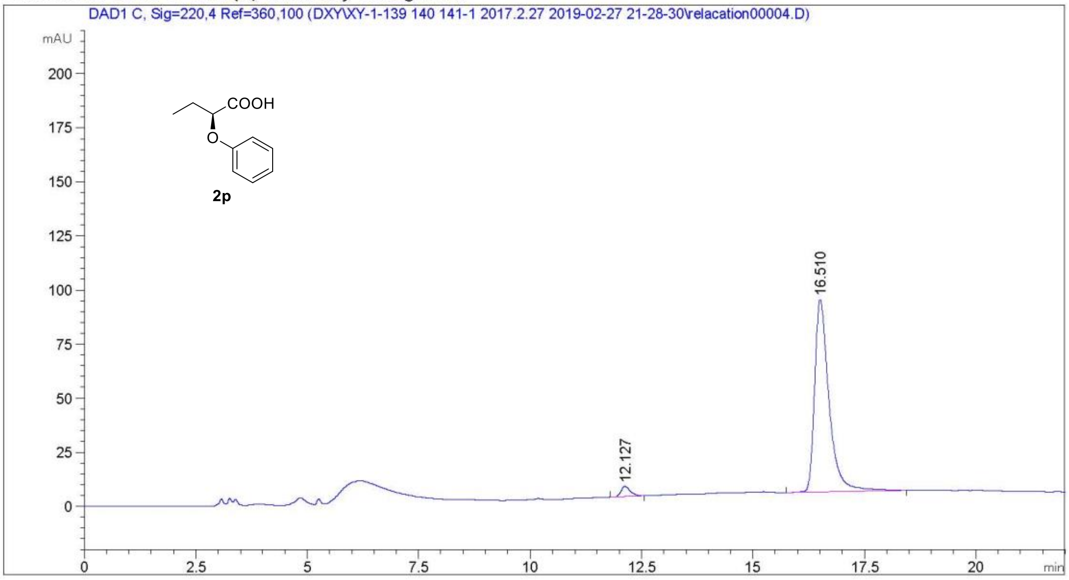

===
Area Percent Report
===

Sorted By : Signal
Multiplier : 1.0000
Dilution : 1.0000
Use Multiplier & Dilution Factor with ISTDs

Signal 1: DAD1 C, Sig=220,4 Ref=360,100

Peak #	RetTime [min]	Type	Width [min]	Area [mAU*s]	Height [mAU]	Area %
1	12.127	BB	0.2192	69.19384	4.71460	3.3655
2	16.510	BB	0.3350	1986.80103	88.82665	96.6345

Totals : 2055.99487 93.54126

Supplementary Figure 122. HPLC spectrum of **2p**

Data File D:\CHEMSTATION\1\DATA\DXY\2019.1.15-2 2019-01-25 17-54-26\OnlineEdited--004.D
Sample Name: XY-1-119-1

===

Acq. Operator : SYSTEM   Seq. Line : 4
Sample Operator : SYSTEM
Acq. Instrument : LC   Location : P2-B-01
Injection Date : 1/25/2019 7:06:07 PM   Inj : 1
Inj Volume : 1.000 µl
Acq. Method : D:\ChemStation\1\Data\DXY\2019.1.15-2 2019-01-25 17-54-26\AD3-97-3-1ML-40min.M
Last changed : 1/25/2019 7:23:47 PM by SYSTEM
(modified after loading)
Analysis Method : D:\ChemStation\1\Data\DXY\2019.1.15-2 2019-01-25 17-54-26\AD3-97-3-1ML-40min.M (Sequence Method)
Last changed : 10/24/2019 4:36:44 PM by SYSTEM
(modified after loading)
Additional Info : Peak(s) manually integrated

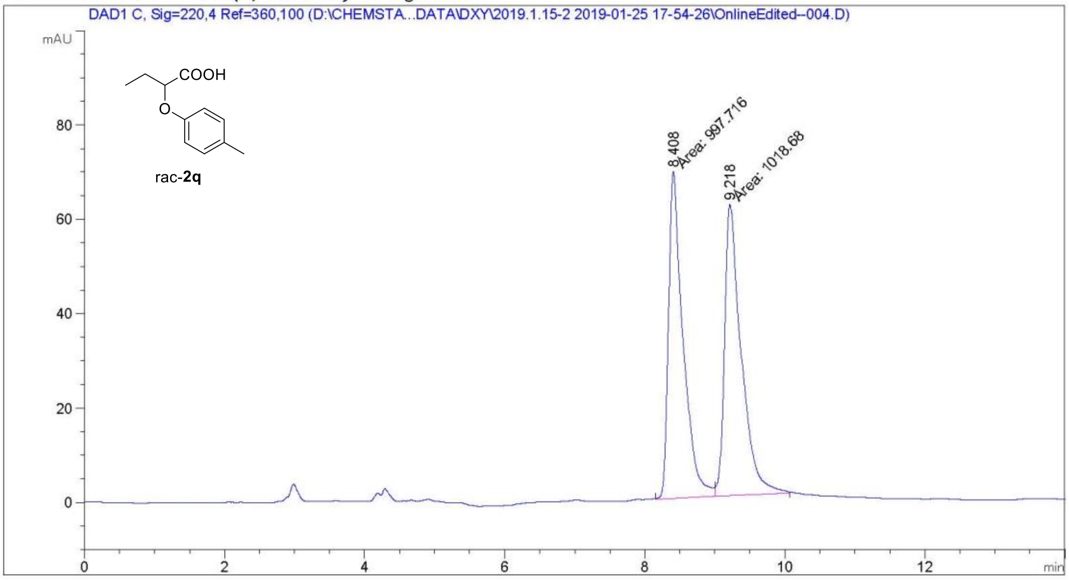

===
Area Percent Report
===

Sorted By : Signal
Multiplier : 1.0000
Dilution : 1.0000
Use Multiplier & Dilution Factor with ISTDs

Signal 1: DAD1 C, Sig=220,4 Ref=360,100

Peak #	RetTime [min]	Type	Width [min]	Area [mAU*s]	Height [mAU]	Area %
1	8.408	MF	0.2400	997.71576	69.28680	49.4801
2	9.218	FM	0.2751	1018.68250	61.71930	50.5199

Supplementary Figure 123. HPLC spectrum of rac-**2q**

Data File D:\ChemStation\1\Data\DXY\2019.1.15-2 2019-01-25 17-54-26\OnlineEdited--005.D
Sample Name: XY-1-137-1

===

Acq. Operator : SYSTEM Seq. Line : 5
Sample Operator : SYSTEM
Acq. Instrument : LC Location : P2-B-02
Injection Date : 1/25/2019 7:24:55 PM Inj : 1
Inj Volume : 1.000 µl
Acq. Method : D:\ChemStation\1\Data\DXY\2019.1.15-2 2019-01-25 17-54-26\AD3-97-3-1ML-40min.M
Last changed : 1/25/2019 7:23:47 PM by SYSTEM
Analysis Method : D:\ChemStation\1\Data\DXY\2019.1.15-2 2019-01-25 17-54-26\AD3-97-3-1ML-40min.M (Sequence Method)
Last changed : 10/24/2019 4:37:40 PM by SYSTEM
(modified after loading)
Additional Info : Peak(s) manually integrated

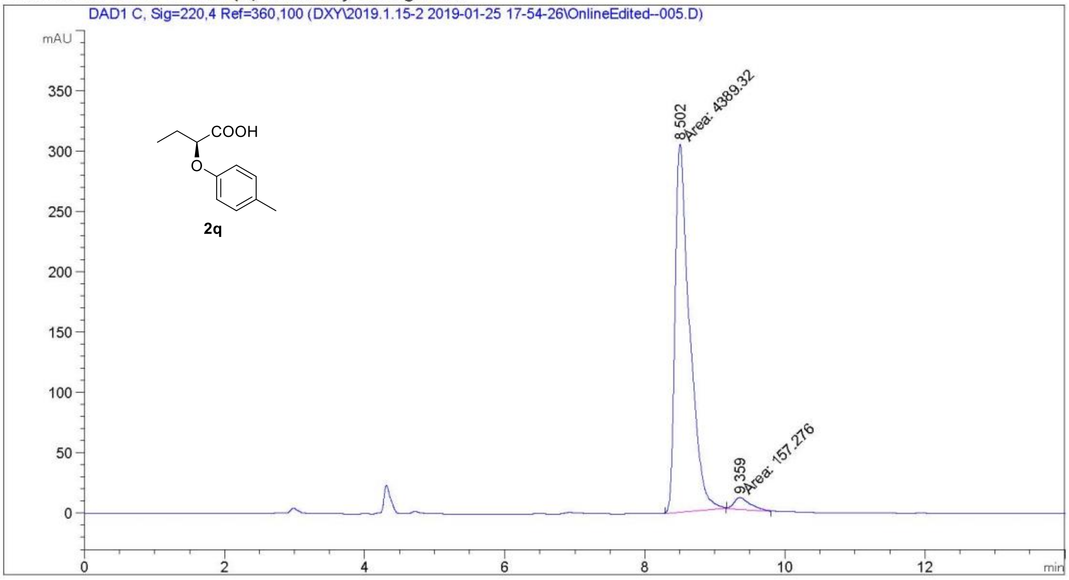

===

Area Percent Report

===

Sorted By : Signal
Multiplier : 1.0000
Dilution : 1.0000
Use Multiplier & Dilution Factor with ISTDs

Signal 1: DAD1 C, Sig=220,4 Ref=360,100

Peak #	RetTime [min]	Type	Width [min]	Area [mAU*s]	Height [mAU]	Area %
1	8.502	MM	0.2398	4389.32422	305.01480	96.5408
2	9.359	MM	0.2675	157.27599	9.79813	3.4592

Totals : 4546.60020 314.81293

Supplementary Figure 124. HPLC spectrum of rac-**2q**

Data File D:\CHEMSTA...ATA\DXY\XY-1-175 179 2019.4.1 2019-04-01 21-24-05\relacation00006.D
Sample Name: xy-1-117-1

===

Acq. Operator : SYSTEM Seq. Line : 6
Sample Operator : SYSTEM
Acq. Instrument : LC Location : P1-F-01
Injection Date : 4/2/2019 12:34:59 AM Inj : 1
Inj Volume : 2.000 µl
Acq. Method : D:\ChemStation\1\Data\DXY\xy-1-175 179 2019.4.1 2019-04-01 21-24-05\AD3-97-3-0.8ML-40min.M
Last changed : 3/12/2019 11:14:16 AM by SYSTEM
Analysis Method : D:\ChemStation\1\Data\DXY\xy-1-175 179 2019.4.1 2019-04-01 21-24-05\AD3-97-3-0.8ML-40min.M (Sequence Method)
Last changed : 10/24/2019 4:03:22 PM by SYSTEM
(modified after loading)
Additional Info : Peak(s) manually integrated

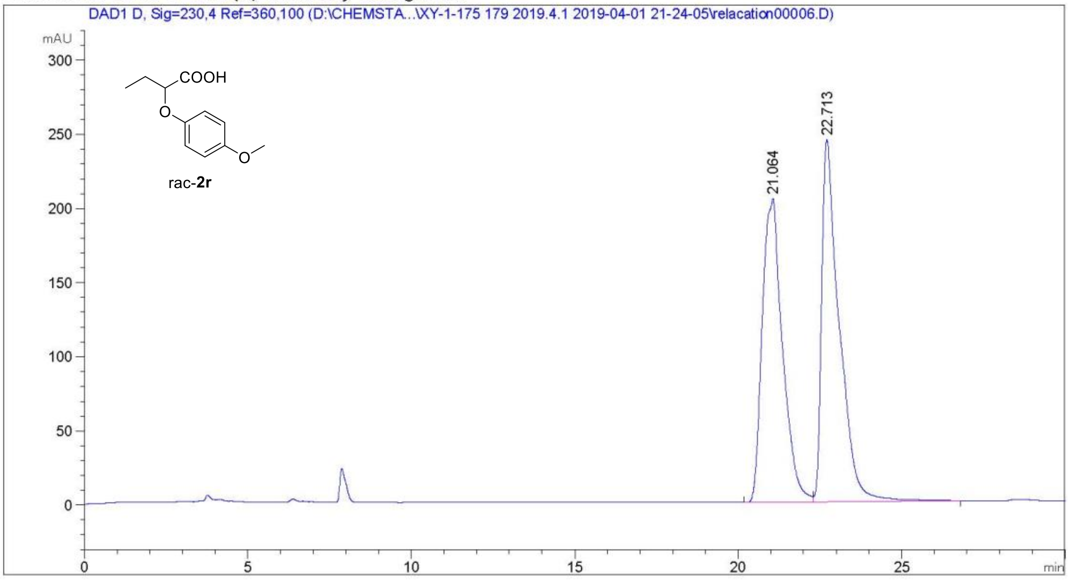

===

Area Percent Report

===

Sorted By : Signal
Multiplier : 1.0000
Dilution : 1.0000
Use Multiplier & Dilution Factor with ISTDs

Signal 1: DAD1 D, Sig=230,4 Ref=360,100

Peak #	RetTime [min]	Type	Width [min]	Area [mAU*s]	Height [mAU]	Area %
1	21.064	BV	0.5431	8783.70996	204.49966	49.1752
2	22.713	VB	0.5237	9078.35156	244.20110	50.8248

Totals : 1.78621e4 448.70076

Supplementary Figure 125. HPLC spectrum of rac-**2r**

```
Data File D:\ChemSta...ata\DXY\xy-1-175 179 2019.4.1 2019-04-01 21-24-05\relacation00007.D
Sample Name: xy-1-179-1

    =====================================================================
    Acq. Operator   : SYSTEM                        Seq. Line :   7
    Sample Operator : SYSTEM
    Acq. Instrument : LC                             Location :   P1-F-02
    Injection Date  : 4/2/2019 1:15:52 AM                 Inj :   1
                                                Inj Volume : 2.000 µl
    Acq. Method     : D:\ChemStation\1\Data\DXY\xy-1-175 179 2019.4.1 2019-04-01 21-24-05\AD3-97-
                      3-0.8ML-40min.M
    Last changed    : 3/12/2019 11:14:16 AM by SYSTEM
    Analysis Method : D:\ChemStation\1\Data\DXY\xy-1-175 179 2019.4.1 2019-04-01 21-24-05\AD3-97-
                      3-0.8ML-40min.M (Sequence Method)
    Last changed    : 10/24/2019 4:04:40 PM by SYSTEM
                      (modified after loading)
    Additional Info : Peak(s) manually integrated
```

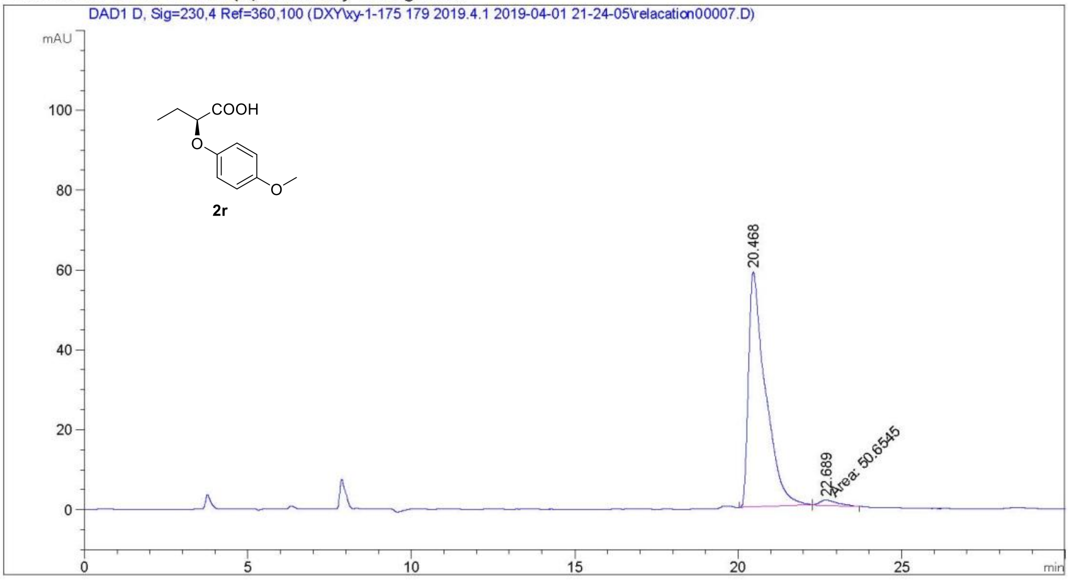


```
    =====================================================================
                             Area Percent Report
    =====================================================================

    Sorted By             :      Signal
    Multiplier            :      1.0000
    Dilution              :      1.0000
    Use Multiplier & Dilution Factor with ISTDs


    Signal 1: DAD1 D, Sig=230,4 Ref=360,100

    Peak RetTime Type  Width     Area      Height     Area
      #   [min]        [min]   [mAU*s]     [mAU]        %
    ----|-------|----|-------|----------|----------|--------|
       1  20.468 BB    0.5024 2119.79517   58.82557  97.6662
       2  22.689 MM    0.6300   50.65445    1.34008   2.3338

    Totals :                  2170.44962   60.16565
```


Supplementary Figure 126. HPLC spectrum of **2r**

Data File D:\CHEM32\...-1-162-2 163-2 2019.10.5 2019-10-05 16-10-28\005-P2-D3-xy-2-143-1.D
Sample Name: xy-2-143-1

===

Acq. Operator : SYSTEM Seq. Line : 5
Acq. Instrument : 1260-DAD Location : P2-D-03
Injection Date : 10/5/2019 19:53:47 Inj : 1
Inj Volume : 1.000 µl
Acq. Method : d:\Chem32\1\Data\XY\xy-1-162-2 163-2 2019.10.5 2019-10-05 16-10-28\DXY-AD3-99-1-0.5mL-100Min.M
Last changed : 10/5/2019 16:06:16 by SYSTEM
Analysis Method : d:\Chem32\1\Data\XY\xy-1-162-2 163-2 2019.10.5 2019-10-05 16-10-28\DXY-AD3-99-1-0.5mL-100Min.M (Sequence Method)
Last changed : 10/24/2019 17:06:00 by SYSTEM
(modified after loading)
Additional Info : Peak(s) manually integrated

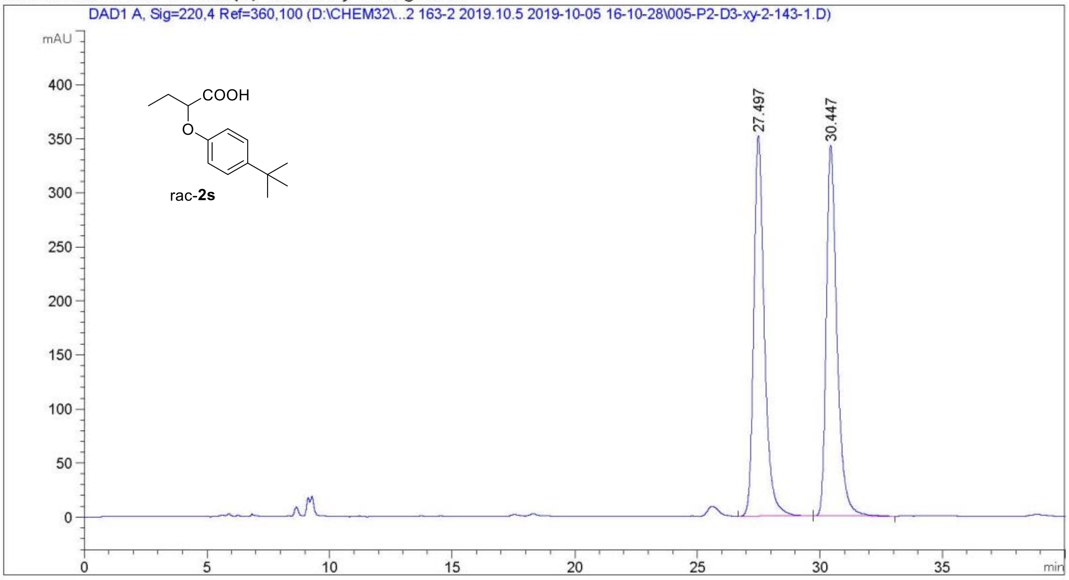

===

Area Percent Report

===

Sorted By : Signal
Multiplier : 1.0000
Dilution : 1.0000
Use Multiplier & Dilution Factor with ISTDs

Signal 1: DAD1 A, Sig=220,4 Ref=360,100

Peak #	RetTime [min]	Type	Width [min]	Area [mAU*s]	Height [mAU]	Area %
1	27.497	BB	0.4526	1.05634e4	351.55801	50.4201
2	30.447	BB	0.4559	1.03873e4	342.40427	49.5799

Totals : 2.09507e4 693.96228

Supplementary Figure 127. HPLC spectrum of rac-**2s**

Data File d:\Chem32\...-1-162-2 163-2 2019.10.5 2019-10-05 16-10-28\006-P2-D4-xy-1-163-2.D
Sample Name: xy-1-163-2

```
=====================================================================
Acq. Operator   : SYSTEM                        Seq. Line :   6
Acq. Instrument : 1260-DAD                       Location :   P2-D-04
Injection Date  : 10/5/2019 21:34:36                  Inj :   1
                                               Inj Volume : 1.000 µl
Acq. Method     : d:\Chem32\1\Data\XY\xy-1-162-2 163-2 2019.10.5 2019-10-05 16-10-28\DXY-AD3-
                  99-1-0.5mL-100Min.M
Last changed    : 10/5/2019 16:06:16 by SYSTEM
Analysis Method : d:\Chem32\1\Data\XY\xy-1-162-2 163-2 2019.10.5 2019-10-05 16-10-28\DXY-AD3-
                  99-1-0.5mL-100Min.M (Sequence Method)
Last changed    : 10/24/2019 17:06:41 by SYSTEM
                  (modified after loading)
Additional Info : Peak(s) manually integrated
```

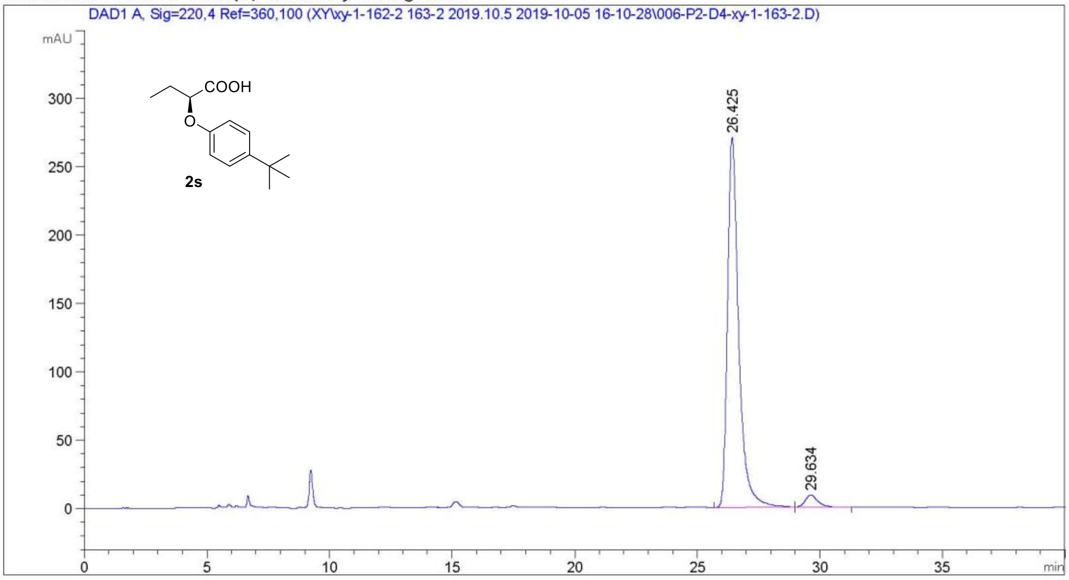


```
=====================================================================
                         Area Percent Report
=====================================================================

Sorted By             :      Signal
Multiplier            :      1.0000
Dilution              :      1.0000
Use Multiplier & Dilution Factor with ISTDs


Signal 1: DAD1 A, Sig=220,4 Ref=360,100
```

Peak #	RetTime [min]	Type	Width [min]	Area [mAU*s]	Height [mAU]	Area %
1	26.425	BB	0.4714	8545.58789	271.33640	96.4186
2	29.634	BB	0.5383	317.42001	8.76901	3.5814

Totals : 8863.00790 280.10541

Supplementary Figure 128. HPLC spectrum of **2s**

Data File D:\CHEMSTA...A\DXY\XY-2-165-2 -2 2019.12.2 2019-12-02 09-35-08\relacation00002.D
Sample Name: xy-2-165-2-rac

===

Acq. Operator : SYSTEM   Seq. Line : 2
Sample Operator : SYSTEM
Acq. Instrument : LC   Location : P1-A-02
Injection Date : 12/2/2019 9:57:17 AM   Inj : 1
Inj Volume : 1.000 µl
Different Inj Volume from Sample Entry! Actual Inj Volume : 2.000 µl
Acq. Method : D:\ChemStation\1\Data\DXY\xy-2-165-2 -2 2019.12.2 2019-12-02 09-35-08\OJ3-99-1-0.5ML-60min.M
Last changed : 12/16/2018 3:39:20 PM by SYSTEM
Analysis Method : D:\ChemStation\1\Data\DXY\xy-2-165-2 -2 2019.12.2 2019-12-02 09-35-08\OJ3-99-1-0.5ML-60min.M (Sequence Method)
Last changed : 12/12/2019 8:00:04 PM by SYSTEM
(modified after loading)
Additional Info : Peak(s) manually integrated

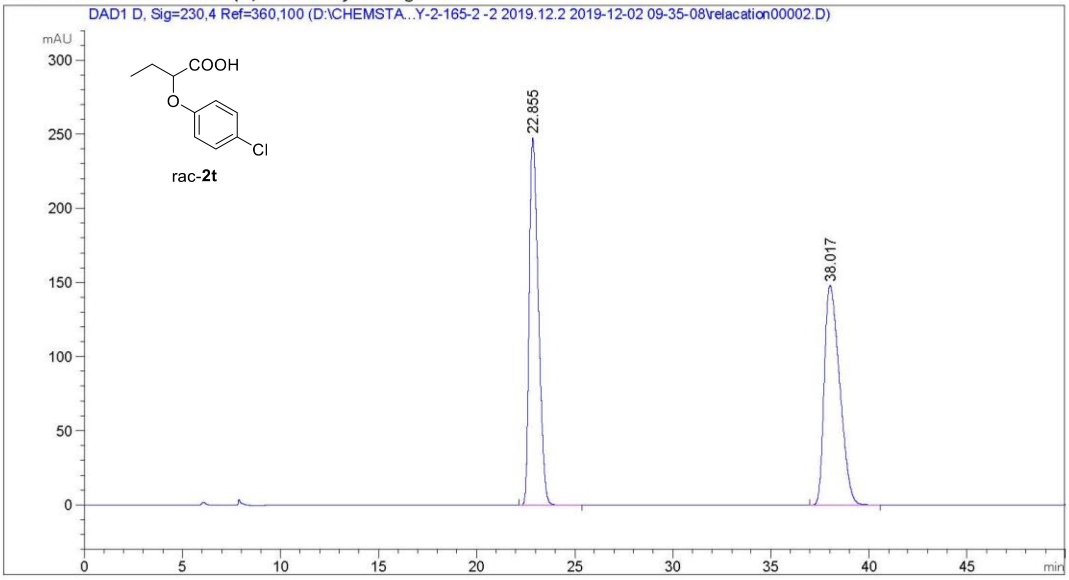

===

Area Percent Report

===

Sorted By : Signal
Multiplier : 1.0000
Dilution : 1.0000
Use Multiplier & Dilution Factor with ISTDs

Signal 1: DAD1 D, Sig=230,4 Ref=360,100

Peak #	RetTime [min]	Type	Width [min]	Area [mAU*s]	Height [mAU]	Area %
1	22.855	BB	0.5077	8025.80371	247.92172	49.9736
2	38.017	BB	0.8577	8034.27100	147.92268	50.0264

Supplementary Figure 129. HPLC spectrum of rac-**2t**

Data File D:\ChemSta...a\DXY\xy-2-165-2 -2 2019.12.2 2019-12-02 09-35-08\relacation00003.D
Sample Name: xy-2-165-2

===

Acq. Operator : SYSTEM Seq. Line : 3
Sample Operator : SYSTEM
Acq. Instrument : LC Location : P1-A-03
Injection Date : 12/2/2019 10:58:10 AM Inj : 1
Inj Volume : 1.000 µl
Different Inj Volume from Sample Entry! Actual Inj Volume : 2.500 µl
Acq. Method : D:\ChemStation\1\Data\DXY\xy-2-165-2 -2 2019.12.2 2019-12-02 09-35-08\OJ3-99-1-0.5ML-60min.M
Last changed : 12/16/2018 3:39:20 PM by SYSTEM
Analysis Method : D:\ChemStation\1\Data\DXY\xy-2-165-2 -2 2019.12.2 2019-12-02 09-35-08\OJ3-99-1-0.5ML-60min.M (Sequence Method)
Last changed : 12/12/2019 8:08:56 PM by SYSTEM
(modified after loading)
Additional Info : Peak(s) manually integrated

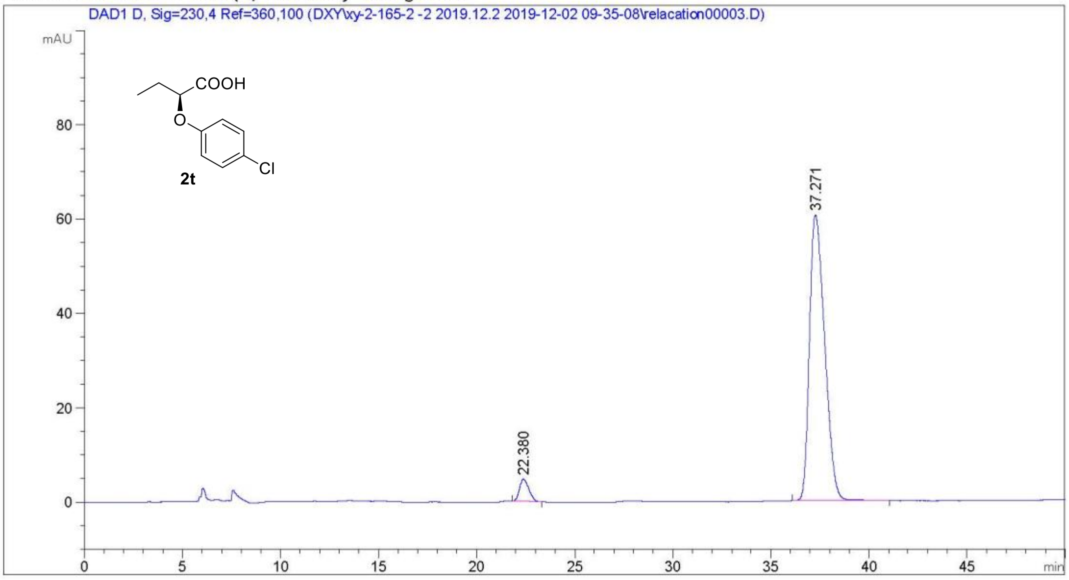

===

Area Percent Report

===

Sorted By : Signal
Multiplier : 1.0000
Dilution : 1.0000
Use Multiplier & Dilution Factor with ISTDs

Signal 1: DAD1 D, Sig=230,4 Ref=360,100

Peak #	RetTime [min]	Type	Width [min]	Area [mAU*s]	Height [mAU]	Area %
1	22.380	BB	0.4804	150.54846	4.69077	4.4199
2	37.271	BB	0.8271	3255.63110	60.57089	95.5801

Supplementary Figure 130. HPLC spectrum of **2t**

Data File D:\ChemSta...\Data\DXY\xy-1-158-4 2019.3.7 2019-03-07 19-51-43\relacation00004.D
Sample Name: xy-1-144-1

===

Acq. Operator : SYSTEM Seq. Line : 4
Sample Operator : SYSTEM
Acq. Instrument : LC Location : P1-D-01
Injection Date : 3/7/2019 8:23:42 PM Inj : 1
Inj Volume : 1.000 µl
Different Inj Volume from Sample Entry! Actual Inj Volume : 2.000 µl
Acq. Method : D:\ChemStation\1\Data\DXY\xy-1-158-4 2019.3.7 2019-03-07 19-51-43\OJ3-97-3-1ML-50min.M
Last changed : 9/20/2018 7:08:47 PM by SYSTEM
Analysis Method : D:\ChemStation\1\Data\DXY\xy-1-158-4 2019.3.7 2019-03-07 19-51-43\OJ3-97-3-1ML-50min.M (Sequence Method)
Last changed : 10/24/2019 5:11:25 PM by SYSTEM
(modified after loading)
Additional Info : Peak(s) manually integrated

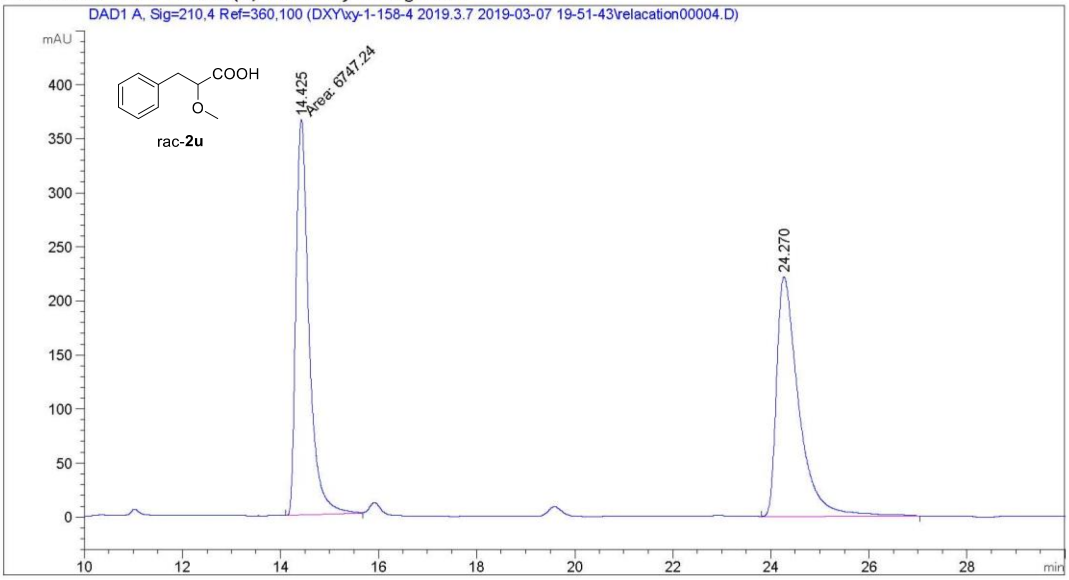

===

Area Percent Report

===

Sorted By : Signal
Multiplier : 1.0000
Dilution : 1.0000
Use Multiplier & Dilution Factor with ISTDs

Signal 1: DAD1 A, Sig=210,4 Ref=360,100

Peak #	RetTime [min]	Type	Width [min]	Area [mAU*s]	Height [mAU]	Area %
1	14.425	MM	0.3075	6747.24414	365.67746	49.3451
2	24.270	BB	0.4646	6926.33936	221.65652	50.6549

Supplementary Figure 131. HPLC spectrum of rac-**2u**

Data File D:\ChemSta...\Data\DXY\xy-1-158-4 2019.3.7 2019-03-07 19-51-43\relacation00005.D
Sample Name: xy-1-158-1

===

Acq. Operator : SYSTEM   Seq. Line : 5
Sample Operator : SYSTEM
Acq. Instrument : LC   Location : P1-D-02
Injection Date : 3/7/2019 9:14:34 PM   Inj : 1
Inj Volume : 1.000 µl
Acq. Method : D:\ChemStation\1\Data\DXY\xy-1-158-4 2019.3.7 2019-03-07 19-51-43\OJ3-97-3-1ML-50min.M
Last changed : 9/20/2018 7:08:47 PM by SYSTEM
Analysis Method : D:\ChemStation\1\Data\DXY\xy-1-158-4 2019.3.7 2019-03-07 19-51-43\OJ3-97-3-1ML-50min.M (Sequence Method)
Last changed : 10/24/2019 5:10:37 PM by SYSTEM
(modified after loading)
Additional Info : Peak(s) manually integrated

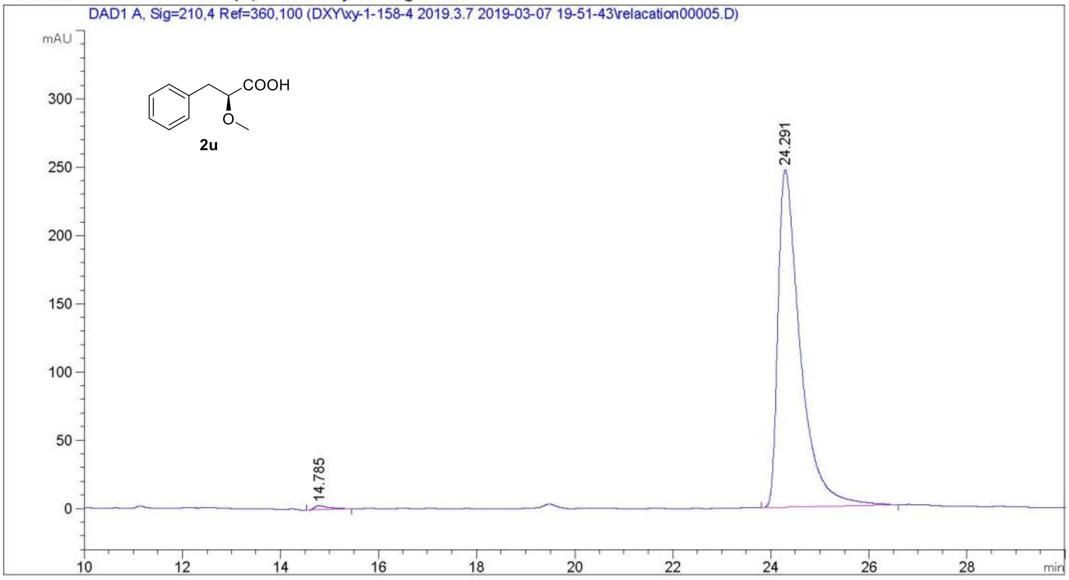

===

Area Percent Report

===

Sorted By : Signal
Multiplier : 1.0000
Dilution : 1.0000
Use Multiplier & Dilution Factor with ISTDs

Signal 1: DAD1 A, Sig=210,4 Ref=360,100

Peak #	RetTime [min]	Type	Width [min]	Area [mAU*s]	Height [mAU]	Area %
1	14.785	BB	0.2932	58.54392	2.90387	0.7483
2	24.291	BB	0.4645	7765.38232	247.15958	99.2517

Totals : 7823.92625 250.06344

Supplementary Figure 132. HPLC spectrum of **2u**

Data File D:\CHEMSTA...DATA\DXY\XY-1-155 2019.2.28 2019-02-28 11-30-11\OnlineEdited--007.D
Sample Name: xy-1-131-1

===

Acq. Operator : SYSTEM   Seq. Line : 7
Sample Operator : SYSTEM
Acq. Instrument : LC   Location : P1-F-01
Injection Date : 2/28/2019 1:58:56 PM   Inj : 1
Inj Volume : 2.000 µl
Acq. Method : D:\ChemStation\1\Data\DXY\xy-1-155 2019.2.28 2019-02-28 11-30-11\AD3-97-3-0.8ML-50min.M
Last changed : 2/28/2019 2:45:51 PM by SYSTEM
(modified after loading)
Analysis Method : D:\ChemStation\1\Data\DXY\xy-1-155 2019.2.28 2019-02-28 11-30-11\AD3-97-3-0.8ML-50min.M (Sequence Method)
Last changed : 10/24/2019 7:33:34 PM by SYSTEM
(modified after loading)
Additional Info : Peak(s) manually integrated

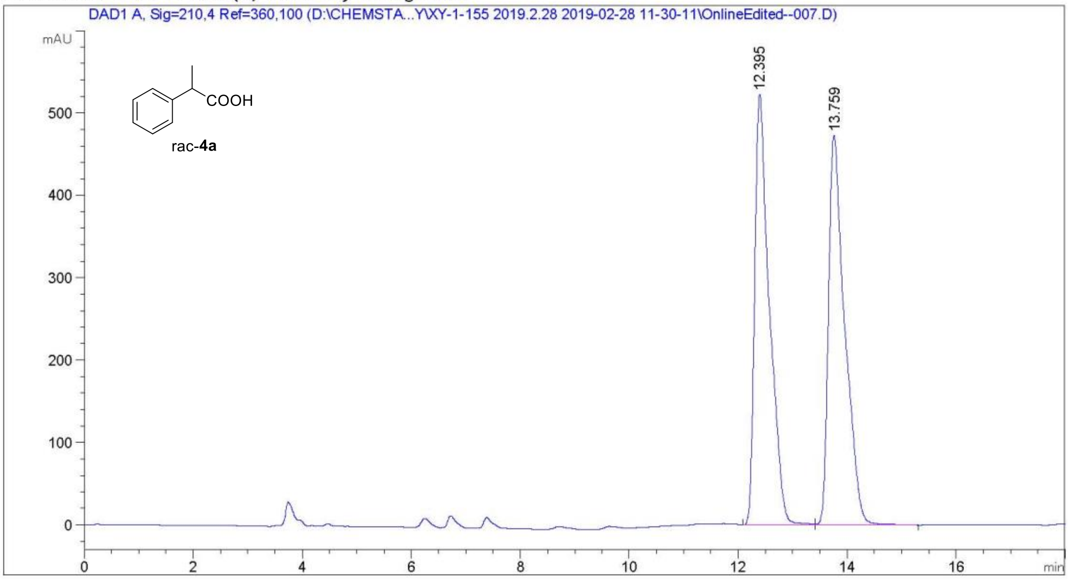

===

Area Percent Report

===

Sorted By : Signal
Multiplier : 1.0000
Dilution : 1.0000
Use Multiplier & Dilution Factor with ISTDs

Signal 1: DAD1 A, Sig=210,4 Ref=360,100

Peak #	RetTime [min]	Type	Width [min]	Area [mAU*s]	Height [mAU]	Area %
1	12.395	BV	0.2678	9749.84180	522.19812	49.9106
2	13.759	VB	0.2974	9784.75977	472.95010	50.0894

Supplementary Figure 133. HPLC spectrum of rac-**4a**

Data File D:\ChemSta...a\DXY\xy-1-155 2019.2.28 2019-02-28 11-30-11\relacation00013--017.D
Sample Name: xy-1-155-10

===

Acq. Operator : SYSTEM Seq. Line : 17
Sample Operator : SYSTEM
Acq. Instrument : LC Location : P1-F-11
Injection Date : 2/28/2019 6:24:53 PM Inj : 1
Inj Volume : 2.000 µl
Acq. Method : D:\ChemStation\1\Data\DXY\xy-1-155 2019.2.28 2019-02-28 11-30-11\AD3-97-3-0.8ML-50min.M
Last changed : 2/28/2019 4:25:43 PM by SYSTEM
Analysis Method : D:\ChemStation\1\Data\DXY\xy-1-155 2019.2.28 2019-02-28 11-30-11\AD3-97-3-0.8ML-50min.M (Sequence Method)
Last changed : 10/24/2019 7:35:42 PM by SYSTEM
(modified after loading)
Additional Info : Peak(s) manually integrated

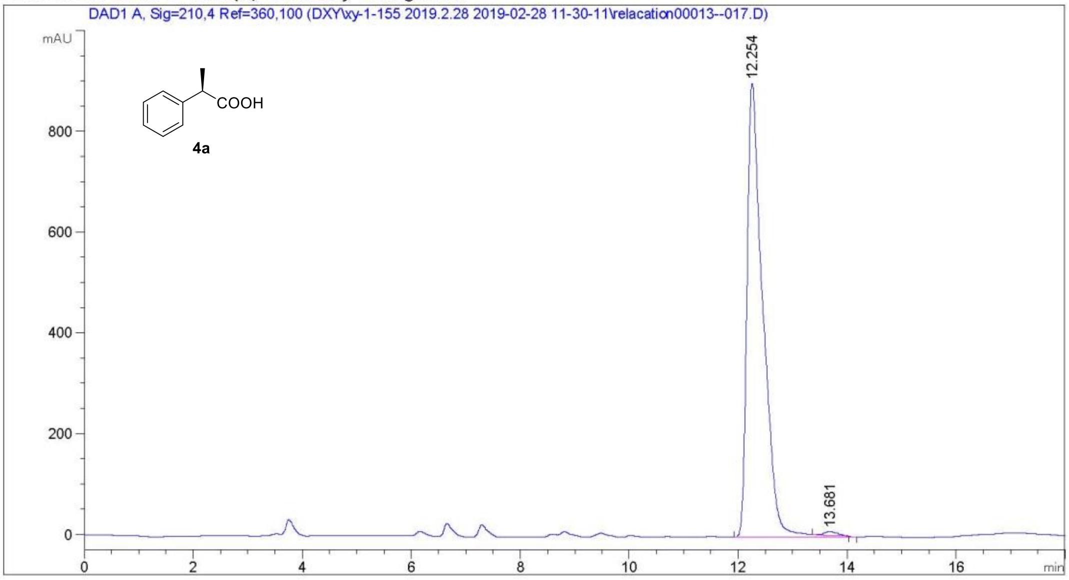

===

Area Percent Report

===

Sorted By : Signal
Multiplier : 1.0000
Dilution : 1.0000
Use Multiplier & Dilution Factor with ISTDs

Signal 1: DAD1 A, Sig=210,4 Ref=360,100

Peak #	RetTime [min]	Type	Width [min]	Area [mAU*s]	Height [mAU]	Area %
1	12.254	BV R	0.2866	1.80952e4	899.76581	99.2474
2	13.681	VB E	0.2778	137.20824	7.70432	0.7526

Totals : 1.82324e4 907.47013

Supplementary Figure 134. HPLC spectrum of **4a**

Data File D:\ChemSta...1\Data\DXY\xy-2-9-3 2019.5.10 2019-05-10 18-58-26\relacation00002.D
Sample Name: xy-2-10-2

===

Acq. Operator : SYSTEM Seq. Line : 2
Sample Operator : SYSTEM
Acq. Instrument : LC Location : P1-C-01
Injection Date : 5/10/2019 7:10:31 PM Inj : 1
Inj Volume : 2.000 µl
Acq. Method : D:\ChemStation\1\Data\DXY\xy-2-9-3 2019.5.10 2019-05-10 18-58-26\AD3-97-3-0.8ML-30min.M
Last changed : 3/4/2019 3:29:55 PM by SYSTEM
Analysis Method : D:\ChemStation\1\Data\DXY\xy-2-9-3 2019.5.10 2019-05-10 18-58-26\AD3-97-3-0.8ML-30min.M (Sequence Method)
Last changed : 10/24/2019 8:21:44 PM by SYSTEM
(modified after loading)
Additional Info : Peak(s) manually integrated

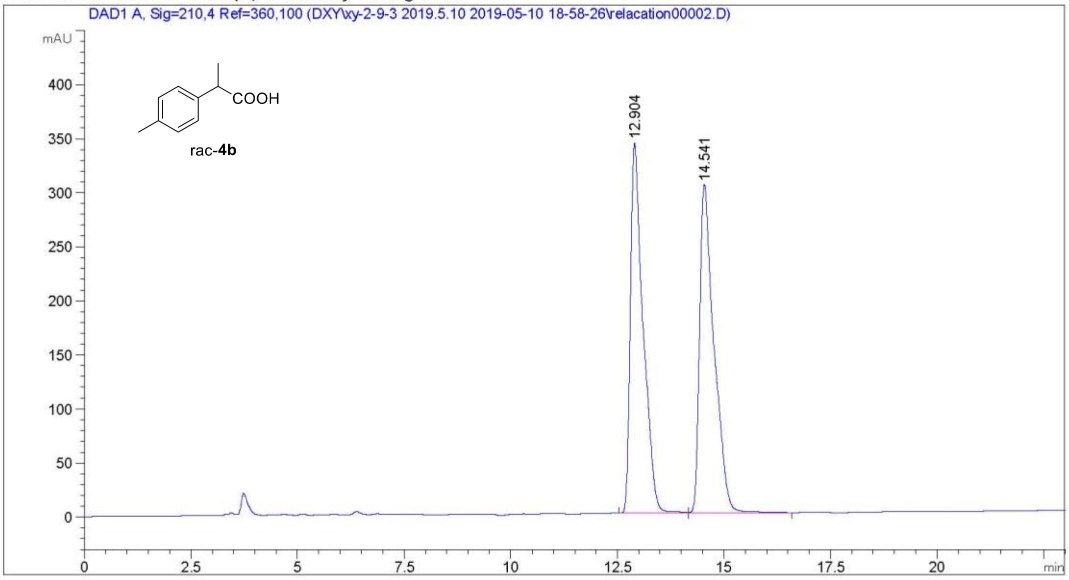

===

Area Percent Report

===

Sorted By : Signal
Multiplier : 1.0000
Dilution : 1.0000
Use Multiplier & Dilution Factor with ISTDs

Signal 1: DAD1 A, Sig=210,4 Ref=360,100

Peak #	RetTime [min]	Type	Width [min]	Area [mAU*s]	Height [mAU]	Area %
1	12.904	BB	0.2942	7049.89111	342.44708	49.9034
2	14.541	BB	0.3343	7077.19727	303.22897	50.0966

Totals : 1.41271e4 645.67606

Supplementary Figure 135. HPLC spectrum of rac-**4b**

Data File D:\ChemSta...1\Data\DXY\xy-2-9-3 2019.5.10 2019-05-10 18-58-26\relacation00003.D
Sample Name: xy-2-9-1

===

Acq. Operator : SYSTEM   Seq. Line : 3
Sample Operator : SYSTEM
Acq. Instrument : LC   Location : P1-C-02
Injection Date : 5/10/2019 7:41:23 PM   Inj : 1
                                           Inj Volume : 2.000 µl
Acq. Method : D:\ChemStation\1\Data\DXY\xy-2-9-3 2019.5.10 2019-05-10 18-58-26\AD3-97-3-0.8ML-30min.M
Last changed : 3/4/2019 3:29:55 PM by SYSTEM
Analysis Method : D:\ChemStation\1\Data\DXY\xy-2-9-3 2019.5.10 2019-05-10 18-58-26\AD3-97-3-0.8ML-30min.M (Sequence Method)
Last changed : 10/24/2019 8:21:03 PM by SYSTEM
(modified after loading)
Additional Info : Peak(s) manually integrated

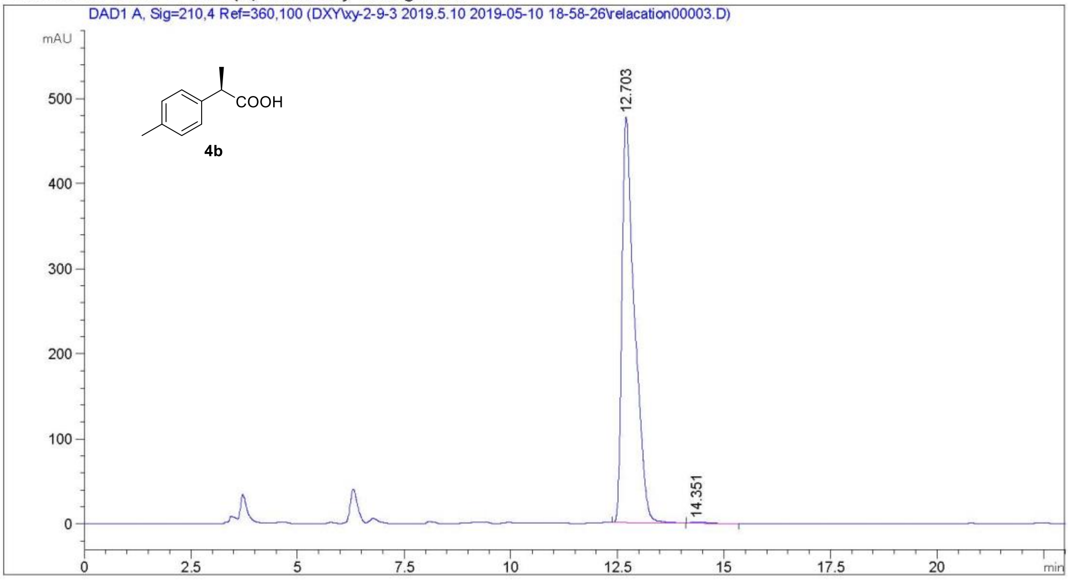

===
Area Percent Report
===

Sorted By : Signal
Multiplier : 1.0000
Dilution : 1.0000
Use Multiplier & Dilution Factor with ISTDs

Signal 1: DAD1 A, Sig=210,4 Ref=360,100

Peak #	RetTime [min]	Type	Width [min]	Area [mAU*s]	Height [mAU]	Area %
1	12.703	BB	0.2901	9732.91504	476.93481	99.6238
2	14.351	BB	0.3597	36.75318	1.40285	0.3762

Totals : 9769.66822 478.33767

Supplementary Figure 136. HPLC spectrum of **4b**

Data File D:\CHEMSTA...1\DATA\DXY\XY-1-194 2019.4.21 2019-04-21 19-54-22\relacation00002.D
Sample Name: xy-1-195-1

===

Acq. Operator	: SYSTEM	Seq. Line :	2
Sample Operator	: SYSTEM		
Acq. Instrument	: LC	Location :	P2-B-01
Injection Date	: 4/21/2019 8:06:24 PM	Inj :	1
		Inj Volume :	2.000 µl

Acq. Method : D:\ChemStation\1\Data\DXY\xy-1-194 2019.4.21 2019-04-21 19-54-22\AD3-97-3-0.8ML-50min.M
Last changed : 9/12/2018 9:16:06 PM by SYSTEM
Analysis Method : D:\ChemStation\1\Data\DXY\xy-1-194 2019.4.21 2019-04-21 19-54-22\AD3-97-3-0.8ML-50min.M (Sequence Method)
Last changed : 10/24/2019 7:38:25 PM by SYSTEM
(modified after loading)
Additional Info : Peak(s) manually integrated

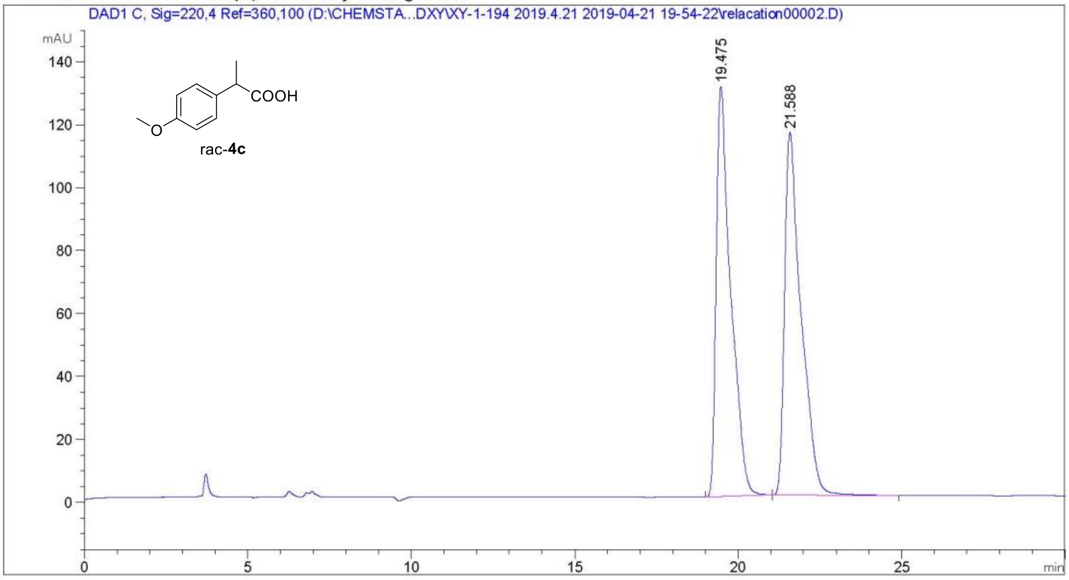

===
Area Percent Report
===

Sorted By : Signal
Multiplier : 1.0000
Dilution : 1.0000
Use Multiplier & Dilution Factor with ISTDs

Signal 1: DAD1 C, Sig=220,4 Ref=360,100

Peak #	RetTime [min]	Type	Width [min]	Area [mAU*s]	Height [mAU]	Area %
1	19.475	BB	0.4360	4001.28198	130.40225	49.7881
2	21.588	BB	0.4965	4035.33496	115.25728	50.2119

Totals : 8036.61694 245.65953

Supplementary Figure 137. HPLC spectrum of rac-**4c**

Data File D:\ChemSta...1\Data\DXY\xy-1-194 2019.4.21 2019-04-21 19-54-22\relacation00003.D
Sample Name: xy-1-194-1

===

Acq. Operator : SYSTEM Seq. Line : 3
Sample Operator : SYSTEM
Acq. Instrument : LC Location : P2-B-02
Injection Date : 4/21/2019 8:57:14 PM Inj : 1
Inj Volume : 2.000 µl
Acq. Method : D:\ChemStation\1\Data\DXY\xy-1-194 2019.4.21 2019-04-21 19-54-22\AD3-97-3-0.8ML-50min.M
Last changed : 9/12/2018 9:16:06 PM by SYSTEM
Analysis Method : D:\ChemStation\1\Data\DXY\xy-1-194 2019.4.21 2019-04-21 19-54-22\AD3-97-3-0.8ML-50min.M (Sequence Method)
Last changed : 10/24/2019 7:39:43 PM by SYSTEM
(modified after loading)
Additional Info : Peak(s) manually integrated

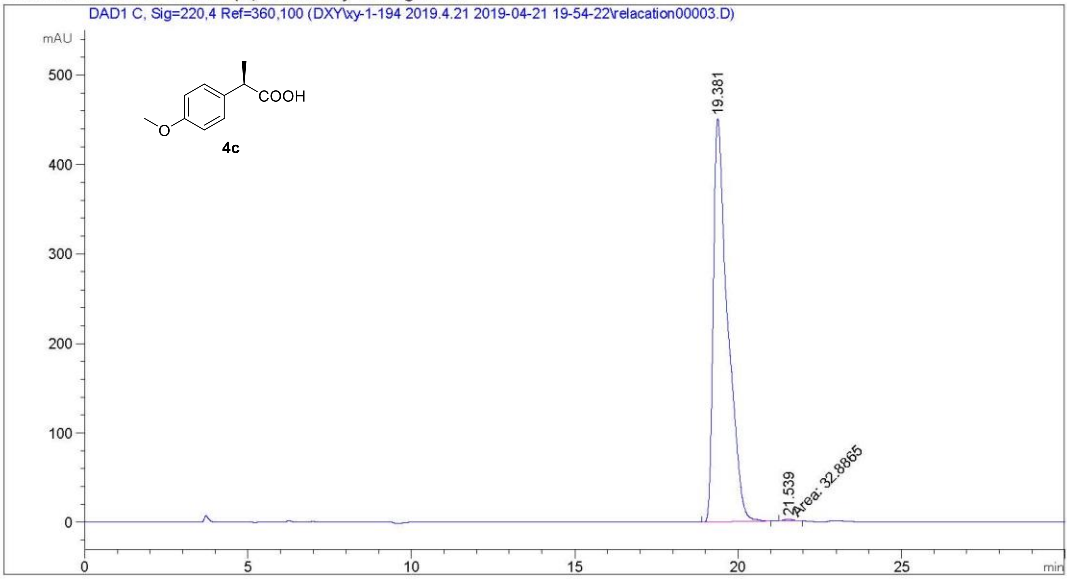

===

Area Percent Report

===

Sorted By : Signal
Multiplier : 1.0000
Dilution : 1.0000
Use Multiplier & Dilution Factor with ISTDs

Signal 1: DAD1 C, Sig=220,4 Ref=360,100

Peak #	RetTime [min]	Type	Width [min]	Area [mAU*s]	Height [mAU]	Area %
1	19.381	BB	0.4350	1.37042e4	450.42941	99.7606
2	21.539	MM	0.3844	32.88648	1.42571	0.2394

Totals : 1.37370e4 451.85512

Supplementary Figure 138. HPLC spectrum of **4c**

Data File D:\CHEMSTA...\1\DATA\DXY\XY-2-72 2019.7.28 2019-07-28 17-23-39\relacation00004.D
Sample Name: xy-2-73-1

===

Acq. Operator : SYSTEM Seq. Line : 4
Sample Operator : SYSTEM
Acq. Instrument : LC Location : P1-E-01
Injection Date : 7/28/2019 5:55:52 PM Inj : 1
Inj Volume : 1.000 µl
Acq. Method : D:\ChemStation\1\Data\DXY\xy-2-72 2019.7.28 2019-07-28 17-23-39\OJ3-97-3-0.8ML-55min.M
Last changed : 10/30/2018 4:44:46 PM by SYSTEM
Analysis Method : D:\ChemStation\1\Data\DXY\xy-2-72 2019.7.28 2019-07-28 17-23-39\OJ3-97-3-0.8ML-55min.M (Sequence Method)
Last changed : 10/24/2019 9:14:46 PM by SYSTEM
(modified after loading)
Additional Info : Peak(s) manually integrated

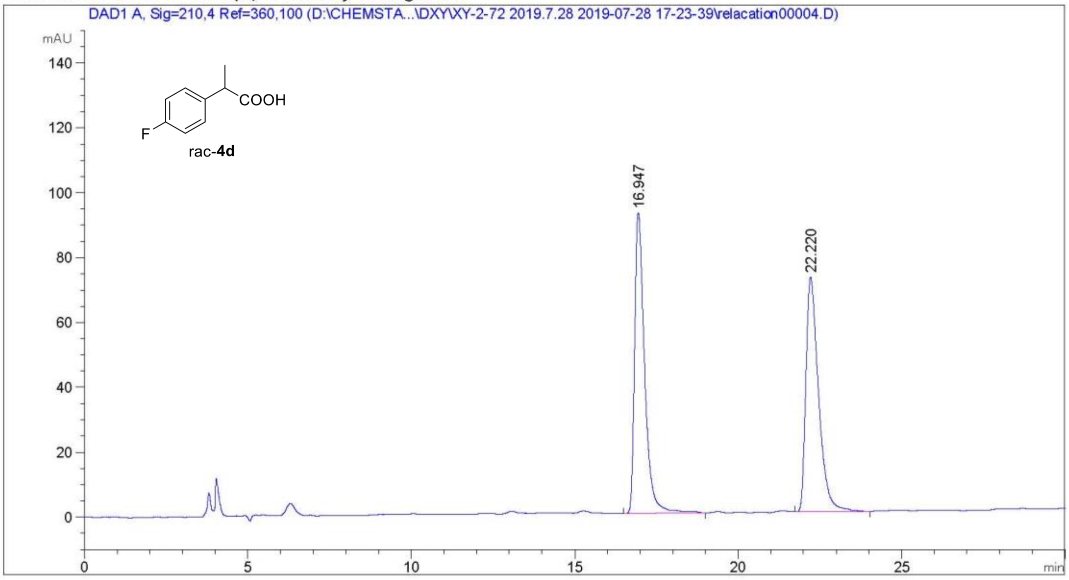

===

Area Percent Report

===

Sorted By : Signal
Multiplier : 1.0000
Dilution : 1.0000
Use Multiplier & Dilution Factor with ISTDs

Signal 1: DAD1 A, Sig=210,4 Ref=360,100

Peak #	RetTime [min]	Type	Width [min]	Area [mAU*s]	Height [mAU]	Area %
1	16.947	BB	0.3187	1979.43115	92.86239	50.4805
2	22.220	BB	0.4009	1941.74768	72.40175	49.5195

Totals : 3921.17883 165.26414

Supplementary Figure 139. HPLC spectrum of rac-**4d**

Data File D:\ChemSta...\1\Data\DXY\xy-2-72 2019.7.28 2019-07-28 17-23-39\relacation00005.D
Sample Name: xy-2-72-1

===

Acq. Operator : SYSTEM Seq. Line : 5
Sample Operator : SYSTEM
Acq. Instrument : LC Location : P1-E-02
Injection Date : 7/28/2019 6:51:44 PM Inj : 1
Inj Volume : 1.000 µl
Acq. Method : D:\ChemStation\1\Data\DXY\xy-2-72 2019.7.28 2019-07-28 17-23-39\OJ3-97-3-0.8ML-55min.M
Last changed : 10/30/2018 4:44:46 PM by SYSTEM
Analysis Method : D:\ChemStation\1\Data\DXY\xy-2-72 2019.7.28 2019-07-28 17-23-39\OJ3-97-3-0.8ML-55min.M (Sequence Method)
Last changed : 10/24/2019 9:15:38 PM by SYSTEM
(modified after loading)
Additional Info : Peak(s) manually integrated

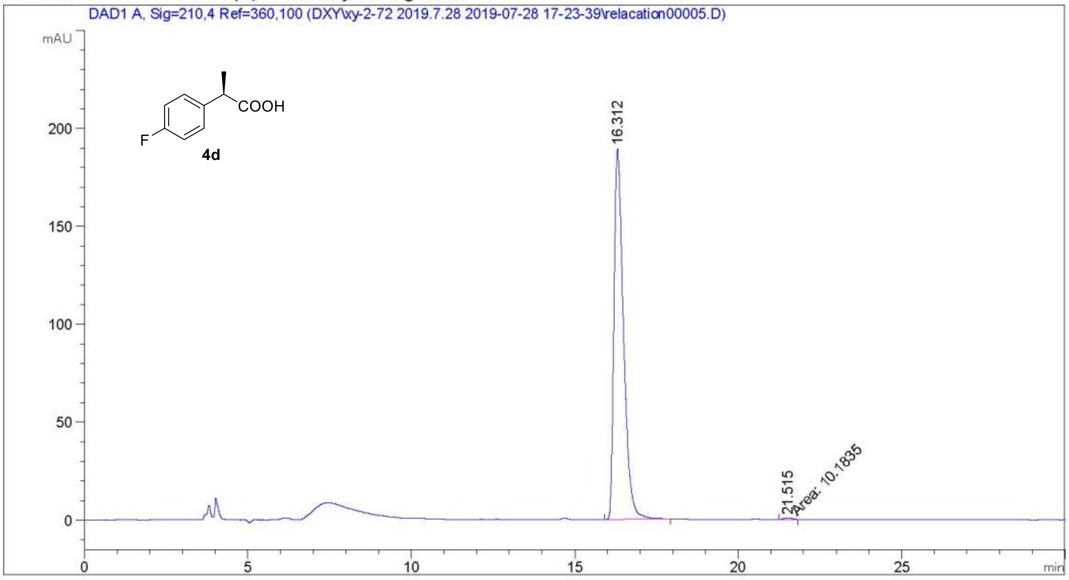

===

Area Percent Report

===

Sorted By : Signal
Multiplier : 1.0000
Dilution : 1.0000
Use Multiplier & Dilution Factor with ISTDs

Signal 1: DAD1 A, Sig=210,4 Ref=360,100

Peak #	RetTime [min]	Type	Width [min]	Area [mAU*s]	Height [mAU]	Area %
1	16.312	BB	0.3053	3812.60425	189.04425	99.7336
2	21.515	MM	0.3293	10.18349	5.15471e-1	0.2664

Totals : 3822.78774 189.55972

Supplementary Figure 140. HPLC spectrum of **4d**

Data File D:\CHEMSTA...\1\DATA\DXY\XY-2-44 2019.6.28 2019-06-28 18-57-45\relacation00003.D
Sample Name: xy-2-45-1

===
Acq. Operator : SYSTEM   Seq. Line : 3
Sample Operator : SYSTEM
Acq. Instrument : LC   Location : P1-C-01
Injection Date : 6/28/2019 7:20:11 PM   Inj : 1
Inj Volume : 2.000 µl
Acq. Method : D:\ChemStation\1\Data\DXY\xy-2-44 2019.6.28 2019-06-28 18-57-45\AD3-97-3-0.8ML-50min.M
Last changed : 9/12/2018 9:16:06 PM by SYSTEM
Analysis Method : D:\ChemStation\1\Data\DXY\xy-2-44 2019.6.28 2019-06-28 18-57-45\AD3-97-3-0.8ML-50min.M (Sequence Method)
Last changed : 10/24/2019 8:57:54 PM by SYSTEM
(modified after loading)
Additional Info : Peak(s) manually integrated

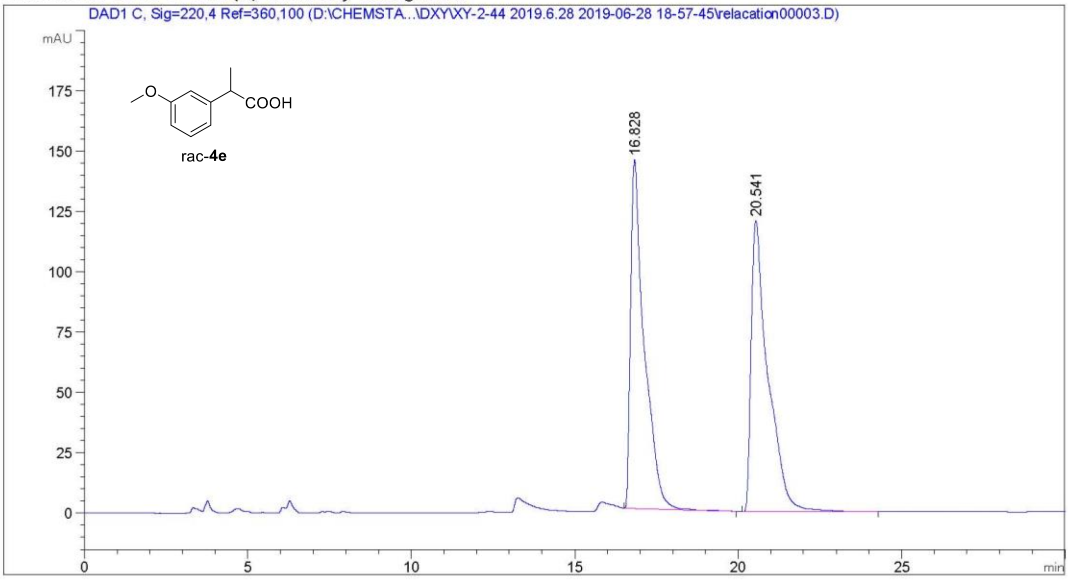

===
Area Percent Report
===

Sorted By : Signal
Multiplier : 1.0000
Dilution : 1.0000
Use Multiplier & Dilution Factor with ISTDs

Signal 1: DAD1 C, Sig=220,4 Ref=360,100

Peak #	RetTime [min]	Type	Width [min]	Area [mAU*s]	Height [mAU]	Area %
1	16.828	BB	0.4044	4164.86426	144.42793	49.2587
2	20.541	BB	0.4977	4290.21631	120.41064	50.7413

Totals : 8455.08057 264.83858

Supplementary Figure 141. HPLC spectrum of rac-**4e**

Data File D:\ChemSta...\1\Data\DXY\xy-2-44 2019.6.28 2019-06-28 18-57-45\relacation00004.D
Sample Name: xy-2-44-1

===

Acq. Operator : SYSTEM Seq. Line : 4
Sample Operator : SYSTEM
Acq. Instrument : LC Location : P1-C-02
Injection Date : 6/28/2019 8:11:02 PM Inj : 1
Inj Volume : 2.000 µl
Acq. Method : D:\ChemStation\1\Data\DXY\xy-2-44 2019.6.28 2019-06-28 18-57-45\AD3-97-3-0.8ML-50min.M
Last changed : 9/12/2018 9:16:06 PM by SYSTEM
Analysis Method : D:\ChemStation\1\Data\DXY\xy-2-44 2019.6.28 2019-06-28 18-57-45\AD3-97-3-0.8ML-50min.M (Sequence Method)
Last changed : 10/24/2019 8:57:54 PM by SYSTEM
(modified after loading)
Additional Info : Peak(s) manually integrated

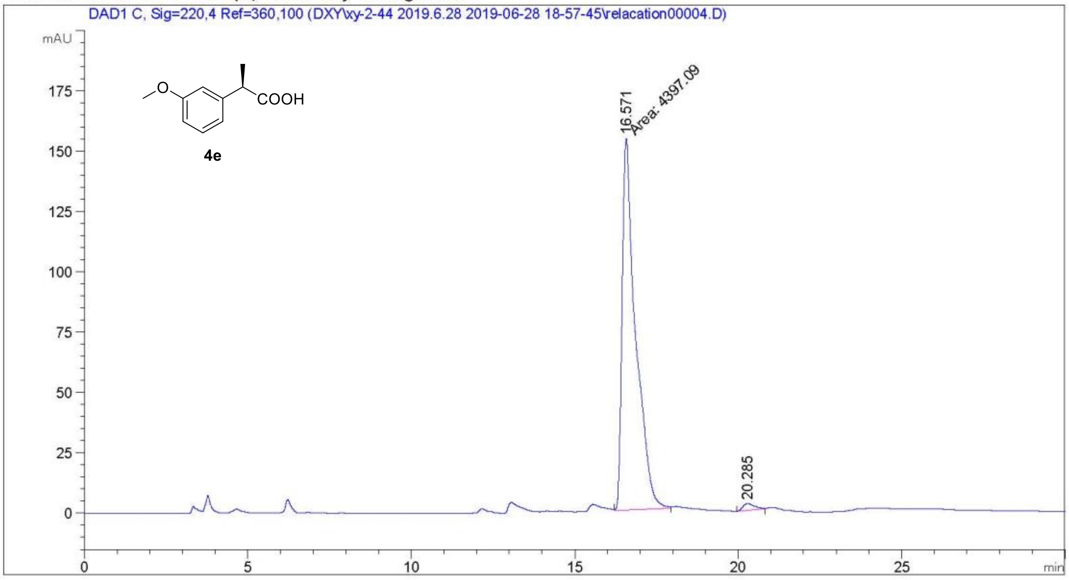

===

Area Percent Report

===

Sorted By : Signal
Multiplier : 1.0000
Dilution : 1.0000
Use Multiplier & Dilution Factor with ISTDs

Signal 1: DAD1 C, Sig=220,4 Ref=360,100

Peak #	RetTime [min]	Type	Width [min]	Area [mAU*s]	Height [mAU]	Area %
1	16.571	MM	0.4762	4397.09277	153.90993	98.5696
2	20.285	BB	0.3439	63.80873	2.71798	1.4304

Totals : 4460.90150 156.62791

Supplementary Figure 142. HPLC spectrum of **4e**

Data File D:\CHEMSTA...\1\DATA\DXY\XY-2-58 2019.7.15 2019-07-15 12-01-05\relacation00004.D
Sample Name: xy-2-59-1

===

Acq. Operator : SYSTEM Seq. Line : 4
Sample Operator : SYSTEM
Acq. Instrument : LC Location : P1-B-01
Injection Date : 7/15/2019 12:35:37 PM Inj : 1
Inj Volume : 2.000 µl
Acq. Method : D:\ChemStation\1\Data\DXY\xy-2-58 2019.7.15 2019-07-15 12-01-05\AD3-97-3-0.8ML-50min.M
Last changed : 9/12/2018 9:16:06 PM by SYSTEM
Analysis Method : D:\ChemStation\1\Data\DXY\xy-2-58 2019.7.15 2019-07-15 12-01-05\AD3-97-3-0.8ML-50min.M (Sequence Method)
Last changed : 10/24/2019 9:02:24 PM by SYSTEM
(modified after loading)
Additional Info : Peak(s) manually integrated

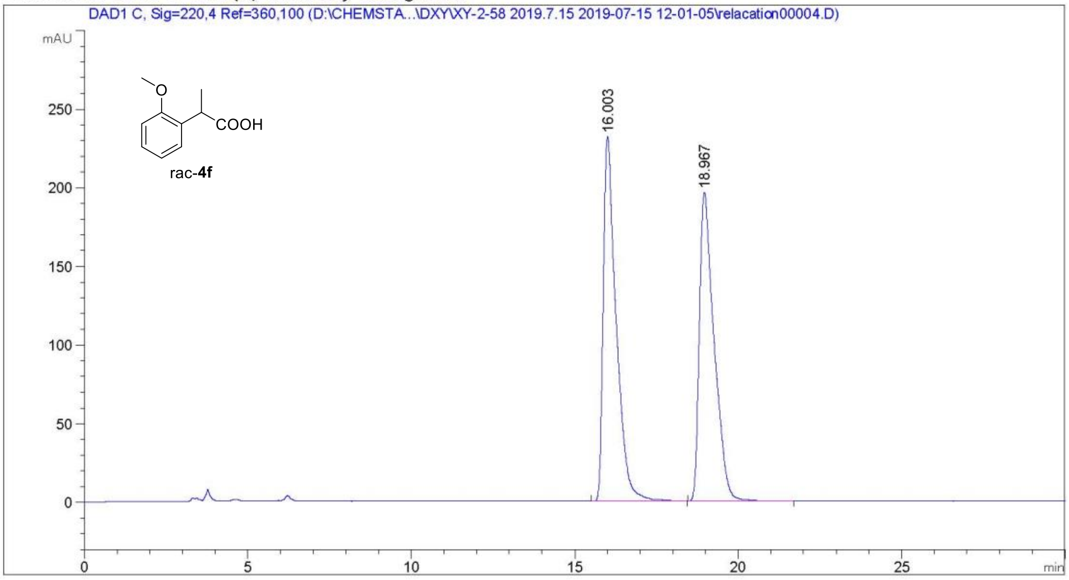

===

Area Percent Report

===

Sorted By : Signal
Multiplier : 1.0000
Dilution : 1.0000
Use Multiplier & Dilution Factor with ISTDs

Signal 1: DAD1 C, Sig=220,4 Ref=360,100

Peak #	RetTime [min]	Type	Width [min]	Area [mAU*s]	Height [mAU]	Area %
1	16.003	BB	0.3916	6312.28906	231.85049	50.8287
2	18.967	BB	0.4514	6106.46240	196.06352	49.1713

Totals : 1.24188e4 427.91402

Supplementary Figure 143. HPLC spectrum of rac-**4f**

Data File D:\ChemSta...\1\Data\DXY\xy-2-58 2019.7.15 2019-07-15 12-01-05\relacation00005.D
Sample Name: xy-2-58-1

```
=====================================================================
Acq. Operator   : SYSTEM                         Seq. Line :   5
Sample Operator : SYSTEM
Acq. Instrument : LC                              Location :   P1-B-02
Injection Date  : 7/15/2019 1:26:28 PM                 Inj :   1
                                                Inj Volume : 2.000 µl
Acq. Method     : D:\ChemStation\1\Data\DXY\xy-2-58 2019.7.15 2019-07-15 12-01-05\AD3-97-3-0.
                  8ML-50min.M
Last changed    : 9/12/2018 9:16:06 PM by SYSTEM
Analysis Method : D:\ChemStation\1\Data\DXY\xy-2-58 2019.7.15 2019-07-15 12-01-05\AD3-97-3-0.
                  8ML-50min.M (Sequence Method)
Last changed    : 10/24/2019 9:03:45 PM by SYSTEM
                  (modified after loading)
Additional Info : Peak(s) manually integrated
```

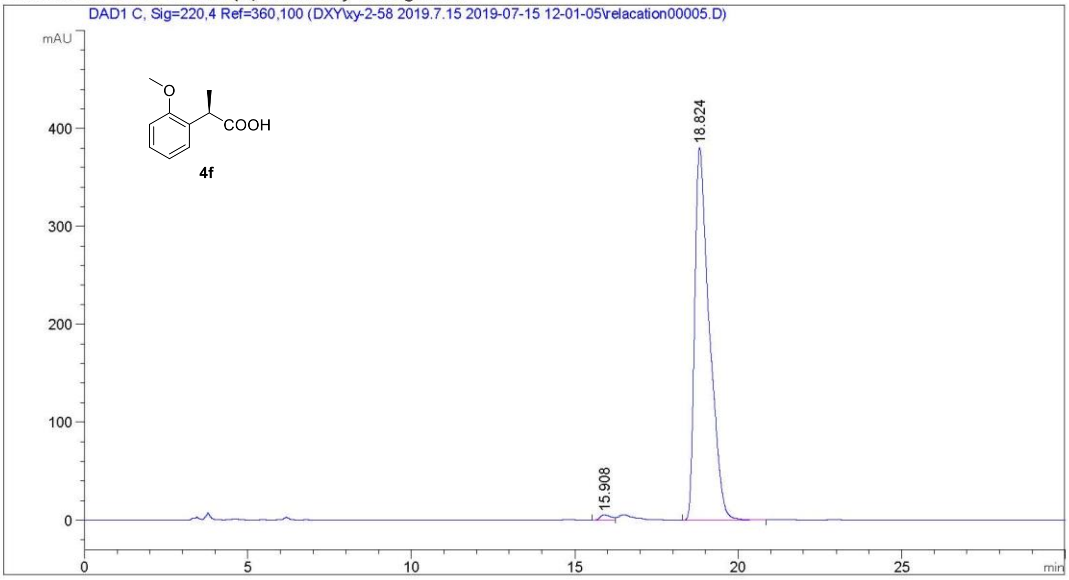


```
=====================================================================
                         Area Percent Report
=====================================================================

Sorted By             :      Signal
Multiplier            :      1.0000
Dilution              :      1.0000
Use Multiplier & Dilution Factor with ISTDs


Signal 1: DAD1 C, Sig=220,4 Ref=360,100
```

Peak #	RetTime [min]	Type	Width [min]	Area [mAU*s]	Height [mAU]	Area %
1	15.908	BV	0.3380	127.90163	5.56765	1.0699
2	18.824	BB	0.4508	1.18262e4	380.31393	98.9301
Totals :				1.19541e4	385.88158	

Supplementary Figure 144. HPLC spectrum of **4f**

Data File D:\CHEMSTA...\DATA\DXY\XY-2-66-2 2019.7.22 2019-07-22 18-12-15\relacation00004.D
Sample Name: xy-2-67-1

===

Acq. Operator : SYSTEM Seq. Line : 4
Sample Operator : SYSTEM
Acq. Instrument : LC Location : P1-B-01
Injection Date : 7/22/2019 6:45:35 PM Inj : 1
Inj Volume : 1.000 µl
Acq. Method : D:\ChemStation\1\Data\DXY\xy-2-66-2 2019.7.22 2019-07-22 18-12-15\OJ3-97-3-0.8ML-55min.M
Last changed : 10/30/2018 4:44:46 PM by SYSTEM
Analysis Method : D:\ChemStation\1\Data\DXY\xy-2-66-2 2019.7.22 2019-07-22 18-12-15\OJ3-97-3-0.8ML-55min.M (Sequence Method)
Last changed : 10/24/2019 9:08:53 PM by SYSTEM
(modified after loading)
Additional Info : Peak(s) manually integrated

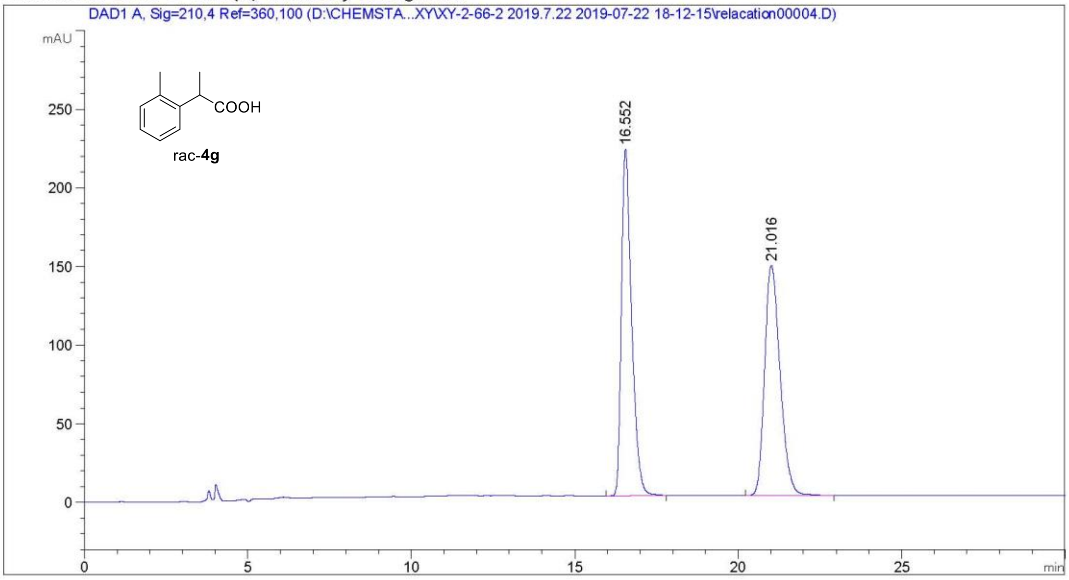

===

Area Percent Report

===

Sorted By : Signal
Multiplier : 1.0000
Dilution : 1.0000
Use Multiplier & Dilution Factor with ISTDs

Signal 1: DAD1 A, Sig=210,4 Ref=360,100

Peak #	RetTime [min]	Type	Width [min]	Area [mAU*s]	Height [mAU]	Area %
1	16.552	BB	0.3243	4764.19580	220.31232	49.8309
2	21.016	BB	0.5066	4796.52295	146.26927	50.1691

Totals : 9560.71875 366.58159

Supplementary Figure 145. HPLC spectrum of rac-**4g**

Data File D:\ChemSta...\Data\DXY\xy-2-66-2 2019.7.22 2019-07-22 18-12-15\relacation00005.D
Sample Name: xy-2-66-1

===

Acq. Operator : SYSTEM Seq. Line : 5
Sample Operator : SYSTEM
Acq. Instrument : LC Location : P1-B-02
Injection Date : 7/22/2019 7:41:25 PM Inj : 1
Inj Volume : 1.000 µl
Acq. Method : D:\ChemStation\1\Data\DXY\xy-2-66-2 2019.7.22 2019-07-22 18-12-15\OJ3-97-3-0.8ML-55min.M
Last changed : 10/30/2018 4:44:46 PM by SYSTEM
Analysis Method : D:\ChemStation\1\Data\DXY\xy-2-66-2 2019.7.22 2019-07-22 18-12-15\OJ3-97-3-0.8ML-55min.M (Sequence Method)
Last changed : 10/24/2019 9:11:16 PM by SYSTEM
(modified after loading)
Additional Info : Peak(s) manually integrated

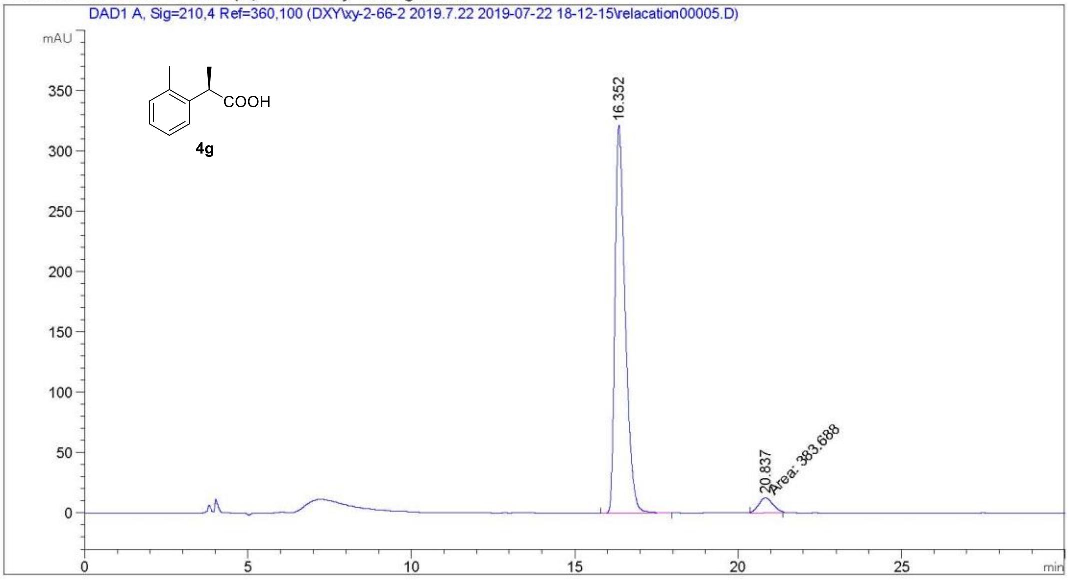

===
Area Percent Report
===

Sorted By : Signal
Multiplier : 1.0000
Dilution : 1.0000
Use Multiplier & Dilution Factor with ISTDs

Signal 1: DAD1 A, Sig=210,4 Ref=360,100

Peak #	RetTime [min]	Type	Width [min]	Area [mAU*s]	Height [mAU]	Area %
1	16.352	BB	0.3269	6968.10205	321.43784	94.7810
2	20.837	MM	0.5129	383.68820	12.46895	5.2190

Totals : 7351.79025 333.90678

Supplementary Figure 146. HPLC spectrum of **4g**

Data File D:\ChemSta...1\Data\DXY\xy-2-4 5 2019.4.30 2019-04-30 12-48-55\relacation00002.D
Sample Name: xy-1-127-1

===

```
Acq. Operator   : SYSTEM                        Seq. Line :   2
Sample Operator : SYSTEM
Acq. Instrument : LC                             Location :   P2-F-04
Injection Date  : 4/30/2019 1:01:27 PM                Inj :   1
                                              Inj Volume : 1.000 µl
Acq. Method     : D:\ChemStation\1\Data\DXY\xy-2-4 5 2019.4.30 2019-04-30 12-48-55\OJ3-97-3-0
                  .8ML-70min.M
Last changed    : 1/22/2019 9:53:20 PM by SYSTEM
Analysis Method : D:\ChemStation\1\Data\DXY\xy-2-4 5 2019.4.30 2019-04-30 12-48-55\OJ3-97-3-0
                  .8ML-70min.M (Sequence Method)
Last changed    : 10/24/2019 8:49:20 PM by SYSTEM
                  (modified after loading)
Additional Info : Peak(s) manually integrated
```

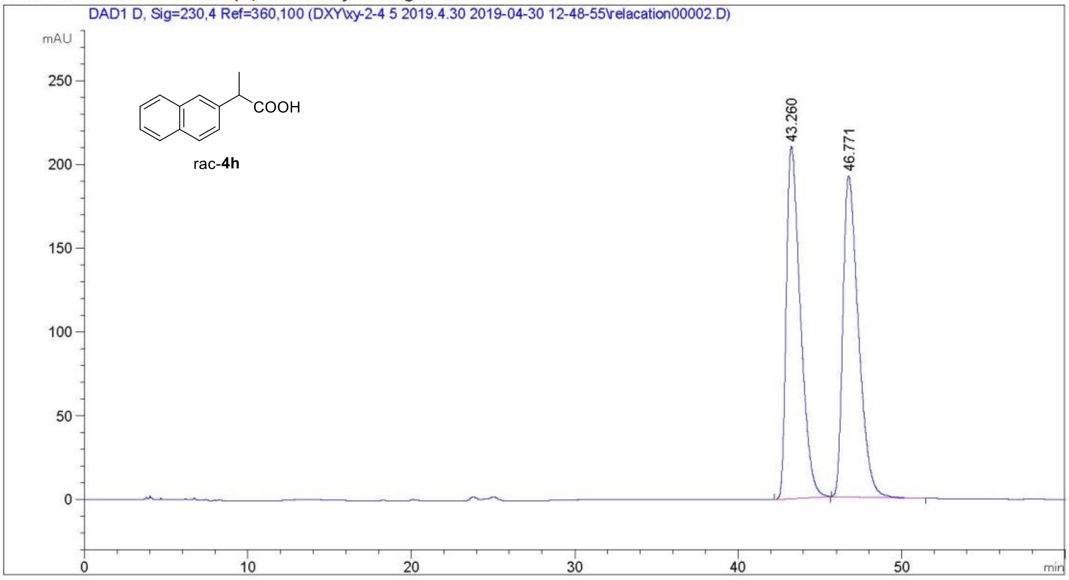

===

Area Percent Report

===

```
Sorted By             :      Signal
Multiplier            :      1.0000
Dilution              :      1.0000
Use Multiplier & Dilution Factor with ISTDs
```

Signal 1: DAD1 D, Sig=230,4 Ref=360,100

Peak #	RetTime [min]	Type	Width [min]	Area [mAU*s]	Height [mAU]	Area %
1	43.260	BB	0.8952	1.25412e4	210.45731	49.7924
2	46.771	BB	0.9995	1.26458e4	191.72777	50.2076

Totals : 2.51871e4 402.18507

Supplementary Figure 147. HPLC spectrum of rac-**4h**

Data File D:\ChemSta...1\Data\DXY\xy-2-4 5 2019.4.30 2019-04-30 12-48-55\relacation00003.D
Sample Name: xy-2-5-1

===

Acq. Operator : SYSTEM Seq. Line : 3
Sample Operator : SYSTEM
Acq. Instrument : LC Location : P2-F-05
Injection Date : 4/30/2019 2:12:16 PM Inj : 1
Inj Volume : 1.000 µl
Acq. Method : D:\ChemStation\1\Data\DXY\xy-2-4 5 2019.4.30 2019-04-30 12-48-55\OJ3-97-3-0.8ML-70min.M
Last changed : 1/22/2019 9:53:20 PM by SYSTEM
Analysis Method : D:\ChemStation\1\Data\DXY\xy-2-4 5 2019.4.30 2019-04-30 12-48-55\OJ3-97-3-0.8ML-70min.M (Sequence Method)
Last changed : 10/24/2019 8:50:18 PM by SYSTEM
(modified after loading)
Additional Info : Peak(s) manually integrated

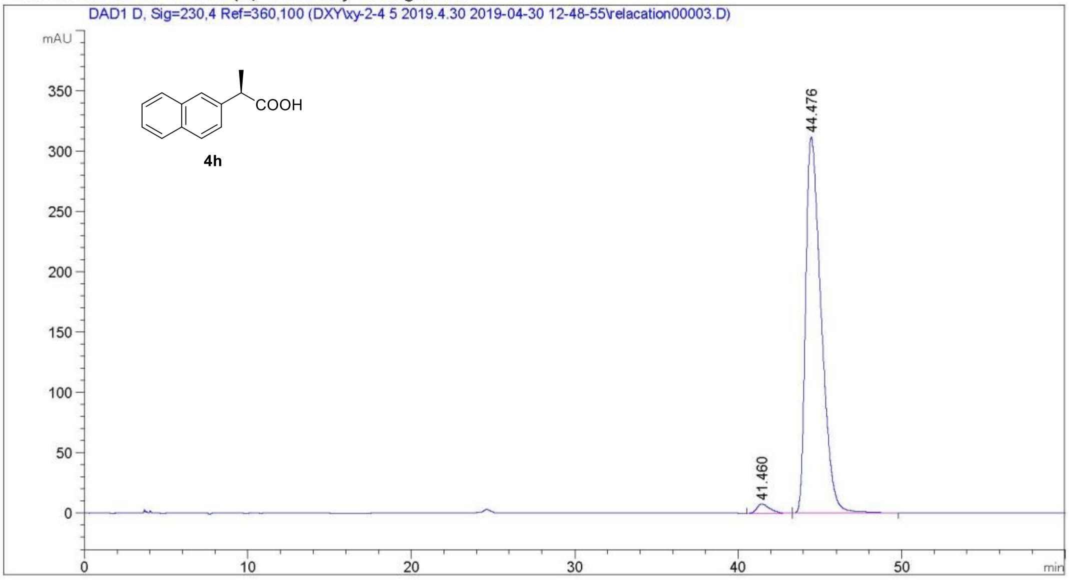

===

Area Percent Report

===

Sorted By : Signal
Multiplier : 1.0000
Dilution : 1.0000
Use Multiplier & Dilution Factor with ISTDs

Signal 1: DAD1 D, Sig=230,4 Ref=360,100

Peak #	RetTime [min]	Type	Width [min]	Area [mAU*s]	Height [mAU]	Area %
1	41.460	BB	0.7701	430.09723	7.76635	2.0533
2	44.476	BB	0.9939	2.05161e4	311.69571	97.9467

Totals : 2.09462e4 319.46206

Supplementary Figure 148. HPLC spectrum of **4h**

Data File D:\ChemStation\1\Data\DXY\xy-2-6 2019.4.29 2019-04-29 22-37-59\relacation00002.D
Sample Name: xy-2-7-1

===

Acq. Operator : SYSTEM   Seq. Line : 2
Sample Operator : SYSTEM
Acq. Instrument : LC   Location : P2-F-02
Injection Date : 4/29/2019 10:50:06 PM   Inj : 1
Inj Volume : 1.000 µl
Acq. Method : D:\ChemStation\1\Data\DXY\xy-2-6 2019.4.29 2019-04-29 22-37-59\OJ3-97-3-0.8ML-30min.M
Last changed : 1/2/2019 10:18:19 PM by SYSTEM
Analysis Method : D:\ChemStation\1\Data\DXY\xy-2-6 2019.4.29 2019-04-29 22-37-59\OJ3-97-3-0.8ML-30min.M (Sequence Method)
Last changed : 10/24/2019 8:42:08 PM by SYSTEM
(modified after loading)
Additional Info : Peak(s) manually integrated

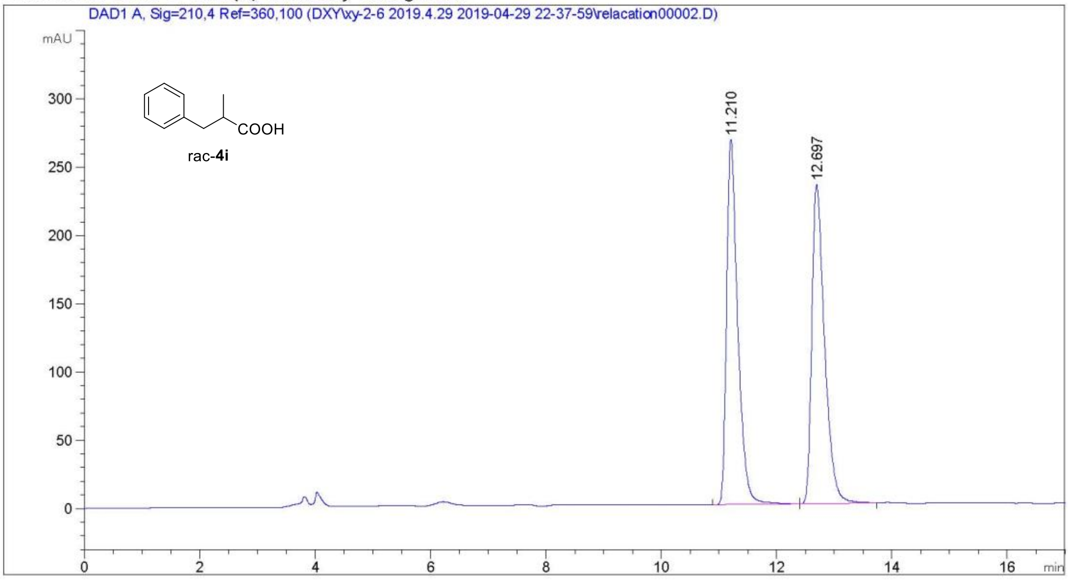

===

Area Percent Report

===

Sorted By : Signal
Multiplier : 1.0000
Dilution : 1.0000
Use Multiplier & Dilution Factor with ISTDs

Signal 1: DAD1 A, Sig=210,4 Ref=360,100

Peak #	RetTime [min]	Type	Width [min]	Area [mAU*s]	Height [mAU]	Area %
1	11.210	BB	0.1980	3574.10645	267.25992	50.1179
2	12.697	BB	0.2278	3557.28833	233.47301	49.8821

Totals : 7131.39478 500.73293

Supplementary Figure 149. HPLC spectrum of rac-**4i**

Data File D:\CHEMSTATION\1\DATA\DXY\XY-2-6 2019.4.29 2019-04-29 22-37-59\relacation00003.D
Sample Name: xy-2-6-1

===
Acq. Operator : SYSTEM Seq. Line : 3
Sample Operator : SYSTEM
Acq. Instrument : LC Location : P2-F-03
Injection Date : 4/29/2019 11:20:57 PM Inj : 1
Inj Volume : 1.000 µl
Acq. Method : D:\ChemStation\1\Data\DXY\xy-2-6 2019.4.29 2019-04-29 22-37-59\OJ3-97-3-0.8ML-30min.M
Last changed : 1/2/2019 10:18:19 PM by SYSTEM
Analysis Method : D:\ChemStation\1\Data\DXY\xy-2-6 2019.4.29 2019-04-29 22-37-59\OJ3-97-3-0.8ML-30min.M (Sequence Method)
Last changed : 10/24/2019 8:40:10 PM by SYSTEM
(modified after loading)
Additional Info : Peak(s) manually integrated

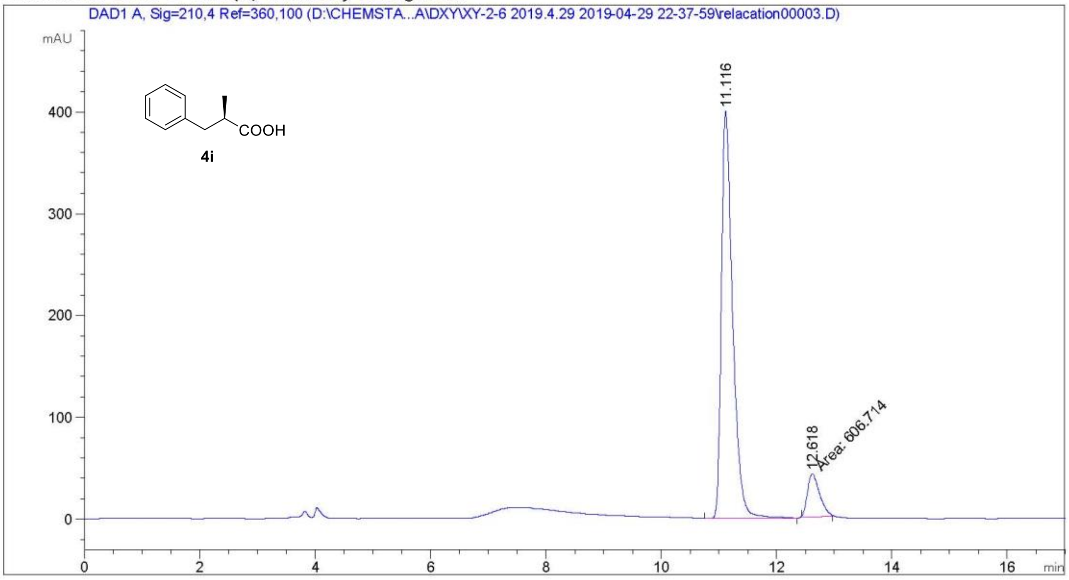

===
Area Percent Report
===

Sorted By : Signal
Multiplier : 1.0000
Dilution : 1.0000
Use Multiplier & Dilution Factor with ISTDs

Signal 1: DAD1 A, Sig=210,4 Ref=360,100

Peak #	RetTime [min]	Type	Width [min]	Area [mAU*s]	Height [mAU]	Area %
1	11.116	BV	0.2009	5379.44678	400.02304	89.8647
2	12.618	MM	0.2402	606.71429	42.09695	10.1353

Totals : 5986.16107 442.12000

Supplementary Figure 150. HPLC spectrum of **4i**

Data File D:\CHEMSTA...DXY\XY-2-169 165-2 2019.11.30 2019-11-30 17-45-21\relacation00007.D
Sample Name: xy-2-25-2

===

Acq. Operator : SYSTEM Seq. Line : 7
Sample Operator : SYSTEM
Acq. Instrument : LC Location : P1-A-04
Injection Date : 11/30/2019 7:37:16 PM Inj : 1
Inj Volume : 1.000 µl
Acq. Method : D:\ChemStation\1\Data\DXY\xy-2-169 165-2 2019.11.30 2019-11-30 17-45-21\OD-3-97-3-0.8ML-30min.M
Last changed : 10/18/2019 3:04:19 PM by SYSTEM
Analysis Method : D:\ChemStation\1\Data\DXY\xy-2-169 165-2 2019.11.30 2019-11-30 17-45-21\OD-3-97-3-0.8ML-30min.M (Sequence Method)
Last changed : 12/12/2019 8:25:58 PM by SYSTEM
(modified after loading)
Additional Info : Peak(s) manually integrated

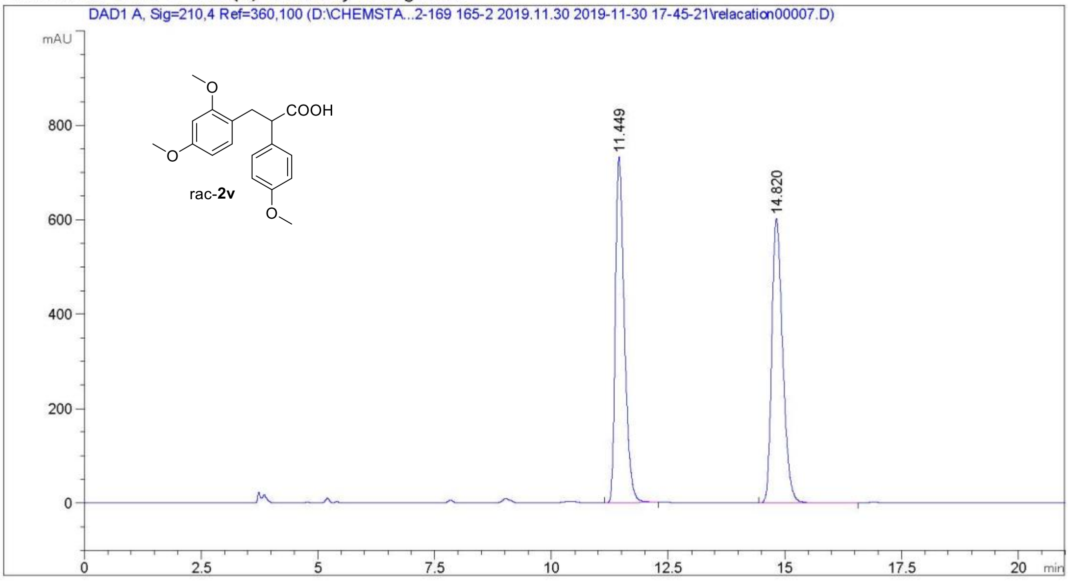

===

Area Percent Report

===

Sorted By : Signal
Multiplier : 1.0000
Dilution : 1.0000
Use Multiplier & Dilution Factor with ISTDs

Signal 1: DAD1 A, Sig=210,4 Ref=360,100

Peak #	RetTime [min]	Type	Width [min]	Area [mAU*s]	Height [mAU]	Area %
1	11.449	BB	0.2032	9747.75098	732.72064	49.8825
2	14.820	BB	0.2497	9793.66797	602.26282	50.1175

Totals : 1.95414e4 1334.98346

Supplementary Figure 151. HPLC spectrum of rac-**2v**

Data File D:\ChemSta...DXY\xy-2-169 165-2 2019.11.30 2019-11-30 17-45-21\relacation00008.D
Sample Name: xy-2-169-2

===

Acq. Operator : SYSTEM Seq. Line : 8
Sample Operator : SYSTEM
Acq. Instrument : LC Location : P1-A-05
Injection Date : 11/30/2019 8:08:06 PM Inj : 1
Inj Volume : 1.000 µl
Acq. Method : D:\ChemStation\1\Data\DXY\xy-2-169 165-2 2019.11.30 2019-11-30 17-45-21\OD-3-97-3-0.8ML-30min.M
Last changed : 10/18/2019 3:04:19 PM by SYSTEM
Analysis Method : D:\ChemStation\1\Data\DXY\xy-2-169 165-2 2019.11.30 2019-11-30 17-45-21\OD-3-97-3-0.8ML-30min.M (Sequence Method)
Last changed : 12/12/2019 8:26:55 PM by SYSTEM
(modified after loading)
Additional Info : Peak(s) manually integrated

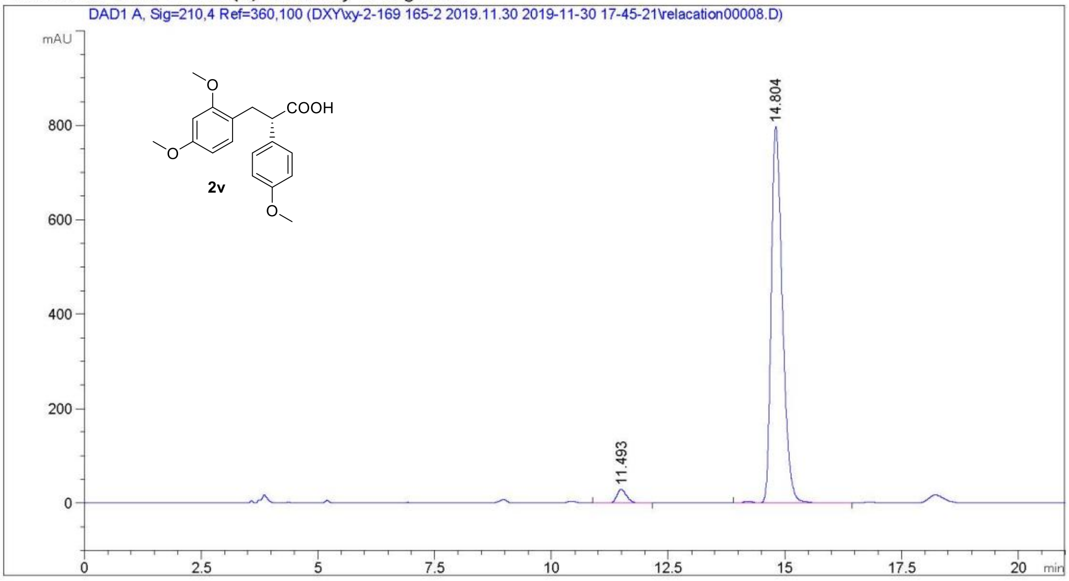

===

Area Percent Report

===

Sorted By : Signal
Multiplier : 1.0000
Dilution : 1.0000
Use Multiplier & Dilution Factor with ISTDs

Signal 1: DAD1 A, Sig=210,4 Ref=360,100

Peak #	RetTime [min]	Type	Width [min]	Area [mAU*s]	Height [mAU]	Area %
1	11.493	BB	0.2232	429.61826	29.27245	3.1407
2	14.804	VB R	0.2553	1.32493e4	796.68097	96.8593

Totals : 1.36789e4 825.95342

Supplementary Figure 152. HPLC spectrum of **2v**

Data File D:\CHEMSTA...TA\DXY\XY-2-137 -3 2019.10.23 2019-10-23 16-33-02\relacation00002.D
Sample Name: xy-2-23-1

===

Acq. Operator : SYSTEM Seq. Line : 2
Sample Operator : SYSTEM
Acq. Instrument : LC Location : P1-A-02
Injection Date : 10/23/2019 4:45:26 PM Inj : 1
Inj Volume : 1.000 µl
Different Inj Volume from Sample Entry! Actual Inj Volume : 5.000 µl
Acq. Method : D:\ChemStation\1\Data\DXY\xy-2-137 -3 2019.10.23 2019-10-23 16-33-02\OJ3-97 -3-0.8ML-60min.M
Last changed : 10/23/2019 5:12:50 PM by SYSTEM (modified after loading)
Analysis Method : D:\ChemStation\1\Data\DXY\xy-2-137 -3 2019.10.23 2019-10-23 16-33-02\OJ3-97 -3-0.8ML-60min.M (Sequence Method)
Last changed : 10/24/2019 4:16:07 PM by SYSTEM (modified after loading)
Additional Info : Peak(s) manually integrated

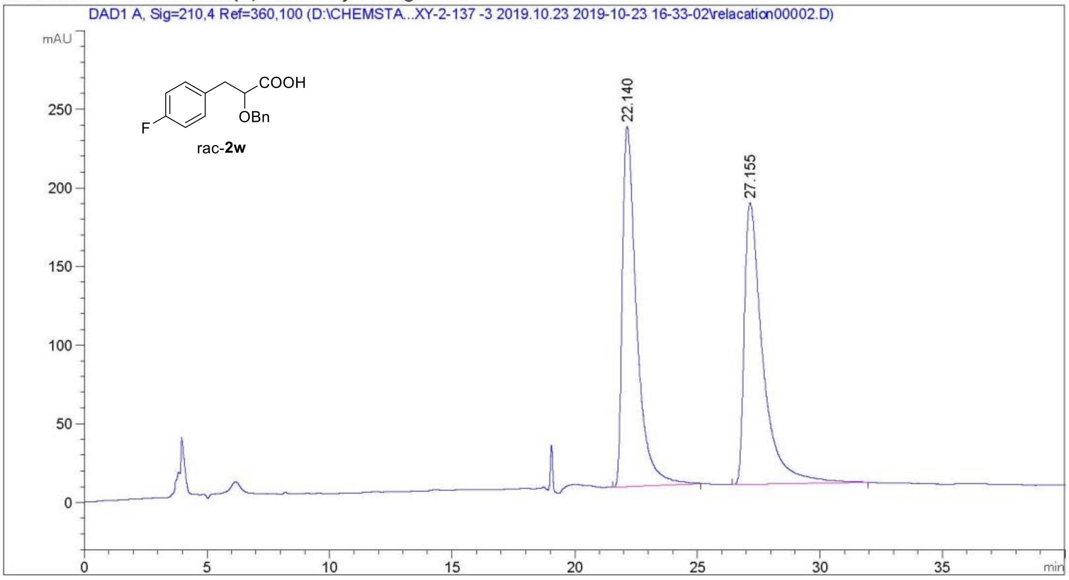

===

Area Percent Report

===

Sorted By : Signal
Multiplier : 1.0000
Dilution : 1.0000
Use Multiplier & Dilution Factor with ISTDs

Signal 1: DAD1 A, Sig=210,4 Ref=360,100

Peak #	RetTime [min]	Type	Width [min]	Area [mAU*s]	Height [mAU]	Area %
1	22.140	BB	0.6041	9269.76367	229.09258	49.5618
2	27.155	BB	0.7706	9433.67090	179.06207	50.4382

Supplementary Figure 153. HPLC spectrum of rac-**2w**

```
Data File D:\ChemSta...ta\DXY\xy-2-137 -3 2019.10.23 2019-10-23 16-33-02\relacation00003.D
Sample Name: xy2-137-1

    =====================================================================
    Acq. Operator   : SYSTEM                        Seq. Line :   3
    Sample Operator : SYSTEM
    Acq. Instrument : LC                             Location :   P1-A-03
    Injection Date  : 10/23/2019 5:36:19 PM               Inj :   1
                                                   Inj Volume : 1.000 µl
    Different Inj Volume from Sample Entry!  Actual Inj Volume : 2.000 µl
    Acq. Method     : D:\ChemStation\1\Data\DXY\xy-2-137 -3 2019.10.23 2019-10-23 16-33-02\OJ3-97
                      -3-0.8ML-60min.M
    Last changed    : 10/23/2019 5:12:50 PM by SYSTEM
    Analysis Method : D:\ChemStation\1\Data\DXY\xy-2-137 -3 2019.10.23 2019-10-23 16-33-02\OJ3-97
                      -3-0.8ML-60min.M (Sequence Method)
    Last changed    : 10/24/2019 4:17:37 PM by SYSTEM
                      (modified after loading)
    Additional Info : Peak(s) manually integrated
```

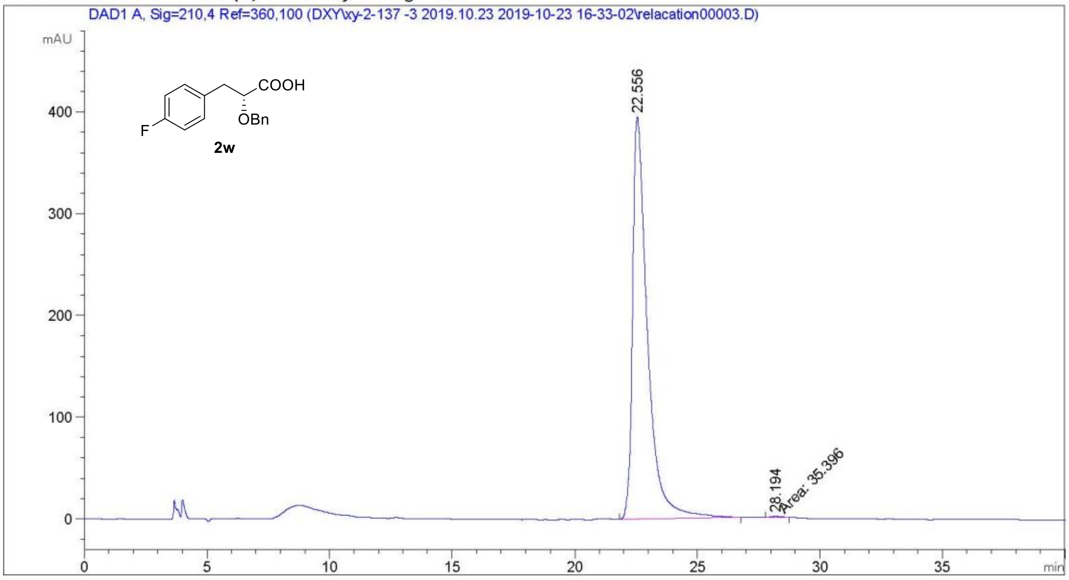


```
    =====================================================================
                             Area Percent Report
    =====================================================================

    Sorted By             :      Signal
    Multiplier            :      1.0000
    Dilution              :      1.0000
    Use Multiplier & Dilution Factor with ISTDs


    Signal 1: DAD1 A, Sig=210,4 Ref=360,100

    Peak RetTime Type  Width     Area      Height     Area
      #   [min]        [min]   [mAU*s]     [mAU]        %
    ----|-------|----|-------|----------|----------|--------|
       1  22.556 BB    0.6176 1.66441e4  395.00250  99.7878
       2  28.194 MM    0.5808   35.39597    1.01579   0.2122
```


Supplementary Figure 154. HPLC spectrum of **2w**

Data File D:\CHEMSTA...TA\DXY\XY-2-13-8 -2 2019.11.4 2019-11-04 18-47-27\relacation00002.D
Sample Name: xy-2-146-1

===

Acq. Operator : SYSTEM   Seq. Line : 2
Sample Operator : SYSTEM
Acq. Instrument : LC   Location : P1-A-02
Injection Date : 11/4/2019 7:08:58 PM   Inj : 1
Inj Volume : 1.000 µl
Acq. Method : D:\ChemStation\1\Data\DXY\xy-2-13-8 -2 2019.11.4 2019-11-04 18-47-27\AS3-92-8-0.9ML-25min.M
Last changed : 4/18/2019 8:14:31 PM by SYSTEM
Analysis Method : D:\ChemStation\1\Data\DXY\xy-2-13-8 -2 2019.11.4 2019-11-04 18-47-27\AS3-92-8-0.9ML-25min.M (Sequence Method)
Last changed : 12/12/2019 8:19:25 PM by SYSTEM
(modified after loading)
Additional Info : Peak(s) manually integrated

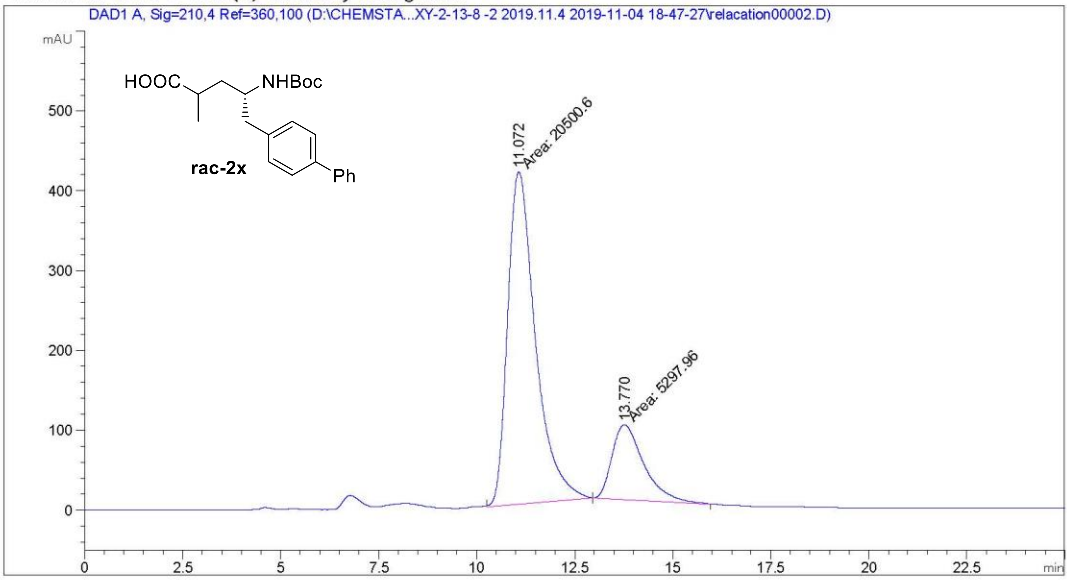

===

Area Percent Report

===

Sorted By : Signal
Multiplier : 1.0000
Dilution : 1.0000
Use Multiplier & Dilution Factor with ISTDs

Signal 1: DAD1 A, Sig=210,4 Ref=360,100

Peak #	RetTime [min]	Type	Width [min]	Area [mAU*s]	Height [mAU]	Area %
1	11.072	MM	0.8202	2.05006e4	416.58246	79.4641
2	13.770	MM	0.9404	5297.96240	93.89396	20.5359

Totals : 2.57986e4 510.47642

Supplementary Figure 155. HPLC spectrum of rac-**2x**

Data File D:\ChemSta...ta\DXY\xy-2-13-8 -2 2019.11.4 2019-11-04 18-47-27\relacation00003.D
Sample Name: xy-2-13-8

===

Acq. Operator : SYSTEM Seq. Line : 3
Sample Operator : SYSTEM
Acq. Instrument : LC Location : P1-A-03
Injection Date : 11/4/2019 7:34:48 PM Inj : 1
Inj Volume : 1.000 µl
Acq. Method : D:\ChemStation\1\Data\DXY\xy-2-13-8 -2 2019.11.4 2019-11-04 18-47-27\AS3-92 -8-0.9ML-25min.M
Last changed : 4/18/2019 8:14:31 PM by SYSTEM
Analysis Method : D:\ChemStation\1\Data\DXY\xy-2-13-8 -2 2019.11.4 2019-11-04 18-47-27\AS3-92 -8-0.9ML-25min.M (Sequence Method)
Last changed : 12/12/2019 8:21:27 PM by SYSTEM
(modified after loading)
Additional Info : Peak(s) manually integrated

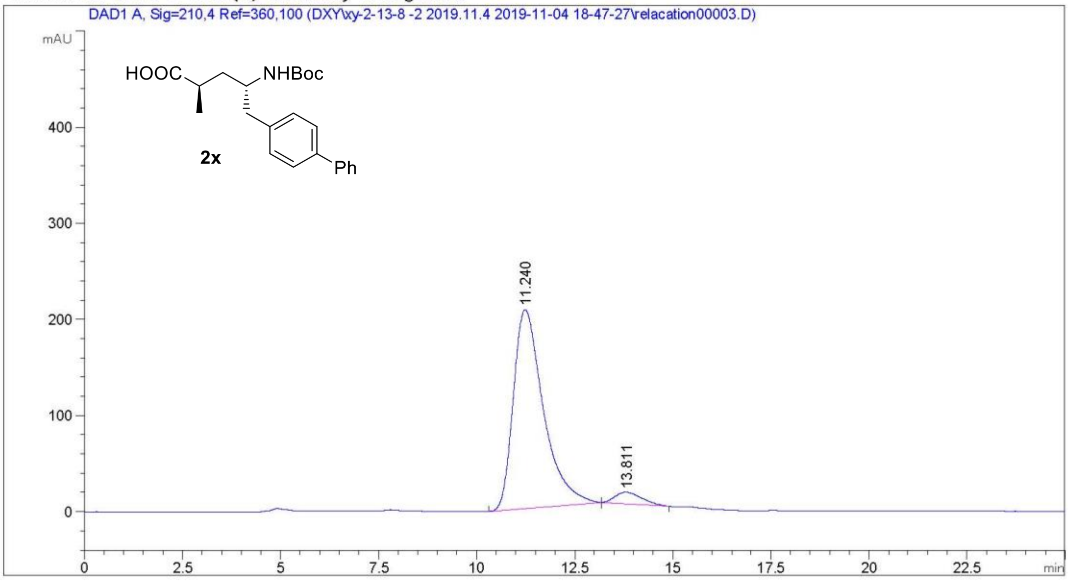

===

Area Percent Report

===

Sorted By : Signal
Multiplier : 1.0000
Dilution : 1.0000
Use Multiplier & Dilution Factor with ISTDs

Signal 1: DAD1 A, Sig=210,4 Ref=360,100

Peak #	RetTime [min]	Type	Width [min]	Area [mAU*s]	Height [mAU]	Area %
1	11.240	BB	0.7981	1.09974e4	206.90541	95.0694
2	13.811	BB	0.6035	570.36212	11.97923	4.9306

Totals : 1.15678e4 218.88464

Supplementary Figure 156. HPLC spectrum of **2x**

Data File D:\CHEMSTA...\DATA\DXY\XY-2-54-2 2019.7.11 2019-07-11 22-48-40\relacation00004.D
Sample Name: xy-2-55-1

===

Acq. Operator : SYSTEM   Seq. Line : 4
Sample Operator : SYSTEM
Acq. Instrument : LC   Location : P1-F-01
Injection Date : 7/11/2019 11:21:28 PM   Inj : 1
                    Inj Volume : 1.000 µl
Acq. Method : D:\ChemStation\1\Data\DXY\xy-2-54-2 2019.7.11 2019-07-11 22-48-40\OJ3-97-3-0.8ML-120min.M
Last changed : 7/11/2019 10:42:30 PM by SYSTEM
Analysis Method : D:\ChemStation\1\Data\DXY\xy-2-54-2 2019.7.11 2019-07-11 22-48-40\OJ3-97-3-0.8ML-120min.M (Sequence Method)
Last changed : 10/24/2019 9:17:25 PM by SYSTEM
(modified after loading)
Additional Info : Peak(s) manually integrated

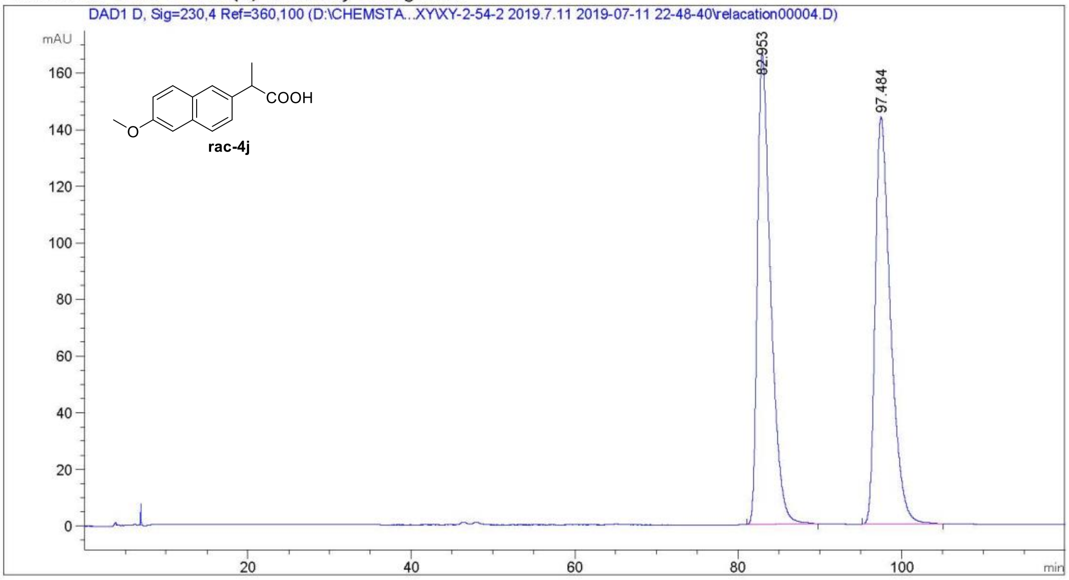

===

Area Percent Report

===

Sorted By : Signal
Multiplier : 1.0000
Dilution : 1.0000
Use Multiplier & Dilution Factor with ISTDs

Signal 1: DAD1 D, Sig=230,4 Ref=360,100

Peak #	RetTime [min]	Type	Width [min]	Area [mAU*s]	Height [mAU]	Area %
1	82.953	BB	1.6510	1.86244e4	165.99751	49.9089
2	97.484	BB	1.8857	1.86923e4	143.87833	50.0911

Totals : 3.73167e4 309.87584

Supplementary Figure 157. HPLC spectrum of rac-**4j**

Data File D:\ChemSta...\Data\DXY\xy-2-54-2 2019.7.11 2019-07-11 22-48-40\relacation00005.D
Sample Name: xy-2-54-1

===

Acq. Operator : SYSTEM Seq. Line : 5
Sample Operator : SYSTEM
Acq. Instrument : LC Location : P1-F-02
Injection Date : 7/12/2019 1:22:21 AM Inj : 1
Inj Volume : 1.000 µl
Acq. Method : D:\ChemStation\1\Data\DXY\xy-2-54-2 2019.7.11 2019-07-11 22-48-40\OJ3-97-3-0.8ML-120min.M
Last changed : 7/11/2019 10:42:30 PM by SYSTEM
Analysis Method : D:\ChemStation\1\Data\DXY\xy-2-54-2 2019.7.11 2019-07-11 22-48-40\OJ3-97-3-0.8ML-120min.M (Sequence Method)
Last changed : 10/24/2019 9:17:25 PM by SYSTEM
(modified after loading)
Additional Info : Peak(s) manually integrated

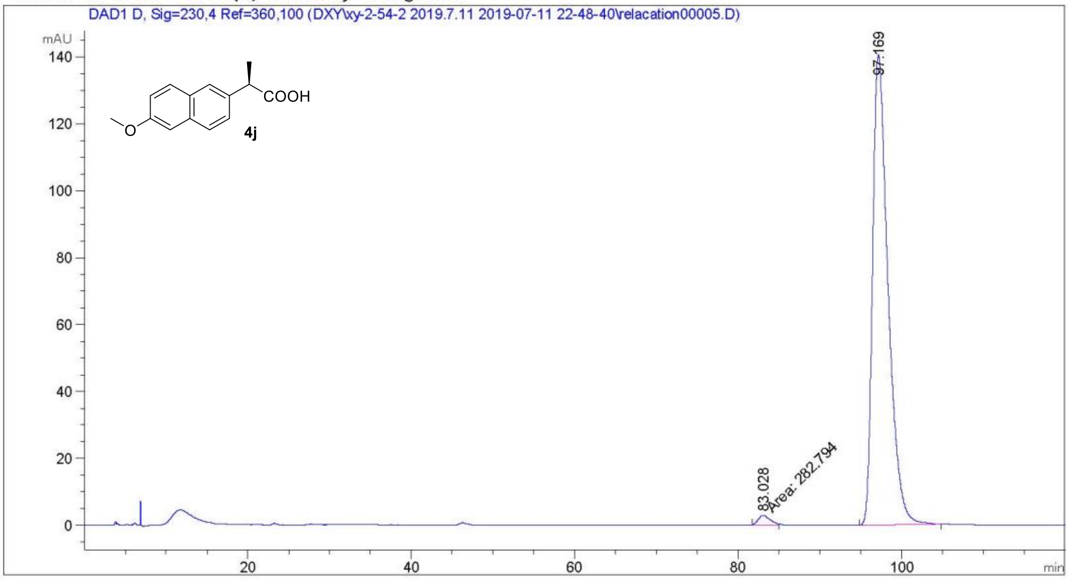

===

Area Percent Report

===

Sorted By : Signal
Multiplier : 1.0000
Dilution : 1.0000
Use Multiplier & Dilution Factor with ISTDs

Signal 1: DAD1 D, Sig=230,4 Ref=360,100

Peak #	RetTime [min]	Type	Width [min]	Area [mAU*s]	Height [mAU]	Area %
1	83.028	MM	1.6817	282.79428	2.80264	1.5119
2	97.169	BB	1.9213	1.84212e4	140.71799	98.4881

Totals : 1.87040e4 143.52063

Supplementary Figure 158. HPLC spectrum of **4j**

Data File d:\Chem32\...ta\XY\xy-2-21-3 2019.6.12 2019-06-12 21-12-45\003-P1-E1-xy-2-21-1.D
Sample Name: xy-2-21-1

===

Acq. Operator : SYSTEM Seq. Line : 3
Acq. Instrument : 1260-DAD Location : P1-E-01
Injection Date : 6/12/2019 21:35:08 Inj : 1
Inj Volume : 1.000 µl
Acq. Method : d:\Chem32\1\Data\XY\xy-2-21-3 2019.6.12 2019-06-12 21-12-45\DXY-AD3-99-1-0.5mL-30Min.M
Last changed : 6/12/2019 21:09:33 by SYSTEM
Analysis Method : d:\Chem32\1\Data\XY\xy-2-21-3 2019.6.12 2019-06-12 21-12-45\DXY-AD3-99-1-0.5mL-30Min.M (Sequence Method)
Last changed : 10/24/2019 20:36:29 by SYSTEM
(modified after loading)
Additional Info : Peak(s) manually integrated

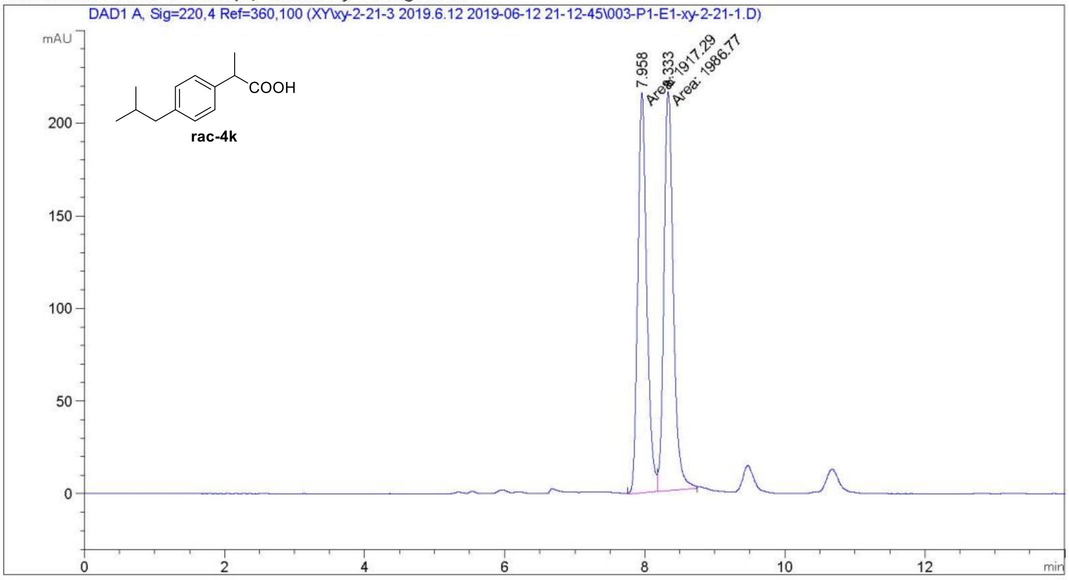

===

Area Percent Report

===

Sorted By : Signal
Multiplier : 1.0000
Dilution : 1.0000
Use Multiplier & Dilution Factor with ISTDs

Signal 1: DAD1 A, Sig=220,4 Ref=360,100

Peak #	RetTime [min]	Type	Width [min]	Area [mAU*s]	Height [mAU]	Area %
1	7.958	MF	0.1478	1917.28955	216.19675	49.1102
2	8.333	FM	0.1537	1986.76721	215.49268	50.8898

Totals : 3904.05676 431.68942

Supplementary Figure 159. HPLC spectrum of rac-**4k**

Data File d:\Chem32\...ta\XY\xy-2-21-3 2019.6.12 2019-06-12 21-12-45\004-P1-E2-xy-2-20-1.D
Sample Name: xy-2-20-1

===

Acq. Operator : SYSTEM Seq. Line : 4
Acq. Instrument : 1260-DAD Location : P1-E-02
Injection Date : 6/12/2019 22:05:59 Inj : 1
Inj Volume : 1.000 µl
Acq. Method : d:\Chem32\1\Data\XY\xy-2-21-3 2019.6.12 2019-06-12 21-12-45\DXY-AD3-99-1-0.5mL-30Min.M
Last changed : 6/12/2019 21:09:33 by SYSTEM
Analysis Method : d:\Chem32\1\Data\XY\xy-2-21-3 2019.6.12 2019-06-12 21-12-45\DXY-AD3-99-1-0.5mL-30Min.M (Sequence Method)
Last changed : 10/24/2019 20:37:41 by SYSTEM
(modified after loading)
Additional Info : Peak(s) manually integrated

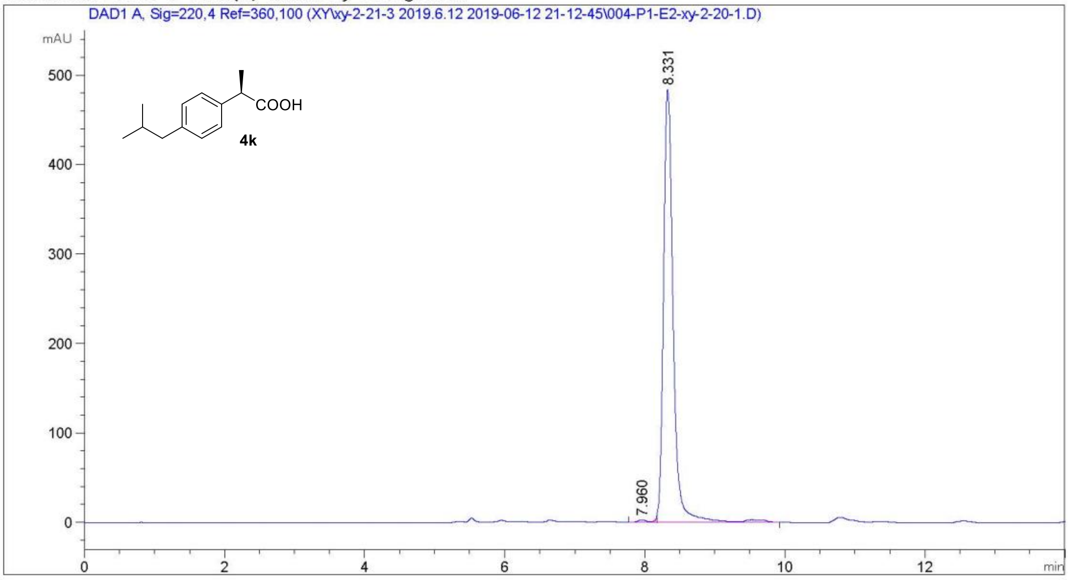

===

Area Percent Report

===

Sorted By : Signal
Multiplier : 1.0000
Dilution : 1.0000
Use Multiplier & Dilution Factor with ISTDs

Signal 1: DAD1 A, Sig=220,4 Ref=360,100

Peak #	RetTime [min]	Type	Width [min]	Area [mAU*s]	Height [mAU]	Area %
1	7.960	BV E	0.1419	27.04151	2.76871	0.5768
2	8.331	VV R	0.1453	4661.32178	484.28815	99.4232

Totals : 4688.36329 487.05686

Supplementary Figure 160. HPLC spectrum of **4k**

Data File D:\CHEM32\...CID-20190731 2019-08-08 11-01-16\Sc-Artemisinic Acid-2.7um-296-11.D
Sample Name: Sc-Artemisinic Acid-2.7um-296-11

===

Acq. Operator : SYSTEM Seq. Line : 1
Acq. Instrument : 1260 Location : P1-D1
Injection Date : 8/8/2019 11:05:28 Inj : 1
Inj Volume : 10.000 µl
Different Inj Volume from Sample Entry! Actual Inj Volume : 2.000 µl
Acq. Method : D:\Chem32\DATA\Sc-2\Sc-Artemisinic Acid-20190731 2019-08-08 11-01-16\Sc_2.7um C18_0.1%FA_lowgradient.M
Last changed : 8/8/2019 11:01:16 by SYSTEM
Analysis Method : D:\Chem32\DATA\Sc-2\Sc-Artemisinic Acid-20190731 2019-08-08 11-01-16\Sc_2.7um C18_0.1%FA_lowgradient.M (Sequence Method)
Last changed : 11/29/2019 9:19:48 by SYSTEM
(modified after loading)
Additional Info : Peak(s) manually integrated

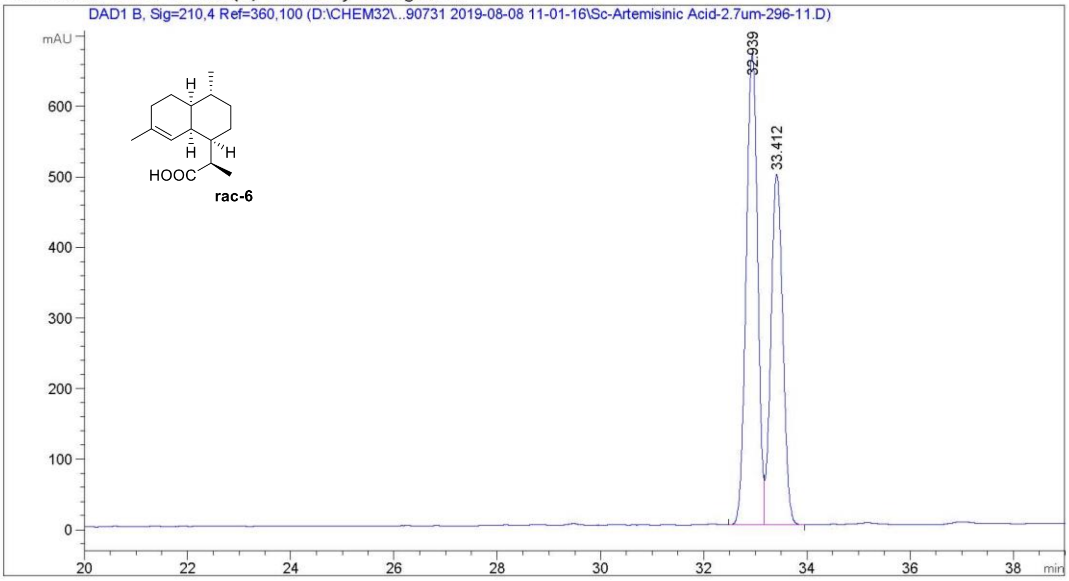

===

Area Percent Report

===

Sorted By : Signal
Multiplier : 1.0000
Dilution : 1.0000
Do not use Multiplier & Dilution Factor with ISTDs

Signal 1: DAD1 B, Sig=210,4 Ref=360,100

Peak #	RetTime [min]	Type	Width [min]	Area [mAU*s]	Height [mAU]	Area %
1	32.939	BV	0.2448	1.03301e4	666.64160	56.5153
2	33.412	VB	0.2507	7948.34326	496.64804	43.4847

Totals : 1.82785e4 1163.28964

Supplementary Figure 161. HPLC spectrum of rac-**6**

Data File D:\Chem32\...iaoye-20191128 2019-11-28 23-00-38\Sc-Artemisinic Acid-2.7um-0071.D
Sample Name: Sc-Artemisinic Acid-2.7um-007

===

Acq. Operator : SYSTEM Seq. Line : 2
Acq. Instrument : 1260 Location : P1-D1
Injection Date : 11/28/2019 23:44:04 Inj : 2
Inj Volume : 10.000 µl
Different Inj Volume from Sample Entry! Actual Inj Volume : 2.000 µl
Acq. Method : D:\Chem32\DATA\Sc-2\Sc-Artemisinic Acid-Xiaoye-20191128 2019-11-28 23-00-38 \Sc_2.7um C18_0.1%FA_lowgradient.M
Last changed : 11/28/2019 23:00:38 by SYSTEM
Analysis Method : D:\Chem32\DATA\Sc-2\Sc-Artemisinic Acid-Xiaoye-20191128 2019-11-28 23-00-38 \Sc_2.7um C18_0.1%FA_lowgradient.M (Sequence Method)
Last changed : 12/3/2019 14:17:01 by SYSTEM
(modified after loading)
Additional Info : Peak(s) manually integrated

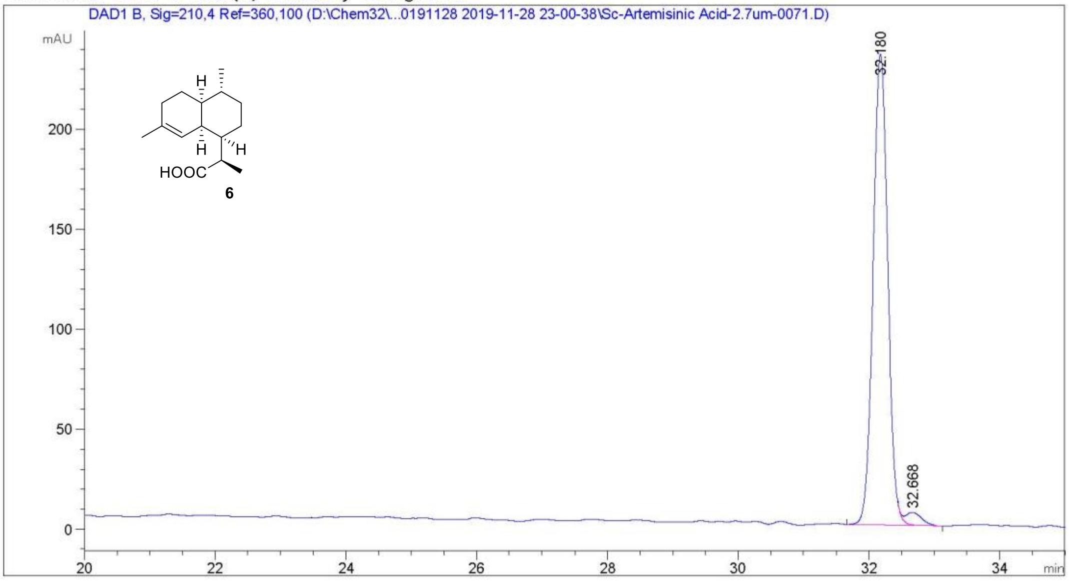

===
Area Percent Report
===

Sorted By : Signal
Multiplier : 1.0000
Dilution : 1.0000
Do not use Multiplier & Dilution Factor with ISTDs

Signal 1: DAD1 B, Sig=210,4 Ref=360,100

Peak #	RetTime [min]	Type	Width [min]	Area [mAU*s]	Height [mAU]	Area %
1	32.180	BV R	0.2414	3658.33374	235.28957	97.2285
2	32.668	VB E	0.2459	104.27972	6.33941	2.7715

Totals : 3762.61346 241.62897

Supplementary Figure 162. HPLC spectrum of **6**

Supplementary References

1 Yang, S. *et al.* Enantioselective iridium-catalyzed hydrogenation of α-arylcinnamic acids and synthesis of (S)-equol. *Tetrahedron* **68**, 5172-5178 (2012).

2 Li, S., Zhu, S.-F., Zhang, C.-M., Song, S. & Zhou, Q.-L. Iridium-Catalyzed Enantioselective Hydrogenation of α,β-Unsaturated Carboxylic Acids. *J. Am. Chem. Soc.* **130**, 8584-8585 (2008).

3 Cheng, X., Zhang, Q., Xie, J.-H., Wang, L.-X. & Zhou, Q.-L. Highly Rigid Diphosphane Ligands with a Large Dihedral Angle Based on a Chiral Spirobifluorene Backbone. *Angew. Chem. Int. Ed.* **44**, 1118-1121 (2005).

4 Beghetto, V. *et al.* A Practical, Enantioselective Synthesis of the Fragrances Canthoxal and Silvial®, and Evaluation of Their Olfactory Activity. *Synthesis* **47**, 272-288 (2015).

5 Cai, Z., Li, S., Gao, Y., Fu, L. & Li, G. Weak, bidentate chelating group assisted cross-coupling of C(sp3)–H bonds in aliphatic acid derivatives with aryltrifluoroborates. *Chem. Commun.* **54**, 12766-12769 (2018).

6 Kato, D.-i., Miyamoto, K. & Ohta, H. Microbial deracemization of α-substituted carboxylic acids: control of the reaction path. *Tetrahedron: Asymmetry* **15**, 2965-2973 (2004).

7 Sugimura, T. *et al.* Structural requirements for substrate in highly enantioselective hydrogenation over the cinchonidine-modified Pd/C. *J. Catal.* **262**, 57-64 (2009).

8 Song, S., Zhu, S.-F., Pu, L.-Y. & Zhou, Q.-L. Iridium-Catalyzed Enantioselective Hydrogenation of Unsaturated Heterocyclic Acids. *Angew. Chem. Int. Ed.* **52**, 6072-6075 (2013).

9 Li, S. *et al.* Enantioselective Hydrogenation of α-Aryloxy and α-Alkoxy α,β-Unsaturated Carboxylic Acids Catalyzed by Chiral Spiro Iridium/Phosphino-Oxazoline Complexes. *J. Am. Chem. Soc.* **132**, 1172-1179 (2010).

10 Dong, K., Li, Y., Wang, Z. & Ding, K. Asymmetric hydrogenation of α-arylacrylic and β-arylbut-3-enoic acids catalyzed by a Rh(i) complex of a monodentate secondary phosphine oxide ligand. *Org. Chem. Front.,* **1**, 155-160 (2014).

11 Li, J. *et al.* Asymmetric Hydrogenation of α-Substituted Acrylic Acids Catalyzed by a Ruthenocenyl Phosphino-oxazoline–Ruthenium Complex. *Org. Lett.* **18**, 2122-2125 (2016).

12 Shiina, I., Nakata, K., Ono, K., Onda, Y.-s. & Itagaki, M. Kinetic Resolution of Racemic α-Arylalkanoic Acids with Achiral Alcohols via the Asymmetric Esterification Using Carboxylic Anhydrides and Acyl-Transfer Catalysts. *J. Am. Chem. Soc.* **132**, 11629-11641 (2010).

13 Iwamoto, T., Okuzono, C., Adak, L., Jin, M. & Nakamura, M. Iron-catalysed enantioselective Suzuki–Miyaura coupling of racemic alkyl bromides. *Chem. Commun.* **55**, 1128-1131 (2019).

14 Zhu, S.-F., Yu, Y.-B., Li, S., Wang, L.-X. & Zhou, Q.-L. Enantioselective Hydrogenation of α-Substituted Acrylic Acids Catalyzed by Iridium Complexes with Chiral Spiro Aminophosphine Ligands. *Angew. Chem. Int. Ed.* **51**, 8872-8875 (2012).

15 Zhong, H., Shevlin, M. & Chirik, P. J. Cobalt-Catalyzed Asymmetric Hydrogenation of α,β-Unsaturated Carboxylic Acids by Homolytic H_2 Cleavage. *J. Am. Chem. Soc.* **142**, 5272-5281 (2020).
